# Supplementary material for: Topographic volume-standardization atlas of the human brain
Source: Brain Struct Funct. 2021 May 7;226(6):1699–711. doi: 10.1007/s00429-021-02280-1 (PMC8203509; doi:10.1007/s00429-021-02280-1)
Supplement: Supplementary file 1 — Supplementary file1 (HTML 31323 KB) [file 429_2021_2280_MOESM1_ESM.html]

 

 

 

 
 
 


 


 Statistical code 

 
 
 
 
 
 
 
 
 
 
 
 
 

 
 
 


 


 

 


 

 

 
 


 

 


 


 

 
  Code     
 
  Show All Code  
  Hide All Code  
  
  Download Rmd  
 
 


 Statistical code 
 Topographic volume-standardization atlas of the human brain 
 Akeret et al. 2020 

 


 
 
 Setup 
 
 
 
  
knitr::opts_chunk$set(message = FALSE, warning = FALSE, error = FALSE)

library(readxl)
library(biostatUZH)
library(dplyr)
library(tableone)
library(knitr)
library(captioner)
library(beeswarm)
library(readr)
library(ggplot2)
library(reshape2)
library(tidyr)
library(ggpubr)
library(graphics)
library(ggbeeswarm)
library(jcolors)
library(pals)
library(ggsci)
library(Hmisc)
library(stringr)

kableone <- function(x, ...) {
  capture.output(x <- print(x))
  knitr::kable(x, ...)
}
  
 
 
 
 
 
 
  
All.Volumes <- read_csv("Encephalic structures_volumes.csv")  
 
 
  Parsed with column specification:
cols(
  .default = col_double(),
  Gender = [31mcol_character()[39m,
  Handedness = [31mcol_character()[39m
)
See spec(...) for full column specifications.  
 
 
  All.Volumes$ID <- as.factor(All.Volumes$ID)
All.Volumes$Gender <- as.factor(All.Volumes$Gender)
All.Volumes$Handedness <- as.factor(All.Volumes$Handedness)
  
 
 
 
 
 
 Baseline Data 
 
 
 
  
Baseline.data <- CreateTableOne(vars = c("Age (years)", "Handedness"), 
               data = All.Volumes, strat = c("Gender"))
Baseline.data <- print(Baseline.data)  
 
 
                           Stratified by Gender
                          f              m             p      test
  n                          14             16                    
  Age (years) (mean (SD)) 37.79 (13.04)  38.31 (16.91)  0.925     
  Handedness = right (%)     14 (100.0)     15 (93.8)   1.000       
 
 
 
 
 
 
  
kable(Baseline.data)  
 
 
 

 
 
 
  
 f 
 m 
 p 
 test 
 
 
 
 
 n 
 14 
 16 
  
  
 
 
 Age (years) (mean (SD)) 
 37.79 (13.04) 
 38.31 (16.91) 
 0.925 
  
 
 
 Handedness = right (%) 
 14 (100.0) 
 15 (93.8) 
 1.000 
  
 
 
 


 
   
  NA  
 
 
 
 
 
 Total encephalic volume (w/o ventricles) 
 
 
 
  
summary(All.Volumes$`Total encephalic volume (without ventricles)`)  
 
 
     Min. 1st Qu.  Median    Mean 3rd Qu.    Max. 
 921292  999649 1108766 1093437 1187487 1274242   
 
 
  Total.encephalic.volume <- CreateTableOne(vars = c("Total encephalic volume (without ventricles)"), 
                                                  data = All.Volumes)
Total.encephalic.volume.stratified.gender <- CreateTableOne(vars = c("Total encephalic volume (without ventricles)"), 
                                                   strata = c("Gender"), data = All.Volumes)
Total.encephalic.volume <- print(Total.encephalic.volume)  
 
 
                                                            
                                                           Overall               
  n                                                                30            
  Total encephalic volume (without ventricles) (mean (SD)) 1093437.27 (111353.18)  
 
 
  Total.encephalic.volume.stratified.gender <- print(Total.encephalic.volume.stratified.gender)  
 
 
                                                            Stratified by Gender
                                                           f                     m                     p      test
  n                                                                14                    16                       
  Total encephalic volume (without ventricles) (mean (SD)) 1024921.71 (89784.56) 1153388.38 (93652.70)  0.001       
 
 
 
 
 
 
  
Total.encephalic.volume.RSD <- round((111353.18/1093437.27)*100,1)
Total.encephalic.volume.stratified.gender.female.RDS <- round((89784.56/1024921.71)*100,1)
Total.encephalic.volume.stratified.gender.male.RSD <- round((93652.70/1153388.38)*100,1)
  
 
 
 
 
 
 
  
kable(Total.encephalic.volume)  
 
 
 

 
 
 
 
 
 
 
  
 Overall 
 
 
 
 
 n 
 30 
 
 
 Total encephalic volume (without ventricles) (mean (SD)) 
 1093437.27 (111353.18) 
 
 
 


 
   
  kable(Total.encephalic.volume.RSD)  
 
 
 

 
 
 
 x 
 
 
 
 
 10.2 
 
 
 


 
   
  kable(Total.encephalic.volume.stratified.gender)  
 
 
 

 
 
 
 
 
 
 
 
 
 
  
 f 
 m 
 p 
 test 
 
 
 
 
 n 
 14 
 16 
  
  
 
 
 Total encephalic volume (without ventricles) (mean (SD)) 
 1024921.71 (89784.56) 
 1153388.38 (93652.70) 
 0.001 
  
 
 
 


 
   
  kable(Total.encephalic.volume.stratified.gender.female.RDS)  
 
 
 

 
 
 
 x 
 
 
 
 
 8.8 
 
 
 


 
   
  kable(Total.encephalic.volume.stratified.gender.male.RSD)  
 
 
 

 
 
 
 x 
 
 
 
 
 8.1 
 
 
 


 
   
  NA  
 
 
 
 
 
 
  
Total.encephalic.volume.plot <-  ggplot(All.Volumes, aes(x= Gender, y = `Total encephalic volume (without ventricles)`))  +
  geom_quasirandom(aes(color = `Age (years)`), alpha = 1, size = 2, shape = 16, position = "dodge") +
  scale_color_continuous(low = "steelblue1", high = "red4") +
  geom_boxplot(aes(fill = Gender), alpha = 0.5, size = 0.3, width = 0.35, outlier.shape = NA, color = "gray30") +
  scale_fill_manual(values = c("chartreuse4", "orangered2")) +
  xlab("Gender") + ylab("") +
  theme_minimal() +
  coord_flip() +
  ggtitle("TOTAL ENCEPHALIC VOLUME (mm3)") +
  theme(plot.title = element_text(hjust = 0.5))
Total.encephalic.volume.plot
ggsave("Total.encephalic.volume.plot.pdf", plot = Total.encephalic.volume.plot, width = 8, height = 6, units = "in", dpi = 600)
  
 
 
   
 
 
 
 
 
 
  
Total.encephalic.volume.Age.plot <-  ggplot(All.Volumes, aes(y=`Total encephalic volume (without ventricles)`, x = `Age (years)`))  +
  geom_point(aes(color = Gender), size = 1.5, alpha = 0.8, shape = 16) +
  geom_smooth(method='lm', alpha = 0.2, colour = "dodgerblue4", size = 0.8, weight = 0.3) +
  geom_smooth(aes(color = Gender, group = Gender), method='lm', se = F, alpha = 0.2,linetype = "longdash", size = 0.3, weight = 0.3) +
  scale_color_manual(values = c("chartreuse4", "orangered2")) +
  stat_cor(method = "pearson", label.y = 1250000, label.x = 65, color = "dodgerblue4") +
  ylab("Volume in mm3") + xlab("Age (in years)") +
  theme_minimal() +
  ggtitle("ABSOLUTE VOLUME encephalon") +
  theme(plot.title = element_text(hjust = 0.5))
Total.encephalic.volume.Age.plot
ggsave("Total.encephalic.volume.Age.plot.pdf", plot = Total.encephalic.volume.Age.plot, width = 8, height = 6, units = "in", dpi = 600)
  
 
 
   
 
 
 
 
 
 Topographic overview 
 
 Absolute Volumes 
 
 
 
  
Frontal.lobe <- All.Volumes$`Total volume frontal pole`+
                       All.Volumes$`Total volume F1`+
                       All.Volumes$`Total volume F2`+
                       All.Volumes$`Total volume F3 orbital`+
                       All.Volumes$`Total volume F3 triangular`+
                       All.Volumes$`Total volume F3 opercular`+
                       All.Volumes$`Total volume anterior orbital`+
                       All.Volumes$`Total volume posterior orbital`+
                       All.Volumes$`Total volume medial orbital`+
                       All.Volumes$`Total volume lateral orbital`+
                       All.Volumes$`Total volume rectus`+
                       All.Volumes$`Total volume rostral`
  
Central.lobe <- (All.Volumes$`Total volume PreC`+
                       All.Volumes$`Total volume PostC`+
                       All.Volumes$`Total volume ParaC lobule`+
                       All.Volumes$`Total volume SubC gyrus`)
  
Parietal.lobe <- (All.Volumes$`Total volume SPL`+
                       All.Volumes$`Total volume SMG`+
                       All.Volumes$`Total volume ANG`+
                       All.Volumes$`Total volume Precuneus`)

Occipital.lobe <- (All.Volumes$`Total volume Cuneus`+
                       All.Volumes$`Total volume O1`+
                       All.Volumes$`Total volume O2`+
                       All.Volumes$`Total volume O3`+
                       All.Volumes$`Total volume occipital pole`+
                       All.Volumes$`Total volume lingual`)

Temporal.lobe <- (All.Volumes$`Total volume fusiform`+
                       All.Volumes$`Total volume T1`+
                       All.Volumes$`Total volume T2`+
                       All.Volumes$`Total volume T3`+
                       All.Volumes$`Total volume Planum temporale`+
                       All.Volumes$`Total volume Planum polare`+
                       All.Volumes$`Total volume temporal pole`)

Insular.lobe <- (All.Volumes$`Total volume short insular gyri`+
                       All.Volumes$`Total volume long insular gyri`)

Limbic.lobe <- (All.Volumes$`Total volume SCA`+
                       All.Volumes$`Total volume PHG`+
                       All.Volumes$`Total volume ant cingulate`+
                       All.Volumes$`Total volume mid cingulate`+
                       All.Volumes$`Total volume post cingulate`+
                       All.Volumes$`Total volume hippocampus` +
                       All.Volumes$`Total volume amygdala`)

Basal.ganglia <- (All.Volumes$`Total volume caudate`+
                       All.Volumes$`Total volume putamen`+
                       All.Volumes$`Total volume pallidum`)

Diencephalon <- (All.Volumes$`Total volume hypothalamus`+
                        All.Volumes$`Total volume thalamus`)

Brainstem <- (All.Volumes$`Total volume brainstem`)

Cerebellum <- (All.Volumes$`Total volume cerebellum`)

Topographic.overview.absolute <- as.data.frame(cbind(Frontal.lobe,
                                       Central.lobe,
                                       Parietal.lobe,
                                       Occipital.lobe,
                                       Temporal.lobe,
                                       Insular.lobe,
                                       Limbic.lobe,
                                       Basal.ganglia,
                                       Diencephalon,
                                       Brainstem,
                                       Cerebellum))

Topographic.overview.absolute$Gender <- All.Volumes$Gender
Topographic.overview.absolute$Age <- All.Volumes$`Age (years)`

Table.topographic.overview.absolute <- CreateTableOne(vars = c("Frontal.lobe","Central.lobe", "Parietal.lobe", "Occipital.lobe", "Temporal.lobe", "Insular.lobe","Limbic.lobe","Basal.ganglia", "Diencephalon","Brainstem","Cerebellum"), 
               data = Topographic.overview.absolute)

Table.topographic.overview.absolute.stratified.gender <- CreateTableOne(vars = c("Frontal.lobe","Central.lobe", "Parietal.lobe", "Occipital.lobe", "Temporal.lobe", "Insular.lobe","Limbic.lobe","Basal.ganglia", "Diencephalon","Brainstem","Cerebellum"), 
               strata = c("Gender"), data = Topographic.overview.absolute)

Table.topographic.overview.absolute <- print(Table.topographic.overview.absolute, contDigits = 10)  
 
 
                              
                             Overall                             
  n                                         30                   
  Frontal.lobe (mean (SD))   199479.6000000000 (24675.6628190333)
  Central.lobe (mean (SD))    81517.1333333333 (9207.9403392916) 
  Parietal.lobe (mean (SD))  129215.8000000000 (16770.1086592001)
  Occipital.lobe (mean (SD))  75910.6000000000 (8884.5868496884) 
  Temporal.lobe (mean (SD))  116476.8666666667 (13867.9503836608)
  Insular.lobe (mean (SD))    24547.4333333333 (3014.6818175256) 
  Limbic.lobe (mean (SD))     75816.2000000000 (9372.2558407689) 
  Basal.ganglia (mean (SD))   22117.1000000000 (2978.7042930639) 
  Diencephalon (mean (SD))    22568.8333333333 (2268.6469016504) 
  Brainstem (mean (SD))       28072.4000000000 (3292.5882450929) 
  Cerebellum (mean (SD))     116732.9666666667 (12623.3422659915)  
 
 
  Table.topographic.overview.absolute.stratified.gender <- print(Table.topographic.overview.absolute.stratified.gender,contDigits = 10)  
 
 
                              Stratified by Gender
                             f                                    m                                    p      test
  n                                         14                                   16                               
  Frontal.lobe (mean (SD))   186347.1428571429 (19261.7710658549) 210970.5000000000 (23533.5837446545)  0.004     
  Central.lobe (mean (SD))    76746.0714285714 (8368.0106308239)   85691.8125000000 (7962.2542303776)   0.006     
  Parietal.lobe (mean (SD))  120829.0000000000 (15805.5035546288) 136554.2500000000 (14287.2790994880)  0.008     
  Occipital.lobe (mean (SD))  70930.2142857143 (5286.0529724429)   80268.4375000000 (9218.7441080207)   0.002     
  Temporal.lobe (mean (SD))  109341.7142857143 (9773.9892290993)  122720.1250000000 (14139.7363241564)  0.006     
  Insular.lobe (mean (SD))    22746.3571428571 (2320.9156882291)   26123.3750000000 (2688.0316435389)   0.001     
  Limbic.lobe (mean (SD))     71384.9285714286 (8462.9316293904)   79693.5625000000 (8566.6449439575)   0.013     
  Basal.ganglia (mean (SD))   21022.8571428571 (2275.7467703234)   23074.5625000000 (3251.1485143715)   0.058     
  Diencephalon (mean (SD))    21697.2857142857 (1746.7271121717)   23331.4375000000 (2444.7618280383)   0.047     
  Brainstem (mean (SD))       26807.6428571429 (3260.5798117499)   29179.0625000000 (2991.0450229254)   0.047     
  Cerebellum (mean (SD))     111616.6428571429 (12960.7820253560) 121209.7500000000 (10801.8819810871)  0.035       
 
 
  write.csv(Table.topographic.overview.absolute, "Table.topographic.overview.absolute.csv")
write.csv(Table.topographic.overview.absolute.stratified.gender, "Table.topographic.overview.absolute.stratified.gender.csv")
  
 
 
 
 
 
 
  
Table.topographic.overview.absolute.RSD <- as.data.frame(Table.topographic.overview.absolute)
Table.topographic.overview.absolute.RSD <- data.frame(do.call('rbind', 
                                                              strsplit(as.character(Table.topographic.overview.absolute.RSD[-1,]),
                                                                       ' (',fixed=TRUE)))
Table.topographic.overview.absolute.RSD <- data.frame(cbind(str_replace_all(Table.topographic.overview.absolute.RSD$X1, "[ ]", ""),
                                                            str_replace_all(Table.topographic.overview.absolute.RSD$X2, "[)]", "")))
Table.topographic.overview.absolute.RSD$X1 <- as.character(Table.topographic.overview.absolute.RSD$X1)
Table.topographic.overview.absolute.RSD$X2 <- as.character(Table.topographic.overview.absolute.RSD$X2)
Table.topographic.overview.absolute.RSD <- as.data.frame(sapply(Table.topographic.overview.absolute.RSD, as.numeric))
Table.topographic.overview.absolute.RSD <- as.data.frame(Table.topographic.overview.absolute.RSD$X2/Table.topographic.overview.absolute.RSD$X1)
Table.topographic.overview.absolute.RSD <- round(Table.topographic.overview.absolute.RSD * 100, 1)


Table.topographic.overview.absolute.stratified.gender.RSD <- as.data.frame(Table.topographic.overview.absolute.stratified.gender)
Table.topographic.overview.absolute.stratified.gender.RSD <- select(Table.topographic.overview.absolute.stratified.gender.RSD, - c(p, test))

Table.topographic.overview.absolute.stratified.gender.RSD.female <- data.frame(do.call('rbind', 
                                                              strsplit(as.character(Table.topographic.overview.absolute.stratified.gender.RSD[-1, "f"]),
                                                                       ' (',fixed=TRUE)))
Table.topographic.overview.absolute.stratified.gender.RSD.female <- data.frame(cbind(str_replace_all(Table.topographic.overview.absolute.stratified.gender.RSD.female$X1, "[ ]", ""),
                                                            str_replace_all(Table.topographic.overview.absolute.stratified.gender.RSD.female$X2, "[)]", "")))
Table.topographic.overview.absolute.stratified.gender.RSD.female$X1 <- as.character(Table.topographic.overview.absolute.stratified.gender.RSD.female$X1)
Table.topographic.overview.absolute.stratified.gender.RSD.female$X2 <- as.character(Table.topographic.overview.absolute.stratified.gender.RSD.female$X2)
Table.topographic.overview.absolute.stratified.gender.RSD.female <- as.data.frame(sapply(Table.topographic.overview.absolute.stratified.gender.RSD.female, as.numeric))
Table.topographic.overview.absolute.stratified.gender.RSD.female <- as.data.frame(Table.topographic.overview.absolute.stratified.gender.RSD.female$X2/Table.topographic.overview.absolute.stratified.gender.RSD.female$X1)
Table.topographic.overview.absolute.stratified.gender.RSD.female <- round(Table.topographic.overview.absolute.stratified.gender.RSD.female * 100, 1)

Table.topographic.overview.absolute.stratified.gender.RSD.male <- data.frame(do.call('rbind', 
                                                              strsplit(as.character(Table.topographic.overview.absolute.stratified.gender.RSD[-1, "m"]),
                                                                       ' (',fixed=TRUE)))
Table.topographic.overview.absolute.stratified.gender.RSD.male <- data.frame(cbind(str_replace_all(Table.topographic.overview.absolute.stratified.gender.RSD.male$X1, "[ ]", ""),
                                                            str_replace_all(Table.topographic.overview.absolute.stratified.gender.RSD.male$X2, "[)]", "")))
Table.topographic.overview.absolute.stratified.gender.RSD.male$X1 <- as.character(Table.topographic.overview.absolute.stratified.gender.RSD.male$X1)
Table.topographic.overview.absolute.stratified.gender.RSD.male$X2 <- as.character(Table.topographic.overview.absolute.stratified.gender.RSD.male$X2)
Table.topographic.overview.absolute.stratified.gender.RSD.male <- as.data.frame(sapply(Table.topographic.overview.absolute.stratified.gender.RSD.male, as.numeric))
Table.topographic.overview.absolute.stratified.gender.RSD.male <- as.data.frame(Table.topographic.overview.absolute.stratified.gender.RSD.male$X2/Table.topographic.overview.absolute.stratified.gender.RSD.male$X1)
Table.topographic.overview.absolute.stratified.gender.RSD.male <- round(Table.topographic.overview.absolute.stratified.gender.RSD.male * 100, 1)
  
 
 
 
 
 
 
  
kable(Table.topographic.overview.absolute)  
 
 
 

 
 
 
  
 Overall 
 
 
 
 
 n 
 30 
 
 
 Frontal.lobe (mean (SD)) 
 199479.6000000000 (24675.6628190333) 
 
 
 Central.lobe (mean (SD)) 
 81517.1333333333 (9207.9403392916) 
 
 
 Parietal.lobe (mean (SD)) 
 129215.8000000000 (16770.1086592001) 
 
 
 Occipital.lobe (mean (SD)) 
 75910.6000000000 (8884.5868496884) 
 
 
 Temporal.lobe (mean (SD)) 
 116476.8666666667 (13867.9503836608) 
 
 
 Insular.lobe (mean (SD)) 
 24547.4333333333 (3014.6818175256) 
 
 
 Limbic.lobe (mean (SD)) 
 75816.2000000000 (9372.2558407689) 
 
 
 Basal.ganglia (mean (SD)) 
 22117.1000000000 (2978.7042930639) 
 
 
 Diencephalon (mean (SD)) 
 22568.8333333333 (2268.6469016504) 
 
 
 Brainstem (mean (SD)) 
 28072.4000000000 (3292.5882450929) 
 
 
 Cerebellum (mean (SD)) 
 116732.9666666667 (12623.3422659915) 
 
 
 


 
   
  kable(Table.topographic.overview.absolute.RSD)  
 
 
 

 
 
 
 
 
 
 Table.topographic.overview.absolute.RSD  X 2/ T  a  b  l  e . t  o  p  o  g  r  a  p  h  i  c . o  v  e  r  v  i  e  w . a  b  s  o  l  u  t  e . R  S  D  X1 
 
 
 
 
 12.4 
 
 
 11.3 
 
 
 13.0 
 
 
 11.7 
 
 
 11.9 
 
 
 12.3 
 
 
 12.4 
 
 
 13.5 
 
 
 10.1 
 
 
 11.7 
 
 
 10.8 
 
 
 


 
   
  kable(Table.topographic.overview.absolute.stratified.gender)  
 
 
 

 
 
 
 
 
 
 
 
 
 
  
 f 
 m 
 p 
 test 
 
 
 
 
 n 
 14 
 16 
  
  
 
 
 Frontal.lobe (mean (SD)) 
 186347.1428571429 (19261.7710658549) 
 210970.5000000000 (23533.5837446545) 
 0.004 
  
 
 
 Central.lobe (mean (SD)) 
 76746.0714285714 (8368.0106308239) 
 85691.8125000000 (7962.2542303776) 
 0.006 
  
 
 
 Parietal.lobe (mean (SD)) 
 120829.0000000000 (15805.5035546288) 
 136554.2500000000 (14287.2790994880) 
 0.008 
  
 
 
 Occipital.lobe (mean (SD)) 
 70930.2142857143 (5286.0529724429) 
 80268.4375000000 (9218.7441080207) 
 0.002 
  
 
 
 Temporal.lobe (mean (SD)) 
 109341.7142857143 (9773.9892290993) 
 122720.1250000000 (14139.7363241564) 
 0.006 
  
 
 
 Insular.lobe (mean (SD)) 
 22746.3571428571 (2320.9156882291) 
 26123.3750000000 (2688.0316435389) 
 0.001 
  
 
 
 Limbic.lobe (mean (SD)) 
 71384.9285714286 (8462.9316293904) 
 79693.5625000000 (8566.6449439575) 
 0.013 
  
 
 
 Basal.ganglia (mean (SD)) 
 21022.8571428571 (2275.7467703234) 
 23074.5625000000 (3251.1485143715) 
 0.058 
  
 
 
 Diencephalon (mean (SD)) 
 21697.2857142857 (1746.7271121717) 
 23331.4375000000 (2444.7618280383) 
 0.047 
  
 
 
 Brainstem (mean (SD)) 
 26807.6428571429 (3260.5798117499) 
 29179.0625000000 (2991.0450229254) 
 0.047 
  
 
 
 Cerebellum (mean (SD)) 
 111616.6428571429 (12960.7820253560) 
 121209.7500000000 (10801.8819810871) 
 0.035 
  
 
 
 


 
   
  kable(Table.topographic.overview.absolute.stratified.gender.RSD.female)  
 
 
 

 
 
 
 
 
 
 Table.topographic.overview.absolute.stratified.gender.RSD.female  X 2/ T  a  b  l  e . t  o  p  o  g  r  a  p  h  i  c . o  v  e  r  v  i  e  w . a  b  s  o  l  u  t  e . s  t  r  a  t  i  f  i  e  d . g  e  n  d  e  r . R  S  D . f  e  m  a  l  e  X1 
 
 
 
 
 10.3 
 
 
 10.9 
 
 
 13.1 
 
 
 7.5 
 
 
 8.9 
 
 
 10.2 
 
 
 11.9 
 
 
 10.8 
 
 
 8.1 
 
 
 12.2 
 
 
 11.6 
 
 
 


 
   
  kable(Table.topographic.overview.absolute.stratified.gender.RSD.male)  
 
 
 

 
 
 
 
 
 
 Table.topographic.overview.absolute.stratified.gender.RSD.male  X 2/ T  a  b  l  e . t  o  p  o  g  r  a  p  h  i  c . o  v  e  r  v  i  e  w . a  b  s  o  l  u  t  e . s  t  r  a  t  i  f  i  e  d . g  e  n  d  e  r . R  S  D . m  a  l  e  X1 
 
 
 
 
 11.2 
 
 
 9.3 
 
 
 10.5 
 
 
 11.5 
 
 
 11.5 
 
 
 10.3 
 
 
 10.7 
 
 
 14.1 
 
 
 10.5 
 
 
 10.3 
 
 
 8.9 
 
 
 


 
   
  NA  
 
 
 
 
 
 
  
Topographic.overview.absolute <- select(Topographic.overview.absolute, - c(Gender, Age))
Topographic.overview.absolute.plotdata <- gather(Topographic.overview.absolute, "anatomical.structure", "relative.volume")
Topographic.overview.absolute.plotdata$Gender <- All.Volumes$Gender
Topographic.overview.absolute.plotdata$Age <- All.Volumes$`Age (years)`

Topographic.overview.absolute.plotdata$Gender <- factor(Topographic.overview.absolute.plotdata$Gender, levels = c("f", "m"), c("f", "m"))
Topographic.overview.absolute.plotdata$anatomical.structure <- factor(Topographic.overview.absolute.plotdata$anatomical.structure, levels = rev(c("Frontal.lobe", "Central.lobe", "Parietal.lobe", "Occipital.lobe", "Temporal.lobe", "Insular.lobe", "Limbic.lobe", "Basal.ganglia", "Diencephalon", "Brainstem", "Cerebellum")), rev(c("Frontal lobe", "Central lobe", "Parietal lobe", "Occipital lobe", "Temporal lobe", "Insular lobe", "Limbic lobe", "Basal ganglia", "Diencephalon", "Brainstem", "Cerebellum")))

Topographic.overview.absolute.plot <-  ggplot(Topographic.overview.absolute.plotdata, aes(x=anatomical.structure, y = relative.volume))  +
  stat_summary(alpha = 0.3, fun = mean, geom = "bar", width = 0.3, fill = "gray50") + 
  geom_boxplot(aes(fill = Gender), alpha = 0.5, width = 0.4, size = 0.2, position = position_dodge(width = 0.6), 
               outlier.shape = NA, color = "gray30") +
  scale_fill_manual(values = c("chartreuse4", "orangered2")) +
  geom_quasirandom(aes(color = Age), size = 0.7, alpha = 0.8, shape = 16, position = "dodge") +
  scale_color_continuous(low = "steelblue1", high = "red4") +
  xlab("") + ylab("Absolute volume (in %)") +
  theme_minimal() +
  coord_flip() +
  ggtitle("TOPOGRAPHIC OVERVIEW") +
  theme(plot.title = element_text(hjust = 0.5))

Topographic.overview.absolute.plot
ggsave("Topographic.overview.absolute.plot.pdf", plot = Topographic.overview.absolute.plot, width = 12, height = 6, units = "in", dpi = 600)
  
 
 
   
 
 
 
 
 
 
  
Topographic.overview.absolute$Gender <- All.Volumes$Gender
Topographic.overview.absolute$Age <- All.Volumes$`Age (years)`

Total.frontal.lobe.Age.plot <-  ggplot(Topographic.overview.absolute, aes(y=Frontal.lobe, x = Age))  +
  geom_point(aes(color = Gender), size = 1.5, alpha = 1, shape = 16) +
  scale_color_manual(values = c("chartreuse4", "orangered2")) +
  geom_smooth(method='lm', alpha = 0.2, colour = "dodgerblue4", size = 0.8, weight = 0.3) +
  geom_smooth(aes(color = Gender), method='lm', se = F, alpha = 0.2, linetype = "longdash", size = 0.3, weight = 0.3) +
  stat_cor(method = "pearson", label.y = 230000, label.x = 70, color = "dodgerblue4") +
  ylab("Volume in mm3") + xlab("Age (in years)") +
  theme_minimal() +
  ggtitle("ABSOLUTE VOLUME frontal lobe") +
  theme(plot.title = element_text(hjust = 0.5))
Total.frontal.lobe.Age.plot
ggsave("TO.Total.frontal.lobe.Age.plot.pdf", plot = Total.frontal.lobe.Age.plot, width = 8, height = 6, units = "in", dpi = 600)  
 
 
   
 
 
  
Total.central.lobe.Age.plot <-  ggplot(Topographic.overview.absolute, aes(y=Central.lobe, x = Age))  +
  geom_point(aes(color = Gender), size = 1.5, alpha = 1, shape = 16) +
  scale_color_manual(values = c("chartreuse4", "orangered2")) +
  geom_smooth(method='lm', alpha = 0.2, colour = "dodgerblue4", size = 0.8, weight = 0.3) +
  geom_smooth(aes(color = Gender), method='lm', se = F, alpha = 0.2, linetype = "longdash", size = 0.3, weight = 0.3) +
  stat_cor(method = "pearson", label.y = 97000, label.x = 70, color = "dodgerblue4") +
  ylab("Volume in mm3") + xlab("Age (in years)") +
  theme_minimal() +
  ggtitle("ABSOLUTE VOLUME central lobe") +
  theme(plot.title = element_text(hjust = 0.5))
Total.central.lobe.Age.plot
ggsave("TO.Total.central.lobe.Age.plot.pdf", plot = Total.central.lobe.Age.plot, width = 8, height = 6, units = "in", dpi = 600)  
 
 
   
 
 
  
Total.parietal.lobe.Age.plot <-  ggplot(Topographic.overview.absolute, aes(y=Parietal.lobe, x = Age))  +
  geom_point(aes(color = Gender), size = 1.5, alpha = 1, shape = 16) +
  scale_color_manual(values = c("chartreuse4", "orangered2")) +
  geom_smooth(method='lm', alpha = 0.2, colour = "dodgerblue4", size = 0.8, weight = 0.3) +
  geom_smooth(aes(color = Gender), method='lm', se = F, alpha = 0.2, linetype = "longdash", size = 0.3, weight = 0.3) +
  stat_cor(method = "pearson", label.y = 145000, label.x = 70, color = "dodgerblue4") +
  ylab("Volume in mm3") + xlab("Age (in years)") +
  theme_minimal() +
  ggtitle("ABSOLUTE VOLUME parietal lobe") +
  theme(plot.title = element_text(hjust = 0.5))
Total.parietal.lobe.Age.plot
ggsave("TO.Total.parietal.lobe.Age.plot.pdf", plot = Total.parietal.lobe.Age.plot, width = 8, height = 6, units = "in", dpi = 600)  
 
 
   
 
 
  
Total.occipital.lobe.Age.plot <-  ggplot(Topographic.overview.absolute, aes(y=Occipital.lobe, x = Age))  +
  geom_point(aes(color = Gender), size = 1.5, alpha = 1, shape = 16) +
  scale_color_manual(values = c("chartreuse4", "orangered2")) +
  geom_smooth(method='lm', alpha = 0.2, colour = "dodgerblue4", size = 0.8, weight = 0.3) +
  geom_smooth(aes(color = Gender), method='lm', se = F, alpha = 0.2, linetype = "longdash", size = 0.3, weight = 0.3) +
  stat_cor(method = "pearson", label.y = 86000, label.x = 70, color = "dodgerblue4") +
  ylab("Volume in mm3") + xlab("Age (in years)") +
  theme_minimal() +
  ggtitle("ABSOLUTE VOLUME occipital lobe") +
  theme(plot.title = element_text(hjust = 0.5))
Total.occipital.lobe.Age.plot
ggsave("TO.Total.occipital.lobe.Age.plot.pdf", plot = Total.occipital.lobe.Age.plot, width = 8, height = 6, units = "in", dpi = 600)  
 
 
   
 
 
  
Total.temporal.lobe.Age.plot <-  ggplot(Topographic.overview.absolute, aes(y=Temporal.lobe, x = Age))  +
  geom_point(aes(color = Gender), size = 1.5, alpha = 1, shape = 16) +
  scale_color_manual(values = c("chartreuse4", "orangered2")) +
  geom_smooth(method='lm', alpha = 0.2, colour = "dodgerblue4", size = 0.8, weight = 0.3) +
  geom_smooth(aes(color = Gender), method='lm', se = F, alpha = 0.2, linetype = "longdash", size = 0.3, weight = 0.3) +
  stat_cor(method = "pearson", label.y = 86000, label.x = 70, color = "dodgerblue4") +
  ylab("Volume in mm3") + xlab("Age (in years)") +
  theme_minimal() +
  ggtitle("ABSOLUTE VOLUME temporal lobe") +
  theme(plot.title = element_text(hjust = 0.5))
Total.temporal.lobe.Age.plot
ggsave("TO.Total.temporal.lobe.Age.plot.pdf", plot = Total.temporal.lobe.Age.plot, width = 8, height = 6, units = "in", dpi = 600)  
 
 
   
 
 
  
Total.insular.lobe.Age.plot <-  ggplot(Topographic.overview.absolute, aes(y=Insular.lobe, x = Age))  +
  geom_point(aes(color = Gender), size = 1.5, alpha = 1, shape = 16) +
  scale_color_manual(values = c("chartreuse4", "orangered2")) +
  geom_smooth(method='lm', alpha = 0.2, colour = "dodgerblue4", size = 0.8, weight = 0.3) +
  geom_smooth(aes(color = Gender), method='lm', se = F, alpha = 0.2, linetype = "longdash", size = 0.3, weight = 0.3) +
  stat_cor(method = "pearson", label.y = 29000, label.x = 70, color = "dodgerblue4") +
  ylab("Volume in mm3") + xlab("Age (in years)") +
  theme_minimal() +
  ggtitle("ABSOLUTE VOLUME insular lobe") +
  theme(plot.title = element_text(hjust = 0.5))
Total.insular.lobe.Age.plot
ggsave("TO.Total.insular.lobe.Age.plot.pdf", plot = Total.insular.lobe.Age.plot, width = 8, height = 6, units = "in", dpi = 600)  
 
 
   
 
 
  
Total.limbic.lobe.Age.plot <-  ggplot(Topographic.overview.absolute, aes(y=Limbic.lobe, x = Age))  +
  geom_point(aes(color = Gender), size = 1.5, alpha = 1, shape = 16) +
  scale_color_manual(values = c("chartreuse4", "orangered2")) +
  geom_smooth(method='lm', alpha = 0.2, colour = "dodgerblue4", size = 0.8, weight = 0.3) +
  geom_smooth(aes(color = Gender), method='lm', se = F, alpha = 0.2, linetype = "longdash", size = 0.3, weight = 0.3) +
  stat_cor(method = "pearson", label.y = 82000, label.x = 70, color = "dodgerblue4") +
  ylab("Volume in mm3") + xlab("Age (in years)") +
  theme_minimal() +
  ggtitle("ABSOLUTE VOLUME limbic lobe") +
  theme(plot.title = element_text(hjust = 0.5))
Total.limbic.lobe.Age.plot
ggsave("TO.Total.limbic.lobe.Age.plot.pdf", plot = Total.limbic.lobe.Age.plot, width = 8, height = 6, units = "in", dpi = 600)  
 
 
   
 
 
  
Total.Basal.ganglia.Age.plot <-  ggplot(Topographic.overview.absolute, aes(y=Basal.ganglia, x = Age))  +
  geom_point(aes(color = Gender), size = 1.5, alpha = 1, shape = 16) +
  scale_color_manual(values = c("chartreuse4", "orangered2")) +
  geom_smooth(method='lm', alpha = 0.2, colour = "dodgerblue4", size = 0.8, weight = 0.3) +
  geom_smooth(aes(color = Gender), method='lm', se = F, alpha = 0.2, linetype = "longdash", size = 0.3, weight = 0.3) +
  stat_cor(method = "pearson", label.y = 24000, label.x = 70, color = "dodgerblue4") +
  ylab("Volume in mm3") + xlab("Age (in years)") +
  theme_minimal() +
  ggtitle("ABSOLUTE VOLUME basal ganglia") +
  theme(plot.title = element_text(hjust = 0.5))
Total.Basal.ganglia.Age.plot
ggsave("TO.Total.Basal.ganglia.Age.plot.pdf", plot = Total.Basal.ganglia.Age.plot, width = 8, height = 6, units = "in", dpi = 600)  
 
 
   
 
 
  
Total.Diencephalon.Age.plot <-  ggplot(Topographic.overview.absolute, aes(y=Diencephalon, x = Age))  +
  geom_point(aes(color = Gender), size = 1.5, alpha = 1, shape = 16) +
  scale_color_manual(values = c("chartreuse4", "orangered2")) +
  geom_smooth(method='lm', alpha = 0.2, colour = "dodgerblue4", size = 0.8, weight = 0.3) +
  geom_smooth(aes(color = Gender), method='lm', se = F, alpha = 0.2, linetype = "longdash", size = 0.3, weight = 0.3) +
  stat_cor(method = "pearson", label.y = 25000, label.x = 70, color = "dodgerblue4") +
  ylab("Volume in mm3") + xlab("Age (in years)") +
  theme_minimal() +
  ggtitle("ABSOLUTE VOLUME diencephalon") +
  theme(plot.title = element_text(hjust = 0.5))
Total.Diencephalon.Age.plot
ggsave("TO.Total.Diencephalon.Age.plot.pdf", plot = Total.Diencephalon.Age.plot, width = 8, height = 6, units = "in", dpi = 600)  
 
 
   
 
 
  
Total.Brainstem.Age.plot <-  ggplot(Topographic.overview.absolute, aes(y=Brainstem, x = Age))  +
  geom_point(aes(color = Gender), size = 1.5, alpha = 1, shape = 16) +
  scale_color_manual(values = c("chartreuse4", "orangered2")) +
  geom_smooth(method='lm', alpha = 0.2, colour = "dodgerblue4", size = 0.8, weight = 0.3) +
  geom_smooth(aes(color = Gender), method='lm', se = F, alpha = 0.2, linetype = "longdash", size = 0.3, weight = 0.3) +
  stat_cor(method = "pearson", label.y = 33000, label.x = 70, color = "dodgerblue4") +
  ylab("Volume in mm3") + xlab("Age (in years)") +
  theme_minimal() +
  ggtitle("ABSOLUTE VOLUME brainstem") +
  theme(plot.title = element_text(hjust = 0.5))
Total.Brainstem.Age.plot
ggsave("TO.Total.Brainstem.Age.plot.pdf", plot = Total.Brainstem.Age.plot, width = 8, height = 6, units = "in", dpi = 600)  
 
 
   
 
 
  
Total.Cerebellum.Age.plot <-  ggplot(Topographic.overview.absolute, aes(y=Cerebellum, x = Age))  +
  geom_point(aes(color = Gender), size = 1.5, alpha = 1, shape = 16) +
  scale_color_manual(values = c("chartreuse4", "orangered2")) +
  geom_smooth(method='lm', alpha = 0.2, colour = "dodgerblue4", size = 0.8, weight = 0.3) +
  geom_smooth(aes(color = Gender), method='lm', se = F, alpha = 0.2, linetype = "longdash", size = 0.3, weight = 0.3) +
  stat_cor(method = "pearson", label.y = 122000, label.x = 70, color = "dodgerblue4") +
  ylab("Volume in mm3") + xlab("Age (in years)") +
  theme_minimal() +
  ggtitle("ABSOLUTE VOLUME cerebellum") +
  theme(plot.title = element_text(hjust = 0.5))
Total.Cerebellum.Age.plot
ggsave("TO.Total.Cerebellum.Age.plot.pdf", plot = Total.Cerebellum.Age.plot, width = 8, height = 6, units = "in", dpi = 600)
  
 
 
   
 
 
 
 
 
 Relative Volumes 
 
 
 
  
Topographic.overview.absolute <- select(Topographic.overview.absolute, - c(Gender, Age))
Topographic.overview.relative1 <- (100 * (Topographic.overview.absolute/All.Volumes$`Total encephalic volume (without ventricles)`))

Topographic.overview.relative2 <- CreateTableOne(vars = c("Frontal.lobe","Central.lobe", "Parietal.lobe", "Occipital.lobe", "Temporal.lobe", "Insular.lobe","Limbic.lobe","Basal.ganglia", "Diencephalon","Brainstem","Cerebellum"), 
               data = Topographic.overview.relative1)
Topographic.overview.relative1$Gender <- All.Volumes$Gender
Topographic.overview.relative.stratified.gender <- CreateTableOne(vars = c("Frontal.lobe","Central.lobe", "Parietal.lobe", "Occipital.lobe", "Temporal.lobe", "Insular.lobe","Limbic.lobe","Basal.ganglia", "Diencephalon","Brainstem","Cerebellum"), 
                strata = c("Gender"), data = Topographic.overview.relative1)

Topographic.overview.relative2 <- print(Topographic.overview.relative2, quote = FALSE, contDigits = 10)  
 
 
                              
                             Overall                     
  n                                     30               
  Frontal.lobe (mean (SD))   18.2262527269 (0.9597402604)
  Central.lobe (mean (SD))    7.4652325684 (0.5232606277)
  Parietal.lobe (mean (SD))  11.8153299902 (0.8890245686)
  Occipital.lobe (mean (SD))  6.9466013623 (0.4663487454)
  Temporal.lobe (mean (SD))  10.6576458547 (0.7070314916)
  Insular.lobe (mean (SD))    2.2473539433 (0.1834431574)
  Limbic.lobe (mean (SD))     6.9273443947 (0.3816087377)
  Basal.ganglia (mean (SD))   2.0288505705 (0.2308767019)
  Diencephalon (mean (SD))    2.0690086186 (0.1329050351)
  Brainstem (mean (SD))       2.5737214227 (0.2369790952)
  Cerebellum (mean (SD))     10.7318266204 (1.1655208385)  
 
 
  Topographic.overview.relative.stratified.gender <- print(Topographic.overview.relative.stratified.gender, quote = FALSE, contDigits = 10)  
 
 
                              Stratified by Gender
                             f                            m                            p      test
  n                                     14                           16                           
  Frontal.lobe (mean (SD))   18.1736795014 (0.7427106379) 18.2722542992 (1.1392494000)  0.784     
  Central.lobe (mean (SD))    7.4860247454 (0.4299043550)  7.4470394134 (0.6069744522)  0.843     
  Parietal.lobe (mean (SD))  11.7665136761 (0.7842958817) 11.8580442651 (0.9953713153)  0.784     
  Occipital.lobe (mean (SD))  6.9414616026 (0.4870496255)  6.9510986521 (0.4634964636)  0.956     
  Temporal.lobe (mean (SD))  10.6703456390 (0.3193451746) 10.6465335435 (0.9369070117)  0.929     
  Insular.lobe (mean (SD))    2.2226928694 (0.1710334769)  2.2689323829 (0.1965780744)  0.501     
  Limbic.lobe (mean (SD))     6.9547003947 (0.3570504302)  6.9034078946 (0.4120018041)  0.720     
  Basal.ganglia (mean (SD))   2.0548417724 (0.1820952611)  2.0061082688 (0.2704344168)  0.573     
  Diencephalon (mean (SD))    2.1204998650 (0.1057097871)  2.0239537780 (0.1408028261)  0.045     
  Brainstem (mean (SD))       2.6191165363 (0.2600478612)  2.5340006983 (0.2153131486)  0.335     
  Cerebellum (mean (SD))     10.9282585511 (1.2213015050) 10.5599486811 (1.1252066685)  0.397       
 
 
 
 
 
 
  
Table.topographic.overview.relative.RSD <- as.data.frame(Topographic.overview.relative2)
Table.topographic.overview.relative.RSD <- data.frame(do.call('rbind', 
                                                              strsplit(as.character(Table.topographic.overview.relative.RSD[-1,]),
                                                                       ' (',fixed=TRUE)))
Table.topographic.overview.relative.RSD <- data.frame(cbind(str_replace_all(Table.topographic.overview.relative.RSD$X1, "[ ]", ""),
                                                            str_replace_all(Table.topographic.overview.relative.RSD$X2, "[)]", "")))
Table.topographic.overview.relative.RSD$X1 <- as.character(Table.topographic.overview.relative.RSD$X1)
Table.topographic.overview.relative.RSD$X2 <- as.character(Table.topographic.overview.relative.RSD$X2)
Table.topographic.overview.relative.RSD <- as.data.frame(sapply(Table.topographic.overview.relative.RSD, as.numeric))
Table.topographic.overview.relative.RSD <- as.data.frame(Table.topographic.overview.relative.RSD$X2/Table.topographic.overview.relative.RSD$X1)
Table.topographic.overview.relative.RSD <- round(Table.topographic.overview.relative.RSD*100, 1)

Table.topographic.overview.relative.stratified.gender.RSD <- as.data.frame(Topographic.overview.relative.stratified.gender)
Table.topographic.overview.relative.stratified.gender.RSD <- select(Table.topographic.overview.relative.stratified.gender.RSD, - c(p, test))

Table.topographic.overview.relative.stratified.gender.RSD.female <- data.frame(do.call('rbind', 
                                                              strsplit(as.character(Table.topographic.overview.relative.stratified.gender.RSD[-1, "f"]),
                                                                       ' (',fixed=TRUE)))
Table.topographic.overview.relative.stratified.gender.RSD.female <- data.frame(cbind(str_replace_all(Table.topographic.overview.relative.stratified.gender.RSD.female$X1, "[ ]", ""),
                                                            str_replace_all(Table.topographic.overview.relative.stratified.gender.RSD.female$X2, "[)]", "")))
Table.topographic.overview.relative.stratified.gender.RSD.female$X1 <- as.character(Table.topographic.overview.relative.stratified.gender.RSD.female$X1)
Table.topographic.overview.relative.stratified.gender.RSD.female$X2 <- as.character(Table.topographic.overview.relative.stratified.gender.RSD.female$X2)
Table.topographic.overview.relative.stratified.gender.RSD.female <- as.data.frame(sapply(Table.topographic.overview.relative.stratified.gender.RSD.female, as.numeric))
Table.topographic.overview.relative.stratified.gender.RSD.female <- as.data.frame(Table.topographic.overview.relative.stratified.gender.RSD.female$X2/Table.topographic.overview.relative.stratified.gender.RSD.female$X1)
Table.topographic.overview.relative.stratified.gender.RSD.female <- round(Table.topographic.overview.relative.stratified.gender.RSD.female*100, 1)

Table.topographic.overview.relative.stratified.gender.RSD.male <- data.frame(do.call('rbind', 
                                                              strsplit(as.character(Table.topographic.overview.relative.stratified.gender.RSD[-1, "m"]),
                                                                       ' (',fixed=TRUE)))
Table.topographic.overview.relative.stratified.gender.RSD.male <- data.frame(cbind(str_replace_all(Table.topographic.overview.relative.stratified.gender.RSD.male$X1, "[ ]", ""),
                                                            str_replace_all(Table.topographic.overview.relative.stratified.gender.RSD.male$X2, "[)]", "")))
Table.topographic.overview.relative.stratified.gender.RSD.male$X1 <- as.character(Table.topographic.overview.relative.stratified.gender.RSD.male$X1)
Table.topographic.overview.relative.stratified.gender.RSD.male$X2 <- as.character(Table.topographic.overview.relative.stratified.gender.RSD.male$X2)
Table.topographic.overview.relative.stratified.gender.RSD.male <- as.data.frame(sapply(Table.topographic.overview.relative.stratified.gender.RSD.male, as.numeric))
Table.topographic.overview.relative.stratified.gender.RSD.male <- as.data.frame(Table.topographic.overview.relative.stratified.gender.RSD.male$X2/Table.topographic.overview.relative.stratified.gender.RSD.male$X1)
Table.topographic.overview.relative.stratified.gender.RSD.male <- round(Table.topographic.overview.relative.stratified.gender.RSD.male*100, 1)
  
 
 
 
 
 
 
  
kable(Topographic.overview.relative2)  
 
 
 

 
 
 
  
 Overall 
 
 
 
 
 n 
 30 
 
 
 Frontal.lobe (mean (SD)) 
 18.2262527269 (0.9597402604) 
 
 
 Central.lobe (mean (SD)) 
 7.4652325684 (0.5232606277) 
 
 
 Parietal.lobe (mean (SD)) 
 11.8153299902 (0.8890245686) 
 
 
 Occipital.lobe (mean (SD)) 
 6.9466013623 (0.4663487454) 
 
 
 Temporal.lobe (mean (SD)) 
 10.6576458547 (0.7070314916) 
 
 
 Insular.lobe (mean (SD)) 
 2.2473539433 (0.1834431574) 
 
 
 Limbic.lobe (mean (SD)) 
 6.9273443947 (0.3816087377) 
 
 
 Basal.ganglia (mean (SD)) 
 2.0288505705 (0.2308767019) 
 
 
 Diencephalon (mean (SD)) 
 2.0690086186 (0.1329050351) 
 
 
 Brainstem (mean (SD)) 
 2.5737214227 (0.2369790952) 
 
 
 Cerebellum (mean (SD)) 
 10.7318266204 (1.1655208385) 
 
 
 


 
   
  kable(Table.topographic.overview.relative.RSD)  
 
 
 

 
 
 
 
 
 
 Table.topographic.overview.relative.RSD  X 2/ T  a  b  l  e . t  o  p  o  g  r  a  p  h  i  c . o  v  e  r  v  i  e  w . r  e  l  a  t  i  v  e . R  S  D  X1 
 
 
 
 
 5.3 
 
 
 7.0 
 
 
 7.5 
 
 
 6.7 
 
 
 6.6 
 
 
 8.2 
 
 
 5.5 
 
 
 11.4 
 
 
 6.4 
 
 
 9.2 
 
 
 10.9 
 
 
 


 
   
  kable(Topographic.overview.relative.stratified.gender)  
 
 
 

 
 
 
 
 
 
 
 
 
 
  
 f 
 m 
 p 
 test 
 
 
 
 
 n 
 14 
 16 
  
  
 
 
 Frontal.lobe (mean (SD)) 
 18.1736795014 (0.7427106379) 
 18.2722542992 (1.1392494000) 
 0.784 
  
 
 
 Central.lobe (mean (SD)) 
 7.4860247454 (0.4299043550) 
 7.4470394134 (0.6069744522) 
 0.843 
  
 
 
 Parietal.lobe (mean (SD)) 
 11.7665136761 (0.7842958817) 
 11.8580442651 (0.9953713153) 
 0.784 
  
 
 
 Occipital.lobe (mean (SD)) 
 6.9414616026 (0.4870496255) 
 6.9510986521 (0.4634964636) 
 0.956 
  
 
 
 Temporal.lobe (mean (SD)) 
 10.6703456390 (0.3193451746) 
 10.6465335435 (0.9369070117) 
 0.929 
  
 
 
 Insular.lobe (mean (SD)) 
 2.2226928694 (0.1710334769) 
 2.2689323829 (0.1965780744) 
 0.501 
  
 
 
 Limbic.lobe (mean (SD)) 
 6.9547003947 (0.3570504302) 
 6.9034078946 (0.4120018041) 
 0.720 
  
 
 
 Basal.ganglia (mean (SD)) 
 2.0548417724 (0.1820952611) 
 2.0061082688 (0.2704344168) 
 0.573 
  
 
 
 Diencephalon (mean (SD)) 
 2.1204998650 (0.1057097871) 
 2.0239537780 (0.1408028261) 
 0.045 
  
 
 
 Brainstem (mean (SD)) 
 2.6191165363 (0.2600478612) 
 2.5340006983 (0.2153131486) 
 0.335 
  
 
 
 Cerebellum (mean (SD)) 
 10.9282585511 (1.2213015050) 
 10.5599486811 (1.1252066685) 
 0.397 
  
 
 
 


 
   
  kable(Table.topographic.overview.relative.stratified.gender.RSD.female)  
 
 
 

 
 
 
 
 
 
 Table.topographic.overview.relative.stratified.gender.RSD.female  X 2/ T  a  b  l  e . t  o  p  o  g  r  a  p  h  i  c . o  v  e  r  v  i  e  w . r  e  l  a  t  i  v  e . s  t  r  a  t  i  f  i  e  d . g  e  n  d  e  r . R  S  D . f  e  m  a  l  e  X1 
 
 
 
 
 4.1 
 
 
 5.7 
 
 
 6.7 
 
 
 7.0 
 
 
 3.0 
 
 
 7.7 
 
 
 5.1 
 
 
 8.9 
 
 
 5.0 
 
 
 9.9 
 
 
 11.2 
 
 
 


 
   
  kable(Table.topographic.overview.relative.stratified.gender.RSD.male)  
 
 
 

 
 
 
 
 
 
 Table.topographic.overview.relative.stratified.gender.RSD.male  X 2/ T  a  b  l  e . t  o  p  o  g  r  a  p  h  i  c . o  v  e  r  v  i  e  w . r  e  l  a  t  i  v  e . s  t  r  a  t  i  f  i  e  d . g  e  n  d  e  r . R  S  D . m  a  l  e  X1 
 
 
 
 
 6.2 
 
 
 8.2 
 
 
 8.4 
 
 
 6.7 
 
 
 8.8 
 
 
 8.7 
 
 
 6.0 
 
 
 13.5 
 
 
 7.0 
 
 
 8.5 
 
 
 10.7 
 
 
 


 
   
  NA  
 
 
 
 
 
 
  
Topographic.overview.relative1 <- select(Topographic.overview.relative1, - c(Gender))
Topographic.overview.relative.plotdata <- gather(Topographic.overview.relative1, "anatomical.structure", "relative.volume")
Topographic.overview.relative.plotdata$Gender <- All.Volumes$Gender
Topographic.overview.relative.plotdata$Age <- All.Volumes$`Age (years)`

Topographic.overview.relative.plotdata$Gender <- factor(Topographic.overview.relative.plotdata$Gender, levels = c("f", "m"), c("f", "m"))
Topographic.overview.relative.plotdata$anatomical.structure <- factor(Topographic.overview.relative.plotdata$anatomical.structure, levels = rev(c("Frontal.lobe", "Central.lobe", "Parietal.lobe", "Occipital.lobe", "Temporal.lobe", "Insular.lobe", "Limbic.lobe", "Basal.ganglia", "Diencephalon", "Brainstem", "Cerebellum")), rev(c("Frontal lobe", "Central lobe", "Parietal lobe", "Occipital lobe", "Temporal lobe", "Insular lobe", "Limbic lobe", "Basal ganglia", "Diencephalon", "Brainstem", "Cerebellum")))

Topographic.overview.relative.plot <-  ggplot(Topographic.overview.relative.plotdata, aes(x=anatomical.structure, y = relative.volume))  +
  stat_summary(alpha = 0.3, fun = mean, geom = "bar", width = 0.3, fill = "gray50") + 
  geom_boxplot(aes(fill = Gender), alpha = 0.5, width = 0.4, size = 0.2, position = position_dodge(width = 0.6), 
               outlier.shape = NA, color = "gray30") +
  scale_fill_manual(values = c("chartreuse4", "orangered2")) +
  geom_quasirandom(aes(color = Age), size = 0.7, alpha = 0.8, shape = 16, position = "dodge") +
  scale_color_continuous(low = "steelblue1", high = "red4") +
  xlab("") + ylab("Relative volume (in %)") +
  theme_minimal() +
  coord_flip() +
  ggtitle("TOPOGRAPHIC OVERVIEW") +
  theme(plot.title = element_text(hjust = 0.5))

Topographic.overview.relative.plot
ggsave("Topographic.overview.relative.plot.pdf", plot = Topographic.overview.relative.plot, width = 12, height = 6, units = "in", dpi = 600)
  
 
 
   
 
 
 
 
 
 
  
Topographic.overview.relative1$Gender <- All.Volumes$Gender
Topographic.overview.relative1$Age <- All.Volumes$`Age (years)`

Relative.frontal.lobe.Age.plot <-  ggplot(Topographic.overview.relative1, aes(y=Frontal.lobe, x = Age))  +
  geom_point(aes(color = Gender), size = 1.5, alpha = 1, shape = 16) +
  scale_color_manual(values = c("chartreuse4", "orangered2")) +
  geom_smooth(method='lm', alpha = 0.2, colour = "dodgerblue4", size = 0.8, weight = 0.3) +
  geom_smooth(aes(color = Gender), method='lm', se = F, alpha = 0.2, linetype = "longdash", size = 0.3, weight = 0.3) +
  stat_cor(method = "pearson", label.y = 19.3, label.x = 70, color = "dodgerblue4") +
  ylab("Relative Volume (in %)") + xlab("Age (in years)") +
  theme_minimal() +
  ggtitle("RELATIVE VOLUME frontal lobe") +
  theme(plot.title = element_text(hjust = 0.5))
Relative.frontal.lobe.Age.plot
ggsave("TOR.Relative.frontal.lobe.Age.plot.pdf", plot = Relative.frontal.lobe.Age.plot, width = 8, height = 6, units = "in", dpi = 600)  
 
 
   
 
 
  
Relative.Central.lobe.Age.plot <-  ggplot(Topographic.overview.relative1, aes(y=Central.lobe, x = Age))  +
  geom_point(aes(color = Gender), size = 1.5, alpha = 1, shape = 16) +
  scale_color_manual(values = c("chartreuse4", "orangered2")) +
  geom_smooth(method='lm', alpha = 0.2, colour = "dodgerblue4", size = 0.8, weight = 0.3) +
  geom_smooth(aes(color = Gender), method='lm', se = F, alpha = 0.2, linetype = "longdash", size = 0.3, weight = 0.3) +
  stat_cor(method = "pearson", label.y = 9.7, label.x = 70, color = "dodgerblue4") +
  ylab("Relative Volume (in %)") + xlab("Age (in years)") +
  theme_minimal() +
  ggtitle("RELATIVE VOLUME central lobe") +
  theme(plot.title = element_text(hjust = 0.5))
Relative.Central.lobe.Age.plot
ggsave("TOR.Relative.Central.lobe.Age.plot.pdf", plot = Relative.Central.lobe.Age.plot, width = 8, height = 6, units = "in", dpi = 600)  
 
 
   
 
 
  
Relative.Parietal.lobe.Age.plot <-  ggplot(Topographic.overview.relative1, aes(y=Parietal.lobe, x = Age))  +
  geom_point(aes(color = Gender), size = 1.5, alpha = 1, shape = 16) +
  scale_color_manual(values = c("chartreuse4", "orangered2")) +
  geom_smooth(method='lm', alpha = 0.2, colour = "dodgerblue4", size = 0.8, weight = 0.3) +
  geom_smooth(aes(color = Gender), method='lm', se = F, alpha = 0.2, linetype = "longdash", size = 0.3, weight = 0.3) +
  stat_cor(method = "pearson", label.y = 13.2, label.x = 70, color = "dodgerblue4") +
  ylab("Relative Volume (in %)") + xlab("Age (in years)") +
  theme_minimal() +
  ggtitle("RELATIVE VOLUME parietal lobe") +
  theme(plot.title = element_text(hjust = 0.5))
Relative.Parietal.lobe.Age.plot
ggsave("TOR.Relative.Parietal.lobe.Age.plot.pdf", plot = Relative.Parietal.lobe.Age.plot, width = 8, height = 6, units = "in", dpi = 600)  
 
 
   
 
 
  
Relative.Occipital.lobe.Age.plot <-  ggplot(Topographic.overview.relative1, aes(y=Occipital.lobe, x = Age))  +
  geom_point(aes(color = Gender), size = 1.5, alpha = 1, shape = 16) +
  scale_color_manual(values = c("chartreuse4", "orangered2")) +
  geom_smooth(method='lm', alpha = 0.2, colour = "dodgerblue4", size = 0.8, weight = 0.3) +
  geom_smooth(aes(color = Gender), method='lm', se = F, alpha = 0.2, linetype = "longdash", size = 0.3, weight = 0.3) +
  stat_cor(method = "pearson", label.y = 7.6, label.x = 70, color = "dodgerblue4") +
  ylab("Relative Volume (in %)") + xlab("Age (in years)") +
  theme_minimal() +
  ggtitle("RELATIVE VOLUME occipital lobe") +
  theme(plot.title = element_text(hjust = 0.5))
Relative.Occipital.lobe.Age.plot
ggsave("TOR.Relative.Occipital.lobe.Age.plot.pdf", plot = Relative.Occipital.lobe.Age.plot, width = 8, height = 6, units = "in", dpi = 600)  
 
 
   
 
 
  
Relative.Temporal.lobe.Age.plot <-  ggplot(Topographic.overview.relative1, aes(y=Temporal.lobe, x = Age))  +
  geom_point(aes(color = Gender), size = 1.5, alpha = 1, shape = 16) +
  scale_color_manual(values = c("chartreuse4", "orangered2")) +
  geom_smooth(method='lm', alpha = 0.2, colour = "dodgerblue4", size = 0.8, weight = 0.3) +
  geom_smooth(aes(color = Gender), method='lm', se = F, alpha = 0.2, linetype = "longdash", size = 0.3, weight = 0.3) +
  stat_cor(method = "pearson", label.y = 8.6, label.x = 70, color = "dodgerblue4") +
  ylab("Relative Volume (in %)") + xlab("Age (in years)") +
  theme_minimal() +
  ggtitle("RELATIVE VOLUME temporal lobe") +
  theme(plot.title = element_text(hjust = 0.5))
Relative.Temporal.lobe.Age.plot
ggsave("TOR.Relative.Temporal.lobe.Age.plot.pdf", plot = Relative.Temporal.lobe.Age.plot, width = 8, height = 6, units = "in", dpi = 600)  
 
 
   
 
 
  
Relative.Insular.lobe.Age.plot <-  ggplot(Topographic.overview.relative1, aes(y=Insular.lobe, x = Age))  +
  geom_point(aes(color = Gender), size = 1.5, alpha = 1, shape = 16) +
  scale_color_manual(values = c("chartreuse4", "orangered2")) +
  geom_smooth(method='lm', alpha = 0.2, colour = "dodgerblue4", size = 0.8, weight = 0.3) +
  geom_smooth(aes(color = Gender), method='lm', se = F, alpha = 0.2, linetype = "longdash", size = 0.3, weight = 0.3) +
  stat_cor(method = "pearson", label.y = 2.05, label.x = 70, color = "dodgerblue4") +
  ylab("Relative Volume (in %)") + xlab("Age (in years)") +
  theme_minimal() +
  ggtitle("RELATIVE VOLUME insular lobe") +
  theme(plot.title = element_text(hjust = 0.5))
Relative.Insular.lobe.Age.plot
ggsave("TOR.Relative.Insular.lobe.Age.plot.pdf", plot = Relative.Insular.lobe.Age.plot, width = 8, height = 6, units = "in", dpi = 600)  
 
 
   
 
 
  
Relative.Limbic.lobe.Age.plot <-  ggplot(Topographic.overview.relative1, aes(y=Limbic.lobe, x = Age))  +
  geom_point(aes(color = Gender), size = 1.5, alpha = 1, shape = 16) +
  scale_color_manual(values = c("chartreuse4", "orangered2")) +
  geom_smooth(method='lm', alpha = 0.2, colour = "dodgerblue4", size = 0.8, weight = 0.3) +
  geom_smooth(aes(color = Gender), method='lm', se = F, alpha = 0.2, linetype = "longdash", size = 0.3, weight = 0.3) +
  stat_cor(method = "pearson", label.y = 7.7, label.x = 70, color = "dodgerblue4") +
  ylab("Relative Volume (in %)") + xlab("Age (in years)") +
  theme_minimal() +
  ggtitle("RELATIVE VOLUME limbic lobe") +
  theme(plot.title = element_text(hjust = 0.5))
Relative.Limbic.lobe.Age.plot
ggsave("TOR.Relative.Limbic.lobe.Age.plot.pdf", plot = Relative.Limbic.lobe.Age.plot, width = 8, height = 6, units = "in", dpi = 600)  
 
 
   
 
 
  
Relative.Basal.ganglia.Age.plot <-  ggplot(Topographic.overview.relative1, aes(y=Basal.ganglia, x = Age))  +
  geom_point(aes(color = Gender), size = 1.5, alpha = 1, shape = 16) +
  scale_color_manual(values = c("chartreuse4", "orangered2")) +
  geom_smooth(method='lm', alpha = 0.2, colour = "dodgerblue4", size = 0.8, weight = 0.3) +
  geom_smooth(aes(color = Gender), method='lm', se = F, alpha = 0.2, linetype = "longdash", size = 0.3, weight = 0.3) +
  stat_cor(method = "pearson", label.y = 2.25, label.x = 70, color = "dodgerblue4") +
  ylab("Relative Volume (in %)") + xlab("Age (in years)") +
  theme_minimal() +
  ggtitle("RELATIVE VOLUME basal ganglia") +
  theme(plot.title = element_text(hjust = 0.5))
Relative.Basal.ganglia.Age.plot
ggsave("TOR.Relative.Basal.ganglia.Age.plot.pdf", plot = Relative.Basal.ganglia.Age.plot, width = 8, height = 6, units = "in", dpi = 600)  
 
 
   
 
 
  
Relative.Diencephalon.Age.plot <-  ggplot(Topographic.overview.relative1, aes(y=Diencephalon, x = Age))  +
  geom_point(aes(color = Gender), size = 1.5, alpha = 1, shape = 16) +
  scale_color_manual(values = c("chartreuse4", "orangered2")) +
  geom_smooth(method='lm', alpha = 0.2, colour = "dodgerblue4", size = 0.8, weight = 0.3) +
  geom_smooth(aes(color = Gender), method='lm', se = F, alpha = 0.2, linetype = "longdash", size = 0.3, weight = 0.3) +
  stat_cor(method = "pearson", label.y = 2.25, label.x = 70, color = "dodgerblue4") +
  ylab("Relative Volume (in %)") + xlab("Age (in years)") +
  theme_minimal() +
  ggtitle("RELATIVE VOLUME diencephalon") +
  theme(plot.title = element_text(hjust = 0.5))
Relative.Diencephalon.Age.plot
ggsave("TOR.Relative.Diencephalon.Age.plot.pdf", plot = Relative.Diencephalon.Age.plot, width = 8, height = 6, units = "in", dpi = 600)  
 
 
   
 
 
  
Relative.Brainstem.Age.plot <-  ggplot(Topographic.overview.relative1, aes(y=Brainstem, x = Age))  +
  geom_point(aes(color = Gender), size = 1.5, alpha = 1, shape = 16) +
  scale_color_manual(values = c("chartreuse4", "orangered2")) +
  geom_smooth(method='lm', alpha = 0.2, colour = "dodgerblue4", size = 0.8, weight = 0.3) +
  geom_smooth(aes(color = Gender), method='lm', se = F, alpha = 0.2, linetype = "longdash", size = 0.3, weight = 0.3) +
  stat_cor(method = "pearson", label.y = 3.18, label.x = 70, color = "dodgerblue4") +
  ylab("Relative Volume (in %)") + xlab("Age (in years)") +
  theme_minimal() +
  ggtitle("RELATIVE VOLUME brainstem") +
  theme(plot.title = element_text(hjust = 0.5))
Relative.Brainstem.Age.plot
ggsave("TOR.Relative.Brainstem.Age.plot.pdf", plot = Relative.Brainstem.Age.plot, width = 8, height = 6, units = "in", dpi = 600)  
 
 
   
 
 
  
Relative.Cerebellum.Age.plot <-  ggplot(Topographic.overview.relative1, aes(y=Cerebellum, x = Age))  +
  geom_point(aes(color = Gender), size = 1.5, alpha = 1, shape = 16) +
  scale_color_manual(values = c("chartreuse4", "orangered2")) +
  geom_smooth(method='lm', alpha = 0.2, colour = "dodgerblue4", size = 0.8, weight = 0.3) +
  geom_smooth(aes(color = Gender), method='lm', se = F, alpha = 0.2, linetype = "longdash", size = 0.3, weight = 0.3) +
  stat_cor(method = "pearson", label.y = 12.7, label.x = 70, color = "dodgerblue4") +
  ylab("Relative Volume (in %)") + xlab("Age (in years)") +
  theme_minimal() +
  ggtitle("RELATIVE VOLUME cerebellum") +
  theme(plot.title = element_text(hjust = 0.5))
Relative.Cerebellum.Age.plot
ggsave("TOR.Relative.Cerebellum.Age.plot.pdf", plot = Relative.Cerebellum.Age.plot, width = 8, height = 6, units = "in", dpi = 600)
  
 
 
   
 
 
 
 
 
 
 The Prosencephalon 
 
 Absolute Volumes 
 
 
 
  
Frontal.pole <- All.Volumes$`Total volume frontal pole`
F1 <- All.Volumes$`Total volume F1`
F2 <- All.Volumes$`Total volume F2`
F3.orbital <- All.Volumes$`Total volume F3 orbital`
F3.triangular <- All.Volumes$`Total volume F3 triangular`
F3.opercular <- All.Volumes$`Total volume F3 opercular`
Anterior.orbital <- All.Volumes$`Total volume anterior orbital`
Medial.orbital <- All.Volumes$`Total volume medial orbital`
Lateral.orbital <- All.Volumes$`Total volume lateral orbital`
Posterior.orbital <- All.Volumes$`Total volume posterior orbital`
Rectus <- All.Volumes$`Total volume rectus`
Rostral <- All.Volumes$`Total volume rostral`

Precentral <- All.Volumes$`Total volume PreC`
Postcentral <- All.Volumes$`Total volume PostC`
Paracentral.lobule <- All.Volumes$`Total volume ParaC lobule`
Subcentral <- All.Volumes$`Total volume SubC gyrus`

SPL <- All.Volumes$`Total volume SPL`
SMG <- All.Volumes$`Total volume SMG`
ANG <- All.Volumes$`Total volume ANG`
Precuneus <- All.Volumes$`Total volume Precuneus`

Cuneus <- All.Volumes$`Total volume Cuneus`
O1 <- All.Volumes$`Total volume O1`
O2 <- All.Volumes$`Total volume O2`
O3 <- All.Volumes$`Total volume O3`
Occipital.pole <- All.Volumes$`Total volume occipital pole`
Lingual <- All.Volumes$`Total volume lingual`

Fusiform <- All.Volumes$`Total volume fusiform`
Temporal.pole <- All.Volumes$`Total volume T1`
T1 <- All.Volumes$`Total volume T2`
T2 <- All.Volumes$`Total volume T3`
T3 <- All.Volumes$`Total volume Planum temporale`
Planum.temporale <- All.Volumes$`Total volume Planum polare`
Planum.polare <- All.Volumes$`Total volume temporal pole`

Short.insular <- All.Volumes$`Total volume short insular gyri`
Long.insular <- All.Volumes$`Total volume long insular gyri`

SCA <- All.Volumes$`Total volume SCA`
Cingulate.anterior <- All.Volumes$`Total volume ant cingulate`
Cingulate.middle <- All.Volumes$`Total volume mid cingulate`
Cingulate.posterior <- All.Volumes$`Total volume post cingulate`
PHG <- All.Volumes$`Total volume PHG`
Hippocampus <- All.Volumes$`Total volume hippocampus`
Amygdala <- All.Volumes$`Total volume amygdala`

Corpus.callosum <- All.Volumes$`Total volume corpus callosum`

Claustrum <- All.Volumes$`Total volume claustrum`
Putamen <- All.Volumes$`Total volume putamen`
Caudate <- All.Volumes$`Total volume caudate`
Globus.pallidum <- All.Volumes$`Total volume pallidum`
Internal.capsule <- All.Volumes$`Total volume internal capsule`
Innominate.substance <- All.Volumes$`Total volume substantia innominata`
Hypothalamus <- All.Volumes$`Total volume hypothalamus`
Thalamus <- All.Volumes$`Total volume thalamus`

Prosencephalon.absolute <- as.data.frame(cbind(
  Frontal.pole,
  F1,
  F2,
  F3.orbital, 
  F3.triangular, 
  F3.opercular, 
  Anterior.orbital, 
  Medial.orbital, 
  Lateral.orbital, 
  Posterior.orbital,
  Rectus, 
  Rostral, 
  Precentral, 
  Postcentral, 
  Paracentral.lobule, 
  Subcentral, 
  SPL, 
  SMG, 
  ANG, 
  Precuneus,
  Cuneus, 
  O1,
  O2, 
  O3, 
  Occipital.pole, 
  Lingual, 
  Fusiform, 
  Temporal.pole, 
  T1,
  T2, 
  T3, 
  Planum.temporale, 
  Planum.polare, 
  Short.insular, 
  Long.insular, 
  SCA, 
  Cingulate.anterior, 
  Cingulate.middle, 
  Cingulate.posterior, 
  PHG, 
  Hippocampus, 
  Amygdala, 
  Corpus.callosum, 
  Claustrum, 
  Putamen, 
  Caudate, 
  Globus.pallidum, 
  Internal.capsule,
  Innominate.substance, 
  Hypothalamus,
  Thalamus
))

Prosencephalon.absolute$Gender <- All.Volumes$Gender

Table.Prosencephalon.absolute <- CreateTableOne(
  vars = c("Frontal.pole",
  "F1",
  "F2",
  "F3.orbital", 
  "F3.triangular", 
  "F3.opercular", 
  "Anterior.orbital", 
  "Medial.orbital", 
  "Lateral.orbital", 
  "Posterior.orbital",
  "Rectus", 
  "Rostral", 
  "Precentral", 
  "Postcentral", 
  "Paracentral.lobule", 
  "Subcentral", 
  "SPL", 
  "SMG", 
  "ANG", 
  "Precuneus",
  "Cuneus", 
  "O1",
  "O2", 
  "O3", 
  "Occipital.pole", 
  "Lingual", 
  "Fusiform", 
  "Temporal.pole", 
  "T1",
  "T2", 
  "T3", 
  "Planum.temporale", 
  "Planum.polare", 
  "Short.insular", 
  "Long.insular", 
  "SCA", 
  "Cingulate.anterior", 
  "Cingulate.middle", 
  "Cingulate.posterior", 
  "PHG", 
  "Hippocampus", 
  "Amygdala", 
  "Corpus.callosum", 
  "Claustrum", 
  "Putamen", 
  "Caudate", 
  "Globus.pallidum", 
  "Internal.capsule",
  "Innominate.substance", 
  "Hypothalamus",
  "Thalamus"),
  data = Prosencephalon.absolute)

Table.Prosencephalon.absolute.stratified.gender <- CreateTableOne(
  vars = c("Frontal.pole",
  "F1",
  "F2",
  "F3.orbital", 
  "F3.triangular", 
  "F3.opercular", 
  "Anterior.orbital", 
  "Medial.orbital", 
  "Lateral.orbital", 
  "Posterior.orbital",
  "Rectus", 
  "Rostral", 
  "Precentral", 
  "Postcentral", 
  "Paracentral.lobule", 
  "Subcentral", 
  "SPL", 
  "SMG", 
  "ANG", 
  "Precuneus",
  "Cuneus", 
  "O1",
  "O2", 
  "O3", 
  "Occipital.pole", 
  "Lingual", 
  "Fusiform", 
  "Temporal.pole", 
  "T1",
  "T2", 
  "T3", 
  "Planum.temporale", 
  "Planum.polare", 
  "Short.insular", 
  "Long.insular", 
  "SCA", 
  "Cingulate.anterior", 
  "Cingulate.middle", 
  "Cingulate.posterior", 
  "PHG", 
  "Hippocampus", 
  "Amygdala", 
  "Corpus.callosum", 
  "Claustrum", 
  "Putamen", 
  "Caudate", 
  "Globus.pallidum", 
  "Internal.capsule",
  "Innominate.substance", 
  "Hypothalamus",
  "Thalamus"),
  strata = c("Gender"),
  data = Prosencephalon.absolute)

Table.Prosencephalon.absolute <- print(Table.Prosencephalon.absolute, contDigits = 2)  
 
 
                                    
                                   Overall           
  n                                      30          
  Frontal.pole (mean (SD))          4710.60 (976.19) 
  F1 (mean (SD))                   68776.43 (9196.02)
  F2 (mean (SD))                   58458.30 (7689.55)
  F3.orbital (mean (SD))            3916.57 (613.82) 
  F3.triangular (mean (SD))        11688.77 (1898.88)
  F3.opercular (mean (SD))         13949.13 (2022.23)
  Anterior.orbital (mean (SD))      3349.47 (604.18) 
  Medial.orbital (mean (SD))        7566.23 (1369.20)
  Lateral.orbital (mean (SD))       6063.57 (1102.27)
  Posterior.orbital (mean (SD))     7420.67 (1341.23)
  Rectus (mean (SD))                9986.63 (4584.25)
  Rostral (mean (SD))               3593.23 (379.75) 
  Precentral (mean (SD))           39114.37 (4348.35)
  Postcentral (mean (SD))          22307.97 (3280.88)
  Paracentral.lobule (mean (SD))   14036.93 (1769.85)
  Subcentral (mean (SD))            6057.87 (1027.51)
  SPL (mean (SD))                  33315.33 (4452.27)
  SMG (mean (SD))                  30707.70 (4785.83)
  ANG (mean (SD))                  34333.43 (4760.76)
  Precuneus (mean (SD))            30859.33 (4302.66)
  Cuneus (mean (SD))               10063.17 (1461.83)
  O1 (mean (SD))                    9149.23 (1312.27)
  O2 (mean (SD))                   15786.67 (2399.63)
  O3 (mean (SD))                    9263.87 (1668.79)
  Occipital.pole (mean (SD))       11367.23 (1511.90)
  Lingual (mean (SD))              20280.43 (2733.84)
  Fusiform (mean (SD))             22196.20 (3298.55)
  Temporal.pole (mean (SD))        25353.77 (3578.13)
  T1 (mean (SD))                   27247.23 (3925.33)
  T2 (mean (SD))                   23693.10 (3139.43)
  T3 (mean (SD))                    5010.53 (871.47) 
  Planum.temporale (mean (SD))      3078.07 (770.86) 
  Planum.polare (mean (SD))         9897.97 (1847.85)
  Short.insular (mean (SD))        15405.47 (2108.68)
  Long.insular (mean (SD))          9141.97 (1881.34)
  SCA (mean (SD))                   2394.33 (662.65) 
  Cingulate.anterior (mean (SD))   14419.77 (2135.23)
  Cingulate.middle (mean (SD))     17135.30 (2613.59)
  Cingulate.posterior (mean (SD))  20174.30 (2957.84)
  PHG (mean (SD))                  10443.13 (1652.70)
  Hippocampus (mean (SD))           8127.77 (952.70) 
  Amygdala (mean (SD))              3121.60 (469.63) 
  Corpus.callosum (mean (SD))       3311.90 (552.40) 
  Claustrum (mean (SD))             1344.90 (417.64) 
  Putamen (mean (SD))              11264.80 (1661.91)
  Caudate (mean (SD))               7779.73 (1318.33)
  Globus.pallidum (mean (SD))       3072.57 (592.30) 
  Internal.capsule (mean (SD))     10616.47 (1548.67)
  Innominate.substance (mean (SD))  2687.93 (322.72) 
  Hypothalamus (mean (SD))          7962.50 (917.36) 
  Thalamus (mean (SD))             14606.33 (1462.98)  
 
 
  Table.Prosencephalon.absolute.stratified.gender <- print(Table.Prosencephalon.absolute.stratified.gender, contDigits = 2)  
 
 
                                    Stratified by Gender
                                   f                  m                  p      test
  n                                      14                 16                      
  Frontal.pole (mean (SD))          4369.21 (705.73)   5009.31 (1098.53)  0.072     
  F1 (mean (SD))                   64718.00 (8455.20) 72327.56 (8527.23)  0.021     
  F2 (mean (SD))                   55366.50 (7297.68) 61163.62 (7171.64)  0.037     
  F3.orbital (mean (SD))            3665.71 (565.37)   4136.06 (584.21)   0.034     
  F3.triangular (mean (SD))        10845.50 (1038.66) 12426.62 (2189.00)  0.020     
  F3.opercular (mean (SD))         12752.21 (1540.15) 14996.44 (1828.47)  0.001     
  Anterior.orbital (mean (SD))      3087.00 (244.87)   3579.12 (730.22)   0.023     
  Medial.orbital (mean (SD))        6973.14 (558.72)   8085.19 (1654.78)  0.024     
  Lateral.orbital (mean (SD))       5590.57 (456.44)   6477.44 (1333.01)  0.025     
  Posterior.orbital (mean (SD))     6845.21 (546.20)   7924.19 (1624.75)  0.025     
  Rectus (mean (SD))                8721.00 (747.46)  11094.06 (6110.83)  0.161     
  Rostral (mean (SD))               3413.07 (295.50)   3750.88 (382.52)   0.012     
  Precentral (mean (SD))           36944.14 (3427.70) 41013.31 (4258.04)  0.008     
  Postcentral (mean (SD))          20938.00 (3435.44) 23506.69 (2701.43)  0.030     
  Paracentral.lobule (mean (SD))   13361.57 (1785.89) 14627.88 (1579.10)  0.049     
  Subcentral (mean (SD))            5502.36 (885.64)   6543.94 (906.29)   0.004     
  SPL (mean (SD))                  31813.43 (4849.58) 34629.50 (3740.81)  0.084     
  SMG (mean (SD))                  28640.79 (4365.75) 32516.25 (4504.07)  0.024     
  ANG (mean (SD))                  31864.79 (4552.43) 36493.50 (3897.76)  0.006     
  Precuneus (mean (SD))            28510.00 (3788.70) 32915.00 (3700.31)  0.003     
  Cuneus (mean (SD))                9355.07 (1040.46) 10682.75 (1521.76)  0.010     
  O1 (mean (SD))                    8298.50 (925.51)   9893.62 (1149.08) <0.001     
  O2 (mean (SD))                   14683.57 (1913.46) 16751.88 (2414.54)  0.016     
  O3 (mean (SD))                    8261.00 (1177.06) 10141.38 (1556.68)  0.001     
  Occipital.pole (mean (SD))       10666.64 (1017.58) 11980.25 (1631.86)  0.015     
  Lingual (mean (SD))              19665.43 (2166.02) 20818.56 (3117.94)  0.256     
  Fusiform (mean (SD))             20947.07 (2857.52) 23289.19 (3350.86)  0.050     
  Temporal.pole (mean (SD))        23479.07 (2923.96) 26994.12 (3345.51)  0.005     
  T1 (mean (SD))                   24988.57 (2642.33) 29223.56 (3848.44)  0.002     
  T2 (mean (SD))                   22875.64 (2875.80) 24408.38 (3273.84)  0.187     
  T3 (mean (SD))                    4531.00 (734.14)   5430.12 (773.80)   0.003     
  Planum.temporale (mean (SD))      2714.57 (409.79)   3396.12 (878.68)   0.013     
  Planum.polare (mean (SD))         9805.79 (1645.78)  9978.62 (2058.92)  0.803     
  Short.insular (mean (SD))        14652.79 (1336.31) 16064.06 (2461.22)  0.066     
  Long.insular (mean (SD))          8093.57 (1221.68) 10059.31 (1904.19)  0.003     
  SCA (mean (SD))                   2108.21 (542.11)   2644.69 (671.54)   0.024     
  Cingulate.anterior (mean (SD))   13500.14 (2193.65) 15224.44 (1778.78)  0.025     
  Cingulate.middle (mean (SD))     16044.50 (2245.99) 18089.75 (2598.50)  0.030     
  Cingulate.posterior (mean (SD))  18885.71 (2679.94) 21301.81 (2790.00)  0.023     
  PHG (mean (SD))                  10013.00 (1197.43) 10819.50 (1927.25)  0.187     
  Hippocampus (mean (SD))           7823.93 (712.09)   8393.62 (1074.12)  0.103     
  Amygdala (mean (SD))              3009.43 (469.08)   3219.75 (462.25)   0.227     
  Corpus.callosum (mean (SD))       3319.00 (580.49)   3305.69 (545.72)   0.949     
  Claustrum (mean (SD))             1189.93 (305.72)   1480.50 (462.80)   0.056     
  Putamen (mean (SD))              10793.64 (1243.13) 11677.06 (1900.52)  0.149     
  Caudate (mean (SD))               7368.07 (931.75)   8139.94 (1520.25)  0.111     
  Globus.pallidum (mean (SD))       2861.14 (330.56)   3257.56 (710.86)   0.066     
  Internal.capsule (mean (SD))     10153.14 (1654.93) 11021.88 (1373.90)  0.127     
  Innominate.substance (mean (SD))  2639.07 (362.97)   2730.69 (288.08)   0.448     
  Hypothalamus (mean (SD))          7507.07 (671.29)   8361.00 (934.61)   0.008     
  Thalamus (mean (SD))             14190.21 (1205.08) 14970.44 (1605.09)  0.148       
 
 
  write.csv(Table.Prosencephalon.absolute, "Table.Prosencephalon.absolute.csv")
write.csv(Table.Prosencephalon.absolute.stratified.gender, "Table.Prosencephalon.absolute.stratified.gender.csv")
  
 
 
 
 
 
 
  
Table.Prosencephalon.absolute.RSD <- as.data.frame(Table.Prosencephalon.absolute)
Table.Prosencephalon.absolute.RSD <- data.frame(do.call('rbind', 
                                                              strsplit(as.character(Table.Prosencephalon.absolute.RSD[-1,]),
                                                                       ' (',fixed=TRUE)))
Table.Prosencephalon.absolute.RSD <- data.frame(cbind(str_replace_all(Table.Prosencephalon.absolute.RSD$X1, "[ ]", ""),
                                                            str_replace_all(Table.Prosencephalon.absolute.RSD$X2, "[)]", "")))
Table.Prosencephalon.absolute.RSD$X1 <- as.character(Table.Prosencephalon.absolute.RSD$X1)
Table.Prosencephalon.absolute.RSD$X2 <- as.character(Table.Prosencephalon.absolute.RSD$X2)
Table.Prosencephalon.absolute.RSD <- as.data.frame(sapply(Table.Prosencephalon.absolute.RSD, as.numeric))
Table.Prosencephalon.absolute.RSD <- as.data.frame(Table.Prosencephalon.absolute.RSD$X2/Table.Prosencephalon.absolute.RSD$X1)
Table.Prosencephalon.absolute.RSD <- round(Table.Prosencephalon.absolute.RSD * 100, 1)


Table.Prosencephalon.absolute.stratified.gender.RSD <- as.data.frame(Table.Prosencephalon.absolute.stratified.gender)
Table.Prosencephalon.absolute.stratified.gender.RSD <- select(Table.Prosencephalon.absolute.stratified.gender.RSD, - c(p, test))

Table.Prosencephalon.absolute.stratified.gender.RSD.female <- data.frame(do.call('rbind', 
                                                              strsplit(as.character(Table.Prosencephalon.absolute.stratified.gender.RSD[-1, "f"]),
                                                                       ' (',fixed=TRUE)))
Table.Prosencephalon.absolute.stratified.gender.RSD.female <- data.frame(cbind(str_replace_all(Table.Prosencephalon.absolute.stratified.gender.RSD.female$X1, "[ ]", ""),
                                                            str_replace_all(Table.Prosencephalon.absolute.stratified.gender.RSD.female$X2, "[)]", "")))
Table.Prosencephalon.absolute.stratified.gender.RSD.female$X1 <- as.character(Table.Prosencephalon.absolute.stratified.gender.RSD.female$X1)
Table.Prosencephalon.absolute.stratified.gender.RSD.female$X2 <- as.character(Table.Prosencephalon.absolute.stratified.gender.RSD.female$X2)
Table.Prosencephalon.absolute.stratified.gender.RSD.female <- as.data.frame(sapply(Table.Prosencephalon.absolute.stratified.gender.RSD.female, as.numeric))
Table.Prosencephalon.absolute.stratified.gender.RSD.female <- as.data.frame(Table.Prosencephalon.absolute.stratified.gender.RSD.female$X2/Table.Prosencephalon.absolute.stratified.gender.RSD.female$X1)
Table.Prosencephalon.absolute.stratified.gender.RSD.female <- round(Table.Prosencephalon.absolute.stratified.gender.RSD.female * 100, 1)

Table.Prosencephalon.absolute.stratified.gender.RSD.male <- data.frame(do.call('rbind', 
                                                              strsplit(as.character(Table.Prosencephalon.absolute.stratified.gender.RSD[-1, "m"]),
                                                                       ' (',fixed=TRUE)))
Table.Prosencephalon.absolute.stratified.gender.RSD.male <- data.frame(cbind(str_replace_all(Table.Prosencephalon.absolute.stratified.gender.RSD.male$X1, "[ ]", ""),
                                                            str_replace_all(Table.Prosencephalon.absolute.stratified.gender.RSD.male$X2, "[)]", "")))
Table.Prosencephalon.absolute.stratified.gender.RSD.male$X1 <- as.character(Table.Prosencephalon.absolute.stratified.gender.RSD.male$X1)
Table.Prosencephalon.absolute.stratified.gender.RSD.male$X2 <- as.character(Table.Prosencephalon.absolute.stratified.gender.RSD.male$X2)
Table.Prosencephalon.absolute.stratified.gender.RSD.male <- as.data.frame(sapply(Table.Prosencephalon.absolute.stratified.gender.RSD.male, as.numeric))
Table.Prosencephalon.absolute.stratified.gender.RSD.male <- as.data.frame(Table.Prosencephalon.absolute.stratified.gender.RSD.male$X2/Table.Prosencephalon.absolute.stratified.gender.RSD.male$X1)
Table.Prosencephalon.absolute.stratified.gender.RSD.male <- round(Table.Prosencephalon.absolute.stratified.gender.RSD.male * 100, 1)
  
 
 
 
 
 
 
  
kable(Table.Prosencephalon.absolute)  
 
 
 

 
 
 
  
 Overall 
 
 
 
 
 n 
 30 
 
 
 Frontal.pole (mean (SD)) 
 4710.60 (976.19) 
 
 
 F1 (mean (SD)) 
 68776.43 (9196.02) 
 
 
 F2 (mean (SD)) 
 58458.30 (7689.55) 
 
 
 F3.orbital (mean (SD)) 
 3916.57 (613.82) 
 
 
 F3.triangular (mean (SD)) 
 11688.77 (1898.88) 
 
 
 F3.opercular (mean (SD)) 
 13949.13 (2022.23) 
 
 
 Anterior.orbital (mean (SD)) 
 3349.47 (604.18) 
 
 
 Medial.orbital (mean (SD)) 
 7566.23 (1369.20) 
 
 
 Lateral.orbital (mean (SD)) 
 6063.57 (1102.27) 
 
 
 Posterior.orbital (mean (SD)) 
 7420.67 (1341.23) 
 
 
 Rectus (mean (SD)) 
 9986.63 (4584.25) 
 
 
 Rostral (mean (SD)) 
 3593.23 (379.75) 
 
 
 Precentral (mean (SD)) 
 39114.37 (4348.35) 
 
 
 Postcentral (mean (SD)) 
 22307.97 (3280.88) 
 
 
 Paracentral.lobule (mean (SD)) 
 14036.93 (1769.85) 
 
 
 Subcentral (mean (SD)) 
 6057.87 (1027.51) 
 
 
 SPL (mean (SD)) 
 33315.33 (4452.27) 
 
 
 SMG (mean (SD)) 
 30707.70 (4785.83) 
 
 
 ANG (mean (SD)) 
 34333.43 (4760.76) 
 
 
 Precuneus (mean (SD)) 
 30859.33 (4302.66) 
 
 
 Cuneus (mean (SD)) 
 10063.17 (1461.83) 
 
 
 O1 (mean (SD)) 
 9149.23 (1312.27) 
 
 
 O2 (mean (SD)) 
 15786.67 (2399.63) 
 
 
 O3 (mean (SD)) 
 9263.87 (1668.79) 
 
 
 Occipital.pole (mean (SD)) 
 11367.23 (1511.90) 
 
 
 Lingual (mean (SD)) 
 20280.43 (2733.84) 
 
 
 Fusiform (mean (SD)) 
 22196.20 (3298.55) 
 
 
 Temporal.pole (mean (SD)) 
 25353.77 (3578.13) 
 
 
 T1 (mean (SD)) 
 27247.23 (3925.33) 
 
 
 T2 (mean (SD)) 
 23693.10 (3139.43) 
 
 
 T3 (mean (SD)) 
 5010.53 (871.47) 
 
 
 Planum.temporale (mean (SD)) 
 3078.07 (770.86) 
 
 
 Planum.polare (mean (SD)) 
 9897.97 (1847.85) 
 
 
 Short.insular (mean (SD)) 
 15405.47 (2108.68) 
 
 
 Long.insular (mean (SD)) 
 9141.97 (1881.34) 
 
 
 SCA (mean (SD)) 
 2394.33 (662.65) 
 
 
 Cingulate.anterior (mean (SD)) 
 14419.77 (2135.23) 
 
 
 Cingulate.middle (mean (SD)) 
 17135.30 (2613.59) 
 
 
 Cingulate.posterior (mean (SD)) 
 20174.30 (2957.84) 
 
 
 PHG (mean (SD)) 
 10443.13 (1652.70) 
 
 
 Hippocampus (mean (SD)) 
 8127.77 (952.70) 
 
 
 Amygdala (mean (SD)) 
 3121.60 (469.63) 
 
 
 Corpus.callosum (mean (SD)) 
 3311.90 (552.40) 
 
 
 Claustrum (mean (SD)) 
 1344.90 (417.64) 
 
 
 Putamen (mean (SD)) 
 11264.80 (1661.91) 
 
 
 Caudate (mean (SD)) 
 7779.73 (1318.33) 
 
 
 Globus.pallidum (mean (SD)) 
 3072.57 (592.30) 
 
 
 Internal.capsule (mean (SD)) 
 10616.47 (1548.67) 
 
 
 Innominate.substance (mean (SD)) 
 2687.93 (322.72) 
 
 
 Hypothalamus (mean (SD)) 
 7962.50 (917.36) 
 
 
 Thalamus (mean (SD)) 
 14606.33 (1462.98) 
 
 
 


 
   
  kable(Table.Prosencephalon.absolute.RSD)  
 
 
 

 
 
 
 Table.Prosencephalon.absolute.RSD  X 2/ T  a  b  l  e . P  r  o  s  e  n  c  e  p  h  a  l  o  n . a  b  s  o  l  u  t  e . R  S  D  X1 
 
 
 
 
 20.7 
 
 
 13.4 
 
 
 13.2 
 
 
 15.7 
 
 
 16.2 
 
 
 14.5 
 
 
 18.0 
 
 
 18.1 
 
 
 18.2 
 
 
 18.1 
 
 
 45.9 
 
 
 10.6 
 
 
 11.1 
 
 
 14.7 
 
 
 12.6 
 
 
 17.0 
 
 
 13.4 
 
 
 15.6 
 
 
 13.9 
 
 
 13.9 
 
 
 14.5 
 
 
 14.3 
 
 
 15.2 
 
 
 18.0 
 
 
 13.3 
 
 
 13.5 
 
 
 14.9 
 
 
 14.1 
 
 
 14.4 
 
 
 13.3 
 
 
 17.4 
 
 
 25.0 
 
 
 18.7 
 
 
 13.7 
 
 
 20.6 
 
 
 27.7 
 
 
 14.8 
 
 
 15.3 
 
 
 14.7 
 
 
 15.8 
 
 
 11.7 
 
 
 15.0 
 
 
 16.7 
 
 
 31.1 
 
 
 14.8 
 
 
 16.9 
 
 
 19.3 
 
 
 14.6 
 
 
 12.0 
 
 
 11.5 
 
 
 10.0 
 
 
 


 
   
  kable(Table.Prosencephalon.absolute.stratified.gender)  
 
 
 

 
 
 
  
 f 
 m 
 p 
 test 
 
 
 
 
 n 
 14 
 16 
  
  
 
 
 Frontal.pole (mean (SD)) 
 4369.21 (705.73) 
 5009.31 (1098.53) 
 0.072 
  
 
 
 F1 (mean (SD)) 
 64718.00 (8455.20) 
 72327.56 (8527.23) 
 0.021 
  
 
 
 F2 (mean (SD)) 
 55366.50 (7297.68) 
 61163.62 (7171.64) 
 0.037 
  
 
 
 F3.orbital (mean (SD)) 
 3665.71 (565.37) 
 4136.06 (584.21) 
 0.034 
  
 
 
 F3.triangular (mean (SD)) 
 10845.50 (1038.66) 
 12426.62 (2189.00) 
 0.020 
  
 
 
 F3.opercular (mean (SD)) 
 12752.21 (1540.15) 
 14996.44 (1828.47) 
 0.001 
  
 
 
 Anterior.orbital (mean (SD)) 
 3087.00 (244.87) 
 3579.12 (730.22) 
 0.023 
  
 
 
 Medial.orbital (mean (SD)) 
 6973.14 (558.72) 
 8085.19 (1654.78) 
 0.024 
  
 
 
 Lateral.orbital (mean (SD)) 
 5590.57 (456.44) 
 6477.44 (1333.01) 
 0.025 
  
 
 
 Posterior.orbital (mean (SD)) 
 6845.21 (546.20) 
 7924.19 (1624.75) 
 0.025 
  
 
 
 Rectus (mean (SD)) 
 8721.00 (747.46) 
 11094.06 (6110.83) 
 0.161 
  
 
 
 Rostral (mean (SD)) 
 3413.07 (295.50) 
 3750.88 (382.52) 
 0.012 
  
 
 
 Precentral (mean (SD)) 
 36944.14 (3427.70) 
 41013.31 (4258.04) 
 0.008 
  
 
 
 Postcentral (mean (SD)) 
 20938.00 (3435.44) 
 23506.69 (2701.43) 
 0.030 
  
 
 
 Paracentral.lobule (mean (SD)) 
 13361.57 (1785.89) 
 14627.88 (1579.10) 
 0.049 
  
 
 
 Subcentral (mean (SD)) 
 5502.36 (885.64) 
 6543.94 (906.29) 
 0.004 
  
 
 
 SPL (mean (SD)) 
 31813.43 (4849.58) 
 34629.50 (3740.81) 
 0.084 
  
 
 
 SMG (mean (SD)) 
 28640.79 (4365.75) 
 32516.25 (4504.07) 
 0.024 
  
 
 
 ANG (mean (SD)) 
 31864.79 (4552.43) 
 36493.50 (3897.76) 
 0.006 
  
 
 
 Precuneus (mean (SD)) 
 28510.00 (3788.70) 
 32915.00 (3700.31) 
 0.003 
  
 
 
 Cuneus (mean (SD)) 
 9355.07 (1040.46) 
 10682.75 (1521.76) 
 0.010 
  
 
 
 O1 (mean (SD)) 
 8298.50 (925.51) 
 9893.62 (1149.08) 
 <0.001 
  
 
 
 O2 (mean (SD)) 
 14683.57 (1913.46) 
 16751.88 (2414.54) 
 0.016 
  
 
 
 O3 (mean (SD)) 
 8261.00 (1177.06) 
 10141.38 (1556.68) 
 0.001 
  
 
 
 Occipital.pole (mean (SD)) 
 10666.64 (1017.58) 
 11980.25 (1631.86) 
 0.015 
  
 
 
 Lingual (mean (SD)) 
 19665.43 (2166.02) 
 20818.56 (3117.94) 
 0.256 
  
 
 
 Fusiform (mean (SD)) 
 20947.07 (2857.52) 
 23289.19 (3350.86) 
 0.050 
  
 
 
 Temporal.pole (mean (SD)) 
 23479.07 (2923.96) 
 26994.12 (3345.51) 
 0.005 
  
 
 
 T1 (mean (SD)) 
 24988.57 (2642.33) 
 29223.56 (3848.44) 
 0.002 
  
 
 
 T2 (mean (SD)) 
 22875.64 (2875.80) 
 24408.38 (3273.84) 
 0.187 
  
 
 
 T3 (mean (SD)) 
 4531.00 (734.14) 
 5430.12 (773.80) 
 0.003 
  
 
 
 Planum.temporale (mean (SD)) 
 2714.57 (409.79) 
 3396.12 (878.68) 
 0.013 
  
 
 
 Planum.polare (mean (SD)) 
 9805.79 (1645.78) 
 9978.62 (2058.92) 
 0.803 
  
 
 
 Short.insular (mean (SD)) 
 14652.79 (1336.31) 
 16064.06 (2461.22) 
 0.066 
  
 
 
 Long.insular (mean (SD)) 
 8093.57 (1221.68) 
 10059.31 (1904.19) 
 0.003 
  
 
 
 SCA (mean (SD)) 
 2108.21 (542.11) 
 2644.69 (671.54) 
 0.024 
  
 
 
 Cingulate.anterior (mean (SD)) 
 13500.14 (2193.65) 
 15224.44 (1778.78) 
 0.025 
  
 
 
 Cingulate.middle (mean (SD)) 
 16044.50 (2245.99) 
 18089.75 (2598.50) 
 0.030 
  
 
 
 Cingulate.posterior (mean (SD)) 
 18885.71 (2679.94) 
 21301.81 (2790.00) 
 0.023 
  
 
 
 PHG (mean (SD)) 
 10013.00 (1197.43) 
 10819.50 (1927.25) 
 0.187 
  
 
 
 Hippocampus (mean (SD)) 
 7823.93 (712.09) 
 8393.62 (1074.12) 
 0.103 
  
 
 
 Amygdala (mean (SD)) 
 3009.43 (469.08) 
 3219.75 (462.25) 
 0.227 
  
 
 
 Corpus.callosum (mean (SD)) 
 3319.00 (580.49) 
 3305.69 (545.72) 
 0.949 
  
 
 
 Claustrum (mean (SD)) 
 1189.93 (305.72) 
 1480.50 (462.80) 
 0.056 
  
 
 
 Putamen (mean (SD)) 
 10793.64 (1243.13) 
 11677.06 (1900.52) 
 0.149 
  
 
 
 Caudate (mean (SD)) 
 7368.07 (931.75) 
 8139.94 (1520.25) 
 0.111 
  
 
 
 Globus.pallidum (mean (SD)) 
 2861.14 (330.56) 
 3257.56 (710.86) 
 0.066 
  
 
 
 Internal.capsule (mean (SD)) 
 10153.14 (1654.93) 
 11021.88 (1373.90) 
 0.127 
  
 
 
 Innominate.substance (mean (SD)) 
 2639.07 (362.97) 
 2730.69 (288.08) 
 0.448 
  
 
 
 Hypothalamus (mean (SD)) 
 7507.07 (671.29) 
 8361.00 (934.61) 
 0.008 
  
 
 
 Thalamus (mean (SD)) 
 14190.21 (1205.08) 
 14970.44 (1605.09) 
 0.148 
  
 
 
 


 
   
  kable(Table.Prosencephalon.absolute.stratified.gender.RSD.female)  
 
 
 

 
 
 
 
 
 
 Table.Prosencephalon.absolute.stratified.gender.RSD.female  X 2/ T  a  b  l  e . P  r  o  s  e  n  c  e  p  h  a  l  o  n . a  b  s  o  l  u  t  e . s  t  r  a  t  i  f  i  e  d . g  e  n  d  e  r . R  S  D . f  e  m  a  l  e  X1 
 
 
 
 
 16.2 
 
 
 13.1 
 
 
 13.2 
 
 
 15.4 
 
 
 9.6 
 
 
 12.1 
 
 
 7.9 
 
 
 8.0 
 
 
 8.2 
 
 
 8.0 
 
 
 8.6 
 
 
 8.7 
 
 
 9.3 
 
 
 16.4 
 
 
 13.4 
 
 
 16.1 
 
 
 15.2 
 
 
 15.2 
 
 
 14.3 
 
 
 13.3 
 
 
 11.1 
 
 
 11.2 
 
 
 13.0 
 
 
 14.2 
 
 
 9.5 
 
 
 11.0 
 
 
 13.6 
 
 
 12.5 
 
 
 10.6 
 
 
 12.6 
 
 
 16.2 
 
 
 15.1 
 
 
 16.8 
 
 
 9.1 
 
 
 15.1 
 
 
 25.7 
 
 
 16.2 
 
 
 14.0 
 
 
 14.2 
 
 
 12.0 
 
 
 9.1 
 
 
 15.6 
 
 
 17.5 
 
 
 25.7 
 
 
 11.5 
 
 
 12.6 
 
 
 11.6 
 
 
 16.3 
 
 
 13.8 
 
 
 8.9 
 
 
 8.5 
 
 
 


 
   
  kable(Table.Prosencephalon.absolute.stratified.gender.RSD.male)  
 
 
 

 
 
 
 
 
 
 Table.Prosencephalon.absolute.stratified.gender.RSD.male  X 2/ T  a  b  l  e . P  r  o  s  e  n  c  e  p  h  a  l  o  n . a  b  s  o  l  u  t  e . s  t  r  a  t  i  f  i  e  d . g  e  n  d  e  r . R  S  D . m  a  l  e  X1 
 
 
 
 
 21.9 
 
 
 11.8 
 
 
 11.7 
 
 
 14.1 
 
 
 17.6 
 
 
 12.2 
 
 
 20.4 
 
 
 20.5 
 
 
 20.6 
 
 
 20.5 
 
 
 55.1 
 
 
 10.2 
 
 
 10.4 
 
 
 11.5 
 
 
 10.8 
 
 
 13.8 
 
 
 10.8 
 
 
 13.9 
 
 
 10.7 
 
 
 11.2 
 
 
 14.2 
 
 
 11.6 
 
 
 14.4 
 
 
 15.3 
 
 
 13.6 
 
 
 15.0 
 
 
 14.4 
 
 
 12.4 
 
 
 13.2 
 
 
 13.4 
 
 
 14.3 
 
 
 25.9 
 
 
 20.6 
 
 
 15.3 
 
 
 18.9 
 
 
 25.4 
 
 
 11.7 
 
 
 14.4 
 
 
 13.1 
 
 
 17.8 
 
 
 12.8 
 
 
 14.4 
 
 
 16.5 
 
 
 31.3 
 
 
 16.3 
 
 
 18.7 
 
 
 21.8 
 
 
 12.5 
 
 
 10.5 
 
 
 11.2 
 
 
 10.7 
 
 
 


 
   
  NA  
 
 
 
 
 
 
  
Prosencephalon.absolute <- select(Prosencephalon.absolute, - c(Gender))
Prosencephalon.absolute1 <- Prosencephalon.absolute[,-c(41:51)]
Prosencephalon.absolute2 <- Prosencephalon.absolute[, c(41:51)]

names.anatomical.structures.temporary <- c("Frontal.pole",
  "F1",
  "F2",
  "F3.orbital", 
  "F3.triangular", 
  "F3.opercular", 
  "Anterior.orbital", 
  "Medial.orbital", 
  "Lateral.orbital", 
  "Posterior.orbital",
  "Rectus", 
  "Rostral", 
  "Precentral", 
  "Postcentral", 
  "Paracentral.lobule", 
  "Subcentral", 
  "SPL", 
  "SMG", 
  "ANG", 
  "Precuneus",
  "Cuneus", 
  "O1",
  "O2", 
  "O3", 
  "Occipital.pole", 
  "Lingual", 
  "Fusiform", 
  "Temporal.pole", 
  "T1",
  "T2", 
  "T3", 
  "Planum.temporale", 
  "Planum.polare", 
  "Short.insular", 
  "Long.insular", 
  "SCA", 
  "Cingulate.anterior", 
  "Cingulate.middle", 
  "Cingulate.posterior", 
  "PHG")

names.anatomical.structures.definitive <- c("Frontal pole",
  "F1",
  "F2",
  "F3 orbital", 
  "F3 triangular", 
  "F3 opercular", 
  "Anterior orbital", 
  "Medial orbital", 
  "Lateral orbital", 
  "Posterior orbital",
  "Rectus", 
  "Rostral", 
  "Precentral", 
  "Postcentral", 
  "Paracentral lobule", 
  "Subcentral", 
  "SPL", 
  "SMG", 
  "ANG", 
  "Precuneus",
  "Cuneus", 
  "O1",
  "O2", 
  "O3", 
  "Occipital pole", 
  "Lingual", 
  "Fusiform", 
  "Temporal pole", 
  "T1",
  "T2", 
  "T3", 
  "Planum temporale", 
  "Planum polare", 
  "Short insular", 
  "Long insular", 
  "SCA", 
  "Cingulate anterior", 
  "Cingulate middle", 
  "Cingulate posterior", 
  "PHG")


Prosencephalon.absolute.plotdata1 <- gather(Prosencephalon.absolute1, "anatomical.structure", "relative.volume")
Prosencephalon.absolute.plotdata1$Gender <- All.Volumes$Gender
Prosencephalon.absolute.plotdata1$Age <- All.Volumes$`Age (years)`
Prosencephalon.absolute.plotdata1$Gender <- factor(Prosencephalon.absolute.plotdata1$Gender, levels = c("f", "m"), c("f", "m"))

Prosencephalon.absolute.plotdata1$anatomical.structure <- factor(Prosencephalon.absolute.plotdata1$anatomical.structure, 
                                                                levels = rev(c(names.anatomical.structures.temporary)), rev(c(names.anatomical.structures.definitive)))

Prosencephalon.absolute.plot1 <-  ggplot(Prosencephalon.absolute.plotdata1, aes(x=anatomical.structure, y = relative.volume))  +
  stat_summary(alpha = 0.3, fun = mean, geom = "bar", width = 0.3, fill = "gray50") + 
  geom_boxplot(aes(fill = Gender), alpha = 0.5, width = 0.4, size = 0.2, position = position_dodge(width = 0.6), 
               outlier.shape = NA, color = "gray30") +
  scale_fill_manual(values = c("chartreuse4", "orangered2")) +
  geom_quasirandom(aes(color = Age), size = 0.7, alpha = 0.8, shape = 16, position = "dodge") +
  scale_color_continuous(low = "steelblue1", high = "red4") +
  xlab("") + ylab("Absolute volume (in mm3)") +
  theme_minimal() +
  coord_flip() +
  ggtitle("CEREBRAL GYRI") +
  theme(plot.title = element_text(hjust = 0.5))

Prosencephalon.absolute.plot1
ggsave("Prosencephalon.absolute.plot1.pdf", plot = Prosencephalon.absolute.plot1, width = 14, height = 12, units = "in", dpi = 600)
  
 
 
   
 
 
 
 
 
 
  
names.anatomical.structures.temporary <- c(
  "Corpus.callosum", 
  "Claustrum", 
  "Putamen", 
  "Caudate", 
  "Globus.pallidum", 
  "Internal.capsule",
  "Innominate.substance", 
  "Hypothalamus",
  "Thalamus", 
  "Hippocampus", 
  "Amygdala")

names.anatomical.structures.definitive <- c(
  "Corpus callosum", 
  "Claustrum", 
  "Putamen", 
  "Caudate", 
  "Globus pallidum", 
  "Internal capsule",
  "Innominate substance", 
  "Hypothalamus",
  "Thalamus",
  "Hippocampus", 
  "Amygdala")

Prosencephalon.absolute.plotdata2 <- gather(Prosencephalon.absolute2, "anatomical.structure", "relative.volume")
Prosencephalon.absolute.plotdata2$Gender <- All.Volumes$Gender
Prosencephalon.absolute.plotdata2$Age <- All.Volumes$`Age (years)`

Prosencephalon.absolute.plotdata2$Gender <- factor(Prosencephalon.absolute.plotdata2$Gender, levels = c("f", "m"), c("f", "m"))
Prosencephalon.absolute.plotdata2$anatomical.structure <- factor(Prosencephalon.absolute.plotdata2$anatomical.structure, 
                                                                levels = rev(c(names.anatomical.structures.temporary)), rev(c(names.anatomical.structures.definitive)))

Prosencephalon.absolute.plot2 <-  ggplot(Prosencephalon.absolute.plotdata2, aes(x=anatomical.structure, y = relative.volume))  +
  stat_summary(alpha = 0.3, fun = mean, geom = "bar", width = 0.3, fill = "gray50") + 
  geom_boxplot(aes(fill = Gender), alpha = 0.5, width = 0.4, size = 0.2, position = position_dodge(width = 0.6), 
               outlier.shape = NA, color = "gray30") +
  scale_fill_manual(values = c("chartreuse4", "orangered2")) +
  geom_quasirandom(aes(color = Age), size = 0.7, alpha = 0.8, shape = 16, position = "dodge") +
  scale_color_continuous(low = "steelblue1", high = "red4") +
  xlab("") + ylab("Absolute volume (in mm3)") +
  theme_minimal() +
  coord_flip() +
  ggtitle("CENTRAL PROSENCEPHALON") +
  theme(plot.title = element_text(hjust = 0.5))

Prosencephalon.absolute.plot2
ggsave("Prosencephalon.absolute.plot2.pdf", plot = Prosencephalon.absolute.plot2, width = 10, height = 6, units = "in", dpi = 600)
  
 
 
   
 
 
 
 
 
 
  
Prosencephalon.absolute$Gender <- All.Volumes$Gender
Prosencephalon.absolute$Age <- All.Volumes$`Age (years)`

Total.Frontal.pole.Age.plot <-  ggplot(Prosencephalon.absolute, aes(y=Frontal.pole, x = Age))  +
  geom_point(aes(color = Gender), size = 1.5, alpha = 1, shape = 16) +
  scale_color_manual(values = c("chartreuse4", "orangered2")) +
  geom_smooth(method='lm', alpha = 0.2, colour = "dodgerblue4", size = 0.8, weight = 0.3) +
  geom_smooth(aes(color = Gender), method='lm', se = F, alpha = 0.2, linetype = "longdash", size = 0.3, weight = 0.3) +
  stat_cor(method = "pearson", label.y = 6700, label.x = 70, color = "dodgerblue4") +
  ylab("Volume in mm3") + xlab("Age (in years)") +
  theme_minimal() +
  ggtitle("ABSOLUTE VOLUME frontal pole") +
  theme(plot.title = element_text(hjust = 0.5))
Total.Frontal.pole.Age.plot
ggsave("Total.Frontal.pole.Age.plot.pdf", plot = Total.Frontal.pole.Age.plot, width = 8, height = 6, units = "in", dpi = 600)  
 
 
   
 
 
  
Total.F1.Age.plot <-  ggplot(Prosencephalon.absolute, aes(y=F1, x = Age))  +
  geom_point(aes(color = Gender), size = 1.5, alpha = 1, shape = 16) +
  scale_color_manual(values = c("chartreuse4", "orangered2")) +
  geom_smooth(method='lm', alpha = 0.2, colour = "dodgerblue4", size = 0.8, weight = 0.3) +
  geom_smooth(aes(color = Gender), method='lm', se = F, alpha = 0.2, linetype = "longdash", size = 0.3, weight = 0.3) +
  stat_cor(method = "pearson", label.y = 77000, label.x = 70, color = "dodgerblue4") +
  ylab("Volume in mm3") + xlab("Age (in years)") +
  theme_minimal() +
  ggtitle("ABSOLUTE VOLUME F1") +
  theme(plot.title = element_text(hjust = 0.5))
Total.F1.Age.plot
ggsave("Total.F1.Age.plot.pdf", plot = Total.F1.Age.plot, width = 8, height = 6, units = "in", dpi = 600)  
 
 
   
 
 
  
Total.F2.Age.plot <-  ggplot(Prosencephalon.absolute, aes(y=F2, x = Age))  +
  geom_point(aes(color = Gender), size = 1.5, alpha = 1, shape = 16) +
  scale_color_manual(values = c("chartreuse4", "orangered2")) +
  geom_smooth(method='lm', alpha = 0.2, colour = "dodgerblue4", size = 0.8, weight = 0.3) +
  geom_smooth(aes(color = Gender), method='lm', se = F, alpha = 0.2, linetype = "longdash", size = 0.3, weight = 0.3) +
  stat_cor(method = "pearson", label.y = 72000, label.x = 70, color = "dodgerblue4") +
  ylab("Volume in mm3") + xlab("Age (in years)") +
  theme_minimal() +
  ggtitle("ABSOLUTE VOLUME F2") +
  theme(plot.title = element_text(hjust = 0.5))
Total.F2.Age.plot
ggsave("Total.F2.Age.plot.pdf", plot = Total.F2.Age.plot, width = 8, height = 6, units = "in", dpi = 600)  
 
 
   
 
 
  
Total.F3.orbital.Age.plot <-  ggplot(Prosencephalon.absolute, aes(y=F3.orbital, x = Age))  +
  geom_point(aes(color = Gender), size = 1.5, alpha = 1, shape = 16) +
  scale_color_manual(values = c("chartreuse4", "orangered2")) +
  geom_smooth(method='lm', alpha = 0.2, colour = "dodgerblue4", size = 0.8, weight = 0.3) +
  geom_smooth(aes(color = Gender), method='lm', se = F, alpha = 0.2, linetype = "longdash", size = 0.3, weight = 0.3) +
  stat_cor(method = "pearson", label.y = 4600, label.x = 70, color = "dodgerblue4") +
  ylab("Volume in mm3") + xlab("Age (in years)") +
  theme_minimal() +
  ggtitle("ABSOLUTE VOLUME F3 orbital") +
  theme(plot.title = element_text(hjust = 0.5))
Total.F3.orbital.Age.plot
ggsave("Total.F3.orbital.Age.plot.pdf", plot = Total.F3.orbital.Age.plot, width = 8, height = 6, units = "in", dpi = 600)  
 
 
   
 
 
  
Total.F3.triangular.Age.plot <-  ggplot(Prosencephalon.absolute, aes(y=F3.triangular, x = Age))  +
  geom_point(aes(color = Gender), size = 1.5, alpha = 1, shape = 16) +
  scale_color_manual(values = c("chartreuse4", "orangered2")) +
  geom_smooth(method='lm', alpha = 0.2, colour = "dodgerblue4", size = 0.8, weight = 0.3) +
  geom_smooth(aes(color = Gender), method='lm', se = F, alpha = 0.2, linetype = "longdash", size = 0.3, weight = 0.3) +
  stat_cor(method = "pearson", label.y = 13300, label.x = 70, color = "dodgerblue4") +
  ylab("Volume in mm3") + xlab("Age (in years)") +
  theme_minimal() +
  ggtitle("ABSOLUTE VOLUME F3 triangular") +
  theme(plot.title = element_text(hjust = 0.5))
Total.F3.triangular.Age.plot
ggsave("Total.F3.triangular.Age.plot.pdf", plot = Total.F3.triangular.Age.plot, width = 8, height = 6, units = "in", dpi = 600)  
 
 
   
 
 
  
Total.F3.opercular.Age.plot <-  ggplot(Prosencephalon.absolute, aes(y=F3.opercular, x = Age))  +
  geom_point(aes(color = Gender), size = 1.5, alpha = 1, shape = 16) +
  scale_color_manual(values = c("chartreuse4", "orangered2")) +
  geom_smooth(method='lm', alpha = 0.2, colour = "dodgerblue4", size = 0.8, weight = 0.3) +
  geom_smooth(aes(color = Gender), method='lm', se = F, alpha = 0.2, linetype = "longdash", size = 0.3, weight = 0.3) +
  stat_cor(method = "pearson", label.y = 17300, label.x = 70, color = "dodgerblue4") +
  ylab("Volume in mm3") + xlab("Age (in years)") +
  theme_minimal() +
  ggtitle("ABSOLUTE VOLUME F3 opercular") +
  theme(plot.title = element_text(hjust = 0.5))
Total.F3.opercular.Age.plot
ggsave("Total.F3.opercular.Age.plot.pdf", plot = Total.F3.opercular.Age.plot, width = 8, height = 6, units = "in", dpi = 600)  
 
 
   
 
 
  
Total.Anterior.orbital.Age.plot <-  ggplot(Prosencephalon.absolute, aes(y=Anterior.orbital, x = Age))  +
  geom_point(aes(color = Gender), size = 1.5, alpha = 1, shape = 16) +
  scale_color_manual(values = c("chartreuse4", "orangered2")) +
  geom_smooth(method='lm', alpha = 0.2, colour = "dodgerblue4", size = 0.8, weight = 0.3) +
  geom_smooth(aes(color = Gender), method='lm', se = F, alpha = 0.2, linetype = "longdash", size = 0.3, weight = 0.3) +
  stat_cor(method = "pearson", label.y = 5200, label.x = 70, color = "dodgerblue4") +
  ylab("Volume in mm3") + xlab("Age (in years)") +
  theme_minimal() +
  ggtitle("ABSOLUTE VOLUME anterior orbital") +
  theme(plot.title = element_text(hjust = 0.5))
Total.Anterior.orbital.Age.plot
ggsave("Total.Anterior.orbital.Age.plot.pdf", plot = Total.Anterior.orbital.Age.plot, width = 8, height = 6, units = "in", dpi = 600)  
 
 
   
 
 
  
Total.Medial.orbital.Age.plot <-  ggplot(Prosencephalon.absolute, aes(y=Medial.orbital, x = Age))  +
  geom_point(aes(color = Gender), size = 1.5, alpha = 1, shape = 16) +
  scale_color_manual(values = c("chartreuse4", "orangered2")) +
  geom_smooth(method='lm', alpha = 0.2, colour = "dodgerblue4", size = 0.8, weight = 0.3) +
  geom_smooth(aes(color = Gender), method='lm', se = F, alpha = 0.2, linetype = "longdash", size = 0.3, weight = 0.3) +
  stat_cor(method = "pearson", label.y = 10500, label.x = 70, color = "dodgerblue4") +
  ylab("Volume in mm3") + xlab("Age (in years)") +
  theme_minimal() +
  ggtitle("ABSOLUTE VOLUME medial orbital") +
  theme(plot.title = element_text(hjust = 0.5))
Total.Medial.orbital.Age.plot
ggsave("Total.Medial.orbital.Age.plot.pdf", plot = Total.Medial.orbital.Age.plot, width = 8, height = 6, units = "in", dpi = 600)  
 
 
   
 
 
  
Total.Lateral.orbital.Age.plot <-  ggplot(Prosencephalon.absolute, aes(y=Lateral.orbital, x = Age))  +
  geom_point(aes(color = Gender), size = 1.5, alpha = 1, shape = 16) +
  scale_color_manual(values = c("chartreuse4", "orangered2")) +
  geom_smooth(method='lm', alpha = 0.2, colour = "dodgerblue4", size = 0.8, weight = 0.3) +
  geom_smooth(aes(color = Gender), method='lm', se = F, alpha = 0.2, linetype = "longdash", size = 0.3, weight = 0.3) +
  stat_cor(method = "pearson", label.y = 8200, label.x = 70, color = "dodgerblue4") +
  ylab("Volume in mm3") + xlab("Age (in years)") +
  theme_minimal() +
  ggtitle("ABSOLUTE VOLUME lateral orbital") +
  theme(plot.title = element_text(hjust = 0.5))
Total.Lateral.orbital.Age.plot
ggsave("Total.Lateral.orbital.Age.plot.pdf", plot = Total.Lateral.orbital.Age.plot, width = 8, height = 6, units = "in", dpi = 600)  
 
 
   
 
 
  
Total.Posterior.orbital.Age.plot <-  ggplot(Prosencephalon.absolute, aes(y=Posterior.orbital, x = Age))  +
  geom_point(aes(color = Gender), size = 1.5, alpha = 1, shape = 16) +
  scale_color_manual(values = c("chartreuse4", "orangered2")) +
  geom_smooth(method='lm', alpha = 0.2, colour = "dodgerblue4", size = 0.8, weight = 0.3) +
  geom_smooth(aes(color = Gender), method='lm', se = F, alpha = 0.2, linetype = "longdash", size = 0.3, weight = 0.3) +
  stat_cor(method = "pearson", label.y = 10200, label.x = 70, color = "dodgerblue4") +
  ylab("Volume in mm3") + xlab("Age (in years)") +
  theme_minimal() +
  ggtitle("ABSOLUTE VOLUME posterior orbital") +
  theme(plot.title = element_text(hjust = 0.5))
Total.Posterior.orbital.Age.plot
ggsave("Total.Posterior.orbital.Age.plot.pdf", plot = Total.Posterior.orbital.Age.plot, width = 8, height = 6, units = "in", dpi = 600)  
 
 
   
 
 
  
Total.Rectus.Age.plot <-  ggplot(Prosencephalon.absolute, aes(y=Rectus, x = Age))  +
  geom_point(aes(color = Gender), size = 1.5, alpha = 1, shape = 16) +
  scale_color_manual(values = c("chartreuse4", "orangered2")) +
  geom_smooth(method='lm', alpha = 0.2, colour = "dodgerblue4", size = 0.8, weight = 0.3) +
  geom_smooth(aes(color = Gender), method='lm', se = F, alpha = 0.2, linetype = "longdash", size = 0.3, weight = 0.3) +
  stat_cor(method = "pearson", label.y = 22000, label.x = 70, color = "dodgerblue4") +
  ylab("Volume in mm3") + xlab("Age (in years)") +
  theme_minimal() +
  ggtitle("ABSOLUTE VOLUME rectus") +
  theme(plot.title = element_text(hjust = 0.5))
Total.Rectus.Age.plot
ggsave("Total.Rectus.Age.plot.pdf", plot = Total.Rectus.Age.plot, width = 8, height = 6, units = "in", dpi = 600)  
 
 
   
 
 
  
Total.Rostral.Age.plot <-  ggplot(Prosencephalon.absolute, aes(y=Rostral, x = Age))  +
  geom_point(aes(color = Gender), size = 1.5, alpha = 1, shape = 16) +
  scale_color_manual(values = c("chartreuse4", "orangered2")) +
  geom_smooth(method='lm', alpha = 0.2, colour = "dodgerblue4", size = 0.8, weight = 0.3) +
  geom_smooth(aes(color = Gender), method='lm', se = F, alpha = 0.2, linetype = "longdash", size = 0.3, weight = 0.3) +
  stat_cor(method = "pearson", label.y = 4300, label.x = 70, color = "dodgerblue4") +
  ylab("Volume in mm3") + xlab("Age (in years)") +
  theme_minimal() +
  ggtitle("ABSOLUTE VOLUME rostral") +
  theme(plot.title = element_text(hjust = 0.5))
Total.Rostral.Age.plot
ggsave("Total.Rostral.Age.plot.pdf", plot = Total.Rostral.Age.plot, width = 8, height = 6, units = "in", dpi = 600)  
 
 
   
 
 
  
Total.Precentral.Age.plot <-  ggplot(Prosencephalon.absolute, aes(y=Precentral, x = Age))  +
  geom_point(aes(color = Gender), size = 1.5, alpha = 1, shape = 16) +
  scale_color_manual(values = c("chartreuse4", "orangered2")) +
  geom_smooth(method='lm', alpha = 0.2, colour = "dodgerblue4", size = 0.8, weight = 0.3) +
  geom_smooth(aes(color = Gender), method='lm', se = F, alpha = 0.2, linetype = "longdash", size = 0.3, weight = 0.3) +
  stat_cor(method = "pearson", label.y = 48000, label.x = 70, color = "dodgerblue4") +
  ylab("Volume in mm3") + xlab("Age (in years)") +
  theme_minimal() +
  ggtitle("ABSOLUTE VOLUME precentral") +
  theme(plot.title = element_text(hjust = 0.5))
Total.Precentral.Age.plot
ggsave("Total.Precentral.Age.plot.pdf", plot = Total.Precentral.Age.plot, width = 8, height = 6, units = "in", dpi = 600)  
 
 
   
 
 
  
Total.Postcentral.Age.plot <-  ggplot(Prosencephalon.absolute, aes(y=Postcentral, x = Age))  +
  geom_point(aes(color = Gender), size = 1.5, alpha = 1, shape = 16) +
  scale_color_manual(values = c("chartreuse4", "orangered2")) +
  geom_smooth(method='lm', alpha = 0.2, colour = "dodgerblue4", size = 0.8, weight = 0.3) +
  geom_smooth(aes(color = Gender), method='lm', se = F, alpha = 0.2, linetype = "longdash", size = 0.3, weight = 0.3) +
  stat_cor(method = "pearson", label.y = 26500, label.x = 70, color = "dodgerblue4") +
  ylab("Volume in mm3") + xlab("Age (in years)") +
  theme_minimal() +
  ggtitle("ABSOLUTE VOLUME postcentral") +
  theme(plot.title = element_text(hjust = 0.5))
Total.Postcentral.Age.plot
ggsave("Total.Postcentral.Age.plot.pdf", plot = Total.Postcentral.Age.plot, width = 8, height = 6, units = "in", dpi = 600)  
 
 
   
 
 
  
Total.Paracentral.lobule.Age.plot <-  ggplot(Prosencephalon.absolute, aes(y=Paracentral.lobule, x = Age))  +
  geom_point(aes(color = Gender), size = 1.5, alpha = 1, shape = 16) +
  scale_color_manual(values = c("chartreuse4", "orangered2")) +
  geom_smooth(method='lm', alpha = 0.2, colour = "dodgerblue4", size = 0.8, weight = 0.3) +
  geom_smooth(aes(color = Gender), method='lm', se = F, alpha = 0.2, linetype = "longdash", size = 0.3, weight = 0.3) +
  stat_cor(method = "pearson", label.y = 17200, label.x = 70, color = "dodgerblue4") +
  ylab("Volume in mm3") + xlab("Age (in years)") +
  theme_minimal() +
  ggtitle("ABSOLUTE VOLUME paracentral lobule") +
  theme(plot.title = element_text(hjust = 0.5))
Total.Paracentral.lobule.Age.plot
ggsave("Total.Paracentral.lobule.Age.plot.pdf", plot = Total.Paracentral.lobule.Age.plot, width = 8, height = 6, units = "in", dpi = 600)  
 
 
   
 
 
  
Total.Subcentral.Age.plot <-  ggplot(Prosencephalon.absolute, aes(y=Subcentral, x = Age))  +
  geom_point(aes(color = Gender), size = 1.5, alpha = 1, shape = 16) +
  scale_color_manual(values = c("chartreuse4", "orangered2")) +
  geom_smooth(method='lm', alpha = 0.2, colour = "dodgerblue4", size = 0.8, weight = 0.3) +
  geom_smooth(aes(color = Gender), method='lm', se = F, alpha = 0.2, linetype = "longdash", size = 0.3, weight = 0.3) +
  stat_cor(method = "pearson", label.y = 7200, label.x = 70, color = "dodgerblue4") +
  ylab("Volume in mm3") + xlab("Age (in years)") +
  theme_minimal() +
  ggtitle("ABSOLUTE VOLUME subcentral") +
  theme(plot.title = element_text(hjust = 0.5))
Total.Subcentral.Age.plot
ggsave("Total.Subcentral.Age.plot.pdf", plot = Total.Subcentral.Age.plot, width = 8, height = 6, units = "in", dpi = 600)  
 
 
   
 
 
  
Total.SPL.Age.plot <-  ggplot(Prosencephalon.absolute, aes(y=SPL, x = Age))  +
  geom_point(aes(color = Gender), size = 1.5, alpha = 1, shape = 16) +
  scale_color_manual(values = c("chartreuse4", "orangered2")) +
  geom_smooth(method='lm', alpha = 0.2, colour = "dodgerblue4", size = 0.8, weight = 0.3) +
  geom_smooth(aes(color = Gender), method='lm', se = F, alpha = 0.2, linetype = "longdash", size = 0.3, weight = 0.3) +
  stat_cor(method = "pearson", label.y = 38000, label.x = 70, color = "dodgerblue4") +
  ylab("Volume in mm3") + xlab("Age (in years)") +
  theme_minimal() +
  ggtitle("ABSOLUTE VOLUME superior parietal lobule") +
  theme(plot.title = element_text(hjust = 0.5))
Total.SPL.Age.plot
ggsave("Total.SPL.Age.plot.pdf", plot = Total.SPL.Age.plot, width = 8, height = 6, units = "in", dpi = 600)  
 
 
   
 
 
  
Total.SMG.Age.plot <-  ggplot(Prosencephalon.absolute, aes(y=SMG, x = Age))  +
  geom_point(aes(color = Gender), size = 1.5, alpha = 1, shape = 16) +
  scale_color_manual(values = c("chartreuse4", "orangered2")) +
  geom_smooth(method='lm', alpha = 0.2, colour = "dodgerblue4", size = 0.8, weight = 0.3) +
  geom_smooth(aes(color = Gender), method='lm', se = F, alpha = 0.2, linetype = "longdash", size = 0.3, weight = 0.3) +
  stat_cor(method = "pearson", label.y = 36000, label.x = 70, color = "dodgerblue4") +
  ylab("Volume in mm3") + xlab("Age (in years)") +
  theme_minimal() +
  ggtitle("ABSOLUTE VOLUME supramarginal") +
  theme(plot.title = element_text(hjust = 0.5))
Total.SMG.Age.plot
ggsave("Total.SMG.Age.plot.pdf", plot = Total.SMG.Age.plot, width = 8, height = 6, units = "in", dpi = 600)  
 
 
   
 
 
  
Total.ANG.Age.plot <-  ggplot(Prosencephalon.absolute, aes(y=ANG, x = Age))  +
  geom_point(aes(color = Gender), size = 1.5, alpha = 1, shape = 16) +
  scale_color_manual(values = c("chartreuse4", "orangered2")) +
  geom_smooth(method='lm', alpha = 0.2, colour = "dodgerblue4", size = 0.8, weight = 0.3) +
  geom_smooth(aes(color = Gender), method='lm', se = F, alpha = 0.2, linetype = "longdash", size = 0.3, weight = 0.3) +
  stat_cor(method = "pearson", label.y = 38000, label.x = 70, color = "dodgerblue4") +
  ylab("Volume in mm3") + xlab("Age (in years)") +
  theme_minimal() +
  ggtitle("ABSOLUTE VOLUME angular") +
  theme(plot.title = element_text(hjust = 0.5))
Total.ANG.Age.plot
ggsave("Total.ANG.Age.plot.pdf", plot = Total.ANG.Age.plot, width = 8, height = 6, units = "in", dpi = 600)  
 
 
   
 
 
  
Total.Precuneus.Age.plot <-  ggplot(Prosencephalon.absolute, aes(y=Precuneus, x = Age))  +
  geom_point(aes(color = Gender), size = 1.5, alpha = 1, shape = 16) +
  scale_color_manual(values = c("chartreuse4", "orangered2")) +
  geom_smooth(method='lm', alpha = 0.2, colour = "dodgerblue4", size = 0.8, weight = 0.3) +
  geom_smooth(aes(color = Gender), method='lm', se = F, alpha = 0.2, linetype = "longdash", size = 0.3, weight = 0.3) +
  stat_cor(method = "pearson", label.y = 33000, label.x = 70, color = "dodgerblue4") +
  ylab("Volume in mm3") + xlab("Age (in years)") +
  theme_minimal() +
  ggtitle("ABSOLUTE VOLUME precuneus") +
  theme(plot.title = element_text(hjust = 0.5))
Total.Precuneus.Age.plot
ggsave("Total.Precuneus.Age.plot.pdf", plot = Total.Precuneus.Age.plot, width = 8, height = 6, units = "in", dpi = 600)  
 
 
   
 
 
  
Total.Cuneus.Age.plot <-  ggplot(Prosencephalon.absolute, aes(y=Cuneus, x = Age))  +
  geom_point(aes(color = Gender), size = 1.5, alpha = 1, shape = 16) +
  scale_color_manual(values = c("chartreuse4", "orangered2")) +
  geom_smooth(method='lm', alpha = 0.2, colour = "dodgerblue4", size = 0.8, weight = 0.3) +
  geom_smooth(aes(color = Gender), method='lm', se = F, alpha = 0.2, linetype = "longdash", size = 0.3, weight = 0.3) +
  stat_cor(method = "pearson", label.y = 11700, label.x = 70, color = "dodgerblue4") +
  ylab("Volume in mm3") + xlab("Age (in years)") +
  theme_minimal() +
  ggtitle("ABSOLUTE VOLUME cuneus") +
  theme(plot.title = element_text(hjust = 0.5))
Total.Cuneus.Age.plot
ggsave("Total.Cuneus.Age.plot.pdf", plot = Total.Cuneus.Age.plot, width = 8, height = 6, units = "in", dpi = 600)  
 
 
   
 
 
  
Total.O1.Age.plot <-  ggplot(Prosencephalon.absolute, aes(y=O1, x = Age))  +
  geom_point(aes(color = Gender), size = 1.5, alpha = 1, shape = 16) +
  scale_color_manual(values = c("chartreuse4", "orangered2")) +
  geom_smooth(method='lm', alpha = 0.2, colour = "dodgerblue4", size = 0.8, weight = 0.3) +
  geom_smooth(aes(color = Gender), method='lm', se = F, alpha = 0.2, linetype = "longdash", size = 0.3, weight = 0.3) +
  stat_cor(method = "pearson", label.y = 11200, label.x = 70, color = "dodgerblue4") +
  ylab("Volume in mm3") + xlab("Age (in years)") +
  theme_minimal() +
  ggtitle("ABSOLUTE VOLUME O1") +
  theme(plot.title = element_text(hjust = 0.5))
Total.O1.Age.plot
ggsave("Total.O1.Age.plot.pdf", plot = Total.O1.Age.plot, width = 8, height = 6, units = "in", dpi = 600)  
 
 
   
 
 
  
Total.O2.Age.plot <-  ggplot(Prosencephalon.absolute, aes(y=O2, x = Age))  +
  geom_point(aes(color = Gender), size = 1.5, alpha = 1, shape = 16) +
  scale_color_manual(values = c("chartreuse4", "orangered2")) +
  geom_smooth(method='lm', alpha = 0.2, colour = "dodgerblue4", size = 0.8, weight = 0.3) +
  geom_smooth(aes(color = Gender), method='lm', se = F, alpha = 0.2, linetype = "longdash", size = 0.3, weight = 0.3) +
  stat_cor(method = "pearson", label.y = 18000, label.x = 70, color = "dodgerblue4") +
  ylab("Volume in mm3") + xlab("Age (in years)") +
  theme_minimal() +
  ggtitle("ABSOLUTE VOLUME O2") +
  theme(plot.title = element_text(hjust = 0.5))
Total.O2.Age.plot
ggsave("Total.O2.Age.plot.pdf", plot = Total.O2.Age.plot, width = 8, height = 6, units = "in", dpi = 600)  
 
 
   
 
 
  
Total.O3.Age.plot <-  ggplot(Prosencephalon.absolute, aes(y=O3, x = Age))  +
  geom_point(aes(color = Gender), size = 1.5, alpha = 1, shape = 16) +
  scale_color_manual(values = c("chartreuse4", "orangered2")) +
  geom_smooth(method='lm', alpha = 0.2, colour = "dodgerblue4", size = 0.8, weight = 0.3) +
  geom_smooth(aes(color = Gender), method='lm', se = F, alpha = 0.2, linetype = "longdash", size = 0.3, weight = 0.3) +
  stat_cor(method = "pearson", label.y = 11500, label.x = 70, color = "dodgerblue4") +
  ylab("Volume in mm3") + xlab("Age (in years)") +
  theme_minimal() +
  ggtitle("ABSOLUTE VOLUME O3") +
  theme(plot.title = element_text(hjust = 0.5))
Total.O3.Age.plot
ggsave("Total.O3.Age.plot.pdf", plot = Total.O3.Age.plot, width = 8, height = 6, units = "in", dpi = 600)  
 
 
   
 
 
  
Total.Occipital.pole.Age.plot <-  ggplot(Prosencephalon.absolute, aes(y=Occipital.pole, x = Age))  +
  geom_point(aes(color = Gender), size = 1.5, alpha = 1, shape = 16) +
  scale_color_manual(values = c("chartreuse4", "orangered2")) +
  geom_smooth(method='lm', alpha = 0.2, colour = "dodgerblue4", size = 0.8, weight = 0.3) +
  geom_smooth(aes(color = Gender), method='lm', se = F, alpha = 0.2, linetype = "longdash", size = 0.3, weight = 0.3) +
  stat_cor(method = "pearson", label.y = 13200, label.x = 70, color = "dodgerblue4") +
  ylab("Volume in mm3") + xlab("Age (in years)") +
  theme_minimal() +
  ggtitle("ABSOLUTE VOLUME occipital pole") +
  theme(plot.title = element_text(hjust = 0.5))
Total.Occipital.pole.Age.plot
ggsave("Total.Occipital.pole.Age.plot.pdf", plot = Total.Occipital.pole.Age.plot, width = 8, height = 6, units = "in", dpi = 600)  
 
 
   
 
 
  
Total.Lingual.Age.plot <-  ggplot(Prosencephalon.absolute, aes(y=Lingual, x = Age))  +
  geom_point(aes(color = Gender), size = 1.5, alpha = 1, shape = 16) +
  scale_color_manual(values = c("chartreuse4", "orangered2")) +
  geom_smooth(method='lm', alpha = 0.2, colour = "dodgerblue4", size = 0.8, weight = 0.3) +
  geom_smooth(aes(color = Gender), method='lm', se = F, alpha = 0.2, linetype = "longdash", size = 0.3, weight = 0.3) +
  stat_cor(method = "pearson", label.y = 25000, label.x = 70, color = "dodgerblue4") +
  ylab("Volume in mm3") + xlab("Age (in years)") +
  theme_minimal() +
  ggtitle("ABSOLUTE VOLUME lingual") +
  theme(plot.title = element_text(hjust = 0.5))
Total.Lingual.Age.plot
ggsave("Total.Lingual.Age.plot.pdf", plot = Total.Lingual.Age.plot, width = 8, height = 6, units = "in", dpi = 600)  
 
 
   
 
 
  
Total.Fusiform.Age.plot <-  ggplot(Prosencephalon.absolute, aes(y=Fusiform, x = Age))  +
  geom_point(aes(color = Gender), size = 1.5, alpha = 1, shape = 16) +
  scale_color_manual(values = c("chartreuse4", "orangered2")) +
  geom_smooth(method='lm', alpha = 0.2, colour = "dodgerblue4", size = 0.8, weight = 0.3) +
  geom_smooth(aes(color = Gender), method='lm', se = F, alpha = 0.2, linetype = "longdash", size = 0.3, weight = 0.3) +
  stat_cor(method = "pearson", label.y = 26000, label.x = 70, color = "dodgerblue4") +
  ylab("Volume in mm3") + xlab("Age (in years)") +
  theme_minimal() +
  ggtitle("ABSOLUTE VOLUME fusiform") +
  theme(plot.title = element_text(hjust = 0.5))
Total.Fusiform.Age.plot
ggsave("Total.Fusiform.Age.plot.pdf", plot = Total.Fusiform.Age.plot, width = 8, height = 6, units = "in", dpi = 600)  
 
 
   
 
 
  
Total.Temporal.pole.Age.plot <-  ggplot(Prosencephalon.absolute, aes(y=Temporal.pole, x = Age))  +
  geom_point(aes(color = Gender), size = 1.5, alpha = 1, shape = 16) +
  scale_color_manual(values = c("chartreuse4", "orangered2")) +
  geom_smooth(method='lm', alpha = 0.2, colour = "dodgerblue4", size = 0.8, weight = 0.3) +
  geom_smooth(aes(color = Gender), method='lm', se = F, alpha = 0.2, linetype = "longdash", size = 0.3, weight = 0.3) +
  stat_cor(method = "pearson", label.y = 31000, label.x = 70, color = "dodgerblue4") +
  ylab("Volume in mm3") + xlab("Age (in years)") +
  theme_minimal() +
  ggtitle("ABSOLUTE VOLUME temporal pole") +
  theme(plot.title = element_text(hjust = 0.5))
Total.Temporal.pole.Age.plot
ggsave("Total.Temporal.pole.Age.plot.pdf", plot = Total.Temporal.pole.Age.plot, width = 8, height = 6, units = "in", dpi = 600)  
 
 
   
 
 
  
Total.T1.Age.plot <-  ggplot(Prosencephalon.absolute, aes(y=T1, x = Age))  +
  geom_point(aes(color = Gender), size = 1.5, alpha = 1, shape = 16) +
  scale_color_manual(values = c("chartreuse4", "orangered2")) +
  geom_smooth(method='lm', alpha = 0.2, colour = "dodgerblue4", size = 0.8, weight = 0.3) +
  geom_smooth(aes(color = Gender), method='lm', se = F, alpha = 0.2, linetype = "longdash", size = 0.3, weight = 0.3) +
  stat_cor(method = "pearson", label.y = 31000, label.x = 70, color = "dodgerblue4") +
  ylab("Volume in mm3") + xlab("Age (in years)") +
  theme_minimal() +
  ggtitle("ABSOLUTE VOLUME T1") +
  theme(plot.title = element_text(hjust = 0.5))
Total.T1.Age.plot
ggsave("Total.T1.Age.plot.pdf", plot = Total.T1.Age.plot, width = 8, height = 6, units = "in", dpi = 600)  
 
 
   
 
 
  
Total.T2.Age.plot <-  ggplot(Prosencephalon.absolute, aes(y=T2, x = Age))  +
  geom_point(aes(color = Gender), size = 1.5, alpha = 1, shape = 16) +
  scale_color_manual(values = c("chartreuse4", "orangered2")) +
  geom_smooth(method='lm', alpha = 0.2, colour = "dodgerblue4", size = 0.8, weight = 0.3) +
  geom_smooth(aes(color = Gender), method='lm', se = F, alpha = 0.2, linetype = "longdash", size = 0.3, weight = 0.3) +
  stat_cor(method = "pearson", label.y = 29000, label.x = 70, color = "dodgerblue4") +
  ylab("Volume in mm3") + xlab("Age (in years)") +
  theme_minimal() +
  ggtitle("ABSOLUTE VOLUME T2") +
  theme(plot.title = element_text(hjust = 0.5))
Total.T2.Age.plot
ggsave("Total.T2.Age.plot.pdf", plot = Total.T2.Age.plot, width = 8, height = 6, units = "in", dpi = 600)  
 
 
   
 
 
  
Total.T3.Age.plot <-  ggplot(Prosencephalon.absolute, aes(y=T3, x = Age))  +
  geom_point(aes(color = Gender), size = 1.5, alpha = 1, shape = 16) +
  scale_color_manual(values = c("chartreuse4", "orangered2")) +
  geom_smooth(method='lm', alpha = 0.2, colour = "dodgerblue4", size = 0.8, weight = 0.3) +
  geom_smooth(aes(color = Gender), method='lm', se = F, alpha = 0.2, linetype = "longdash", size = 0.3, weight = 0.3) +
  stat_cor(method = "pearson", label.y = 6100, label.x = 70, color = "dodgerblue4") +
  ylab("Volume in mm3") + xlab("Age (in years)") +
  theme_minimal() +
  ggtitle("ABSOLUTE VOLUME T3") +
  theme(plot.title = element_text(hjust = 0.5))
Total.T3.Age.plot
ggsave("Total.T3.Age.plot.pdf", plot = Total.T3.Age.plot, width = 8, height = 6, units = "in", dpi = 600)  
 
 
   
 
 
  
Total.Planum.temporale.Age.plot <-  ggplot(Prosencephalon.absolute, aes(y=Planum.temporale, x = Age))  +
  geom_point(aes(color = Gender), size = 1.5, alpha = 1, shape = 16) +
  scale_color_manual(values = c("chartreuse4", "orangered2")) +
  geom_smooth(method='lm', alpha = 0.2, colour = "dodgerblue4", size = 0.8, weight = 0.3) +
  geom_smooth(aes(color = Gender), method='lm', se = F, alpha = 0.2, linetype = "longdash", size = 0.3, weight = 0.3) +
  stat_cor(method = "pearson", label.y = 4100, label.x = 70, color = "dodgerblue4") +
  ylab("Volume in mm3") + xlab("Age (in years)") +
  theme_minimal() +
  ggtitle("ABSOLUTE VOLUME planum temporale") +
  theme(plot.title = element_text(hjust = 0.5))
Total.Planum.temporale.Age.plot
ggsave("Total.Planum.temporale.Age.plot.pdf", plot = Total.Planum.temporale.Age.plot, width = 8, height = 6, units = "in", dpi = 600)  
 
 
   
 
 
  
Total.Planum.polare.Age.plot <-  ggplot(Prosencephalon.absolute, aes(y=Planum.polare, x = Age))  +
  geom_point(aes(color = Gender), size = 1.5, alpha = 1, shape = 16) +
  scale_color_manual(values = c("chartreuse4", "orangered2")) +
  geom_smooth(method='lm', alpha = 0.2, colour = "dodgerblue4", size = 0.8, weight = 0.3) +
  geom_smooth(aes(color = Gender), method='lm', se = F, alpha = 0.2, linetype = "longdash", size = 0.3, weight = 0.3) +
  stat_cor(method = "pearson", label.y = 13300, label.x = 70, color = "dodgerblue4") +
  ylab("Volume in mm3") + xlab("Age (in years)") +
  theme_minimal() +
  ggtitle("ABSOLUTE VOLUME planum polare") +
  theme(plot.title = element_text(hjust = 0.5))
Total.Planum.polare.Age.plot
ggsave("Total.Planum.polare.Age.plot.pdf", plot = Total.Planum.polare.Age.plot, width = 8, height = 6, units = "in", dpi = 600)  
 
 
   
 
 
  
Total.Short.insular.Age.plot <-  ggplot(Prosencephalon.absolute, aes(y=Short.insular, x = Age))  +
  geom_point(aes(color = Gender), size = 1.5, alpha = 1, shape = 16) +
  scale_color_manual(values = c("chartreuse4", "orangered2")) +
  geom_smooth(method='lm', alpha = 0.2, colour = "dodgerblue4", size = 0.8, weight = 0.3) +
  geom_smooth(aes(color = Gender), method='lm', se = F, alpha = 0.2, linetype = "longdash", size = 0.3, weight = 0.3) +
  stat_cor(method = "pearson", label.y = 19000, label.x = 70, color = "dodgerblue4") +
  ylab("Volume in mm3") + xlab("Age (in years)") +
  theme_minimal() +
  ggtitle("ABSOLUTE VOLUME short insular") +
  theme(plot.title = element_text(hjust = 0.5))
Total.Short.insular.Age.plot
ggsave("Total.Short.insular.Age.plot.pdf", plot = Total.Short.insular.Age.plot, width = 8, height = 6, units = "in", dpi = 600)  
 
 
   
 
 
  
Total.Long.insular.Age.plot <-  ggplot(Prosencephalon.absolute, aes(y=Long.insular, x = Age))  +
  geom_point(aes(color = Gender), size = 1.5, alpha = 1, shape = 16) +
  scale_color_manual(values = c("chartreuse4", "orangered2")) +
  geom_smooth(method='lm', alpha = 0.2, colour = "dodgerblue4", size = 0.8, weight = 0.3) +
  geom_smooth(aes(color = Gender), method='lm', se = F, alpha = 0.2, linetype = "longdash", size = 0.3, weight = 0.3) +
  stat_cor(method = "pearson", label.y = 12000, label.x = 70, color = "dodgerblue4") +
  ylab("Volume in mm3") + xlab("Age (in years)") +
  theme_minimal() +
  ggtitle("ABSOLUTE VOLUME long insular") +
  theme(plot.title = element_text(hjust = 0.5))
Total.Long.insular.Age.plot
ggsave("Total.Long.insular.Age.plot.pdf", plot = Total.Long.insular.Age.plot, width = 8, height = 6, units = "in", dpi = 600)  
 
 
   
 
 
  
Total.SCA.Age.plot <-  ggplot(Prosencephalon.absolute, aes(y=SCA, x = Age))  +
  geom_point(aes(color = Gender), size = 1.5, alpha = 1, shape = 16) +
  scale_color_manual(values = c("chartreuse4", "orangered2")) +
  geom_smooth(method='lm', alpha = 0.2, colour = "dodgerblue4", size = 0.8, weight = 0.3) +
  geom_smooth(aes(color = Gender), method='lm', se = F, alpha = 0.2, linetype = "longdash", size = 0.3, weight = 0.3) +
  stat_cor(method = "pearson", label.y = 3800, label.x = 70, color = "dodgerblue4") +
  ylab("Volume in mm3") + xlab("Age (in years)") +
  theme_minimal() +
  ggtitle("ABSOLUTE VOLUME subcallosal area") +
  theme(plot.title = element_text(hjust = 0.5))
Total.SCA.Age.plot
ggsave("Total.SCA.Age.plot.pdf", plot = Total.SCA.Age.plot, width = 8, height = 6, units = "in", dpi = 600)  
 
 
   
 
 
  
Total.Cingulate.anterior.Age.plot <-  ggplot(Prosencephalon.absolute, aes(y=Cingulate.anterior, x = Age))  +
  geom_point(aes(color = Gender), size = 1.5, alpha = 1, shape = 16) +
  scale_color_manual(values = c("chartreuse4", "orangered2")) +
  geom_smooth(method='lm', alpha = 0.2, colour = "dodgerblue4", size = 0.8, weight = 0.3) +
  geom_smooth(aes(color = Gender), method='lm', se = F, alpha = 0.2, linetype = "longdash", size = 0.3, weight = 0.3) +
  stat_cor(method = "pearson", label.y = 17300, label.x = 70, color = "dodgerblue4") +
  ylab("Volume in mm3") + xlab("Age (in years)") +
  theme_minimal() +
  ggtitle("ABSOLUTE VOLUME cingulate anterior") +
  theme(plot.title = element_text(hjust = 0.5))
Total.Cingulate.anterior.Age.plot
ggsave("Total.Cingulate.anterior.Age.plot.pdf", plot = Total.Cingulate.anterior.Age.plot, width = 8, height = 6, units = "in", dpi = 600)  
 
 
   
 
 
  
Total.Cingulate.middle.Age.plot <-  ggplot(Prosencephalon.absolute, aes(y=Cingulate.middle, x = Age))  +
  geom_point(aes(color = Gender), size = 1.5, alpha = 1, shape = 16) +
  scale_color_manual(values = c("chartreuse4", "orangered2")) +
  geom_smooth(method='lm', alpha = 0.2, colour = "dodgerblue4", size = 0.8, weight = 0.3) +
  geom_smooth(aes(color = Gender), method='lm', se = F, alpha = 0.2, linetype = "longdash", size = 0.3, weight = 0.3) +
  stat_cor(method = "pearson", label.y = 20300, label.x = 70, color = "dodgerblue4") +
  ylab("Volume in mm3") + xlab("Age (in years)") +
  theme_minimal() +
  ggtitle("ABSOLUTE VOLUME cingulate middle") +
  theme(plot.title = element_text(hjust = 0.5))
Total.Cingulate.middle.Age.plot
ggsave("Total.Cingulate.middle.Age.plot.pdf", plot = Total.Cingulate.middle.Age.plot, width = 8, height = 6, units = "in", dpi = 600)  
 
 
   
 
 
  
Total.Cingulate.posterior.Age.plot <-  ggplot(Prosencephalon.absolute, aes(y=Cingulate.posterior, x = Age))  +
  geom_point(aes(color = Gender), size = 1.5, alpha = 1, shape = 16) +
  scale_color_manual(values = c("chartreuse4", "orangered2")) +
  geom_smooth(method='lm', alpha = 0.2, colour = "dodgerblue4", size = 0.8, weight = 0.3) +
  geom_smooth(aes(color = Gender), method='lm', se = F, alpha = 0.2, linetype = "longdash", size = 0.3, weight = 0.3) +
  stat_cor(method = "pearson", label.y = 24300, label.x = 70, color = "dodgerblue4") +
  ylab("Volume in mm3") + xlab("Age (in years)") +
  theme_minimal() +
  ggtitle("ABSOLUTE VOLUME cingulate posterior") +
  theme(plot.title = element_text(hjust = 0.5))
Total.Cingulate.posterior.Age.plot
ggsave("Total.Cingulate.posterior.Age.plot.pdf", plot = Total.Cingulate.posterior.Age.plot, width = 8, height = 6, units = "in", dpi = 600)  
 
 
   
 
 
  
Total.PHG.Age.plot <-  ggplot(Prosencephalon.absolute, aes(y=PHG, x = Age))  +
  geom_point(aes(color = Gender), size = 1.5, alpha = 1, shape = 16) +
  scale_color_manual(values = c("chartreuse4", "orangered2")) +
  geom_smooth(method='lm', alpha = 0.2, colour = "dodgerblue4", size = 0.8, weight = 0.3) +
  geom_smooth(aes(color = Gender), method='lm', se = F, alpha = 0.2, linetype = "longdash", size = 0.3, weight = 0.3) +
  stat_cor(method = "pearson", label.y = 14000, label.x = 70, color = "dodgerblue4") +
  ylab("Volume in mm3") + xlab("Age (in years)") +
  theme_minimal() +
  ggtitle("ABSOLUTE VOLUME PHG") +
  theme(plot.title = element_text(hjust = 0.5))
Total.PHG.Age.plot
ggsave("Total.PHG.Age.plot.pdf", plot = Total.PHG.Age.plot, width = 8, height = 6, units = "in", dpi = 600)  
 
 
   
 
 
  
Total.Hippocampus.Age.plot <-  ggplot(Prosencephalon.absolute, aes(y=Hippocampus, x = Age))  +
  geom_point(aes(color = Gender), size = 1.5, alpha = 1, shape = 16) +
  scale_color_manual(values = c("chartreuse4", "orangered2")) +
  geom_smooth(method='lm', alpha = 0.2, colour = "dodgerblue4", size = 0.8, weight = 0.3) +
  geom_smooth(aes(color = Gender), method='lm', se = F, alpha = 0.2, linetype = "longdash", size = 0.3, weight = 0.3) +
  stat_cor(method = "pearson", label.y = 9700, label.x = 70, color = "dodgerblue4") +
  ylab("Volume in mm3") + xlab("Age (in years)") +
  theme_minimal() +
  ggtitle("ABSOLUTE VOLUME hippocampus") +
  theme(plot.title = element_text(hjust = 0.5))
Total.Hippocampus.Age.plot
ggsave("Total.Hippocampus.Age.plot.pdf", plot = Total.Hippocampus.Age.plot, width = 8, height = 6, units = "in", dpi = 600)  
 
 
   
 
 
  
Total.Amygdala.Age.plot <-  ggplot(Prosencephalon.absolute, aes(y=Amygdala, x = Age))  +
  geom_point(aes(color = Gender), size = 1.5, alpha = 1, shape = 16) +
  scale_color_manual(values = c("chartreuse4", "orangered2")) +
  geom_smooth(method='lm', alpha = 0.2, colour = "dodgerblue4", size = 0.8, weight = 0.3) +
  geom_smooth(aes(color = Gender), method='lm', se = F, alpha = 0.2, linetype = "longdash", size = 0.3, weight = 0.3) +
  stat_cor(method = "pearson", label.y = 3600, label.x = 60, color = "dodgerblue4") +
  ylab("Volume in mm3") + xlab("Age (in years)") +
  theme_minimal() +
  ggtitle("ABSOLUTE VOLUME amygdala") +
  theme(plot.title = element_text(hjust = 0.5))
Total.Amygdala.Age.plot
ggsave("Total.Amygdala.Age.plot.pdf", plot = Total.Amygdala.Age.plot, width = 8, height = 6, units = "in", dpi = 600)  
 
 
   
 
 
  
Total.Corpus.callosum.Age.plot <-  ggplot(Prosencephalon.absolute, aes(y=Corpus.callosum, x = Age))  +
  geom_point(aes(color = Gender), size = 1.5, alpha = 1, shape = 16) +
  scale_color_manual(values = c("chartreuse4", "orangered2")) +
  geom_smooth(method='lm', alpha = 0.2, colour = "dodgerblue4", size = 0.8, weight = 0.3) +
  geom_smooth(aes(color = Gender), method='lm', se = F, alpha = 0.2, linetype = "longdash", size = 0.3, weight = 0.3) +
  stat_cor(method = "pearson", label.y = 4100, label.x = 70, color = "dodgerblue4") +
  ylab("Volume in mm3") + xlab("Age (in years)") +
  theme_minimal() +
  ggtitle("ABSOLUTE VOLUME corpus callosum") +
  theme(plot.title = element_text(hjust = 0.5))
Total.Corpus.callosum.Age.plot
ggsave("Total.Corpus.callosum.Age.plot.pdf", plot = Total.Corpus.callosum.Age.plot, width = 8, height = 6, units = "in", dpi = 600)  
 
 
   
 
 
  
Total.Claustrum.Age.plot <-  ggplot(Prosencephalon.absolute, aes(y=Claustrum, x = Age))  +
  geom_point(aes(color = Gender), size = 1.5, alpha = 1, shape = 16) +
  scale_color_manual(values = c("chartreuse4", "orangered2")) +
  geom_smooth(method='lm', alpha = 0.2, colour = "dodgerblue4", size = 0.8, weight = 0.3) +
  geom_smooth(aes(color = Gender), method='lm', se = F, alpha = 0.2, linetype = "longdash", size = 0.3, weight = 0.3) +
  stat_cor(method = "pearson", label.y = 2100, label.x = 70, color = "dodgerblue4") +
  ylab("Volume in mm3") + xlab("Age (in years)") +
  theme_minimal() +
  ggtitle("ABSOLUTE VOLUME claustrum") +
  theme(plot.title = element_text(hjust = 0.5))
Total.Claustrum.Age.plot
ggsave("Total.Claustrum.Age.plot.pdf", plot = Total.Claustrum.Age.plot, width = 8, height = 6, units = "in", dpi = 600)  
 
 
   
 
 
  
Total.Putamen.Age.plot <-  ggplot(Prosencephalon.absolute, aes(y=Putamen, x = Age))  +
  geom_point(aes(color = Gender), size = 1.5, alpha = 1, shape = 16) +
  scale_color_manual(values = c("chartreuse4", "orangered2")) +
  geom_smooth(method='lm', alpha = 0.2, colour = "dodgerblue4", size = 0.8, weight = 0.3) +
  geom_smooth(aes(color = Gender), method='lm', se = F, alpha = 0.2, linetype = "longdash", size = 0.3, weight = 0.3) +
  stat_cor(method = "pearson", label.y = 12300, label.x = 70, color = "dodgerblue4") +
  ylab("Volume in mm3") + xlab("Age (in years)") +
  theme_minimal() +
  ggtitle("ABSOLUTE VOLUME putamen") +
  theme(plot.title = element_text(hjust = 0.5))
Total.Putamen.Age.plot
ggsave("Total.Putamen.Age.plot.pdf", plot = Total.Putamen.Age.plot, width = 8, height = 6, units = "in", dpi = 600)  
 
 
   
 
 
  
Total.Caudate.Age.plot <-  ggplot(Prosencephalon.absolute, aes(y=Caudate, x = Age))  +
  geom_point(aes(color = Gender), size = 1.5, alpha = 1, shape = 16) +
  scale_color_manual(values = c("chartreuse4", "orangered2")) +
  geom_smooth(method='lm', alpha = 0.2, colour = "dodgerblue4", size = 0.8, weight = 0.3) +
  geom_smooth(aes(color = Gender), method='lm', se = F, alpha = 0.2, linetype = "longdash", size = 0.3, weight = 0.3) +
  stat_cor(method = "pearson", label.y = 10200, label.x = 70, color = "dodgerblue4") +
  ylab("Volume in mm3") + xlab("Age (in years)") +
  theme_minimal() +
  ggtitle("ABSOLUTE VOLUME caudate") +
  theme(plot.title = element_text(hjust = 0.5))
Total.Caudate.Age.plot
ggsave("Total.Caudate.Age.plot.pdf", plot = Total.Caudate.Age.plot, width = 8, height = 6, units = "in", dpi = 600)  
 
 
   
 
 
  
Total.Globus.pallidum.Age.plot <-  ggplot(Prosencephalon.absolute, aes(y=Globus.pallidum, x = Age))  +
  geom_point(aes(color = Gender), size = 1.5, alpha = 1, shape = 16) +
  scale_color_manual(values = c("chartreuse4", "orangered2")) +
  geom_smooth(method='lm', alpha = 0.2, colour = "dodgerblue4", size = 0.8, weight = 0.3) +
  geom_smooth(aes(color = Gender), method='lm', se = F, alpha = 0.2, linetype = "longdash", size = 0.3, weight = 0.3) +
  stat_cor(method = "pearson", label.y = 4200, label.x = 70, color = "dodgerblue4") +
  ylab("Volume in mm3") + xlab("Age (in years)") +
  theme_minimal() +
  ggtitle("ABSOLUTE VOLUME globus pallidum") +
  theme(plot.title = element_text(hjust = 0.5))
Total.Globus.pallidum.Age.plot
ggsave("Total.Globus.pallidum.Age.plot.pdf", plot = Total.Globus.pallidum.Age.plot, width = 8, height = 6, units = "in", dpi = 600)  
 
 
   
 
 
  
Total.Internal.capsule.Age.plot <-  ggplot(Prosencephalon.absolute, aes(y=Internal.capsule, x = Age))  +
  geom_point(aes(color = Gender), size = 1.5, alpha = 1, shape = 16) +
  scale_color_manual(values = c("chartreuse4", "orangered2")) +
  geom_smooth(method='lm', alpha = 0.2, colour = "dodgerblue4", size = 0.8, weight = 0.3) +
  geom_smooth(aes(color = Gender), method='lm', se = F, alpha = 0.2, linetype = "longdash", size = 0.3, weight = 0.3) +
  stat_cor(method = "pearson", label.y = 12200, label.x = 70, color = "dodgerblue4") +
  ylab("Volume in mm3") + xlab("Age (in years)") +
  theme_minimal() +
  ggtitle("ABSOLUTE VOLUME internal capsule") +
  theme(plot.title = element_text(hjust = 0.5))
Total.Internal.capsule.Age.plot
ggsave("Total.Internal.capsule.Age.plot.pdf", plot = Total.Internal.capsule.Age.plot, width = 8, height = 6, units = "in", dpi = 600)  
 
 
   
 
 
  
Total.Innominate.substance.Age.plot <-  ggplot(Prosencephalon.absolute, aes(y=Innominate.substance, x = Age))  +
  geom_point(aes(color = Gender), size = 1.5, alpha = 1, shape = 16) +
  scale_color_manual(values = c("chartreuse4", "orangered2")) +
  geom_smooth(method='lm', alpha = 0.2, colour = "dodgerblue4", size = 0.8, weight = 0.3) +
  geom_smooth(aes(color = Gender), method='lm', se = F, alpha = 0.2, linetype = "longdash", size = 0.3, weight = 0.3) +
  stat_cor(method = "pearson", label.y = 3180, label.x = 70, color = "dodgerblue4") +
  ylab("Volume in mm3") + xlab("Age (in years)") +
  theme_minimal() +
  ggtitle("ABSOLUTE VOLUME innominate substance") +
  theme(plot.title = element_text(hjust = 0.5))
Total.Innominate.substance.Age.plot
ggsave("Total.Innominate.substance.Age.plot.pdf", plot = Total.Innominate.substance.Age.plot, width = 8, height = 6, units = "in", dpi = 600)  
 
 
   
 
 
  
Total.Hypothalamus.Age.plot <-  ggplot(Prosencephalon.absolute, aes(y=Hypothalamus, x = Age))  +
  geom_point(aes(color = Gender), size = 1.5, alpha = 1, shape = 16) +
  scale_color_manual(values = c("chartreuse4", "orangered2")) +
  geom_smooth(method='lm', alpha = 0.2, colour = "dodgerblue4", size = 0.8, weight = 0.3) +
  geom_smooth(aes(color = Gender), method='lm', se = F, alpha = 0.2, linetype = "longdash", size = 0.3, weight = 0.3) +
  stat_cor(method = "pearson", label.y = 9200, label.x = 70, color = "dodgerblue4") +
  ylab("Volume in mm3") + xlab("Age (in years)") +
  theme_minimal() +
  ggtitle("ABSOLUTE VOLUME hypothalamus") +
  theme(plot.title = element_text(hjust = 0.5))
Total.Hypothalamus.Age.plot
ggsave("Total.Hypothalamus.Age.plot.pdf", plot = Total.Hypothalamus.Age.plot, width = 8, height = 6, units = "in", dpi = 600)  
 
 
   
 
 
  
Total.Thalamus.Age.plot <-  ggplot(Prosencephalon.absolute, aes(y=Thalamus, x = Age))  +
  geom_point(aes(color = Gender), size = 1.5, alpha = 1, shape = 16) +
  scale_color_manual(values = c("chartreuse4", "orangered2")) +
  geom_smooth(method='lm', alpha = 0.2, colour = "dodgerblue4", size = 0.8, weight = 0.3) +
  geom_smooth(aes(color = Gender), method='lm', se = F, alpha = 0.2, linetype = "longdash", size = 0.3, weight = 0.3) +
  stat_cor(method = "pearson", label.y = 17000, label.x = 70, color = "dodgerblue4") +
  ylab("Volume in mm3") + xlab("Age (in years)") +
  theme_minimal() +
  ggtitle("ABSOLUTE VOLUME thalamus") +
  theme(plot.title = element_text(hjust = 0.5))
Total.Thalamus.Age.plot
ggsave("Total.Thalamus.Age.plot.pdf", plot = Total.Thalamus.Age.plot, width = 8, height = 6, units = "in", dpi = 600)
  
 
 
   
 
 
 
 
 
 Relative Volumes 
 
 
 
  
#Prosencephalon.absolute <- select(Prosencephalon.absolute, - c(Gender))
Prosencephalon.relative <- (100 * (Prosencephalon.absolute/All.Volumes$`Total encephalic volume (without ventricles)`))  
 
 
  ‘/’ not meaningful for factors  
 
 
  Prosencephalon.relative1 <- Prosencephalon.relative[,-c(41:51)]
Prosencephalon.relative2 <- Prosencephalon.relative[, c(41:51)]

Table.Prosencephalon.relative <- CreateTableOne(
  vars = c("Frontal.pole",
  "F1",
  "F2",
  "F3.orbital", 
  "F3.triangular", 
  "F3.opercular", 
  "Anterior.orbital", 
  "Medial.orbital", 
  "Lateral.orbital", 
  "Posterior.orbital",
  "Rectus", 
  "Rostral", 
  "Precentral", 
  "Postcentral", 
  "Paracentral.lobule", 
  "Subcentral", 
  "SPL", 
  "SMG", 
  "ANG", 
  "Precuneus",
  "Cuneus", 
  "O1",
  "O2", 
  "O3", 
  "Occipital.pole", 
  "Lingual", 
  "Fusiform", 
  "Temporal.pole", 
  "T1",
  "T2", 
  "T3", 
  "Planum.temporale", 
  "Planum.polare", 
  "Short.insular", 
  "Long.insular", 
  "SCA", 
  "Cingulate.anterior", 
  "Cingulate.middle", 
  "Cingulate.posterior", 
  "PHG", 
  "Hippocampus", 
  "Amygdala", 
  "Corpus.callosum", 
  "Claustrum", 
  "Putamen", 
  "Caudate", 
  "Globus.pallidum", 
  "Internal.capsule",
  "Innominate.substance", 
  "Hypothalamus",
  "Thalamus"),
  data = Prosencephalon.relative)

Prosencephalon.relative$Gender <- All.Volumes$Gender
Table.Prosencephalon.relative.stratified.gender <- CreateTableOne(
  vars = c("Frontal.pole",
  "F1",
  "F2",
  "F3.orbital", 
  "F3.triangular", 
  "F3.opercular", 
  "Anterior.orbital", 
  "Medial.orbital", 
  "Lateral.orbital", 
  "Posterior.orbital",
  "Rectus", 
  "Rostral", 
  "Precentral", 
  "Postcentral", 
  "Paracentral.lobule", 
  "Subcentral", 
  "SPL", 
  "SMG", 
  "ANG", 
  "Precuneus",
  "Cuneus", 
  "O1",
  "O2", 
  "O3", 
  "Occipital.pole", 
  "Lingual", 
  "Fusiform", 
  "Temporal.pole", 
  "T1",
  "T2", 
  "T3", 
  "Planum.temporale", 
  "Planum.polare", 
  "Short.insular", 
  "Long.insular", 
  "SCA", 
  "Cingulate.anterior", 
  "Cingulate.middle", 
  "Cingulate.posterior", 
  "PHG", 
  "Hippocampus", 
  "Amygdala", 
  "Corpus.callosum", 
  "Claustrum", 
  "Putamen", 
  "Caudate", 
  "Globus.pallidum", 
  "Internal.capsule",
  "Innominate.substance", 
  "Hypothalamus",
  "Thalamus"),
  strata = c("Gender"),
  data = Prosencephalon.relative)

Table.Prosencephalon.relative <- print(Table.Prosencephalon.relative, contDigits = 10)  
 
 
                                    
                                   Overall                    
  n                                          30               
  Frontal.pole (mean (SD))         0.4308161280 (0.0735790922)
  F1 (mean (SD))                   6.2797984283 (0.3863873818)
  F2 (mean (SD))                   5.3376497465 (0.3212511986)
  F3.orbital (mean (SD))           0.3580350033 (0.0400757488)
  F3.triangular (mean (SD))        1.0712810271 (0.1454267486)
  F3.opercular (mean (SD))         1.2780793248 (0.1499873117)
  Anterior.orbital (mean (SD))     0.3058239406 (0.0373903676)
  Medial.orbital (mean (SD))       0.6908155982 (0.0847676556)
  Lateral.orbital (mean (SD))      0.5536005086 (0.0684027573)
  Posterior.orbital (mean (SD))    0.6775656621 (0.0832782152)
  Rectus (mean (SD))               0.9134375442 (0.4012168421)
  Rostral (mean (SD))              0.3293498153 (0.0250770666)
  Precentral (mean (SD))           3.5870207286 (0.3013356393)
  Postcentral (mean (SD))          2.0375742862 (0.1796109149)
  Paracentral.lobule (mean (SD))   1.2872489540 (0.1404521846)
  Subcentral (mean (SD))           0.5533885997 (0.0701240033)
  SPL (mean (SD))                  3.0512299837 (0.2995764614)
  SMG (mean (SD))                  2.8054702345 (0.2963151197)
  ANG (mean (SD))                  3.1401508432 (0.2974069556)
  Precuneus (mean (SD))            2.8184789289 (0.2195354334)
  Cuneus (mean (SD))               0.9218075932 (0.1105395289)
  O1 (mean (SD))                   0.8363347713 (0.0794530354)
  O2 (mean (SD))                   1.4412036853 (0.1395067116)
  O3 (mean (SD))                   0.8459452532 (0.1135967778)
  Occipital.pole (mean (SD))       1.0401371468 (0.0957630116)
  Lingual (mean (SD))              1.8611729124 (0.2307977883)
  Fusiform (mean (SD))             2.0313915705 (0.2297491283)
  Temporal.pole (mean (SD))        2.3163307043 (0.1969989384)
  T1 (mean (SD))                   2.4899711225 (0.2314929390)
  T2 (mean (SD))                   2.1710340083 (0.2222231589)
  T3 (mean (SD))                   0.4573538448 (0.0569894934)
  Planum.temporale (mean (SD))     0.2805395089 (0.0570436553)
  Planum.polare (mean (SD))        0.9110250954 (0.1711155137)
  Short.insular (mean (SD))        1.4149000524 (0.1765065390)
  Long.insular (mean (SD))         0.8324538909 (0.1196168331)
  SCA (mean (SD))                  0.2170797247 (0.0471724812)
  Cingulate.anterior (mean (SD))   1.3176948779 (0.1348973794)
  Cingulate.middle (mean (SD))     1.5637867547 (0.1479091119)
  Cingulate.posterior (mean (SD))  1.8413310529 (0.1454271384)
  PHG (mean (SD))                  0.9563118242 (0.1171393527)
  Hippocampus (mean (SD))          0.7446874971 (0.0621830878)
  Amygdala (mean (SD))             0.2864526632 (0.0401036938)
  Corpus.callosum (mean (SD))      0.3039193139 (0.0468123806)
  Claustrum (mean (SD))            0.1228498024 (0.0358816349)
  Putamen (mean (SD))              1.0344949954 (0.1388220008)
  Caudate (mean (SD))              0.7138494977 (0.1087637962)
  Globus.pallidum (mean (SD))      0.2805060774 (0.0391642507)
  Internal.capsule (mean (SD))     0.9724042811 (0.1172447094)
  Innominate.substance (mean (SD)) 0.2477217623 (0.0361218477)
  Hypothalamus (mean (SD))         0.7289368930 (0.0482037353)
  Thalamus (mean (SD))             1.3400717256 (0.1017134094)  
 
 
  Table.Prosencephalon.relative.stratified.gender <- print(Table.Prosencephalon.relative.stratified.gender, contDigits = 10)  
 
 
                                    Stratified by Gender
                                   f                           m                           p      test
  n                                          14                          16                           
  Frontal.pole (mean (SD))         0.4282337280 (0.0713377035) 0.4330757280 (0.0777473097)  0.861     
  F1 (mean (SD))                   6.3006329062 (0.3639376668) 6.2615682601 (0.4160375339)  0.788     
  F2 (mean (SD))                   5.3883848936 (0.3077446428) 5.2932564928 (0.3360665361)  0.428     
  F3.orbital (mean (SD))           0.3571578969 (0.0385677622) 0.3588024714 (0.0425977851)  0.913     
  F3.triangular (mean (SD))        1.0636322212 (0.1197364194) 1.0779737323 (0.1684052590)  0.793     
  F3.opercular (mean (SD))         1.2495682579 (0.1574229154) 1.3030265083 (0.1435004821)  0.339     
  Anterior.orbital (mean (SD))     0.3017582832 (0.0164770413) 0.3093813908 (0.0493827496)  0.586     
  Medial.orbital (mean (SD))       0.6815918090 (0.0371181605) 0.6988864137 (0.1120228996)  0.586     
  Lateral.orbital (mean (SD))      0.5464232617 (0.0304519052) 0.5598805996 (0.0902891005)  0.600     
  Posterior.orbital (mean (SD))    0.6691168234 (0.0367863500) 0.6849583960 (0.1100474777)  0.612     
  Rectus (mean (SD))               0.8532393638 (0.0662940918) 0.9661109520 (0.5486963089)  0.452     
  Rostral (mean (SD))              0.3339400566 (0.0264801278) 0.3253333540 (0.0239001532)  0.357     
  Precentral (mean (SD))           3.6104693496 (0.2468897213) 3.5665031852 (0.3489460002)  0.697     
  Postcentral (mean (SD))          2.0354425348 (0.1884559911) 2.0394395687 (0.1777112761)  0.953     
  Paracentral.lobule (mean (SD))   1.3045162856 (0.1438324556) 1.2721400388 (0.1403115272)  0.538     
  Subcentral (mean (SD))           0.5355965755 (0.0569946874) 0.5689566208 (0.0783433358)  0.199     
  SPL (mean (SD))                  3.0950976636 (0.2771645426) 3.0128457637 (0.3218134493)  0.463     
  SMG (mean (SD))                  2.7870612121 (0.2560750682) 2.8215781290 (0.3351530207)  0.756     
  ANG (mean (SD))                  3.1074492113 (0.3335758333) 3.1687647712 (0.2696241186)  0.582     
  Precuneus (mean (SD))            2.7769055892 (0.2074598065) 2.8548556011 (0.2298977990)  0.341     
  Cuneus (mean (SD))               0.9162725007 (0.1085944316) 0.9266507992 (0.1155395028)  0.803     
  O1 (mean (SD))                   0.8107747580 (0.0725777711) 0.8586997830 (0.0805991452)  0.100     
  O2 (mean (SD))                   1.4338278528 (0.1550060300) 1.4476575387 (0.1292605063)  0.792     
  O3 (mean (SD))                   0.8091809468 (0.1170210398) 0.8781140214 (0.1035122305)  0.098     
  Occipital.pole (mean (SD))       1.0453411723 (0.1129383712) 1.0355836245 (0.0814121997)  0.786     
  Lingual (mean (SD))              1.9260643720 (0.2287149262) 1.8043928852 (0.2242302480)  0.153     
  Fusiform (mean (SD))             2.0431163714 (0.2142611581) 2.0211323697 (0.2490441211)  0.799     
  Temporal.pole (mean (SD))        2.2887424248 (0.1717974683) 2.3404704489 (0.2193601055)  0.483     
  T1 (mean (SD))                   2.4388032912 (0.1640313381) 2.5347429750 (0.2751451068)  0.265     
  T2 (mean (SD))                   2.2309720524 (0.1865495819) 2.1185882198 (0.2429535202)  0.171     
  T3 (mean (SD))                   0.4422376645 (0.0597357531) 0.4705805025 (0.0527885654)  0.179     
  Planum.temporale (mean (SD))     0.2652397597 (0.0370145950) 0.2939267894 (0.0685126147)  0.174     
  Planum.polare (mean (SD))        0.9612340750 (0.1660219988) 0.8670922381 (0.1682532577)  0.135     
  Short.insular (mean (SD))        1.4338625682 (0.1204101258) 1.3983078510 (0.2168811526)  0.591     
  Long.insular (mean (SD))         0.7888303012 (0.0888448680) 0.8706245319 (0.1322545191)  0.060     
  SCA (mean (SD))                  0.2044625144 (0.0441130883) 0.2281197836 (0.0483429862)  0.175     
  Cingulate.anterior (mean (SD))   1.3135641868 (0.1439613708) 1.3213092326 (0.1311107677)  0.879     
  Cingulate.middle (mean (SD))     1.5619775543 (0.1253223665) 1.5653698051 (0.1693469993)  0.951     
  Cingulate.posterior (mean (SD))  1.8380656367 (0.1343469675) 1.8441882921 (0.1588299348)  0.911     
  PHG (mean (SD))                  0.9762747018 (0.0728687435) 0.9388443063 (0.1457024489)  0.392     
  Hippocampus (mean (SD))          0.7655342036 (0.0635206138) 0.7264466289 (0.0567298899)  0.086     
  Amygdala (mean (SD))             0.2948215970 (0.0466054982) 0.2791298460 (0.0332319267)  0.293     
  Corpus.callosum (mean (SD))      0.3231162484 (0.0427646174) 0.2871219961 (0.0447975428)  0.033     
  Claustrum (mean (SD))            0.1157108693 (0.0257804133) 0.1290963689 (0.0427077348)  0.316     
  Putamen (mean (SD))              1.0552049804 (0.1037510009) 1.0163737585 (0.1648596697)  0.454     
  Caudate (mean (SD))              0.7197152028 (0.0745225273) 0.7087170058 (0.1341534632)  0.788     
  Globus.pallidum (mean (SD))      0.2799215892 (0.0302657494) 0.2810175045 (0.0465933809)  0.941     
  Internal.capsule (mean (SD))     0.9899260573 (0.1268862057) 0.9570727269 (0.1099341721)  0.454     
  Innominate.substance (mean (SD)) 0.2587625040 (0.0391948981) 0.2380611133 (0.0312707598)  0.119     
  Hypothalamus (mean (SD))         0.7337200336 (0.0472119392) 0.7247516451 (0.0502044289)  0.620     
  Thalamus (mean (SD))             1.3867798315 (0.0776479089) 1.2992021329 (0.1046820913)  0.016       
 
 
 
 
 
 
  
Table.Prosencephalon.relative.RSD <- as.data.frame(Table.Prosencephalon.relative)
Table.Prosencephalon.relative.RSD <- data.frame(do.call('rbind', 
                                                              strsplit(as.character(Table.Prosencephalon.relative.RSD[-1,]),
                                                                       ' (',fixed=TRUE)))
Table.Prosencephalon.relative.RSD <- data.frame(cbind(str_replace_all(Table.Prosencephalon.relative.RSD$X1, "[ ]", ""),
                                                            str_replace_all(Table.Prosencephalon.relative.RSD$X2, "[)]", "")))
Table.Prosencephalon.relative.RSD$X1 <- as.character(Table.Prosencephalon.relative.RSD$X1)
Table.Prosencephalon.relative.RSD$X2 <- as.character(Table.Prosencephalon.relative.RSD$X2)
Table.Prosencephalon.relative.RSD <- as.data.frame(sapply(Table.Prosencephalon.relative.RSD, as.numeric))
Table.Prosencephalon.relative.RSD <- as.data.frame(Table.Prosencephalon.relative.RSD$X2/Table.Prosencephalon.relative.RSD$X1)
Table.Prosencephalon.relative.RSD <- round(Table.Prosencephalon.relative.RSD * 100, 1)


Table.Prosencephalon.relative.stratified.gender.RSD <- as.data.frame(Table.Prosencephalon.relative.stratified.gender)
Table.Prosencephalon.relative.stratified.gender.RSD <- select(Table.Prosencephalon.relative.stratified.gender.RSD, - c(p, test))

Table.Prosencephalon.relative.stratified.gender.RSD.female <- data.frame(do.call('rbind', 
                                                              strsplit(as.character(Table.Prosencephalon.relative.stratified.gender.RSD[-1, "f"]),
                                                                       ' (',fixed=TRUE)))
Table.Prosencephalon.relative.stratified.gender.RSD.female <- data.frame(cbind(str_replace_all(Table.Prosencephalon.relative.stratified.gender.RSD.female$X1, "[ ]", ""),
                                                            str_replace_all(Table.Prosencephalon.relative.stratified.gender.RSD.female$X2, "[)]", "")))
Table.Prosencephalon.relative.stratified.gender.RSD.female$X1 <- as.character(Table.Prosencephalon.relative.stratified.gender.RSD.female$X1)
Table.Prosencephalon.relative.stratified.gender.RSD.female$X2 <- as.character(Table.Prosencephalon.relative.stratified.gender.RSD.female$X2)
Table.Prosencephalon.relative.stratified.gender.RSD.female <- as.data.frame(sapply(Table.Prosencephalon.relative.stratified.gender.RSD.female, as.numeric))
Table.Prosencephalon.relative.stratified.gender.RSD.female <- as.data.frame(Table.Prosencephalon.relative.stratified.gender.RSD.female$X2/Table.Prosencephalon.relative.stratified.gender.RSD.female$X1)
Table.Prosencephalon.relative.stratified.gender.RSD.female <- round(Table.Prosencephalon.relative.stratified.gender.RSD.female * 100, 1)

Table.Prosencephalon.relative.stratified.gender.RSD.male <- data.frame(do.call('rbind', 
                                                              strsplit(as.character(Table.Prosencephalon.relative.stratified.gender.RSD[-1, "m"]),
                                                                       ' (',fixed=TRUE)))
Table.Prosencephalon.relative.stratified.gender.RSD.male <- data.frame(cbind(str_replace_all(Table.Prosencephalon.relative.stratified.gender.RSD.male$X1, "[ ]", ""),
                                                            str_replace_all(Table.Prosencephalon.relative.stratified.gender.RSD.male$X2, "[)]", "")))
Table.Prosencephalon.relative.stratified.gender.RSD.male$X1 <- as.character(Table.Prosencephalon.relative.stratified.gender.RSD.male$X1)
Table.Prosencephalon.relative.stratified.gender.RSD.male$X2 <- as.character(Table.Prosencephalon.relative.stratified.gender.RSD.male$X2)
Table.Prosencephalon.relative.stratified.gender.RSD.male <- as.data.frame(sapply(Table.Prosencephalon.relative.stratified.gender.RSD.male, as.numeric))
Table.Prosencephalon.relative.stratified.gender.RSD.male <- as.data.frame(Table.Prosencephalon.relative.stratified.gender.RSD.male$X2/Table.Prosencephalon.relative.stratified.gender.RSD.male$X1)
Table.Prosencephalon.relative.stratified.gender.RSD.male <- round(Table.Prosencephalon.relative.stratified.gender.RSD.male * 100, 1)
  
 
 
 
 
 
 
  
kable(Table.Prosencephalon.relative)  
 
 
 

 
 
 
  
 Overall 
 
 
 
 
 n 
 30 
 
 
 Frontal.pole (mean (SD)) 
 0.4308161280 (0.0735790922) 
 
 
 F1 (mean (SD)) 
 6.2797984283 (0.3863873818) 
 
 
 F2 (mean (SD)) 
 5.3376497465 (0.3212511986) 
 
 
 F3.orbital (mean (SD)) 
 0.3580350033 (0.0400757488) 
 
 
 F3.triangular (mean (SD)) 
 1.0712810271 (0.1454267486) 
 
 
 F3.opercular (mean (SD)) 
 1.2780793248 (0.1499873117) 
 
 
 Anterior.orbital (mean (SD)) 
 0.3058239406 (0.0373903676) 
 
 
 Medial.orbital (mean (SD)) 
 0.6908155982 (0.0847676556) 
 
 
 Lateral.orbital (mean (SD)) 
 0.5536005086 (0.0684027573) 
 
 
 Posterior.orbital (mean (SD)) 
 0.6775656621 (0.0832782152) 
 
 
 Rectus (mean (SD)) 
 0.9134375442 (0.4012168421) 
 
 
 Rostral (mean (SD)) 
 0.3293498153 (0.0250770666) 
 
 
 Precentral (mean (SD)) 
 3.5870207286 (0.3013356393) 
 
 
 Postcentral (mean (SD)) 
 2.0375742862 (0.1796109149) 
 
 
 Paracentral.lobule (mean (SD)) 
 1.2872489540 (0.1404521846) 
 
 
 Subcentral (mean (SD)) 
 0.5533885997 (0.0701240033) 
 
 
 SPL (mean (SD)) 
 3.0512299837 (0.2995764614) 
 
 
 SMG (mean (SD)) 
 2.8054702345 (0.2963151197) 
 
 
 ANG (mean (SD)) 
 3.1401508432 (0.2974069556) 
 
 
 Precuneus (mean (SD)) 
 2.8184789289 (0.2195354334) 
 
 
 Cuneus (mean (SD)) 
 0.9218075932 (0.1105395289) 
 
 
 O1 (mean (SD)) 
 0.8363347713 (0.0794530354) 
 
 
 O2 (mean (SD)) 
 1.4412036853 (0.1395067116) 
 
 
 O3 (mean (SD)) 
 0.8459452532 (0.1135967778) 
 
 
 Occipital.pole (mean (SD)) 
 1.0401371468 (0.0957630116) 
 
 
 Lingual (mean (SD)) 
 1.8611729124 (0.2307977883) 
 
 
 Fusiform (mean (SD)) 
 2.0313915705 (0.2297491283) 
 
 
 Temporal.pole (mean (SD)) 
 2.3163307043 (0.1969989384) 
 
 
 T1 (mean (SD)) 
 2.4899711225 (0.2314929390) 
 
 
 T2 (mean (SD)) 
 2.1710340083 (0.2222231589) 
 
 
 T3 (mean (SD)) 
 0.4573538448 (0.0569894934) 
 
 
 Planum.temporale (mean (SD)) 
 0.2805395089 (0.0570436553) 
 
 
 Planum.polare (mean (SD)) 
 0.9110250954 (0.1711155137) 
 
 
 Short.insular (mean (SD)) 
 1.4149000524 (0.1765065390) 
 
 
 Long.insular (mean (SD)) 
 0.8324538909 (0.1196168331) 
 
 
 SCA (mean (SD)) 
 0.2170797247 (0.0471724812) 
 
 
 Cingulate.anterior (mean (SD)) 
 1.3176948779 (0.1348973794) 
 
 
 Cingulate.middle (mean (SD)) 
 1.5637867547 (0.1479091119) 
 
 
 Cingulate.posterior (mean (SD)) 
 1.8413310529 (0.1454271384) 
 
 
 PHG (mean (SD)) 
 0.9563118242 (0.1171393527) 
 
 
 Hippocampus (mean (SD)) 
 0.7446874971 (0.0621830878) 
 
 
 Amygdala (mean (SD)) 
 0.2864526632 (0.0401036938) 
 
 
 Corpus.callosum (mean (SD)) 
 0.3039193139 (0.0468123806) 
 
 
 Claustrum (mean (SD)) 
 0.1228498024 (0.0358816349) 
 
 
 Putamen (mean (SD)) 
 1.0344949954 (0.1388220008) 
 
 
 Caudate (mean (SD)) 
 0.7138494977 (0.1087637962) 
 
 
 Globus.pallidum (mean (SD)) 
 0.2805060774 (0.0391642507) 
 
 
 Internal.capsule (mean (SD)) 
 0.9724042811 (0.1172447094) 
 
 
 Innominate.substance (mean (SD)) 
 0.2477217623 (0.0361218477) 
 
 
 Hypothalamus (mean (SD)) 
 0.7289368930 (0.0482037353) 
 
 
 Thalamus (mean (SD)) 
 1.3400717256 (0.1017134094) 
 
 
 


 
   
  kable(Table.Prosencephalon.relative.RSD)  
 
 
 

 
 
 
 Table.Prosencephalon.relative.RSD  X 2/ T  a  b  l  e . P  r  o  s  e  n  c  e  p  h  a  l  o  n . r  e  l  a  t  i  v  e . R  S  D  X1 
 
 
 
 
 17.1 
 
 
 6.2 
 
 
 6.0 
 
 
 11.2 
 
 
 13.6 
 
 
 11.7 
 
 
 12.2 
 
 
 12.3 
 
 
 12.4 
 
 
 12.3 
 
 
 43.9 
 
 
 7.6 
 
 
 8.4 
 
 
 8.8 
 
 
 10.9 
 
 
 12.7 
 
 
 9.8 
 
 
 10.6 
 
 
 9.5 
 
 
 7.8 
 
 
 12.0 
 
 
 9.5 
 
 
 9.7 
 
 
 13.4 
 
 
 9.2 
 
 
 12.4 
 
 
 11.3 
 
 
 8.5 
 
 
 9.3 
 
 
 10.2 
 
 
 12.5 
 
 
 20.3 
 
 
 18.8 
 
 
 12.5 
 
 
 14.4 
 
 
 21.7 
 
 
 10.2 
 
 
 9.5 
 
 
 7.9 
 
 
 12.2 
 
 
 8.4 
 
 
 14.0 
 
 
 15.4 
 
 
 29.2 
 
 
 13.4 
 
 
 15.2 
 
 
 14.0 
 
 
 12.1 
 
 
 14.6 
 
 
 6.6 
 
 
 7.6 
 
 
 


 
   
  kable(Table.Prosencephalon.relative.stratified.gender)  
 
 
 

 
 
 
 
 
 
 
 
 
 
  
 f 
 m 
 p 
 test 
 
 
 
 
 n 
 14 
 16 
  
  
 
 
 Frontal.pole (mean (SD)) 
 0.4282337280 (0.0713377035) 
 0.4330757280 (0.0777473097) 
 0.861 
  
 
 
 F1 (mean (SD)) 
 6.3006329062 (0.3639376668) 
 6.2615682601 (0.4160375339) 
 0.788 
  
 
 
 F2 (mean (SD)) 
 5.3883848936 (0.3077446428) 
 5.2932564928 (0.3360665361) 
 0.428 
  
 
 
 F3.orbital (mean (SD)) 
 0.3571578969 (0.0385677622) 
 0.3588024714 (0.0425977851) 
 0.913 
  
 
 
 F3.triangular (mean (SD)) 
 1.0636322212 (0.1197364194) 
 1.0779737323 (0.1684052590) 
 0.793 
  
 
 
 F3.opercular (mean (SD)) 
 1.2495682579 (0.1574229154) 
 1.3030265083 (0.1435004821) 
 0.339 
  
 
 
 Anterior.orbital (mean (SD)) 
 0.3017582832 (0.0164770413) 
 0.3093813908 (0.0493827496) 
 0.586 
  
 
 
 Medial.orbital (mean (SD)) 
 0.6815918090 (0.0371181605) 
 0.6988864137 (0.1120228996) 
 0.586 
  
 
 
 Lateral.orbital (mean (SD)) 
 0.5464232617 (0.0304519052) 
 0.5598805996 (0.0902891005) 
 0.600 
  
 
 
 Posterior.orbital (mean (SD)) 
 0.6691168234 (0.0367863500) 
 0.6849583960 (0.1100474777) 
 0.612 
  
 
 
 Rectus (mean (SD)) 
 0.8532393638 (0.0662940918) 
 0.9661109520 (0.5486963089) 
 0.452 
  
 
 
 Rostral (mean (SD)) 
 0.3339400566 (0.0264801278) 
 0.3253333540 (0.0239001532) 
 0.357 
  
 
 
 Precentral (mean (SD)) 
 3.6104693496 (0.2468897213) 
 3.5665031852 (0.3489460002) 
 0.697 
  
 
 
 Postcentral (mean (SD)) 
 2.0354425348 (0.1884559911) 
 2.0394395687 (0.1777112761) 
 0.953 
  
 
 
 Paracentral.lobule (mean (SD)) 
 1.3045162856 (0.1438324556) 
 1.2721400388 (0.1403115272) 
 0.538 
  
 
 
 Subcentral (mean (SD)) 
 0.5355965755 (0.0569946874) 
 0.5689566208 (0.0783433358) 
 0.199 
  
 
 
 SPL (mean (SD)) 
 3.0950976636 (0.2771645426) 
 3.0128457637 (0.3218134493) 
 0.463 
  
 
 
 SMG (mean (SD)) 
 2.7870612121 (0.2560750682) 
 2.8215781290 (0.3351530207) 
 0.756 
  
 
 
 ANG (mean (SD)) 
 3.1074492113 (0.3335758333) 
 3.1687647712 (0.2696241186) 
 0.582 
  
 
 
 Precuneus (mean (SD)) 
 2.7769055892 (0.2074598065) 
 2.8548556011 (0.2298977990) 
 0.341 
  
 
 
 Cuneus (mean (SD)) 
 0.9162725007 (0.1085944316) 
 0.9266507992 (0.1155395028) 
 0.803 
  
 
 
 O1 (mean (SD)) 
 0.8107747580 (0.0725777711) 
 0.8586997830 (0.0805991452) 
 0.100 
  
 
 
 O2 (mean (SD)) 
 1.4338278528 (0.1550060300) 
 1.4476575387 (0.1292605063) 
 0.792 
  
 
 
 O3 (mean (SD)) 
 0.8091809468 (0.1170210398) 
 0.8781140214 (0.1035122305) 
 0.098 
  
 
 
 Occipital.pole (mean (SD)) 
 1.0453411723 (0.1129383712) 
 1.0355836245 (0.0814121997) 
 0.786 
  
 
 
 Lingual (mean (SD)) 
 1.9260643720 (0.2287149262) 
 1.8043928852 (0.2242302480) 
 0.153 
  
 
 
 Fusiform (mean (SD)) 
 2.0431163714 (0.2142611581) 
 2.0211323697 (0.2490441211) 
 0.799 
  
 
 
 Temporal.pole (mean (SD)) 
 2.2887424248 (0.1717974683) 
 2.3404704489 (0.2193601055) 
 0.483 
  
 
 
 T1 (mean (SD)) 
 2.4388032912 (0.1640313381) 
 2.5347429750 (0.2751451068) 
 0.265 
  
 
 
 T2 (mean (SD)) 
 2.2309720524 (0.1865495819) 
 2.1185882198 (0.2429535202) 
 0.171 
  
 
 
 T3 (mean (SD)) 
 0.4422376645 (0.0597357531) 
 0.4705805025 (0.0527885654) 
 0.179 
  
 
 
 Planum.temporale (mean (SD)) 
 0.2652397597 (0.0370145950) 
 0.2939267894 (0.0685126147) 
 0.174 
  
 
 
 Planum.polare (mean (SD)) 
 0.9612340750 (0.1660219988) 
 0.8670922381 (0.1682532577) 
 0.135 
  
 
 
 Short.insular (mean (SD)) 
 1.4338625682 (0.1204101258) 
 1.3983078510 (0.2168811526) 
 0.591 
  
 
 
 Long.insular (mean (SD)) 
 0.7888303012 (0.0888448680) 
 0.8706245319 (0.1322545191) 
 0.060 
  
 
 
 SCA (mean (SD)) 
 0.2044625144 (0.0441130883) 
 0.2281197836 (0.0483429862) 
 0.175 
  
 
 
 Cingulate.anterior (mean (SD)) 
 1.3135641868 (0.1439613708) 
 1.3213092326 (0.1311107677) 
 0.879 
  
 
 
 Cingulate.middle (mean (SD)) 
 1.5619775543 (0.1253223665) 
 1.5653698051 (0.1693469993) 
 0.951 
  
 
 
 Cingulate.posterior (mean (SD)) 
 1.8380656367 (0.1343469675) 
 1.8441882921 (0.1588299348) 
 0.911 
  
 
 
 PHG (mean (SD)) 
 0.9762747018 (0.0728687435) 
 0.9388443063 (0.1457024489) 
 0.392 
  
 
 
 Hippocampus (mean (SD)) 
 0.7655342036 (0.0635206138) 
 0.7264466289 (0.0567298899) 
 0.086 
  
 
 
 Amygdala (mean (SD)) 
 0.2948215970 (0.0466054982) 
 0.2791298460 (0.0332319267) 
 0.293 
  
 
 
 Corpus.callosum (mean (SD)) 
 0.3231162484 (0.0427646174) 
 0.2871219961 (0.0447975428) 
 0.033 
  
 
 
 Claustrum (mean (SD)) 
 0.1157108693 (0.0257804133) 
 0.1290963689 (0.0427077348) 
 0.316 
  
 
 
 Putamen (mean (SD)) 
 1.0552049804 (0.1037510009) 
 1.0163737585 (0.1648596697) 
 0.454 
  
 
 
 Caudate (mean (SD)) 
 0.7197152028 (0.0745225273) 
 0.7087170058 (0.1341534632) 
 0.788 
  
 
 
 Globus.pallidum (mean (SD)) 
 0.2799215892 (0.0302657494) 
 0.2810175045 (0.0465933809) 
 0.941 
  
 
 
 Internal.capsule (mean (SD)) 
 0.9899260573 (0.1268862057) 
 0.9570727269 (0.1099341721) 
 0.454 
  
 
 
 Innominate.substance (mean (SD)) 
 0.2587625040 (0.0391948981) 
 0.2380611133 (0.0312707598) 
 0.119 
  
 
 
 Hypothalamus (mean (SD)) 
 0.7337200336 (0.0472119392) 
 0.7247516451 (0.0502044289) 
 0.620 
  
 
 
 Thalamus (mean (SD)) 
 1.3867798315 (0.0776479089) 
 1.2992021329 (0.1046820913) 
 0.016 
  
 
 
 


 
   
  kable(Table.Prosencephalon.relative.stratified.gender.RSD.female)  
 
 
 

 
 
 
 
 
 
 Table.Prosencephalon.relative.stratified.gender.RSD.female  X 2/ T  a  b  l  e . P  r  o  s  e  n  c  e  p  h  a  l  o  n . r  e  l  a  t  i  v  e . s  t  r  a  t  i  f  i  e  d . g  e  n  d  e  r . R  S  D . f  e  m  a  l  e  X1 
 
 
 
 
 16.7 
 
 
 5.8 
 
 
 5.7 
 
 
 10.8 
 
 
 11.3 
 
 
 12.6 
 
 
 5.5 
 
 
 5.4 
 
 
 5.6 
 
 
 5.5 
 
 
 7.8 
 
 
 7.9 
 
 
 6.8 
 
 
 9.3 
 
 
 11.0 
 
 
 10.6 
 
 
 9.0 
 
 
 9.2 
 
 
 10.7 
 
 
 7.5 
 
 
 11.9 
 
 
 9.0 
 
 
 10.8 
 
 
 14.5 
 
 
 10.8 
 
 
 11.9 
 
 
 10.5 
 
 
 7.5 
 
 
 6.7 
 
 
 8.4 
 
 
 13.5 
 
 
 14.0 
 
 
 17.3 
 
 
 8.4 
 
 
 11.3 
 
 
 21.6 
 
 
 11.0 
 
 
 8.0 
 
 
 7.3 
 
 
 7.5 
 
 
 8.3 
 
 
 15.8 
 
 
 13.2 
 
 
 22.3 
 
 
 9.8 
 
 
 10.4 
 
 
 10.8 
 
 
 12.8 
 
 
 15.1 
 
 
 6.4 
 
 
 5.6 
 
 
 


 
   
  kable(Table.Prosencephalon.relative.stratified.gender.RSD.male)  
 
 
 

 
 
 
 
 
 
 Table.Prosencephalon.relative.stratified.gender.RSD.male  X 2/ T  a  b  l  e . P  r  o  s  e  n  c  e  p  h  a  l  o  n . r  e  l  a  t  i  v  e . s  t  r  a  t  i  f  i  e  d . g  e  n  d  e  r . R  S  D . m  a  l  e  X1 
 
 
 
 
 18.0 
 
 
 6.6 
 
 
 6.3 
 
 
 11.9 
 
 
 15.6 
 
 
 11.0 
 
 
 16.0 
 
 
 16.0 
 
 
 16.1 
 
 
 16.1 
 
 
 56.8 
 
 
 7.3 
 
 
 9.8 
 
 
 8.7 
 
 
 11.0 
 
 
 13.8 
 
 
 10.7 
 
 
 11.9 
 
 
 8.5 
 
 
 8.1 
 
 
 12.5 
 
 
 9.4 
 
 
 8.9 
 
 
 11.8 
 
 
 7.9 
 
 
 12.4 
 
 
 12.3 
 
 
 9.4 
 
 
 10.9 
 
 
 11.5 
 
 
 11.2 
 
 
 23.3 
 
 
 19.4 
 
 
 15.5 
 
 
 15.2 
 
 
 21.2 
 
 
 9.9 
 
 
 10.8 
 
 
 8.6 
 
 
 15.5 
 
 
 7.8 
 
 
 11.9 
 
 
 15.6 
 
 
 33.1 
 
 
 16.2 
 
 
 18.9 
 
 
 16.6 
 
 
 11.5 
 
 
 13.1 
 
 
 6.9 
 
 
 8.1 
 
 
 


 
   
  NA  
 
 
 
 
 
 
  
Prosencephalon.relative1 <- select(Prosencephalon.relative1, - c(Age))

names.anatomical.structures.temporary <- c("Frontal.pole",
  "F1",
  "F2",
  "F3.orbital", 
  "F3.triangular", 
  "F3.opercular", 
  "Anterior.orbital", 
  "Medial.orbital", 
  "Lateral.orbital", 
  "Posterior.orbital",
  "Rectus", 
  "Rostral", 
  "Precentral", 
  "Postcentral", 
  "Paracentral.lobule", 
  "Subcentral", 
  "SPL", 
  "SMG", 
  "ANG", 
  "Precuneus",
  "Cuneus", 
  "O1",
  "O2", 
  "O3", 
  "Occipital.pole", 
  "Lingual", 
  "Fusiform", 
  "Temporal.pole", 
  "T1",
  "T2", 
  "T3", 
  "Planum.temporale", 
  "Planum.polare", 
  "Short.insular", 
  "Long.insular", 
  "SCA", 
  "Cingulate.anterior", 
  "Cingulate.middle", 
  "Cingulate.posterior", 
  "PHG")

names.anatomical.structures.definitive <- c("Frontal pole",
  "F1",
  "F2",
  "F3 orbital", 
  "F3 triangular", 
  "F3 opercular", 
  "Anterior orbital", 
  "Medial orbital", 
  "Lateral orbital", 
  "Posterior orbital",
  "Rectus", 
  "Rostral", 
  "Precentral", 
  "Postcentral", 
  "Paracentral lobule", 
  "Subcentral", 
  "SPL", 
  "SMG", 
  "ANG", 
  "Precuneus",
  "Cuneus", 
  "O1",
  "O2", 
  "O3", 
  "Occipital pole", 
  "Lingual", 
  "Fusiform", 
  "Temporal pole", 
  "T1",
  "T2", 
  "T3", 
  "Planum temporale", 
  "Planum polare", 
  "Short insular", 
  "Long insular", 
  "SCA", 
  "Cingulate anterior", 
  "Cingulate middle", 
  "Cingulate posterior", 
  "PHG")

Prosencephalon.relative.plotdata1 <- gather(Prosencephalon.relative1, "anatomical.structure", "relative.volume")
Prosencephalon.relative.plotdata1$Gender <- All.Volumes$Gender
Prosencephalon.relative.plotdata1$Age <- All.Volumes$`Age (years)`

Prosencephalon.relative.plotdata1$Gender <- factor(Prosencephalon.relative.plotdata1$Gender, levels = c("f", "m"), c("f", "m"))
Prosencephalon.relative.plotdata1$anatomical.structure <- factor(Prosencephalon.relative.plotdata1$anatomical.structure, 
                                                                levels = rev(c(names.anatomical.structures.temporary)), rev(c(names.anatomical.structures.definitive)))

Prosencephalon.relative.plot1 <-  ggplot(Prosencephalon.relative.plotdata1, aes(x=anatomical.structure, y = relative.volume))  +
  stat_summary(alpha = 0.3, fun = mean, geom = "bar", width = 0.3, fill = "gray50") + 
  geom_boxplot(aes(fill = Gender), alpha = 0.5, width = 0.4, size = 0.2, position = position_dodge(width = 0.6), 
               outlier.shape = NA, color = "gray30") +
  scale_fill_manual(values = c("chartreuse4", "orangered2")) +
  geom_quasirandom(aes(color = Age), size = 0.7, alpha = 0.8, shape = 16, position = "dodge") +
  scale_color_continuous(low = "steelblue1", high = "red4") +
  xlab("") + ylab("Relative volume (in %)") +
  theme_minimal() +
  coord_flip() +
  ggtitle("CEREBRAL GYRI") +
  theme(plot.title = element_text(hjust = 0.5))

Prosencephalon.relative.plot1
ggsave("Prosencephalon.relative.plot1.pdf", plot = Prosencephalon.relative.plot1, width = 14, height = 12, units = "in", dpi = 600)
  
 
 
   
 
 
 
 
 
 
  
names.anatomical.structures.temporary <- c(
  "Corpus.callosum", 
  "Claustrum", 
  "Putamen", 
  "Caudate", 
  "Globus.pallidum", 
  "Internal.capsule",
  "Innominate.substance", 
  "Hypothalamus",
  "Thalamus", 
  "Hippocampus", 
  "Amygdala")

names.anatomical.structures.definitive <- c(
  "Corpus callosum", 
  "Claustrum", 
  "Putamen", 
  "Caudate", 
  "Globus pallidum", 
  "Internal capsule",
  "Innominate substance", 
  "Hypothalamus",
  "Thalamus",
  "Hippocampus", 
  "Amygdala")

Prosencephalon.relative.plotdata2 <- gather(Prosencephalon.relative2, "anatomical.structure", "relative.volume")
Prosencephalon.relative.plotdata2$Gender <- All.Volumes$Gender
Prosencephalon.relative.plotdata2$Age <- All.Volumes$`Age (years)`

Prosencephalon.relative.plotdata2$Gender <- factor(Prosencephalon.relative.plotdata2$Gender, levels = c("f", "m"), c("f", "m"))
Prosencephalon.relative.plotdata2$anatomical.structure <- factor(Prosencephalon.relative.plotdata2$anatomical.structure, 
                                                                levels = rev(c(names.anatomical.structures.temporary)), rev(c(names.anatomical.structures.definitive)))

Prosencephalon.relative.plot2 <-  ggplot(Prosencephalon.relative.plotdata2, aes(x=anatomical.structure, y = relative.volume))  +
  stat_summary(alpha = 0.3, fun = mean, geom = "bar", width = 0.3, fill = "gray50") + 
  geom_boxplot(aes(fill = Gender), alpha = 0.5, width = 0.4, size = 0.2, position = position_dodge(width = 0.6), 
               outlier.shape = NA, color = "gray30") +
  scale_fill_manual(values = c("chartreuse4", "orangered2")) +
  geom_quasirandom(aes(color = Age), size = 0.7, alpha = 0.8, shape = 16, position = "dodge") +
  scale_color_continuous(low = "steelblue1", high = "red4") +
  xlab("") + ylab("Relative volume (in %)") +
  theme_minimal() +
  coord_flip() +
  ggtitle("CENTRAL PROSENCEPHALON") +
  theme(plot.title = element_text(hjust = 0.5))

Prosencephalon.relative.plot2
ggsave("Prosencephalon.relative.plot2.pdf", plot = Prosencephalon.relative.plot2, width = 10, height = 6, units = "in", dpi = 600)
  
 
 
   
 
 
 
 
 
 
  
#Prosencephalon.relative$Gender <- All.Volumes$Gender
Prosencephalon.relative$Age <- All.Volumes$`Age (years)`

Relative.Frontal.pole.Age.plot <-  ggplot(Prosencephalon.relative, aes(y=Frontal.pole, x = Age))  +
  geom_point(aes(color = Gender), size = 1.5, alpha = 1, shape = 16) +
  scale_color_manual(values = c("chartreuse4", "orangered2")) +
  geom_smooth(method='lm', alpha = 0.2, colour = "dodgerblue4", size = 0.8, weight = 0.3) +
  geom_smooth(aes(color = Gender), method='lm', se = F, alpha = 0.2, linetype = "longdash", size = 0.3, weight = 0.3) +
  stat_cor(method = "pearson", label.y = 0.57, label.x = 70, color = "dodgerblue4") +
  ylab("Relative Volume (in %)") + xlab("Age (in years)") +
  theme_minimal() +
  ggtitle("RELATIVE VOLUME frontal pole") +
  theme(plot.title = element_text(hjust = 0.5))
Relative.Frontal.pole.Age.plot
ggsave("Relative.Frontal.pole.Age.plot.pdf", plot = Relative.Frontal.pole.Age.plot, width = 8, height = 6, units = "in", dpi = 600)  
 
 
   
 
 
  
Relative.F1.Age.plot <-  ggplot(Prosencephalon.relative, aes(y=F1, x = Age))  +
  geom_point(aes(color = Gender), size = 1.5, alpha = 1, shape = 16) +
  scale_color_manual(values = c("chartreuse4", "orangered2")) +
  geom_smooth(method='lm', alpha = 0.2, colour = "dodgerblue4", size = 0.8, weight = 0.3) +
  geom_smooth(aes(color = Gender), method='lm', se = F, alpha = 0.2, linetype = "longdash", size = 0.3, weight = 0.3) +
  stat_cor(method = "pearson", label.y = 6.6, label.x = 70, color = "dodgerblue4") +
  ylab("Relative Volume (in %)") + xlab("Age (in years)") +
  theme_minimal() +
  ggtitle("RELATIVE VOLUME F1") +
  theme(plot.title = element_text(hjust = 0.5))
Relative.F1.Age.plot
ggsave("Relative.F1.Age.plot.pdf", plot = Relative.F1.Age.plot, width = 8, height = 6, units = "in", dpi = 600)  
 
 
   
 
 
  
Relative.F2.Age.plot <-  ggplot(Prosencephalon.relative, aes(y=F2, x = Age))  +
  geom_point(aes(color = Gender), size = 1.5, alpha = 1, shape = 16) +
  scale_color_manual(values = c("chartreuse4", "orangered2")) +
  geom_smooth(method='lm', alpha = 0.2, colour = "dodgerblue4", size = 0.8, weight = 0.3) +
  geom_smooth(aes(color = Gender), method='lm', se = F, alpha = 0.2, linetype = "longdash", size = 0.3, weight = 0.3) +
  stat_cor(method = "pearson", label.y = 5.95, label.x = 70, color = "dodgerblue4") +
  ylab("Relative Volume (in %)") + xlab("Age (in years)") +
  theme_minimal() +
  ggtitle("RELATIVE VOLUME F2") +
  theme(plot.title = element_text(hjust = 0.5))
Relative.F2.Age.plot
ggsave("Relative.F2.Age.plot.pdf", plot = Relative.F2.Age.plot, width = 8, height = 6, units = "in", dpi = 600)  
 
 
   
 
 
  
Relative.F3.orbital.Age.plot <-  ggplot(Prosencephalon.relative, aes(y=F3.orbital, x = Age))  +
  geom_point(aes(color = Gender), size = 1.5, alpha = 1, shape = 16) +
  scale_color_manual(values = c("chartreuse4", "orangered2")) +
  geom_smooth(method='lm', alpha = 0.2, colour = "dodgerblue4", size = 0.8, weight = 0.3) +
  geom_smooth(aes(color = Gender), method='lm', se = F, alpha = 0.2, linetype = "longdash", size = 0.3, weight = 0.3) +
  stat_cor(method = "pearson", label.y = 0.41, label.x = 70, color = "dodgerblue4") +
  ylab("Relative Volume (in %)") + xlab("Age (in years)") +
  theme_minimal() +
  ggtitle("RELATIVE VOLUME F3 orbital") +
  theme(plot.title = element_text(hjust = 0.5))
Relative.F3.orbital.Age.plot
ggsave("Relative.F3.orbital.Age.plot.pdf", plot = Relative.F3.orbital.Age.plot, width = 8, height = 6, units = "in", dpi = 600)  
 
 
   
 
 
  
Relative.F3.triangular.Age.plot <-  ggplot(Prosencephalon.relative, aes(y=F3.triangular, x = Age))  +
  geom_point(aes(color = Gender), size = 1.5, alpha = 1, shape = 16) +
  scale_color_manual(values = c("chartreuse4", "orangered2")) +
  geom_smooth(method='lm', alpha = 0.2, colour = "dodgerblue4", size = 0.8, weight = 0.3) +
  geom_smooth(aes(color = Gender), method='lm', se = F, alpha = 0.2, linetype = "longdash", size = 0.3, weight = 0.3) +
  stat_cor(method = "pearson", label.y = 1.22, label.x = 70, color = "dodgerblue4") +
  ylab("Relative Volume (in %)") + xlab("Age (in years)") +
  theme_minimal() +
  ggtitle("RELATIVE VOLUME F3 triangular") +
  theme(plot.title = element_text(hjust = 0.5))
Relative.F3.triangular.Age.plot
ggsave("Relative.F3.triangular.Age.plot.pdf", plot = Relative.F3.triangular.Age.plot, width = 8, height = 6, units = "in", dpi = 600)  
 
 
   
 
 
  
Relative.F3.opercular.Age.plot <-  ggplot(Prosencephalon.relative, aes(y=F3.opercular, x = Age))  +
  geom_point(aes(color = Gender), size = 1.5, alpha = 1, shape = 16) +
  scale_color_manual(values = c("chartreuse4", "orangered2")) +
  geom_smooth(method='lm', alpha = 0.2, colour = "dodgerblue4", size = 0.8, weight = 0.3) +
  geom_smooth(aes(color = Gender), method='lm', se = F, alpha = 0.2, linetype = "longdash", size = 0.3, weight = 0.3) +
  stat_cor(method = "pearson", label.y = 1.65, label.x = 70, color = "dodgerblue4") +
  ylab("Relative Volume (in %)") + xlab("Age (in years)") +
  theme_minimal() +
  ggtitle("RELATIVE VOLUME F3 opercular") +
  theme(plot.title = element_text(hjust = 0.5))
Relative.F3.opercular.Age.plot
ggsave("Relative.F3.opercular.Age.plot.pdf", plot = Relative.F3.opercular.Age.plot, width = 8, height = 6, units = "in", dpi = 600)  
 
 
   
 
 
  
Relative.Anterior.orbital.Age.plot <-  ggplot(Prosencephalon.relative, aes(y=Anterior.orbital, x = Age))  +
  geom_point(aes(color = Gender), size = 1.5, alpha = 1, shape = 16) +
  scale_color_manual(values = c("chartreuse4", "orangered2")) +
  geom_smooth(method='lm', alpha = 0.2, colour = "dodgerblue4", size = 0.8, weight = 0.3) +
  geom_smooth(aes(color = Gender), method='lm', se = F, alpha = 0.2, linetype = "longdash", size = 0.3, weight = 0.3) +
  stat_cor(method = "pearson", label.y = 0.385, label.x = 70, color = "dodgerblue4") +
  ylab("Relative Volume (in %)") + xlab("Age (in years)") +
  theme_minimal() +
  ggtitle("RELATIVE VOLUME anterior orbital") +
  theme(plot.title = element_text(hjust = 0.5))
Relative.Anterior.orbital.Age.plot
ggsave("Relative.Anterior.orbital.Age.plot.pdf", plot = Relative.Anterior.orbital.Age.plot, width = 8, height = 6, units = "in", dpi = 600)  
 
 
   
 
 
  
Relative.Medial.orbital.Age.plot <-  ggplot(Prosencephalon.relative, aes(y=Medial.orbital, x = Age))  +
  geom_point(aes(color = Gender), size = 1.5, alpha = 1, shape = 16) +
  scale_color_manual(values = c("chartreuse4", "orangered2")) +
  geom_smooth(method='lm', alpha = 0.2, colour = "dodgerblue4", size = 0.8, weight = 0.3) +
  geom_smooth(aes(color = Gender), method='lm', se = F, alpha = 0.2, linetype = "longdash", size = 0.3, weight = 0.3) +
  stat_cor(method = "pearson", label.y = 0.87, label.x = 70, color = "dodgerblue4") +
  ylab("Relative Volume (in %)") + xlab("Age (in years)") +
  theme_minimal() +
  ggtitle("RELATIVE VOLUME medial orbital") +
  theme(plot.title = element_text(hjust = 0.5))
Relative.Medial.orbital.Age.plot
ggsave("Relative.Medial.orbital.Age.plot.pdf", plot = Relative.Medial.orbital.Age.plot, width = 8, height = 6, units = "in", dpi = 600)  
 
 
   
 
 
  
Relative.Lateral.orbital.Age.plot <-  ggplot(Prosencephalon.relative, aes(y=Lateral.orbital, x = Age))  +
  geom_point(aes(color = Gender), size = 1.5, alpha = 1, shape = 16) +
  scale_color_manual(values = c("chartreuse4", "orangered2")) +
  geom_smooth(method='lm', alpha = 0.2, colour = "dodgerblue4", size = 0.8, weight = 0.3) +
  geom_smooth(aes(color = Gender), method='lm', se = F, alpha = 0.2, linetype = "longdash", size = 0.3, weight = 0.3) +
  stat_cor(method = "pearson", label.y = 0.67, label.x = 70, color = "dodgerblue4") +
  ylab("Relative Volume (in %)") + xlab("Age (in years)") +
  theme_minimal() +
  ggtitle("RELATIVE VOLUME lateral orbital") +
  theme(plot.title = element_text(hjust = 0.5))
Relative.Lateral.orbital.Age.plot
ggsave("Relative.Lateral.orbital.Age.plot.pdf", plot = Relative.Lateral.orbital.Age.plot, width = 8, height = 6, units = "in", dpi = 600)  
 
 
   
 
 
  
Relative.Posterior.orbital.Age.plot <-  ggplot(Prosencephalon.relative, aes(y=Posterior.orbital, x = Age))  +
  geom_point(aes(color = Gender), size = 1.5, alpha = 1, shape = 16) +
  scale_color_manual(values = c("chartreuse4", "orangered2")) +
  geom_smooth(method='lm', alpha = 0.2, colour = "dodgerblue4", size = 0.8, weight = 0.3) +
  geom_smooth(aes(color = Gender), method='lm', se = F, alpha = 0.2, linetype = "longdash", size = 0.3, weight = 0.3) +
  stat_cor(method = "pearson", label.y = 0.82, label.x = 70, color = "dodgerblue4") +
  ylab("Relative Volume (in %)") + xlab("Age (in years)") +
  theme_minimal() +
  ggtitle("RELATIVE VOLUME posterior orbital") +
  theme(plot.title = element_text(hjust = 0.5))
Relative.Posterior.orbital.Age.plot
ggsave("Relative.Posterior.orbital.Age.plot.pdf", plot = Relative.Posterior.orbital.Age.plot, width = 8, height = 6, units = "in", dpi = 600)  
 
 
   
 
 
  
Relative.Rectus.Age.plot <-  ggplot(Prosencephalon.relative, aes(y=Rectus, x = Age))  +
  geom_point(aes(color = Gender), size = 1.5, alpha = 1, shape = 16) +
  scale_color_manual(values = c("chartreuse4", "orangered2")) +
  geom_smooth(method='lm', alpha = 0.2, colour = "dodgerblue4", size = 0.8, weight = 0.3) +
  geom_smooth(aes(color = Gender), method='lm', se = F, alpha = 0.2, linetype = "longdash", size = 0.3, weight = 0.3) +
  stat_cor(method = "pearson", label.y = 1.8, label.x = 70, color = "dodgerblue4") +
  ylab("Relative Volume (in %)") + xlab("Age (in years)") +
  theme_minimal() +
  ggtitle("RELATIVE VOLUME rectus") +
  theme(plot.title = element_text(hjust = 0.5))
Relative.Rectus.Age.plot
ggsave("Relative.Rectus.Age.plot.pdf", plot = Relative.Rectus.Age.plot, width = 8, height = 6, units = "in", dpi = 600)  
 
 
   
 
 
  
Relative.Rostral.Age.plot <-  ggplot(Prosencephalon.relative, aes(y=Rostral, x = Age))  +
  geom_point(aes(color = Gender), size = 1.5, alpha = 1, shape = 16) +
  scale_color_manual(values = c("chartreuse4", "orangered2")) +
  geom_smooth(method='lm', alpha = 0.2, colour = "dodgerblue4", size = 0.8, weight = 0.3) +
  geom_smooth(aes(color = Gender), method='lm', se = F, alpha = 0.2, linetype = "longdash", size = 0.3, weight = 0.3) +
  stat_cor(method = "pearson", label.y = 0.41, label.x = 70, color = "dodgerblue4") +
  ylab("Relative Volume (in %)") + xlab("Age (in years)") +
  theme_minimal() +
  ggtitle("RELATIVE VOLUME rostral") +
  theme(plot.title = element_text(hjust = 0.5))
Relative.Rostral.Age.plot
ggsave("Relative.Rostral.Age.plot.pdf", plot = Relative.Rostral.Age.plot, width = 8, height = 6, units = "in", dpi = 600)  
 
 
   
 
 
  
Relative.Precentral.Age.plot <-  ggplot(Prosencephalon.relative, aes(y=Precentral, x = Age))  +
  geom_point(aes(color = Gender), size = 1.5, alpha = 1, shape = 16) +
  scale_color_manual(values = c("chartreuse4", "orangered2")) +
  geom_smooth(method='lm', alpha = 0.2, colour = "dodgerblue4", size = 0.8, weight = 0.3) +
  geom_smooth(aes(color = Gender), method='lm', se = F, alpha = 0.2, linetype = "longdash", size = 0.3, weight = 0.3) +
  stat_cor(method = "pearson", label.y = 4.6, label.x = 70, color = "dodgerblue4") +
  ylab("Relative Volume (in %)") + xlab("Age (in years)") +
  theme_minimal() +
  ggtitle("RELATIVE VOLUME precentral") +
  theme(plot.title = element_text(hjust = 0.5))
Relative.Precentral.Age.plot
ggsave("Relative.Precentral.Age.plot.pdf", plot = Relative.Precentral.Age.plot, width = 8, height = 6, units = "in", dpi = 600)  
 
 
   
 
 
  
Relative.Postcentral.Age.plot <-  ggplot(Prosencephalon.relative, aes(y=Postcentral, x = Age))  +
  geom_point(aes(color = Gender), size = 1.5, alpha = 1, shape = 16) +
  scale_color_manual(values = c("chartreuse4", "orangered2")) +
  geom_smooth(method='lm', alpha = 0.2, colour = "dodgerblue4", size = 0.8, weight = 0.3) +
  geom_smooth(aes(color = Gender), method='lm', se = F, alpha = 0.2, linetype = "longdash", size = 0.3, weight = 0.3) +
  stat_cor(method = "pearson", label.y = 2.45, label.x = 70, color = "dodgerblue4") +
  ylab("Relative Volume (in %)") + xlab("Age (in years)") +
  theme_minimal() +
  ggtitle("RELATIVE VOLUME postcentral") +
  theme(plot.title = element_text(hjust = 0.5))
Relative.Postcentral.Age.plot
ggsave("Relative.Postcentral.Age.plot.pdf", plot = Relative.Postcentral.Age.plot, width = 8, height = 6, units = "in", dpi = 600)  
 
 
   
 
 
  
Relative.Paracentral.lobule.Age.plot <-  ggplot(Prosencephalon.relative, aes(y=Paracentral.lobule, x = Age))  +
  geom_point(aes(color = Gender), size = 1.5, alpha = 1, shape = 16) +
  scale_color_manual(values = c("chartreuse4", "orangered2")) +
  geom_smooth(method='lm', alpha = 0.2, colour = "dodgerblue4", size = 0.8, weight = 0.3) +
  geom_smooth(aes(color = Gender), method='lm', se = F, alpha = 0.2, linetype = "longdash", size = 0.3, weight = 0.3) +
  stat_cor(method = "pearson", label.y = 1.62, label.x = 70, color = "dodgerblue4") +
  ylab("Relative Volume (in %)") + xlab("Age (in years)") +
  theme_minimal() +
  ggtitle("RELATIVE VOLUME paracentral lobule") +
  theme(plot.title = element_text(hjust = 0.5))
Relative.Paracentral.lobule.Age.plot
ggsave("Relative.Paracentral.lobule.Age.plot.pdf", plot = Relative.Paracentral.lobule.Age.plot, width = 8, height = 6, units = "in", dpi = 600)  
 
 
   
 
 
  
Relative.Subcentral.Age.plot <-  ggplot(Prosencephalon.relative, aes(y=Subcentral, x = Age))  +
  geom_point(aes(color = Gender), size = 1.5, alpha = 1, shape = 16) +
  scale_color_manual(values = c("chartreuse4", "orangered2")) +
  geom_smooth(method='lm', alpha = 0.2, colour = "dodgerblue4", size = 0.8, weight = 0.3) +
  geom_smooth(aes(color = Gender), method='lm', se = F, alpha = 0.2, linetype = "longdash", size = 0.3, weight = 0.3) +
  stat_cor(method = "pearson", label.y = 0.66, label.x = 70, color = "dodgerblue4") +
  ylab("Relative Volume (in %)") + xlab("Age (in years)") +
  theme_minimal() +
  ggtitle("RELATIVE VOLUME subcentral") +
  theme(plot.title = element_text(hjust = 0.5))
Relative.Subcentral.Age.plot
ggsave("Relative.Subcentral.Age.plot.pdf", plot = Relative.Subcentral.Age.plot, width = 8, height = 6, units = "in", dpi = 600)  
 
 
   
 
 
  
Relative.SPL.Age.plot <-  ggplot(Prosencephalon.relative, aes(y=SPL, x = Age))  +
  geom_point(aes(color = Gender), size = 1.5, alpha = 1, shape = 16) +
  scale_color_manual(values = c("chartreuse4", "orangered2")) +
  geom_smooth(method='lm', alpha = 0.2, colour = "dodgerblue4", size = 0.8, weight = 0.3) +
  geom_smooth(aes(color = Gender), method='lm', se = F, alpha = 0.2, linetype = "longdash", size = 0.3, weight = 0.3) +
  stat_cor(method = "pearson", label.y = 3.5, label.x = 70, color = "dodgerblue4") +
  ylab("Relative Volume (in %)") + xlab("Age (in years)") +
  theme_minimal() +
  ggtitle("RELATIVE VOLUME SPL") +
  theme(plot.title = element_text(hjust = 0.5))
Relative.SPL.Age.plot
ggsave("Relative.SPL.Age.plot.pdf", plot = Relative.SPL.Age.plot, width = 8, height = 6, units = "in", dpi = 600)  
 
 
   
 
 
  
Relative.SMG.Age.plot <-  ggplot(Prosencephalon.relative, aes(y=SMG, x = Age))  +
  geom_point(aes(color = Gender), size = 1.5, alpha = 1, shape = 16) +
  scale_color_manual(values = c("chartreuse4", "orangered2")) +
  geom_smooth(method='lm', alpha = 0.2, colour = "dodgerblue4", size = 0.8, weight = 0.3) +
  geom_smooth(aes(color = Gender), method='lm', se = F, alpha = 0.2, linetype = "longdash", size = 0.3, weight = 0.3) +
  stat_cor(method = "pearson", label.y = 3.3, label.x = 70, color = "dodgerblue4") +
  ylab("Relative Volume (in %)") + xlab("Age (in years)") +
  theme_minimal() +
  ggtitle("RELATIVE VOLUME supramarginal") +
  theme(plot.title = element_text(hjust = 0.5))
Relative.SMG.Age.plot
ggsave("Relative.SMG.Age.plot.pdf", plot = Relative.SMG.Age.plot, width = 8, height = 6, units = "in", dpi = 600)  
 
 
   
 
 
  
Relative.ANG.Age.plot <-  ggplot(Prosencephalon.relative, aes(y=ANG, x = Age))  +
  geom_point(aes(color = Gender), size = 1.5, alpha = 1, shape = 16) +
  scale_color_manual(values = c("chartreuse4", "orangered2")) +
  geom_smooth(method='lm', alpha = 0.2, colour = "dodgerblue4", size = 0.8, weight = 0.3) +
  geom_smooth(aes(color = Gender), method='lm', se = F, alpha = 0.2, linetype = "longdash", size = 0.3, weight = 0.3) +
  stat_cor(method = "pearson", label.y = 3.4, label.x = 70, color = "dodgerblue4") +
  ylab("Relative Volume (in %)") + xlab("Age (in years)") +
  theme_minimal() +
  ggtitle("RELATIVE VOLUME angular") +
  theme(plot.title = element_text(hjust = 0.5))
Relative.ANG.Age.plot
ggsave("Relative.ANG.Age.plot.pdf", plot = Relative.ANG.Age.plot, width = 8, height = 6, units = "in", dpi = 600)  
 
 
   
 
 
  
Relative.Precuneus.Age.plot <-  ggplot(Prosencephalon.relative, aes(y=Precuneus, x = Age))  +
  geom_point(aes(color = Gender), size = 1.5, alpha = 1, shape = 16) +
  scale_color_manual(values = c("chartreuse4", "orangered2")) +
  geom_smooth(method='lm', alpha = 0.2, colour = "dodgerblue4", size = 0.8, weight = 0.3) +
  geom_smooth(aes(color = Gender), method='lm', se = F, alpha = 0.2, linetype = "longdash", size = 0.3, weight = 0.3) +
  stat_cor(method = "pearson", label.y = 3.05, label.x = 70, color = "dodgerblue4") +
  ylab("Relative Volume (in %)") + xlab("Age (in years)") +
  theme_minimal() +
  ggtitle("RELATIVE VOLUME precuneus") +
  theme(plot.title = element_text(hjust = 0.5))
Relative.Precuneus.Age.plot
ggsave("Relative.Precuneus.Age.plot.pdf", plot = Relative.Precuneus.Age.plot, width = 8, height = 6, units = "in", dpi = 600)  
 
 
   
 
 
  
Relative.Cuneus.Age.plot <-  ggplot(Prosencephalon.relative, aes(y=Cuneus, x = Age))  +
  geom_point(aes(color = Gender), size = 1.5, alpha = 1, shape = 16) +
  scale_color_manual(values = c("chartreuse4", "orangered2")) +
  geom_smooth(method='lm', alpha = 0.2, colour = "dodgerblue4", size = 0.8, weight = 0.3) +
  geom_smooth(aes(color = Gender), method='lm', se = F, alpha = 0.2, linetype = "longdash", size = 0.3, weight = 0.3) +
  stat_cor(method = "pearson", label.y = 1.07, label.x = 70, color = "dodgerblue4") +
  ylab("Relative Volume (in %)") + xlab("Age (in years)") +
  theme_minimal() +
  ggtitle("RELATIVE VOLUME cuneus") +
  theme(plot.title = element_text(hjust = 0.5))
Relative.Cuneus.Age.plot
ggsave("Relative.Cuneus.Age.plot.pdf", plot = Relative.Cuneus.Age.plot, width = 8, height = 6, units = "in", dpi = 600)  
 
 
   
 
 
  
Relative.O1.Age.plot <-  ggplot(Prosencephalon.relative, aes(y=O1, x = Age))  +
  geom_point(aes(color = Gender), size = 1.5, alpha = 1, shape = 16) +
  scale_color_manual(values = c("chartreuse4", "orangered2")) +
  geom_smooth(method='lm', alpha = 0.2, colour = "dodgerblue4", size = 0.8, weight = 0.3) +
  geom_smooth(aes(color = Gender), method='lm', se = F, alpha = 0.2, linetype = "longdash", size = 0.3, weight = 0.3) +
  stat_cor(method = "pearson", label.y = 0.97, label.x = 70, color = "dodgerblue4") +
  ylab("Relative Volume (in %)") + xlab("Age (in years)") +
  theme_minimal() +
  ggtitle("RELATIVE VOLUME O1") +
  theme(plot.title = element_text(hjust = 0.5))
Relative.O1.Age.plot
ggsave("Relative.O1.Age.plot.pdf", plot = Relative.O1.Age.plot, width = 8, height = 6, units = "in", dpi = 600)  
 
 
   
 
 
  
Relative.O2.Age.plot <-  ggplot(Prosencephalon.relative, aes(y=O2, x = Age))  +
  geom_point(aes(color = Gender), size = 1.5, alpha = 1, shape = 16) +
  scale_color_manual(values = c("chartreuse4", "orangered2")) +
  geom_smooth(method='lm', alpha = 0.2, colour = "dodgerblue4", size = 0.8, weight = 0.3) +
  geom_smooth(aes(color = Gender), method='lm', se = F, alpha = 0.2, linetype = "longdash", size = 0.3, weight = 0.3) +
  stat_cor(method = "pearson", label.y = 1.62, label.x = 70, color = "dodgerblue4") +
  ylab("Relative Volume (in %)") + xlab("Age (in years)") +
  theme_minimal() +
  ggtitle("RELATIVE VOLUME O2") +
  theme(plot.title = element_text(hjust = 0.5))
Relative.O2.Age.plot
ggsave("Relative.O2.Age.plot.pdf", plot = Relative.O2.Age.plot, width = 8, height = 6, units = "in", dpi = 600)  
 
 
   
 
 
  
Relative.O3.Age.plot <-  ggplot(Prosencephalon.relative, aes(y=O3, x = Age))  +
  geom_point(aes(color = Gender), size = 1.5, alpha = 1, shape = 16) +
  scale_color_manual(values = c("chartreuse4", "orangered2")) +
  geom_smooth(method='lm', alpha = 0.2, colour = "dodgerblue4", size = 0.8, weight = 0.3) +
  geom_smooth(aes(color = Gender), method='lm', se = F, alpha = 0.2, linetype = "longdash", size = 0.3, weight = 0.3) +
  stat_cor(method = "pearson", label.y = 1.02, label.x = 70, color = "dodgerblue4") +
  ylab("Relative Volume (in %)") + xlab("Age (in years)") +
  theme_minimal() +
  ggtitle("RELATIVE VOLUME O3") +
  theme(plot.title = element_text(hjust = 0.5))
Relative.O3.Age.plot
ggsave("Relative.O3.Age.plot.pdf", plot = Relative.O3.Age.plot, width = 8, height = 6, units = "in", dpi = 600)  
 
 
   
 
 
  
Relative.Occipital.pole.Age.plot <-  ggplot(Prosencephalon.relative, aes(y=Occipital.pole, x = Age))  +
  geom_point(aes(color = Gender), size = 1.5, alpha = 1, shape = 16) +
  scale_color_manual(values = c("chartreuse4", "orangered2")) +
  geom_smooth(method='lm', alpha = 0.2, colour = "dodgerblue4", size = 0.8, weight = 0.3) +
  geom_smooth(aes(color = Gender), method='lm', se = F, alpha = 0.2, linetype = "longdash", size = 0.3, weight = 0.3) +
  stat_cor(method = "pearson", label.y = 1.17, label.x = 70, color = "dodgerblue4") +
  ylab("Relative Volume (in %)") + xlab("Age (in years)") +
  theme_minimal() +
  ggtitle("RELATIVE VOLUME occipital pole") +
  theme(plot.title = element_text(hjust = 0.5))
Relative.Occipital.pole.Age.plot
ggsave("Relative.Occipital.pole.Age.plot.pdf", plot = Relative.Occipital.pole.Age.plot, width = 8, height = 6, units = "in", dpi = 600)  
 
 
   
 
 
  
Relative.Lingual.Age.plot <-  ggplot(Prosencephalon.relative, aes(y=Lingual, x = Age))  +
  geom_point(aes(color = Gender), size = 1.5, alpha = 1, shape = 16) +
  scale_color_manual(values = c("chartreuse4", "orangered2")) +
  geom_smooth(method='lm', alpha = 0.2, colour = "dodgerblue4", size = 0.8, weight = 0.3) +
  geom_smooth(aes(color = Gender), method='lm', se = F, alpha = 0.2, linetype = "longdash", size = 0.3, weight = 0.3) +
  stat_cor(method = "pearson", label.y = 2.3, label.x = 70, color = "dodgerblue4") +
  ylab("Relative Volume (in %)") + xlab("Age (in years)") +
  theme_minimal() +
  ggtitle("RELATIVE VOLUME lingual") +
  theme(plot.title = element_text(hjust = 0.5))
Relative.Lingual.Age.plot
ggsave("Relative.Lingual.Age.plot.pdf", plot = Relative.Lingual.Age.plot, width = 8, height = 6, units = "in", dpi = 600)  
 
 
   
 
 
  
Relative.Fusiform.Age.plot <-  ggplot(Prosencephalon.relative, aes(y=Fusiform, x = Age))  +
  geom_point(aes(color = Gender), size = 1.5, alpha = 1, shape = 16) +
  scale_color_manual(values = c("chartreuse4", "orangered2")) +
  geom_smooth(method='lm', alpha = 0.2, colour = "dodgerblue4", size = 0.8, weight = 0.3) +
  geom_smooth(aes(color = Gender), method='lm', se = F, alpha = 0.2, linetype = "longdash", size = 0.3, weight = 0.3) +
  stat_cor(method = "pearson", label.y = 2.28, label.x = 70, color = "dodgerblue4") +
  ylab("Relative Volume (in %)") + xlab("Age (in years)") +
  theme_minimal() +
  ggtitle("RELATIVE VOLUME fusiform") +
  theme(plot.title = element_text(hjust = 0.5))
Relative.Fusiform.Age.plot
ggsave("Relative.Fusiform.Age.plot.pdf", plot = Relative.Fusiform.Age.plot, width = 8, height = 6, units = "in", dpi = 600)  
 
 
   
 
 
  
Relative.Temporal.pole.Age.plot <-  ggplot(Prosencephalon.relative, aes(y=Temporal.pole, x = Age))  +
  geom_point(aes(color = Gender), size = 1.5, alpha = 1, shape = 16) +
  scale_color_manual(values = c("chartreuse4", "orangered2")) +
  geom_smooth(method='lm', alpha = 0.2, colour = "dodgerblue4", size = 0.8, weight = 0.3) +
  geom_smooth(aes(color = Gender), method='lm', se = F, alpha = 0.2, linetype = "longdash", size = 0.3, weight = 0.3) +
  stat_cor(method = "pearson", label.y = 2.7, label.x = 70, color = "dodgerblue4") +
  ylab("Relative Volume (in %)") + xlab("Age (in years)") +
  theme_minimal() +
  ggtitle("RELATIVE VOLUME temporal pole") +
  theme(plot.title = element_text(hjust = 0.5))
Relative.Temporal.pole.Age.plot
ggsave("Relative.Temporal.pole.Age.plot.pdf", plot = Relative.Temporal.pole.Age.plot, width = 8, height = 6, units = "in", dpi = 600)  
 
 
   
 
 
  
Relative.T1.Age.plot <-  ggplot(Prosencephalon.relative, aes(y=T1, x = Age))  +
  geom_point(aes(color = Gender), size = 1.5, alpha = 1, shape = 16) +
  scale_color_manual(values = c("chartreuse4", "orangered2")) +
  geom_smooth(method='lm', alpha = 0.2, colour = "dodgerblue4", size = 0.8, weight = 0.3) +
  geom_smooth(aes(color = Gender), method='lm', se = F, alpha = 0.2, linetype = "longdash", size = 0.3, weight = 0.3) +
  stat_cor(method = "pearson", label.y = 2.75, label.x = 70, color = "dodgerblue4") +
  ylab("Relative Volume (in %)") + xlab("Age (in years)") +
  theme_minimal() +
  ggtitle("RELATIVE VOLUME T1") +
  theme(plot.title = element_text(hjust = 0.5))
Relative.T1.Age.plot
ggsave("Relative.T1.Age.plot.pdf", plot = Relative.T1.Age.plot, width = 8, height = 6, units = "in", dpi = 600)  
 
 
   
 
 
  
Relative.T2.Age.plot <-  ggplot(Prosencephalon.relative, aes(y=T2, x = Age))  +
  geom_point(aes(color = Gender), size = 1.5, alpha = 1, shape = 16) +
  scale_color_manual(values = c("chartreuse4", "orangered2")) +
  geom_smooth(method='lm', alpha = 0.2, colour = "dodgerblue4", size = 0.8, weight = 0.3) +
  geom_smooth(aes(color = Gender), method='lm', se = F, alpha = 0.2, linetype = "longdash", size = 0.3, weight = 0.3) +
  stat_cor(method = "pearson", label.y = 2.43, label.x = 70, color = "dodgerblue4") +
  ylab("Relative Volume (in %)") + xlab("Age (in years)") +
  theme_minimal() +
  ggtitle("RELATIVE VOLUME T2") +
  theme(plot.title = element_text(hjust = 0.5))
Relative.T2.Age.plot
ggsave("Relative.T2.Age.plot.pdf", plot = Relative.T2.Age.plot, width = 8, height = 6, units = "in", dpi = 600)  
 
 
   
 
 
  
Relative.T3.Age.plot <-  ggplot(Prosencephalon.relative, aes(y=T3, x = Age))  +
  geom_point(aes(color = Gender), size = 1.5, alpha = 1, shape = 16) +
  scale_color_manual(values = c("chartreuse4", "orangered2")) +
  geom_smooth(method='lm', alpha = 0.2, colour = "dodgerblue4", size = 0.8, weight = 0.3) +
  geom_smooth(aes(color = Gender), method='lm', se = F, alpha = 0.2, linetype = "longdash", size = 0.3, weight = 0.3) +
  stat_cor(method = "pearson", label.y = 0.51, label.x = 70, color = "dodgerblue4") +
  ylab("Relative Volume (in %)") + xlab("Age (in years)") +
  theme_minimal() +
  ggtitle("RELATIVE VOLUME T3") +
  theme(plot.title = element_text(hjust = 0.5))
Relative.T3.Age.plot
ggsave("Relative.T3.Age.plot.pdf", plot = Relative.T3.Age.plot, width = 8, height = 6, units = "in", dpi = 600)  
 
 
   
 
 
  
Relative.Planum.temporale.Age.plot <-  ggplot(Prosencephalon.relative, aes(y=Planum.temporale, x = Age))  +
  geom_point(aes(color = Gender), size = 1.5, alpha = 1, shape = 16) +
  scale_color_manual(values = c("chartreuse4", "orangered2")) +
  geom_smooth(method='lm', alpha = 0.2, colour = "dodgerblue4", size = 0.8, weight = 0.3) +
  geom_smooth(aes(color = Gender), method='lm', se = F, alpha = 0.2, linetype = "longdash", size = 0.3, weight = 0.3) +
  stat_cor(method = "pearson", label.y = 0.335, label.x = 70, color = "dodgerblue4") +
  ylab("Relative Volume (in %)") + xlab("Age (in years)") +
  theme_minimal() +
  ggtitle("RELATIVE VOLUME planum temporale") +
  theme(plot.title = element_text(hjust = 0.5))
Relative.Planum.temporale.Age.plot
ggsave("Relative.Planum.temporale.Age.plot.pdf", plot = Relative.Planum.temporale.Age.plot, width = 8, height = 6, units = "in", dpi = 600)  
 
 
   
 
 
  
Relative.Planum.polare.Age.plot <-  ggplot(Prosencephalon.relative, aes(y=Planum.polare, x = Age))  +
  geom_point(aes(color = Gender), size = 1.5, alpha = 1, shape = 16) +
  scale_color_manual(values = c("chartreuse4", "orangered2")) +
  geom_smooth(method='lm', alpha = 0.2, colour = "dodgerblue4", size = 0.8, weight = 0.3) +
  geom_smooth(aes(color = Gender), method='lm', se = F, alpha = 0.2, linetype = "longdash", size = 0.3, weight = 0.3) +
  stat_cor(method = "pearson", label.y = 1.18, label.x = 70, color = "dodgerblue4") +
  ylab("Relative Volume (in %)") + xlab("Age (in years)") +
  theme_minimal() +
  ggtitle("RELATIVE VOLUME planum polare") +
  theme(plot.title = element_text(hjust = 0.5))
Relative.Planum.polare.Age.plot
ggsave("Relative.Planum.polare.Age.plot.pdf", plot = Relative.Planum.polare.Age.plot, width = 8, height = 6, units = "in", dpi = 600)  
 
 
   
 
 
  
Relative.Short.insular.Age.plot <-  ggplot(Prosencephalon.relative, aes(y=Short.insular, x = Age))  +
  geom_point(aes(color = Gender), size = 1.5, alpha = 1, shape = 16) +
  scale_color_manual(values = c("chartreuse4", "orangered2")) +
  geom_smooth(method='lm', alpha = 0.2, colour = "dodgerblue4", size = 0.8, weight = 0.3) +
  geom_smooth(aes(color = Gender), method='lm', se = F, alpha = 0.2, linetype = "longdash", size = 0.3, weight = 0.3) +
  stat_cor(method = "pearson", label.y = 1.85, label.x = 70, color = "dodgerblue4") +
  ylab("Relative Volume (in %)") + xlab("Age (in years)") +
  theme_minimal() +
  ggtitle("RELATIVE VOLUME short insular") +
  theme(plot.title = element_text(hjust = 0.5))
Relative.Short.insular.Age.plot
ggsave("Relative.Short.insular.Age.plot.pdf", plot = Relative.Short.insular.Age.plot, width = 8, height = 6, units = "in", dpi = 600)  
 
 
   
 
 
  
Relative.Long.insular.Age.plot <-  ggplot(Prosencephalon.relative, aes(y=Long.insular, x = Age))  +
  geom_point(aes(color = Gender), size = 1.5, alpha = 1, shape = 16) +
  scale_color_manual(values = c("chartreuse4", "orangered2")) +
  geom_smooth(method='lm', alpha = 0.2, colour = "dodgerblue4", size = 0.8, weight = 0.3) +
  geom_smooth(aes(color = Gender), method='lm', se = F, alpha = 0.2, linetype = "longdash", size = 0.3, weight = 0.3) +
  stat_cor(method = "pearson", label.y = 0.97, label.x = 70, color = "dodgerblue4") +
  ylab("Relative Volume (in %)") + xlab("Age (in years)") +
  theme_minimal() +
  ggtitle("RELATIVE VOLUME long insular") +
  theme(plot.title = element_text(hjust = 0.5))
Relative.Long.insular.Age.plot
ggsave("Relative.Long.insular.Age.plot.pdf", plot = Relative.Long.insular.Age.plot, width = 8, height = 6, units = "in", dpi = 600)  
 
 
   
 
 
  
Relative.SCA.Age.plot <-  ggplot(Prosencephalon.relative, aes(y=SCA, x = Age))  +
  geom_point(aes(color = Gender), size = 1.5, alpha = 1, shape = 16) +
  scale_color_manual(values = c("chartreuse4", "orangered2")) +
  geom_smooth(method='lm', alpha = 0.2, colour = "dodgerblue4", size = 0.8, weight = 0.3) +
  geom_smooth(aes(color = Gender), method='lm', se = F, alpha = 0.2, linetype = "longdash", size = 0.3, weight = 0.3) +
  stat_cor(method = "pearson", label.y = 0.33, label.x = 70, color = "dodgerblue4") +
  ylab("Relative Volume (in %)") + xlab("Age (in years)") +
  theme_minimal() +
  ggtitle("RELATIVE VOLUME subcallosal area") +
  theme(plot.title = element_text(hjust = 0.5))
Relative.SCA.Age.plot
ggsave("Relative.SCA.Age.plot.pdf", plot = Relative.SCA.Age.plot, width = 8, height = 6, units = "in", dpi = 600)  
 
 
   
 
 
  
Relative.Cingulate.anterior.Age.plot <-  ggplot(Prosencephalon.relative, aes(y=Cingulate.anterior, x = Age))  +
  geom_point(aes(color = Gender), size = 1.5, alpha = 1, shape = 16) +
  scale_color_manual(values = c("chartreuse4", "orangered2")) +
  geom_smooth(method='lm', alpha = 0.2, colour = "dodgerblue4", size = 0.8, weight = 0.3) +
  geom_smooth(aes(color = Gender), method='lm', se = F, alpha = 0.2, linetype = "longdash", size = 0.3, weight = 0.3) +
  stat_cor(method = "pearson", label.y = 1.57, label.x = 70, color = "dodgerblue4") +
  ylab("Relative Volume (in %)") + xlab("Age (in years)") +
  theme_minimal() +
  ggtitle("RELATIVE VOLUME cingulate anterior") +
  theme(plot.title = element_text(hjust = 0.5))
Relative.Cingulate.anterior.Age.plot
ggsave("Relative.Cingulate.anterior.Age.plot.pdf", plot = Relative.Cingulate.anterior.Age.plot, width = 8, height = 6, units = "in", dpi = 600)  
 
 
   
 
 
  
Relative.Cingulate.middle.Age.plot <-  ggplot(Prosencephalon.relative, aes(y=Cingulate.middle, x = Age))  +
  geom_point(aes(color = Gender), size = 1.5, alpha = 1, shape = 16) +
  scale_color_manual(values = c("chartreuse4", "orangered2")) +
  geom_smooth(method='lm', alpha = 0.2, colour = "dodgerblue4", size = 0.8, weight = 0.3) +
  geom_smooth(aes(color = Gender), method='lm', se = F, alpha = 0.2, linetype = "longdash", size = 0.3, weight = 0.3) +
  stat_cor(method = "pearson", label.y = 1.75, label.x = 70, color = "dodgerblue4") +
  ylab("Relative Volume (in %)") + xlab("Age (in years)") +
  theme_minimal() +
  ggtitle("RELATIVE VOLUME cingulate middle") +
  theme(plot.title = element_text(hjust = 0.5))
Relative.Cingulate.middle.Age.plot
ggsave("Relative.Cingulate.middle.Age.plot.pdf", plot = Relative.Cingulate.middle.Age.plot, width = 8, height = 6, units = "in", dpi = 600)  
 
 
   
 
 
  
Relative.Cingulate.posterior.Age.plot <-  ggplot(Prosencephalon.relative, aes(y=Cingulate.posterior, x = Age))  +
  geom_point(aes(color = Gender), size = 1.5, alpha = 1, shape = 16) +
  scale_color_manual(values = c("chartreuse4", "orangered2")) +
  geom_smooth(method='lm', alpha = 0.2, colour = "dodgerblue4", size = 0.8, weight = 0.3) +
  geom_smooth(aes(color = Gender), method='lm', se = F, alpha = 0.2, linetype = "longdash", size = 0.3, weight = 0.3) +
  stat_cor(method = "pearson", label.y = 2.05, label.x = 70, color = "dodgerblue4") +
  ylab("Relative Volume (in %)") + xlab("Age (in years)") +
  theme_minimal() +
  ggtitle("RELATIVE VOLUME cingulate posterior") +
  theme(plot.title = element_text(hjust = 0.5))
Relative.Cingulate.posterior.Age.plot
ggsave("Relative.Cingulate.posterior.Age.plot.pdf", plot = Relative.Cingulate.posterior.Age.plot, width = 8, height = 6, units = "in", dpi = 600)  
 
 
   
 
 
  
Relative.PHG.Age.plot <-  ggplot(Prosencephalon.relative, aes(y=PHG, x = Age))  +
  geom_point(aes(color = Gender), size = 1.5, alpha = 1, shape = 16) +
  scale_color_manual(values = c("chartreuse4", "orangered2")) +
  geom_smooth(method='lm', alpha = 0.2, colour = "dodgerblue4", size = 0.8, weight = 0.3) +
  geom_smooth(aes(color = Gender), method='lm', se = F, alpha = 0.2, linetype = "longdash", size = 0.3, weight = 0.3) +
  stat_cor(method = "pearson", label.y = 1.15, label.x = 70, color = "dodgerblue4") +
  ylab("Relative Volume (in %)") + xlab("Age (in years)") +
  theme_minimal() +
  ggtitle("RELATIVE VOLUME PHG") +
  theme(plot.title = element_text(hjust = 0.5))
Relative.PHG.Age.plot
ggsave("Relative.PHG.Age.plot.pdf", plot = Relative.PHG.Age.plot, width = 8, height = 6, units = "in", dpi = 600)  
 
 
   
 
 
  
Relative.Hippocampus.Age.plot <-  ggplot(Prosencephalon.relative, aes(y=Hippocampus, x = Age))  +
  geom_point(aes(color = Gender), size = 1.5, alpha = 1, shape = 16) +
  scale_color_manual(values = c("chartreuse4", "orangered2")) +
  geom_smooth(method='lm', alpha = 0.2, colour = "dodgerblue4", size = 0.8, weight = 0.3) +
  geom_smooth(aes(color = Gender), method='lm', se = F, alpha = 0.2, linetype = "longdash", size = 0.5, weight = 0.3) +
  stat_cor(method = "pearson", label.y = 0.77, label.x = 70, color = "dodgerblue4") +
  ylab("Relative Volume (in %)") + xlab("Age (in years)") +
  theme_minimal() +
  ggtitle("RELATIVE VOLUME hippocampus") +
  theme(plot.title = element_text(hjust = 0.5))
Relative.Hippocampus.Age.plot
ggsave("Relative.Hippocampus.Age.plot.pdf", plot = Relative.Hippocampus.Age.plot, width = 8, height = 6, units = "in", dpi = 600)  
 
 
   
 
 
  
Relative.Amygdala.Age.plot <-  ggplot(Prosencephalon.relative, aes(y=Amygdala, x = Age))  +
  geom_point(aes(color = Gender), size = 1.5, alpha = 1, shape = 16) +
  scale_color_manual(values = c("chartreuse4", "orangered2")) +
  geom_smooth(method='lm', alpha = 0.2, colour = "dodgerblue4", size = 0.8, weight = 0.3) +
  geom_smooth(aes(color = Gender), method='lm', se = F, alpha = 0.2, linetype = "longdash", size = 0.3, weight = 0.3) +
  stat_cor(method = "pearson", label.y = 0.335, label.x = 70, color = "dodgerblue4") +
  ylab("Relative Volume (in %)") + xlab("Age (in years)") +
  theme_minimal() +
  ggtitle("RELATIVE VOLUME amygdala") +
  theme(plot.title = element_text(hjust = 0.5))
Relative.Amygdala.Age.plot
ggsave("Relative.Amygdala.Age.plot.pdf", plot = Relative.Amygdala.Age.plot, width = 8, height = 6, units = "in", dpi = 600)  
 
 
   
 
 
  
Relative.Corpus.callosum.Age.plot <-  ggplot(Prosencephalon.relative, aes(y=Corpus.callosum, x = Age))  +
  geom_point(aes(color = Gender), size = 1.5, alpha = 1, shape = 16) +
  scale_color_manual(values = c("chartreuse4", "orangered2")) +
  geom_smooth(method='lm', alpha = 0.2, colour = "dodgerblue4", size = 0.8, weight = 0.3) +
  geom_smooth(aes(color = Gender), method='lm', se = F, alpha = 0.2, linetype = "longdash", size = 0.3, weight = 0.3) +
  stat_cor(method = "pearson", label.y = 0.36, label.x = 70, color = "dodgerblue4") +
  ylab("Relative Volume (in %)") + xlab("Age (in years)") +
  theme_minimal() +
  ggtitle("RELATIVE VOLUME corpus callosum") +
  theme(plot.title = element_text(hjust = 0.5))
Relative.Corpus.callosum.Age.plot
ggsave("Relative.Corpus.callosum.Age.plot.pdf", plot = Relative.Corpus.callosum.Age.plot, width = 8, height = 6, units = "in", dpi = 600)  
 
 
   
 
 
  
Relative.Claustrum.Age.plot <-  ggplot(Prosencephalon.relative, aes(y=Claustrum, x = Age))  +
  geom_point(aes(color = Gender), size = 1.5, alpha = 1, shape = 16) +
  scale_color_manual(values = c("chartreuse4", "orangered2")) +
  geom_smooth(method='lm', alpha = 0.2, colour = "dodgerblue4", size = 0.8, weight = 0.3) +
  geom_smooth(aes(color = Gender), method='lm', se = F, alpha = 0.2, linetype = "longdash", size = 0.3, weight = 0.3) +
  stat_cor(method = "pearson", label.y = 0.18, label.x = 70, color = "dodgerblue4") +
  ylab("Relative Volume (in %)") + xlab("Age (in years)") +
  theme_minimal() +
  ggtitle("RELATIVE VOLUME claustrum") +
  theme(plot.title = element_text(hjust = 0.5))
Relative.Claustrum.Age.plot
ggsave("Relative.Claustrum.Age.plot.pdf", plot = Relative.Claustrum.Age.plot, width = 8, height = 6, units = "in", dpi = 600)  
 
 
   
 
 
  
Relative.Putamen.Age.plot <-  ggplot(Prosencephalon.relative, aes(y=Putamen, x = Age))  +
  geom_point(aes(color = Gender), size = 1.5, alpha = 1, shape = 16) +
  scale_color_manual(values = c("chartreuse4", "orangered2")) +
  geom_smooth(method='lm', alpha = 0.2, colour = "dodgerblue4", size = 0.8, weight = 0.3) +
  geom_smooth(aes(color = Gender), method='lm', se = F, alpha = 0.2, linetype = "longdash", size = 0.3, weight = 0.3) +
  stat_cor(method = "pearson", label.y = 1.15, label.x = 70, color = "dodgerblue4") +
  ylab("Relative Volume (in %)") + xlab("Age (in years)") +
  theme_minimal() +
  ggtitle("RELATIVE VOLUME putamen") +
  theme(plot.title = element_text(hjust = 0.5))
Relative.Putamen.Age.plot
ggsave("Relative.Putamen.Age.plot.pdf", plot = Relative.Putamen.Age.plot, width = 8, height = 6, units = "in", dpi = 600)  
 
 
   
 
 
  
Relative.Caudate.Age.plot <-  ggplot(Prosencephalon.relative, aes(y=Caudate, x = Age))  +
  geom_point(aes(color = Gender), size = 1.5, alpha = 1, shape = 16) +
  scale_color_manual(values = c("chartreuse4", "orangered2")) +
  geom_smooth(method='lm', alpha = 0.2, colour = "dodgerblue4", size = 0.8, weight = 0.3) +
  geom_smooth(aes(color = Gender), method='lm', se = F, alpha = 0.2, linetype = "longdash", size = 0.3, weight = 0.3) +
  stat_cor(method = "pearson", label.y = 0.87, label.x = 70, color = "dodgerblue4") +
  ylab("Relative Volume (in %)") + xlab("Age (in years)") +
  theme_minimal() +
  ggtitle("RELATIVE VOLUME caudate") +
  theme(plot.title = element_text(hjust = 0.5))
Relative.Caudate.Age.plot
ggsave("Relative.Caudate.Age.plot.pdf", plot = Relative.Caudate.Age.plot, width = 8, height = 6, units = "in", dpi = 600)  
 
 
   
 
 
  
Relative.Globus.pallidum.Age.plot <-  ggplot(Prosencephalon.relative, aes(y=Globus.pallidum, x = Age))  +
  geom_point(aes(color = Gender), size = 1.5, alpha = 1, shape = 16) +
  scale_color_manual(values = c("chartreuse4", "orangered2")) +
  geom_smooth(method='lm', alpha = 0.2, colour = "dodgerblue4", size = 0.8, weight = 0.3) +
  geom_smooth(aes(color = Gender), method='lm', se = F, alpha = 0.2, linetype = "longdash", size = 0.3, weight = 0.3) +
  stat_cor(method = "pearson", label.y = 0.335, label.x = 70, color = "dodgerblue4") +
  ylab("Relative Volume (in %)") + xlab("Age (in years)") +
  theme_minimal() +
  ggtitle("RELATIVE VOLUME globus pallidum") +
  theme(plot.title = element_text(hjust = 0.5))
Relative.Globus.pallidum.Age.plot
ggsave("Relative.Globus.pallidum.Age.plot.pdf", plot = Relative.Globus.pallidum.Age.plot, width = 8, height = 6, units = "in", dpi = 600)  
 
 
   
 
 
  
Relative.Internal.capsule.Age.plot <-  ggplot(Prosencephalon.relative, aes(y=Internal.capsule, x = Age))  +
  geom_point(aes(color = Gender), size = 1.5, alpha = 1, shape = 16) +
  scale_color_manual(values = c("chartreuse4", "orangered2")) +
  geom_smooth(method='lm', alpha = 0.2, colour = "dodgerblue4", size = 0.8, weight = 0.3) +
  geom_smooth(aes(color = Gender), method='lm', se = F, alpha = 0.2, linetype = "longdash", size = 0.3, weight = 0.3) +
  stat_cor(method = "pearson", label.y = 1.125, label.x = 70, color = "dodgerblue4") +
  ylab("Relative Volume (in %)") + xlab("Age (in years)") +
  theme_minimal() +
  ggtitle("RELATIVE VOLUME internal capsule") +
  theme(plot.title = element_text(hjust = 0.5))
Relative.Internal.capsule.Age.plot
ggsave("Relative.Internal.capsule.Age.plot.pdf", plot = Relative.Internal.capsule.Age.plot, width = 8, height = 6, units = "in", dpi = 600)  
 
 
   
 
 
  
Relative.Innominate.substance.Age.plot <-  ggplot(Prosencephalon.relative, aes(y=Innominate.substance, x = Age))  +
  geom_point(aes(color = Gender), size = 1.5, alpha = 1, shape = 16) +
  scale_color_manual(values = c("chartreuse4", "orangered2")) +
  geom_smooth(method='lm', alpha = 0.2, colour = "dodgerblue4", size = 0.8, weight = 0.3) +
  geom_smooth(aes(color = Gender), method='lm', se = F, alpha = 0.2, linetype = "longdash", size = 0.3, weight = 0.3) +
  stat_cor(method = "pearson", label.y = 0.31, label.x = 70, color = "dodgerblue4") +
  ylab("Relative Volume (in %)") + xlab("Age (in years)") +
  theme_minimal() +
  ggtitle("RELATIVE VOLUME innominate substance") +
  theme(plot.title = element_text(hjust = 0.5))
Relative.Innominate.substance.Age.plot
ggsave("Relative.Innominate.substance.Age.plot.pdf", plot = Relative.Innominate.substance.Age.plot, width = 8, height = 6, units = "in", dpi = 600)  
 
 
   
 
 
  
Relative.Hypothalamus.Age.plot <-  ggplot(Prosencephalon.relative, aes(y=Hypothalamus, x = Age))  +
  geom_point(aes(color = Gender), size = 1.5, alpha = 1, shape = 16) +
  scale_color_manual(values = c("chartreuse4", "orangered2")) +
  geom_smooth(method='lm', alpha = 0.2, colour = "dodgerblue4", size = 0.8, weight = 0.3) +
  geom_smooth(aes(color = Gender), method='lm', se = F, alpha = 0.2, linetype = "longdash", size = 0.3, weight = 0.3) +
  stat_cor(method = "pearson", label.y = 0.78, label.x = 70, color = "dodgerblue4") +
  ylab("Relative Volume (in %)") + xlab("Age (in years)") +
  theme_minimal() +
  ggtitle("RELATIVE VOLUME hypothalamus") +
  theme(plot.title = element_text(hjust = 0.5))
Relative.Hypothalamus.Age.plot
ggsave("Relative.Hypothalamus.Age.plot.pdf", plot = Relative.Hypothalamus.Age.plot, width = 8, height = 6, units = "in", dpi = 600)  
 
 
   
 
 
  
Relative.Thalamus.Age.plot <-  ggplot(Prosencephalon.relative, aes(y=Thalamus, x = Age))  +
  geom_point(aes(color = Gender), size = 1.5, alpha = 1, shape = 16) +
  scale_color_manual(values = c("chartreuse4", "orangered2")) +
  geom_smooth(method='lm', alpha = 0.2, colour = "dodgerblue4", size = 0.8, weight = 0.3) +
  geom_smooth(aes(color = Gender), method='lm', se = F, alpha = 0.2, linetype = "longdash", size = 0.3, weight = 0.3) +
  stat_cor(method = "pearson", label.y = 1.47, label.x = 70, color = "dodgerblue4") +
  ylab("Relative Volume (in %)") + xlab("Age (in years)") +
  theme_minimal() +
  ggtitle("RELATIVE VOLUME thalamus") +
  theme(plot.title = element_text(hjust = 0.5))
Relative.Thalamus.Age.plot
ggsave("Relative.Thalamus.Age.plot.pdf", plot = Relative.Thalamus.Age.plot, width = 8, height = 6, units = "in", dpi = 600)
  
 
 
   
 
 
 
 
 
 
 Brainstem and Cerebellum 
 
 Absolute Volumes 
 
 
 
  
Mesencephalon <- All.Volumes$Mesencephalon
Pons <- All.Volumes$Pons
Medulla.oblongata <- All.Volumes$`Medulla oblongata`
  
Cerebellar.peduncles <- All.Volumes$`Total volume cerebellar peduncles`

Vermis <- All.Volumes$`Total volume vermis`
Hemisphere <- All.Volumes$`Total volume cerebellar hemisphere`

Anterior.lobe <- (All.Volumes$Central+All.Volumes$Culmen+All.Volumes$`Total volume ala lobuli centralis`+All.Volumes$`Total volume AQL`)
Medial.lobe <- (All.Volumes$Declive+All.Volumes$Folium+All.Volumes$`Total volume PQL`+All.Volumes$`Total volume SSL`)
Posterior.lobe <- (All.Volumes$Tuber+All.Volumes$Pyramid+All.Volumes$Uvula+All.Volumes$`Total volume inferior semilunar /gracile`+All.Volumes$`Total volume biventer`+All.Volumes$`Total volume tonsilla`)
Flocculonodular.lobe <- (All.Volumes$Nodule+All.Volumes$`Total volume flocculus`) 

Central <- All.Volumes$Central
Culmen <- All.Volumes$Culmen
Declive <- All.Volumes$Declive
Folium <- All.Volumes$Folium
Tuber <- All.Volumes$Tuber
Pyramid <- All.Volumes$Pyramid
Uvula <- All.Volumes$Uvula
Nodule <- All.Volumes$Nodule

Ala.lobuli.centralis <- All.Volumes$`Total volume ala lobuli centralis`
AQL <- All.Volumes$`Total volume AQL`
PQL <- All.Volumes$`Total volume PQL`
SSL <-  All.Volumes$`Total volume SSL`
ISL.gracile <- All.Volumes$`Total volume inferior semilunar /gracile`
Biventer <- All.Volumes$`Total volume biventer`
Tonsilla <- All.Volumes$`Total volume tonsilla`
Flocculus <- All.Volumes$`Total volume flocculus`

Brainstem.Cerebellum.absolute <- as.data.frame(cbind(
Mesencephalon,
Pons,
Medulla.oblongata, 
Cerebellar.peduncles, 
Vermis, 
Hemisphere, 
Anterior.lobe, 
Medial.lobe, 
Posterior.lobe, 
Flocculonodular.lobe, 
Central, 
Culmen, 
Declive, 
Folium, 
Tuber,
Pyramid, 
Uvula, 
Nodule,
Ala.lobuli.centralis,
AQL,
PQL,
SSL,
ISL.gracile, 
Biventer,
Tonsilla, 
Flocculus 
))

Brainstem.Cerebellum.absolute$Gender <- All.Volumes$Gender

Table.Brainstem.Cerebellum.absolute <- CreateTableOne(
  vars = c("Mesencephalon",
"Pons",
"Medulla.oblongata", 
"Cerebellar.peduncles", 
"Vermis", 
"Hemisphere", 
"Anterior.lobe", 
"Medial.lobe", 
"Posterior.lobe", 
"Flocculonodular.lobe", 
"Central", 
"Culmen", 
"Declive", 
"Folium", 
"Tuber",
"Pyramid", 
"Uvula", 
"Nodule",
"Ala.lobuli.centralis",
"AQL",
"PQL",
"SSL",
"ISL.gracile", 
"Biventer",
"Tonsilla", 
"Flocculus"),
  data = Brainstem.Cerebellum.absolute)

Table.Brainstem.Cerebellum.absolute.stratified.gender <- CreateTableOne(
  vars = c("Mesencephalon",
"Pons",
"Medulla.oblongata", 
"Cerebellar.peduncles", 
"Vermis", 
"Hemisphere", 
"Anterior.lobe", 
"Medial.lobe", 
"Posterior.lobe", 
"Flocculonodular.lobe", 
"Central", 
"Culmen", 
"Declive", 
"Folium", 
"Tuber",
"Pyramid", 
"Uvula", 
"Nodule",
"Ala.lobuli.centralis",
"AQL",
"PQL",
"SSL",
"ISL.gracile", 
"Biventer",
"Tonsilla", 
"Flocculus"), 
strata = c("Gender"),
data = Brainstem.Cerebellum.absolute)

Table.Brainstem.Cerebellum.absolute <- print(Table.Brainstem.Cerebellum.absolute, contDigits = 10)  
 
 
                                    
                                   Overall                             
  n                                               30                   
  Mesencephalon (mean (SD))         10045.5666666667 (1460.5845472071) 
  Pons (mean (SD))                  15286.1333333333 (2216.2026505953) 
  Medulla.oblongata (mean (SD))      2740.8333333333 (367.7611746898)  
  Cerebellar.peduncles (mean (SD))   6780.7000000000 (962.4036413925)  
  Vermis (mean (SD))                 5946.8333333333 (804.8236938367)  
  Hemisphere (mean (SD))           110786.1333333333 (12111.5249865098)
  Anterior.lobe (mean (SD))         32232.8666666667 (4775.6684076923) 
  Medial.lobe (mean (SD))           28791.7000000000 (4414.8219407996) 
  Posterior.lobe (mean (SD))        54644.6666666667 (7773.0127844438) 
  Flocculonodular.lobe (mean (SD))   1063.7000000000 (150.3644193973)  
  Central (mean (SD))                 481.9666666667 (125.1868901733)  
  Culmen (mean (SD))                 2183.6000000000 (359.9041634888)  
  Declive (mean (SD))                1030.5333333333 (188.4987392866)  
  Folium (mean (SD))                  440.1000000000 (120.4701565563)  
  Tuber (mean (SD))                   387.0333333333 (97.0319831145)   
  Pyramid (mean (SD))                 426.6333333333 (201.7137752521)  
  Uvula (mean (SD))                   784.6666666667 (129.0771844294)  
  Nodule (mean (SD))                  212.5000000000 (35.1290723495)   
  Ala.lobuli.centralis (mean (SD))  11993.7666666667 (2880.0725647388) 
  AQL (mean (SD))                   17573.5333333333 (3822.1072607455) 
  PQL (mean (SD))                   13124.4333333333 (2598.2643767070) 
  SSL (mean (SD))                   14196.6333333333 (2513.7768875200) 
  ISL.gracile (mean (SD))           34930.4333333333 (6197.6310695872) 
  Biventer (mean (SD))              12163.3000000000 (3786.6815073268) 
  Tonsilla (mean (SD))               5952.6000000000 (1337.6721571446) 
  Flocculus (mean (SD))               851.2000000000 (139.2406500297)    
 
 
  Table.Brainstem.Cerebellum.absolute.stratified.gender <- print(Table.Brainstem.Cerebellum.absolute.stratified.gender, contDigits = 10)  
 
 
                                    Stratified by Gender
                                   f                                    m                                    p      test
  n                                               14                                   16                               
  Mesencephalon (mean (SD))          9763.7857142857 (1207.3040645983)   10292.1250000000 (1649.9104167601)   0.332     
  Pons (mean (SD))                  14388.7142857143 (2400.8001688082)   16071.3750000000 (1758.1127713166)   0.036     
  Medulla.oblongata (mean (SD))      2654.9285714286 (273.5303285914)     2816.0000000000 (428.6288215539)    0.238     
  Cerebellar.peduncles (mean (SD))   6442.8571428571 (696.4864411009)     7076.3125000000 (1081.9140889337)   0.071     
  Vermis (mean (SD))                 5729.2857142857 (698.0237408979)     6137.1875000000 (864.4103361059)    0.170     
  Hemisphere (mean (SD))           105887.2142857143 (12510.9148234564) 115072.6875000000 (10293.0357214235)  0.036     
  Anterior.lobe (mean (SD))         30952.2142857143 (5066.7219440425)   33353.4375000000 (4355.9895617988)   0.174     
  Medial.lobe (mean (SD))           28501.7142857143 (4694.7263282010)   29045.4375000000 (4293.3716660103)   0.743     
  Posterior.lobe (mean (SD))        51123.5000000000 (7944.6905807009)   57725.6875000000 (6357.0069447684)   0.017     
  Flocculonodular.lobe (mean (SD))   1039.6428571429 (171.7814385705)     1084.7500000000 (130.8604855052)    0.422     
  Central (mean (SD))                 494.1428571429 (138.8357397696)      471.3125000000 (115.4727781774)    0.627     
  Culmen (mean (SD))                 2090.3571428571 (300.4288235539)     2265.1875000000 (396.2178220373)    0.189     
  Declive (mean (SD))                1009.5000000000 (222.2752105629)     1048.9375000000 (158.4354626759)    0.577     
  Folium (mean (SD))                  429.9285714286 (104.0461911370)      449.0000000000 (135.9975489975)    0.673     
  Tuber (mean (SD))                   359.0714285714 (74.5442194898)       411.5000000000 (109.6290715702)    0.143     
  Pyramid (mean (SD))                 368.1428571429 (137.8856031660)      477.8125000000 (237.0651721222)    0.140     
  Uvula (mean (SD))                   768.3571428571 (121.3416075148)      798.9375000000 (137.7860509389)    0.527     
  Nodule (mean (SD))                  210.4285714286 (32.6937168410)       214.3125000000 (38.1046257034)     0.768     
  Ala.lobuli.centralis (mean (SD))  11410.7857142857 (3163.7587717672)   12503.8750000000 (2601.3639210999)   0.308     
  AQL (mean (SD))                   16956.9285714286 (3679.3877096037)   18113.0625000000 (3980.5670026392)   0.418     
  PQL (mean (SD))                   12835.1428571429 (3258.6597022311)   13377.5625000000 (1924.1673513064)   0.577     
  SSL (mean (SD))                   14227.1428571429 (2163.5692325821)   14169.9375000000 (2856.2831901792)   0.952     
  ISL.gracile (mean (SD))           33388.7857142857 (6276.2341866707)   36279.3750000000 (5996.8659134029)   0.208     
  Biventer (mean (SD))              10529.6428571429 (3581.7178217924)   13592.7500000000 (3454.4626113671)   0.024     
  Tonsilla (mean (SD))               5709.5000000000 (1079.5718346427)    6165.3125000000 (1531.6471620992)   0.361     
  Flocculus (mean (SD))               829.2142857143 (158.8619035376)      870.4375000000 (121.5126708071)    0.428       
 
 
  write.csv(Table.Brainstem.Cerebellum.absolute, "Table.Brainstem.Cerebellum.absolute.csv")
write.csv(Table.Brainstem.Cerebellum.absolute.stratified.gender, "Table.Brainstem.Cerebellum.absolute.stratified.gender.csv")
  
 
 
 
 
 
 
  
Table.Brainstem.Cerebellum.absolute.RSD <- as.data.frame(Table.Brainstem.Cerebellum.absolute)
Table.Brainstem.Cerebellum.absolute.RSD <- data.frame(do.call('rbind', 
                                                              strsplit(as.character(Table.Brainstem.Cerebellum.absolute.RSD[-1,]),' (',fixed=TRUE)))
Table.Brainstem.Cerebellum.absolute.RSD <- data.frame(cbind(str_replace_all(Table.Brainstem.Cerebellum.absolute.RSD$X1, "[ ]", ""),
                                                            str_replace_all(Table.Brainstem.Cerebellum.absolute.RSD$X2, "[)]", "")))
Table.Brainstem.Cerebellum.absolute.RSD$X1 <- as.character(Table.Brainstem.Cerebellum.absolute.RSD$X1)
Table.Brainstem.Cerebellum.absolute.RSD$X2 <- as.character(Table.Brainstem.Cerebellum.absolute.RSD$X2)
Table.Brainstem.Cerebellum.absolute.RSD <- as.data.frame(sapply(Table.Brainstem.Cerebellum.absolute.RSD, as.numeric))
Table.Brainstem.Cerebellum.absolute.RSD <- as.data.frame(Table.Brainstem.Cerebellum.absolute.RSD$X2/Table.Brainstem.Cerebellum.absolute.RSD$X1)
Table.Brainstem.Cerebellum.absolute.RSD <- round(Table.Brainstem.Cerebellum.absolute.RSD * 100, 1)


Table.Prosencephalon.absolute.stratified.gender.RSD <- as.data.frame(Table.Brainstem.Cerebellum.absolute.stratified.gender)
Table.Prosencephalon.absolute.stratified.gender.RSD <- select(Table.Prosencephalon.absolute.stratified.gender.RSD, - c(p, test))

Table.Prosencephalon.absolute.stratified.gender.RSD.female <- data.frame(do.call('rbind', 
                                                              strsplit(as.character(Table.Prosencephalon.absolute.stratified.gender.RSD[-1, "f"]),
                                                                       ' (',fixed=TRUE)))
Table.Prosencephalon.absolute.stratified.gender.RSD.female <- data.frame(cbind(str_replace_all(Table.Prosencephalon.absolute.stratified.gender.RSD.female$X1, "[ ]", ""),
                                                            str_replace_all(Table.Prosencephalon.absolute.stratified.gender.RSD.female$X2, "[)]", "")))
Table.Prosencephalon.absolute.stratified.gender.RSD.female$X1 <- as.character(Table.Prosencephalon.absolute.stratified.gender.RSD.female$X1)
Table.Prosencephalon.absolute.stratified.gender.RSD.female$X2 <- as.character(Table.Prosencephalon.absolute.stratified.gender.RSD.female$X2)
Table.Prosencephalon.absolute.stratified.gender.RSD.female <- as.data.frame(sapply(Table.Prosencephalon.absolute.stratified.gender.RSD.female, as.numeric))
Table.Prosencephalon.absolute.stratified.gender.RSD.female <- as.data.frame(Table.Prosencephalon.absolute.stratified.gender.RSD.female$X2/Table.Prosencephalon.absolute.stratified.gender.RSD.female$X1)
Table.Prosencephalon.absolute.stratified.gender.RSD.female <- round(Table.Prosencephalon.absolute.stratified.gender.RSD.female * 100, 1)

Table.Prosencephalon.absolute.stratified.gender.RSD.male <- data.frame(do.call('rbind', 
                                                              strsplit(as.character(Table.Prosencephalon.absolute.stratified.gender.RSD[-1, "m"]),
                                                                       ' (',fixed=TRUE)))
Table.Prosencephalon.absolute.stratified.gender.RSD.male <- data.frame(cbind(str_replace_all(Table.Prosencephalon.absolute.stratified.gender.RSD.male$X1, "[ ]", ""),
                                                            str_replace_all(Table.Prosencephalon.absolute.stratified.gender.RSD.male$X2, "[)]", "")))
Table.Prosencephalon.absolute.stratified.gender.RSD.male$X1 <- as.character(Table.Prosencephalon.absolute.stratified.gender.RSD.male$X1)
Table.Prosencephalon.absolute.stratified.gender.RSD.male$X2 <- as.character(Table.Prosencephalon.absolute.stratified.gender.RSD.male$X2)
Table.Prosencephalon.absolute.stratified.gender.RSD.male <- as.data.frame(sapply(Table.Prosencephalon.absolute.stratified.gender.RSD.male, as.numeric))
Table.Prosencephalon.absolute.stratified.gender.RSD.male <- as.data.frame(Table.Prosencephalon.absolute.stratified.gender.RSD.male$X2/Table.Prosencephalon.absolute.stratified.gender.RSD.male$X1)
Table.Prosencephalon.absolute.stratified.gender.RSD.male <- round(Table.Prosencephalon.absolute.stratified.gender.RSD.male * 100, 1)
  
 
 
 
 
 
 
  
kable(Table.Brainstem.Cerebellum.absolute)  
 
 
 

 
 
 
  
 Overall 
 
 
 
 
 n 
 30 
 
 
 Mesencephalon (mean (SD)) 
 10045.5666666667 (1460.5845472071) 
 
 
 Pons (mean (SD)) 
 15286.1333333333 (2216.2026505953) 
 
 
 Medulla.oblongata (mean (SD)) 
 2740.8333333333 (367.7611746898) 
 
 
 Cerebellar.peduncles (mean (SD)) 
 6780.7000000000 (962.4036413925) 
 
 
 Vermis (mean (SD)) 
 5946.8333333333 (804.8236938367) 
 
 
 Hemisphere (mean (SD)) 
 110786.1333333333 (12111.5249865098) 
 
 
 Anterior.lobe (mean (SD)) 
 32232.8666666667 (4775.6684076923) 
 
 
 Medial.lobe (mean (SD)) 
 28791.7000000000 (4414.8219407996) 
 
 
 Posterior.lobe (mean (SD)) 
 54644.6666666667 (7773.0127844438) 
 
 
 Flocculonodular.lobe (mean (SD)) 
 1063.7000000000 (150.3644193973) 
 
 
 Central (mean (SD)) 
 481.9666666667 (125.1868901733) 
 
 
 Culmen (mean (SD)) 
 2183.6000000000 (359.9041634888) 
 
 
 Declive (mean (SD)) 
 1030.5333333333 (188.4987392866) 
 
 
 Folium (mean (SD)) 
 440.1000000000 (120.4701565563) 
 
 
 Tuber (mean (SD)) 
 387.0333333333 (97.0319831145) 
 
 
 Pyramid (mean (SD)) 
 426.6333333333 (201.7137752521) 
 
 
 Uvula (mean (SD)) 
 784.6666666667 (129.0771844294) 
 
 
 Nodule (mean (SD)) 
 212.5000000000 (35.1290723495) 
 
 
 Ala.lobuli.centralis (mean (SD)) 
 11993.7666666667 (2880.0725647388) 
 
 
 AQL (mean (SD)) 
 17573.5333333333 (3822.1072607455) 
 
 
 PQL (mean (SD)) 
 13124.4333333333 (2598.2643767070) 
 
 
 SSL (mean (SD)) 
 14196.6333333333 (2513.7768875200) 
 
 
 ISL.gracile (mean (SD)) 
 34930.4333333333 (6197.6310695872) 
 
 
 Biventer (mean (SD)) 
 12163.3000000000 (3786.6815073268) 
 
 
 Tonsilla (mean (SD)) 
 5952.6000000000 (1337.6721571446) 
 
 
 Flocculus (mean (SD)) 
 851.2000000000 (139.2406500297) 
 
 
 


 
   
  kable(Table.Brainstem.Cerebellum.absolute.RSD)  
 
 
 

 
 
 
 
 
 
 Table.Brainstem.Cerebellum.absolute.RSD  X 2/ T  a  b  l  e . B  r  a  i  n  s  t  e  m . C  e  r  e  b  e  l  l  u  m . a  b  s  o  l  u  t  e . R  S  D  X1 
 
 
 
 
 14.5 
 
 
 14.5 
 
 
 13.4 
 
 
 14.2 
 
 
 13.5 
 
 
 10.9 
 
 
 14.8 
 
 
 15.3 
 
 
 14.2 
 
 
 14.1 
 
 
 26.0 
 
 
 16.5 
 
 
 18.3 
 
 
 27.4 
 
 
 25.1 
 
 
 47.3 
 
 
 16.4 
 
 
 16.5 
 
 
 24.0 
 
 
 21.7 
 
 
 19.8 
 
 
 17.7 
 
 
 17.7 
 
 
 31.1 
 
 
 22.5 
 
 
 16.4 
 
 
 


 
   
  kable(Table.Brainstem.Cerebellum.absolute.stratified.gender)  
 
 
 

 
 
 
 
 
 
 
 
 
 
  
 f 
 m 
 p 
 test 
 
 
 
 
 n 
 14 
 16 
  
  
 
 
 Mesencephalon (mean (SD)) 
 9763.7857142857 (1207.3040645983) 
 10292.1250000000 (1649.9104167601) 
 0.332 
  
 
 
 Pons (mean (SD)) 
 14388.7142857143 (2400.8001688082) 
 16071.3750000000 (1758.1127713166) 
 0.036 
  
 
 
 Medulla.oblongata (mean (SD)) 
 2654.9285714286 (273.5303285914) 
 2816.0000000000 (428.6288215539) 
 0.238 
  
 
 
 Cerebellar.peduncles (mean (SD)) 
 6442.8571428571 (696.4864411009) 
 7076.3125000000 (1081.9140889337) 
 0.071 
  
 
 
 Vermis (mean (SD)) 
 5729.2857142857 (698.0237408979) 
 6137.1875000000 (864.4103361059) 
 0.170 
  
 
 
 Hemisphere (mean (SD)) 
 105887.2142857143 (12510.9148234564) 
 115072.6875000000 (10293.0357214235) 
 0.036 
  
 
 
 Anterior.lobe (mean (SD)) 
 30952.2142857143 (5066.7219440425) 
 33353.4375000000 (4355.9895617988) 
 0.174 
  
 
 
 Medial.lobe (mean (SD)) 
 28501.7142857143 (4694.7263282010) 
 29045.4375000000 (4293.3716660103) 
 0.743 
  
 
 
 Posterior.lobe (mean (SD)) 
 51123.5000000000 (7944.6905807009) 
 57725.6875000000 (6357.0069447684) 
 0.017 
  
 
 
 Flocculonodular.lobe (mean (SD)) 
 1039.6428571429 (171.7814385705) 
 1084.7500000000 (130.8604855052) 
 0.422 
  
 
 
 Central (mean (SD)) 
 494.1428571429 (138.8357397696) 
 471.3125000000 (115.4727781774) 
 0.627 
  
 
 
 Culmen (mean (SD)) 
 2090.3571428571 (300.4288235539) 
 2265.1875000000 (396.2178220373) 
 0.189 
  
 
 
 Declive (mean (SD)) 
 1009.5000000000 (222.2752105629) 
 1048.9375000000 (158.4354626759) 
 0.577 
  
 
 
 Folium (mean (SD)) 
 429.9285714286 (104.0461911370) 
 449.0000000000 (135.9975489975) 
 0.673 
  
 
 
 Tuber (mean (SD)) 
 359.0714285714 (74.5442194898) 
 411.5000000000 (109.6290715702) 
 0.143 
  
 
 
 Pyramid (mean (SD)) 
 368.1428571429 (137.8856031660) 
 477.8125000000 (237.0651721222) 
 0.140 
  
 
 
 Uvula (mean (SD)) 
 768.3571428571 (121.3416075148) 
 798.9375000000 (137.7860509389) 
 0.527 
  
 
 
 Nodule (mean (SD)) 
 210.4285714286 (32.6937168410) 
 214.3125000000 (38.1046257034) 
 0.768 
  
 
 
 Ala.lobuli.centralis (mean (SD)) 
 11410.7857142857 (3163.7587717672) 
 12503.8750000000 (2601.3639210999) 
 0.308 
  
 
 
 AQL (mean (SD)) 
 16956.9285714286 (3679.3877096037) 
 18113.0625000000 (3980.5670026392) 
 0.418 
  
 
 
 PQL (mean (SD)) 
 12835.1428571429 (3258.6597022311) 
 13377.5625000000 (1924.1673513064) 
 0.577 
  
 
 
 SSL (mean (SD)) 
 14227.1428571429 (2163.5692325821) 
 14169.9375000000 (2856.2831901792) 
 0.952 
  
 
 
 ISL.gracile (mean (SD)) 
 33388.7857142857 (6276.2341866707) 
 36279.3750000000 (5996.8659134029) 
 0.208 
  
 
 
 Biventer (mean (SD)) 
 10529.6428571429 (3581.7178217924) 
 13592.7500000000 (3454.4626113671) 
 0.024 
  
 
 
 Tonsilla (mean (SD)) 
 5709.5000000000 (1079.5718346427) 
 6165.3125000000 (1531.6471620992) 
 0.361 
  
 
 
 Flocculus (mean (SD)) 
 829.2142857143 (158.8619035376) 
 870.4375000000 (121.5126708071) 
 0.428 
  
 
 
 


 
   
  kable(Table.Prosencephalon.absolute.stratified.gender.RSD.female)  
 
 
 

 
 
 
 
 
 
 Table.Prosencephalon.absolute.stratified.gender.RSD.female  X 2/ T  a  b  l  e . P  r  o  s  e  n  c  e  p  h  a  l  o  n . a  b  s  o  l  u  t  e . s  t  r  a  t  i  f  i  e  d . g  e  n  d  e  r . R  S  D . f  e  m  a  l  e  X1 
 
 
 
 
 12.4 
 
 
 16.7 
 
 
 10.3 
 
 
 10.8 
 
 
 12.2 
 
 
 11.8 
 
 
 16.4 
 
 
 16.5 
 
 
 15.5 
 
 
 16.5 
 
 
 28.1 
 
 
 14.4 
 
 
 22.0 
 
 
 24.2 
 
 
 20.8 
 
 
 37.5 
 
 
 15.8 
 
 
 15.5 
 
 
 27.7 
 
 
 21.7 
 
 
 25.4 
 
 
 15.2 
 
 
 18.8 
 
 
 34.0 
 
 
 18.9 
 
 
 19.2 
 
 
 


 
   
  kable(Table.Prosencephalon.absolute.stratified.gender.RSD.male)  
 
 
 

 
 
 
 
 
 
 Table.Prosencephalon.absolute.stratified.gender.RSD.male  X 2/ T  a  b  l  e . P  r  o  s  e  n  c  e  p  h  a  l  o  n . a  b  s  o  l  u  t  e . s  t  r  a  t  i  f  i  e  d . g  e  n  d  e  r . R  S  D . m  a  l  e  X1 
 
 
 
 
 16.0 
 
 
 10.9 
 
 
 15.2 
 
 
 15.3 
 
 
 14.1 
 
 
 8.9 
 
 
 13.1 
 
 
 14.8 
 
 
 11.0 
 
 
 12.1 
 
 
 24.5 
 
 
 17.5 
 
 
 15.1 
 
 
 30.3 
 
 
 26.6 
 
 
 49.6 
 
 
 17.2 
 
 
 17.8 
 
 
 20.8 
 
 
 22.0 
 
 
 14.4 
 
 
 20.2 
 
 
 16.5 
 
 
 25.4 
 
 
 24.8 
 
 
 14.0 
 
 
 


 
   
  NA  
 
 
 
 
 
 
  
Brainstem.Cerebellum.absolute <- select(Brainstem.Cerebellum.absolute, - c(Gender))
Brainstem.Cerebellum.absolute1 <- Brainstem.Cerebellum.absolute[,c(1:10)]
Brainstem.Cerebellum.absolute2 <- Brainstem.Cerebellum.absolute[,-c(1:10)]

names.anatomical.structures.temporary <- c("Mesencephalon",
"Pons",
"Medulla.oblongata", 
"Cerebellar.peduncles", 
"Vermis", 
"Hemisphere", 
"Anterior.lobe", 
"Medial.lobe", 
"Posterior.lobe", 
"Flocculonodular.lobe")

names.anatomical.structures.definitive <- c("Mesencephalon",
"Pons",
"Medulla oblongata", 
"Cerebellar peduncles", 
"Vermis", 
"Hemisphere", 
"Anterior lobe", 
"Medial lobe", 
"Posterior lobe", 
"Flocculonodular lobe")

Brainstem.Cerebellum.absolute.plotdata1 <- gather(Brainstem.Cerebellum.absolute1, "anatomical.structure", "relative.volume")
Brainstem.Cerebellum.absolute.plotdata1$Gender <- All.Volumes$Gender
Brainstem.Cerebellum.absolute.plotdata1$Age <- All.Volumes$`Age (years)`

Brainstem.Cerebellum.absolute.plotdata1$Gender <- factor(Brainstem.Cerebellum.absolute.plotdata1$Gender, levels = c("f", "m"), c("f", "m"))
Brainstem.Cerebellum.absolute.plotdata1$anatomical.structure <- factor(Brainstem.Cerebellum.absolute.plotdata1$anatomical.structure, 
                                                                levels = rev(c(names.anatomical.structures.temporary)), rev(c(names.anatomical.structures.definitive)))

Brainstem.Cerebellum.absolute.plot1 <-  ggplot(Brainstem.Cerebellum.absolute.plotdata1, aes(x=anatomical.structure, y = relative.volume))  +
  stat_summary(alpha = 0.3, fun = mean, geom = "bar", width = 0.3, fill = "gray50") + 
  geom_boxplot(aes(fill = Gender), alpha = 0.5, width = 0.4, size = 0.2, position = position_dodge(width = 0.6), 
               outlier.shape = NA, color = "gray30") +
  scale_fill_manual(values = c("chartreuse4", "orangered2")) +
  geom_quasirandom(aes(color = Age), size = 0.7, alpha = 0.8, shape = 16, position = "dodge") +
  scale_color_continuous(low = "steelblue1", high = "red4") +
  xlab("") + ylab("Absolute volume (in mm3)") +
  theme_minimal() +
  coord_flip() +
  ggtitle("BRAINSTEM & CEREBELLUM") +
  theme(plot.title = element_text(hjust = 0.5))

Brainstem.Cerebellum.absolute.plot1
ggsave("Brainstem.Cerebellum.absolute.plot1.pdf", plot = Brainstem.Cerebellum.absolute.plot1, width = 12, height = 6, units = "in", dpi = 600)
  
 
 
   
 
 
 
 
 
 
  
names.anatomical.structures.temporary <- c(
"Central", 
"Culmen", 
"Declive", 
"Folium", 
"Tuber",
"Pyramid", 
"Uvula", 
"Nodule",
"Ala.lobuli.centralis",
"AQL",
"PQL",
"SSL",
"ISL.gracile", 
"Biventer",
"Tonsilla", 
"Flocculus")

names.anatomical.structures.definitive <- c(
"Central", 
"Culmen", 
"Declive", 
"Folium", 
"Tuber",
"Pyramid", 
"Uvula", 
"Nodule",
"Ala lobuli centralis",
"AQL",
"PQL",
"SSL",
"ISL/gracile", 
"Biventer",
"Tonsilla", 
"Flocculus")

Brainstem.Cerebellum.absolute.plotdata2 <- gather(Brainstem.Cerebellum.absolute2, "anatomical.structure", "relative.volume")
Brainstem.Cerebellum.absolute.plotdata2$Gender <- All.Volumes$Gender
Brainstem.Cerebellum.absolute.plotdata2$Age <- All.Volumes$`Age (years)`

Brainstem.Cerebellum.absolute.plotdata2$Gender <- factor(Brainstem.Cerebellum.absolute.plotdata2$Gender, levels = c("f", "m"), c("f", "m"))
Brainstem.Cerebellum.absolute.plotdata2$anatomical.structure <- factor(Brainstem.Cerebellum.absolute.plotdata2$anatomical.structure, 
                                                                levels = rev(c(names.anatomical.structures.temporary)), rev(c(names.anatomical.structures.definitive)))

Brainstem.Cerebellum.absolute.plot2 <-  ggplot(Brainstem.Cerebellum.absolute.plotdata2, aes(x=anatomical.structure, y = relative.volume))  +
  stat_summary(alpha = 0.3, fun = mean, geom = "bar", width = 0.3, fill = "gray50") + 
  geom_boxplot(aes(fill = Gender), alpha = 0.5, width = 0.4, size = 0.2, position = position_dodge(width = 0.6), 
               outlier.shape = NA, color = "gray30") +
  scale_fill_manual(values = c("chartreuse4", "orangered2")) +
  geom_quasirandom(aes(color = Age), size = 0.7, alpha = 0.8, shape = 16, position = "dodge") +
  scale_color_continuous(low = "steelblue1", high = "red4") +
  xlab("") + ylab("Absolute volume (in mm3)") +
  theme_minimal() +
  coord_flip() +
  ggtitle("CEREBELLAR LOBES & LOBULES") +
  theme(plot.title = element_text(hjust = 0.5))

Brainstem.Cerebellum.absolute.plot2
ggsave("Brainstem.Cerebellum.absolute.plot2.pdf", plot = Brainstem.Cerebellum.absolute.plot2, width = 12, height = 6, units = "in", dpi = 600)
  
 
 
   
 
 
 
 
 
 
  
Brainstem.Cerebellum.absolute$Gender <- All.Volumes$Gender
Brainstem.Cerebellum.absolute$Age <- All.Volumes$`Age (years)`

Total.Mesencephalon.Age.plot <-  ggplot(Brainstem.Cerebellum.absolute, aes(y=Mesencephalon, x = Age))  +
  geom_point(aes(color = Gender), size = 1.5, alpha = 1, shape = 16) +
  scale_color_manual(values = c("chartreuse4", "orangered2")) +
  geom_smooth(method='lm', alpha = 0.2, colour = "dodgerblue4", size = 0.8, weight = 0.3) +
  geom_smooth(aes(color = Gender), method='lm', se = F, alpha = 0.2, linetype = "longdash", size = 0.3, weight = 0.3) +
  stat_cor(method = "pearson", label.y = 11700, label.x = 70, color = "dodgerblue4") +
  ylab("Volume in mm3") + xlab("Age (in years)") +
  theme_minimal() +
  ggtitle("ABSOLUTE VOLUME mesencephalon") +
  theme(plot.title = element_text(hjust = 0.5))
Total.Mesencephalon.Age.plot
ggsave("Total.Mesencephalon.Age.plot.pdf", plot = Total.Mesencephalon.Age.plot, width = 8, height = 6, units = "in", dpi = 600)  
 
 
   
 
 
  
Total.Pons.Age.plot <-  ggplot(Brainstem.Cerebellum.absolute, aes(y=Pons, x = Age))  +
  geom_point(aes(color = Gender), size = 1.5, alpha = 1, shape = 16) +
  scale_color_manual(values = c("chartreuse4", "orangered2")) +
  geom_smooth(method='lm', alpha = 0.2, colour = "dodgerblue4", size = 0.8, weight = 0.3) +
  geom_smooth(aes(color = Gender), method='lm', se = F, alpha = 0.2, linetype = "longdash", size = 0.3, weight = 0.3) +
  stat_cor(method = "pearson", label.y = 20500, label.x = 70, color = "dodgerblue4") +
  ylab("Volume in mm3") + xlab("Age (in years)") +
  theme_minimal() +
  ggtitle("ABSOLUTE VOLUME pons") +
  theme(plot.title = element_text(hjust = 0.5))
Total.Pons.Age.plot
ggsave("Total.Pons.Age.plot.pdf", plot = Total.Pons.Age.plot, width = 8, height = 6, units = "in", dpi = 600)  
 
 
   
 
 
  
Total.Medulla.oblongata.Age.plot <-  ggplot(Brainstem.Cerebellum.absolute, aes(y=Medulla.oblongata, x = Age))  +
  geom_point(aes(color = Gender), size = 1.5, alpha = 1, shape = 16) +
  scale_color_manual(values = c("chartreuse4", "orangered2")) +
  geom_smooth(method='lm', alpha = 0.2, colour = "dodgerblue4", size = 0.8, weight = 0.3) +
  geom_smooth(aes(color = Gender), method='lm', se = F, alpha = 0.2, linetype = "longdash", size = 0.3, weight = 0.3) +
  stat_cor(method = "pearson", label.y = 3300, label.x = 70, color = "dodgerblue4") +
  ylab("Volume in mm3") + xlab("Age (in years)") +
  theme_minimal() +
  ggtitle("ABSOLUTE VOLUME medulla oblongata") +
  theme(plot.title = element_text(hjust = 0.5))
Total.Medulla.oblongata.Age.plot
ggsave("Total.Medulla.oblongata.Age.plot.pdf", plot = Total.Medulla.oblongata.Age.plot, width = 8, height = 6, units = "in", dpi = 600)  
 
 
   
 
 
  
Total.Cerebellar.peduncles.Age.plot <-  ggplot(Brainstem.Cerebellum.absolute, aes(y=Cerebellar.peduncles, x = Age))  +
  geom_point(aes(color = Gender), size = 1.5, alpha = 1, shape = 16) +
  scale_color_manual(values = c("chartreuse4", "orangered2")) +
  geom_smooth(method='lm', alpha = 0.2, colour = "dodgerblue4", size = 0.8, weight = 0.3) +
  geom_smooth(aes(color = Gender), method='lm', se = F, alpha = 0.2, linetype = "longdash", size = 0.3, weight = 0.3) +
  stat_cor(method = "pearson", label.y = 8700, label.x = 70, color = "dodgerblue4") +
  ylab("Volume in mm3") + xlab("Age (in years)") +
  theme_minimal() +
  ggtitle("ABSOLUTE VOLUME cerebellar peduncles") +
  theme(plot.title = element_text(hjust = 0.5))
Total.Cerebellar.peduncles.Age.plot
ggsave("Total.Cerebellar.peduncles.Age.plot.pdf", plot = Total.Cerebellar.peduncles.Age.plot, width = 8, height = 6, units = "in", dpi = 600)  
 
 
   
 
 
  
Total.Vermis.Age.plot <-  ggplot(Brainstem.Cerebellum.absolute, aes(y=Vermis, x = Age))  +
  geom_point(aes(color = Gender), size = 1.5, alpha = 1, shape = 16) +
  scale_color_manual(values = c("chartreuse4", "orangered2")) +
  geom_smooth(method='lm', alpha = 0.2, colour = "dodgerblue4", size = 0.8, weight = 0.3) +
  geom_smooth(aes(color = Gender), method='lm', se = F, alpha = 0.2, linetype = "longdash", size = 0.3, weight = 0.3) +
  stat_cor(method = "pearson", label.y = 7200, label.x = 70, color = "dodgerblue4") +
  ylab("Volume in mm3") + xlab("Age (in years)") +
  theme_minimal() +
  ggtitle("ABSOLUTE VOLUME vermis") +
  theme(plot.title = element_text(hjust = 0.5))
Total.Vermis.Age.plot
ggsave("Total.Vermis.Age.plot.pdf", plot = Total.Vermis.Age.plot, width = 8, height = 6, units = "in", dpi = 600)  
 
 
   
 
 
  
Total.Hemisphere.Age.plot <-  ggplot(Brainstem.Cerebellum.absolute, aes(y=Hemisphere, x = Age))  +
  geom_point(aes(color = Gender), size = 1.5, alpha = 1, shape = 16) +
  scale_color_manual(values = c("chartreuse4", "orangered2")) +
  geom_smooth(method='lm', alpha = 0.2, colour = "dodgerblue4", size = 0.8, weight = 0.3) +
  geom_smooth(aes(color = Gender), method='lm', se = F, alpha = 0.2, linetype = "longdash", size = 0.3, weight = 0.3) +
  stat_cor(method = "pearson", label.y = 123000, label.x = 70, color = "dodgerblue4") +
  ylab("Volume in mm3") + xlab("Age (in years)") +
  theme_minimal() +
  ggtitle("ABSOLUTE VOLUME hemisphere") +
  theme(plot.title = element_text(hjust = 0.5))
Total.Hemisphere.Age.plot
ggsave("Total.Hemisphere.Age.plot.pdf", plot = Total.Hemisphere.Age.plot, width = 8, height = 6, units = "in", dpi = 600)  
 
 
   
 
 
  
Total.Anterior.lobe.Age.plot <-  ggplot(Brainstem.Cerebellum.absolute, aes(y=Anterior.lobe, x = Age))  +
  geom_point(aes(color = Gender), size = 1.5, alpha = 1, shape = 16) +
  scale_color_manual(values = c("chartreuse4", "orangered2")) +
  geom_smooth(method='lm', alpha = 0.2, colour = "dodgerblue4", size = 0.8, weight = 0.3) +
  geom_smooth(aes(color = Gender), method='lm', se = F, alpha = 0.2, linetype = "longdash", size = 0.3, weight = 0.3) +
  stat_cor(method = "pearson", label.y = 36000, label.x = 70, color = "dodgerblue4") +
  ylab("Volume in mm3") + xlab("Age (in years)") +
  theme_minimal() +
  ggtitle("ABSOLUTE VOLUME anterior lobe") +
  theme(plot.title = element_text(hjust = 0.5))
Total.Anterior.lobe.Age.plot
ggsave("Total.Anterior.lobe.Age.plot.pdf", plot = Total.Anterior.lobe.Age.plot, width = 8, height = 6, units = "in", dpi = 600)  
 
 
   
 
 
  
Total.Medial.lobe.Age.plot <-  ggplot(Brainstem.Cerebellum.absolute, aes(y=Medial.lobe, x = Age))  +
  geom_point(aes(color = Gender), size = 1.5, alpha = 1, shape = 16) +
  scale_color_manual(values = c("chartreuse4", "orangered2")) +
  geom_smooth(method='lm', alpha = 0.2, colour = "dodgerblue4", size = 0.8, weight = 0.3) +
  geom_smooth(aes(color = Gender), method='lm', se = F, alpha = 0.2, linetype = "longdash", size = 0.3, weight = 0.3) +
  stat_cor(method = "pearson", label.y = 31000, label.x = 70, color = "dodgerblue4") +
  ylab("Volume in mm3") + xlab("Age (in years)") +
  theme_minimal() +
  ggtitle("ABSOLUTE VOLUME medial lobe") +
  theme(plot.title = element_text(hjust = 0.5))
Total.Medial.lobe.Age.plot
ggsave("Total.Medial.lobe.Age.plot.pdf", plot = Total.Medial.lobe.Age.plot, width = 8, height = 6, units = "in", dpi = 600)  
 
 
   
 
 
  
Total.Posterior.lobe.Age.plot <-  ggplot(Brainstem.Cerebellum.absolute, aes(y=Posterior.lobe, x = Age))  +
  geom_point(aes(color = Gender), size = 1.5, alpha = 1, shape = 16) +
  scale_color_manual(values = c("chartreuse4", "orangered2")) +
  geom_smooth(method='lm', alpha = 0.2, colour = "dodgerblue4", size = 0.8, weight = 0.3) +
  geom_smooth(aes(color = Gender), method='lm', se = F, alpha = 0.2, linetype = "longdash", size = 0.3, weight = 0.3) +
  stat_cor(method = "pearson", label.y = 66000, label.x = 70, color = "dodgerblue4") +
  ylab("Volume in mm3") + xlab("Age (in years)") +
  theme_minimal() +
  ggtitle("ABSOLUTE VOLUME posterior lobe") +
  theme(plot.title = element_text(hjust = 0.5))
Total.Posterior.lobe.Age.plot
ggsave("Total.Posterior.lobe.Age.plot.pdf", plot = Total.Posterior.lobe.Age.plot, width = 8, height = 6, units = "in", dpi = 600)  
 
 
   
 
 
  
Total.Flocculonodular.lobe.Age.plot <-  ggplot(Brainstem.Cerebellum.absolute, aes(y=Flocculonodular.lobe, x = Age))  +
  geom_point(aes(color = Gender), size = 1.5, alpha = 1, shape = 16) +
  scale_color_manual(values = c("chartreuse4", "orangered2")) +
  geom_smooth(method='lm', alpha = 0.2, colour = "dodgerblue4", size = 0.8, weight = 0.3) +
  geom_smooth(aes(color = Gender), method='lm', se = F, alpha = 0.2, linetype = "longdash", size = 0.3, weight = 0.3) +
  stat_cor(method = "pearson", label.y = 1250, label.x = 70, color = "dodgerblue4") +
  ylab("Volume in mm3") + xlab("Age (in years)") +
  theme_minimal() +
  ggtitle("ABSOLUTE VOLUME flocculonodular lobe") +
  theme(plot.title = element_text(hjust = 0.5))
Total.Flocculonodular.lobe.Age.plot
ggsave("Total.Flocculonodular.lobe.Age.plot.pdf", plot = Total.Flocculonodular.lobe.Age.plot, width = 8, height = 6, units = "in", dpi = 600)  
 
 
   
 
 
  
Total.Central.Age.plot <-  ggplot(Brainstem.Cerebellum.absolute, aes(y=Central, x = Age))  +
  geom_point(aes(color = Gender), size = 1.5, alpha = 1, shape = 16) +
  scale_color_manual(values = c("chartreuse4", "orangered2")) +
  geom_smooth(method='lm', alpha = 0.2, colour = "dodgerblue4", size = 0.8, weight = 0.3) +
  geom_smooth(aes(color = Gender), method='lm', se = F, alpha = 0.2, linetype = "longdash", size = 0.3, weight = 0.3) +
  stat_cor(method = "pearson", label.y = 770, label.x = 70, color = "dodgerblue4") +
  ylab("Volume in mm3") + xlab("Age (in years)") +
  theme_minimal() +
  ggtitle("ABSOLUTE VOLUME central") +
  theme(plot.title = element_text(hjust = 0.5))
Total.Central.Age.plot
ggsave("Total.Central.Age.plot.pdf", plot = Total.Central.Age.plot, width = 8, height = 6, units = "in", dpi = 600)  
 
 
   
 
 
  
Total.Culmen.Age.plot <-  ggplot(Brainstem.Cerebellum.absolute, aes(y=Culmen, x = Age))  +
  geom_point(aes(color = Gender), size = 1.5, alpha = 1, shape = 16) +
  scale_color_manual(values = c("chartreuse4", "orangered2")) +
  geom_smooth(method='lm', alpha = 0.2, colour = "dodgerblue4", linetype = "longdash", size = 0.5, weight = 0.3) +
  geom_smooth(aes(color = Gender), method='lm', se = F, alpha = 0.2, linetype = "longdash", size = 0.5, weight = 0.3) +
  stat_cor(method = "pearson", label.y = 2600, label.x = 70, color = "dodgerblue4") +
  ylab("Volume in mm3") + xlab("Age (in years)") +
  theme_minimal() +
  ggtitle("ABSOLUTE VOLUME CULMEN") +
  theme(plot.title = element_text(hjust = 0.5))
Total.Culmen.Age.plot
ggsave("Total.Culmen.Age.plot.pdf", plot = Total.Culmen.Age.plot, width = 8, height = 6, units = "in", dpi = 600)  
 
 
   
 
 
  
Total.Declive.Age.plot <-  ggplot(Brainstem.Cerebellum.absolute, aes(y=Declive, x = Age))  +
  geom_point(aes(color = Gender), size = 1.5, alpha = 1, shape = 16) +
  scale_color_manual(values = c("chartreuse4", "orangered2")) +
  geom_smooth(method='lm', alpha = 0.2, colour = "dodgerblue4", linetype = "longdash", size = 0.5, weight = 0.3) +
  geom_smooth(aes(color = Gender), method='lm', se = F, alpha = 0.2, linetype = "longdash", size = 0.5, weight = 0.3) +
  stat_cor(method = "pearson", label.y = 1250, label.x = 70, color = "dodgerblue4") +
  ylab("Volume in mm3") + xlab("Age (in years)") +
  theme_minimal() +
  ggtitle("ABSOLUTE VOLUME DECLIVE") +
  theme(plot.title = element_text(hjust = 0.5))
Total.Declive.Age.plot
ggsave("Total.Declive.Age.plot.pdf", plot = Total.Declive.Age.plot, width = 8, height = 6, units = "in", dpi = 600)  
 
 
   
 
 
  
Total.Folium.Age.plot <-  ggplot(Brainstem.Cerebellum.absolute, aes(y=Folium, x = Age))  +
  geom_point(aes(color = Gender), size = 1.5, alpha = 1, shape = 16) +
  scale_color_manual(values = c("chartreuse4", "orangered2")) +
  geom_smooth(method='lm', alpha = 0.2, colour = "dodgerblue4", linetype = "longdash", size = 0.5, weight = 0.3) +
  geom_smooth(aes(color = Gender), method='lm', se = F, alpha = 0.2, linetype = "longdash", size = 0.5, weight = 0.3) +
  stat_cor(method = "pearson", label.y = 630, label.x = 70, color = "dodgerblue4") +
  ylab("Volume in mm3") + xlab("Age (in years)") +
  theme_minimal() +
  ggtitle("ABSOLUTE VOLUME FOLIUM") +
  theme(plot.title = element_text(hjust = 0.5))
Total.Folium.Age.plot
ggsave("Total.Folium.Age.plot.pdf", plot = Total.Folium.Age.plot, width = 8, height = 6, units = "in", dpi = 600)  
 
 
   
 
 
  
Total.Tuber.Age.plot <-  ggplot(Brainstem.Cerebellum.absolute, aes(y=Tuber, x = Age))  +
  geom_point(aes(color = Gender), size = 1.5, alpha = 1, shape = 16) +
  scale_color_manual(values = c("chartreuse4", "orangered2")) +
  geom_smooth(method='lm', alpha = 0.2, colour = "dodgerblue4", linetype = "longdash", size = 0.5, weight = 0.3) +
  geom_smooth(aes(color = Gender), method='lm', se = F, alpha = 0.2, linetype = "longdash", size = 0.5, weight = 0.3) +
  stat_cor(method = "pearson", label.y = 570, label.x = 70, color = "dodgerblue4") +
  ylab("Volume in mm3") + xlab("Age (in years)") +
  theme_minimal() +
  ggtitle("ABSOLUTE VOLUME TUBER") +
  theme(plot.title = element_text(hjust = 0.5))
Total.Tuber.Age.plot
ggsave("Total.Tuber.Age.plot.pdf", plot = Total.Tuber.Age.plot, width = 8, height = 6, units = "in", dpi = 600)  
 
 
   
 
 
  
Total.Pyramid.Age.plot <-  ggplot(Brainstem.Cerebellum.absolute, aes(y=Pyramid, x = Age))  +
  geom_point(aes(color = Gender), size = 1.5, alpha = 1, shape = 16) +
  scale_color_manual(values = c("chartreuse4", "orangered2")) +
  geom_smooth(method='lm', alpha = 0.2, colour = "dodgerblue4", linetype = "longdash", size = 0.5, weight = 0.3) +
  geom_smooth(aes(color = Gender), method='lm', se = F, alpha = 0.2, linetype = "longdash", size = 0.5, weight = 0.3) +
  stat_cor(method = "pearson", label.y = 700, label.x = 70, color = "dodgerblue4") +
  ylab("Volume in mm3") + xlab("Age (in years)") +
  theme_minimal() +
  ggtitle("ABSOLUTE VOLUME PYRAMID") +
  theme(plot.title = element_text(hjust = 0.5))
Total.Pyramid.Age.plot
ggsave("Total.Pyramid.Age.plot.pdf", plot = Total.Pyramid.Age.plot, width = 8, height = 6, units = "in", dpi = 600)  
 
 
   
 
 
  
Total.Uvula.Age.plot <-  ggplot(Brainstem.Cerebellum.absolute, aes(y=Uvula, x = Age))  +
  geom_point(aes(color = Gender), size = 1.5, alpha = 1, shape = 16) +
  scale_color_manual(values = c("chartreuse4", "orangered2")) +
  geom_smooth(method='lm', alpha = 0.2, colour = "dodgerblue4", linetype = "longdash", size = 0.5, weight = 0.3) +
  geom_smooth(aes(color = Gender), method='lm', se = F, alpha = 0.2, linetype = "longdash", size = 0.5, weight = 0.3) +
  stat_cor(method = "pearson", label.y = 1030, label.x = 70, color = "dodgerblue4") +
  ylab("Volume in mm3") + xlab("Age (in years)") +
  theme_minimal() +
  ggtitle("ABSOLUTE VOLUME UVULA") +
  theme(plot.title = element_text(hjust = 0.5))
Total.Uvula.Age.plot
ggsave("Total.Uvula.Age.plot.pdf", plot = Total.Uvula.Age.plot, width = 8, height = 6, units = "in", dpi = 600)  
 
 
   
 
 
  
Total.Nodule.Age.plot <-  ggplot(Brainstem.Cerebellum.absolute, aes(y=Nodule, x = Age))  +
  geom_point(aes(color = Gender), size = 1.5, alpha = 1, shape = 16) +
  scale_color_manual(values = c("chartreuse4", "orangered2")) +
  geom_smooth(method='lm', alpha = 0.2, colour = "dodgerblue4", linetype = "longdash", size = 0.5, weight = 0.3) +
  geom_smooth(aes(color = Gender), method='lm', se = F, alpha = 0.2, linetype = "longdash", size = 0.5, weight = 0.3) +
  stat_cor(method = "pearson", label.y = 230, label.x = 70, color = "dodgerblue4") +
  ylab("Volume in mm3") + xlab("Age (in years)") +
  theme_minimal() +
  ggtitle("ABSOLUTE VOLUME NODULE") +
  theme(plot.title = element_text(hjust = 0.5))
Total.Nodule.Age.plot
ggsave("Total.Nodule.Age.plot.pdf", plot = Total.Nodule.Age.plot, width = 8, height = 6, units = "in", dpi = 600)  
 
 
   
 
 
  
Total.Ala.lobuli.centralis.Age.plot <-  ggplot(Brainstem.Cerebellum.absolute, aes(y=Ala.lobuli.centralis, x = Age))  +
  geom_point(aes(color = Gender), size = 1.5, alpha = 1, shape = 16) +
  scale_color_manual(values = c("chartreuse4", "orangered2")) +
  geom_smooth(method='lm', alpha = 0.2, colour = "dodgerblue4", linetype = "longdash", size = 0.5, weight = 0.3) +
  geom_smooth(aes(color = Gender), method='lm', se = F, alpha = 0.2, linetype = "longdash", size = 0.5, weight = 0.3) +
  stat_cor(method = "pearson", label.y = 16000, label.x = 70, color = "dodgerblue4") +
  ylab("Volume in mm3") + xlab("Age (in years)") +
  theme_minimal() +
  ggtitle("ABSOLUTE VOLUME ALA LOBULI CENTRALIS") +
  theme(plot.title = element_text(hjust = 0.5))
Total.Ala.lobuli.centralis.Age.plot
ggsave("Total.Ala.lobuli.centralis.Age.plot.pdf", plot = Total.Ala.lobuli.centralis.Age.plot, width = 8, height = 6, units = "in", dpi = 600)  
 
 
   
 
 
  
Total.AQL.Age.plot <-  ggplot(Brainstem.Cerebellum.absolute, aes(y=AQL, x = Age))  +
  geom_point(aes(color = Gender), size = 1.5, alpha = 1, shape = 16) +
  scale_color_manual(values = c("chartreuse4", "orangered2")) +
  geom_smooth(method='lm', alpha = 0.2, colour = "dodgerblue4", linetype = "longdash", size = 0.5, weight = 0.3) +
  geom_smooth(aes(color = Gender), method='lm', se = F, alpha = 0.2, linetype = "longdash", size = 0.5, weight = 0.3) +
  stat_cor(method = "pearson", label.y = 21000, label.x = 70, color = "dodgerblue4") +
  ylab("Volume in mm3") + xlab("Age (in years)") +
  theme_minimal() +
  ggtitle("ABSOLUTE VOLUME AQL") +
  theme(plot.title = element_text(hjust = 0.5))
Total.AQL.Age.plot
ggsave("Total.AQL.Age.plot.pdf", plot = Total.AQL.Age.plot, width = 8, height = 6, units = "in", dpi = 600)  
 
 
   
 
 
  
Total.PQL.Age.plot <-  ggplot(Brainstem.Cerebellum.absolute, aes(y=PQL, x = Age))  +
  geom_point(aes(color = Gender), size = 1.5, alpha = 1, shape = 16) +
  scale_color_manual(values = c("chartreuse4", "orangered2")) +
  geom_smooth(method='lm', alpha = 0.2, colour = "dodgerblue4", linetype = "longdash", size = 0.5, weight = 0.3) +
  geom_smooth(aes(color = Gender), method='lm', se = F, alpha = 0.2, linetype = "longdash", size = 0.5, weight = 0.3) +
  stat_cor(method = "pearson", label.y = 15500, label.x = 70, color = "dodgerblue4") +
  ylab("Volume in mm3") + xlab("Age (in years)") +
  theme_minimal() +
  ggtitle("ABSOLUTE VOLUME PQL") +
  theme(plot.title = element_text(hjust = 0.5))
Total.PQL.Age.plot
ggsave("Total.PQL.Age.plot.pdf", plot = Total.PQL.Age.plot, width = 8, height = 6, units = "in", dpi = 600)  
 
 
   
 
 
  
Total.SSL.Age.plot <-  ggplot(Brainstem.Cerebellum.absolute, aes(y=SSL, x = Age))  +
  geom_point(aes(color = Gender), size = 1.5, alpha = 1, shape = 16) +
  scale_color_manual(values = c("chartreuse4", "orangered2")) +
  geom_smooth(method='lm', alpha = 0.2, colour = "dodgerblue4", linetype = "longdash", size = 0.5, weight = 0.3) +
  geom_smooth(aes(color = Gender), method='lm', se = F, alpha = 0.2, linetype = "longdash", size = 0.5, weight = 0.3) +
  stat_cor(method = "pearson", label.y = 15500, label.x = 70, color = "dodgerblue4") +
  ylab("Volume in mm3") + xlab("Age (in years)") +
  theme_minimal() +
  ggtitle("ABSOLUTE VOLUME SSL") +
  theme(plot.title = element_text(hjust = 0.5))
Total.SSL.Age.plot
ggsave("Total.SSL.Age.plot.pdf", plot = Total.SSL.Age.plot, width = 8, height = 6, units = "in", dpi = 600)  
 
 
   
 
 
  
Total.ISL.gracile.Age.plot <-  ggplot(Brainstem.Cerebellum.absolute, aes(y=ISL.gracile, x = Age))  +
  geom_point(aes(color = Gender), size = 1.5, alpha = 1, shape = 16) +
  scale_color_manual(values = c("chartreuse4", "orangered2")) +
  geom_smooth(method='lm', alpha = 0.2, colour = "dodgerblue4", linetype = "longdash", size = 0.5, weight = 0.3) +
  geom_smooth(aes(color = Gender), method='lm', se = F, alpha = 0.2, linetype = "longdash", size = 0.5, weight = 0.3) +
  stat_cor(method = "pearson", label.y = 42000, label.x = 70, color = "dodgerblue4") +
  ylab("Volume in mm3") + xlab("Age (in years)") +
  theme_minimal() +
  ggtitle("ABSOLUTE VOLUME ISL/gracile") +
  theme(plot.title = element_text(hjust = 0.5))
Total.ISL.gracile.Age.plot
ggsave("Total.ISL.gracile.Age.plot.pdf", plot = Total.ISL.gracile.Age.plot, width = 8, height = 6, units = "in", dpi = 600)  
 
 
   
 
 
  
Total.Biventer.Age.plot <-  ggplot(Brainstem.Cerebellum.absolute, aes(y=Biventer, x = Age))  +
  geom_point(aes(color = Gender), size = 1.5, alpha = 1, shape = 16) +
  scale_color_manual(values = c("chartreuse4", "orangered2")) +
  geom_smooth(method='lm', alpha = 0.2, colour = "dodgerblue4", linetype = "longdash", size = 0.5, weight = 0.3) +
  geom_smooth(aes(color = Gender), method='lm', se = F, alpha = 0.2, linetype = "longdash", size = 0.5, weight = 0.3) +
  stat_cor(method = "pearson", label.y = 21000, label.x = 70, color = "dodgerblue4") +
  ylab("Volume in mm3") + xlab("Age (in years)") +
  theme_minimal() +
  ggtitle("ABSOLUTE VOLUME biventer") +
  theme(plot.title = element_text(hjust = 0.5))
Total.Biventer.Age.plot
ggsave("Total.Biventer.Age.plot.pdf", plot = Total.Biventer.Age.plot, width = 8, height = 6, units = "in", dpi = 600)  
 
 
   
 
 
  
Total.Tonsilla.Age.plot <-  ggplot(Brainstem.Cerebellum.absolute, aes(y=Tonsilla, x = Age))  +
  geom_point(aes(color = Gender), size = 1.5, alpha = 1, shape = 16) +
  scale_color_manual(values = c("chartreuse4", "orangered2")) +
  geom_smooth(method='lm', alpha = 0.2, colour = "dodgerblue4", linetype = "longdash", size = 0.5, weight = 0.3) +
  geom_smooth(aes(color = Gender), method='lm', se = F, alpha = 0.2, linetype = "longdash", size = 0.5, weight = 0.3) +
  stat_cor(method = "pearson", label.y = 7700, label.x = 70, color = "dodgerblue4") +
  ylab("Volume in mm3") + xlab("Age (in years)") +
  theme_minimal() +
  ggtitle("ABSOLUTE VOLUME tonsilla") +
  theme(plot.title = element_text(hjust = 0.5))
Total.Tonsilla.Age.plot
ggsave("Total.Tonsilla.Age.plot.pdf", plot = Total.Tonsilla.Age.plot, width = 8, height = 6, units = "in", dpi = 600)  
 
 
   
 
 
  
Total.Flocculus.Age.plot <-  ggplot(Brainstem.Cerebellum.absolute, aes(y=Flocculus, x = Age))  +
  geom_point(aes(color = Gender), size = 1.5, alpha = 1, shape = 16) +
  scale_color_manual(values = c("chartreuse4", "orangered2")) +
  geom_smooth(method='lm', alpha = 0.2, colour = "dodgerblue4", linetype = "longdash", size = 0.5, weight = 0.3) +
  geom_smooth(aes(color = Gender), method='lm', se = F, alpha = 0.2, linetype = "longdash", size = 0.5, weight = 0.3) +
  stat_cor(method = "pearson", label.y = 1050, label.x = 70, color = "dodgerblue4") +
  ylab("Volume in mm3") + xlab("Age (in years)") +
  theme_minimal() +
  ggtitle("ABSOLUTE VOLUME flocculus") +
  theme(plot.title = element_text(hjust = 0.5))
Total.Flocculus.Age.plot
ggsave("Total.Flocculus.Age.plot.pdf", plot = Total.Flocculus.Age.plot, width = 8, height = 6, units = "in", dpi = 600)
  
 
 
   
 
 
 
 
 
 Relative Volumes 
 
 
 
  
#Brainstem.Cerebellum.absolute <- select(Brainstem.Cerebellum.absolute, - c(Age, Gender))
Brainstem.Cerebellum.relative <- (100 * (Brainstem.Cerebellum.absolute/All.Volumes$`Total encephalic volume (without ventricles)`))  
 
 
  ‘/’ not meaningful for factors  
 
 
  Brainstem.Cerebellum.relative1 <- Brainstem.Cerebellum.relative[,c(1:10)]
Brainstem.Cerebellum.relative2 <- Brainstem.Cerebellum.relative[,-c(1:10)]

Table.Brainstem.Cerebellum.relative <- CreateTableOne(
  vars = c("Mesencephalon",
"Pons",
"Medulla.oblongata", 
"Cerebellar.peduncles", 
"Vermis", 
"Hemisphere", 
"Anterior.lobe", 
"Medial.lobe", 
"Posterior.lobe", 
"Flocculonodular.lobe", 
"Central", 
"Culmen", 
"Declive", 
"Folium", 
"Tuber",
"Pyramid", 
"Uvula", 
"Nodule",
"Ala.lobuli.centralis",
"AQL",
"PQL",
"SSL",
"ISL.gracile", 
"Biventer",
"Tonsilla", 
"Flocculus"),
  data = Brainstem.Cerebellum.relative)

Brainstem.Cerebellum.relative$Gender <- All.Volumes$Gender

Table.Brainstem.Cerebellum.relative.stratified.gender <- CreateTableOne(
  vars = c("Mesencephalon",
"Pons",
"Medulla.oblongata", 
"Cerebellar.peduncles", 
"Vermis", 
"Hemisphere", 
"Anterior.lobe", 
"Medial.lobe", 
"Posterior.lobe", 
"Flocculonodular.lobe", 
"Central", 
"Culmen", 
"Declive", 
"Folium", 
"Tuber",
"Pyramid", 
"Uvula", 
"Nodule",
"Ala.lobuli.centralis",
"AQL",
"PQL",
"SSL",
"ISL.gracile", 
"Biventer",
"Tonsilla", 
"Flocculus"),
strata = c("Gender"),
  data = Brainstem.Cerebellum.relative)

Table.Brainstem.Cerebellum.relative <- print(Table.Brainstem.Cerebellum.relative, contDigits = 10)  
 
 
                                    
                                   Overall                     
  n                                           30               
  Mesencephalon (mean (SD))         0.9207623291 (0.1105191701)
  Pons (mean (SD))                  1.4005625067 (0.1727801503)
  Medulla.oblongata (mean (SD))     0.2524061308 (0.0357809075)
  Cerebellar.peduncles (mean (SD))  0.6213805087 (0.0717960379)
  Vermis (mean (SD))                0.5465728045 (0.0706767901)
  Hemisphere (mean (SD))           10.1852538324 (1.1214009177)
  Anterior.lobe (mean (SD))         2.9710945895 (0.4958752121)
  Medial.lobe (mean (SD))           2.6417850797 (0.3687226983)
  Posterior.lobe (mean (SD))        5.0206290086 (0.6951742366)
  Flocculonodular.lobe (mean (SD))  0.0983188189 (0.0174019012)
  Central (mean (SD))               0.0444979014 (0.0120965572)
  Culmen (mean (SD))                0.2006556434 (0.0318361517)
  Declive (mean (SD))               0.0945552953 (0.0166943131)
  Folium (mean (SD))                0.0405176202 (0.0111004272)
  Tuber (mean (SD))                 0.0355347542 (0.0084832011)
  Pyramid (mean (SD))               0.0389858949 (0.0169198532)
  Uvula (mean (SD))                 0.0722229678 (0.0122092488)
  Nodule (mean (SD))                0.0196238560 (0.0037047686)
  Ala.lobuli.centralis (mean (SD))  1.1040297116 (0.2737967502)
  AQL (mean (SD))                   1.6219113332 (0.3797422298)
  PQL (mean (SD))                   1.2023063114 (0.2187152577)
  SSL (mean (SD))                   1.3044058528 (0.2289872084)
  ISL.gracile (mean (SD))           3.2205673072 (0.6269680120)
  Biventer (mean (SD))              1.1070477516 (0.3117981715)
  Tonsilla (mean (SD))              0.5462703329 (0.1165790011)
  Flocculus (mean (SD))             0.0786949629 (0.0155073243)  
 
 
  Table.Brainstem.Cerebellum.relative.stratified.gender <- print(Table.Brainstem.Cerebellum.relative.stratified.gender, contDigits = 10)  
 
 
                                    Stratified by Gender
                                   f                            m                            p      test
  n                                           14                           16                           
  Mesencephalon (mean (SD))         0.9547649875 (0.1034767729)  0.8910100029 (0.1109575365)  0.116     
  Pons (mean (SD))                  1.4035711394 (0.1967402245)  1.3979299532 (0.1554158961)  0.931     
  Medulla.oblongata (mean (SD))     0.2607569676 (0.0330361387)  0.2450991485 (0.0375138378)  0.238     
  Cerebellar.peduncles (mean (SD))  0.6310216888 (0.0701223453)  0.6129444762 (0.0744412960)  0.501     
  Vermis (mean (SD))                0.5614348351 (0.0700918934)  0.5335685277 (0.0708028232)  0.289     
  Hemisphere (mean (SD))           10.3668103244 (1.1765578929) 10.0263919019 (1.0834401354)  0.416     
  Anterior.lobe (mean (SD))         3.0346127423 (0.5156137908)  2.9155162059 (0.4877717301)  0.521     
  Medial.lobe (mean (SD))           2.7827036278 (0.3948805255)  2.5184813501 (0.3048895712)  0.048     
  Posterior.lobe (mean (SD))        5.0087783797 (0.7749481080)  5.0309983090 (0.6431165505)  0.932     
  Flocculonodular.lobe (mean (SD))  0.1022078567 (0.0181582471)  0.0949159108 (0.0165298769)  0.259     
  Central (mean (SD))               0.0484920828 (0.0133583251)  0.0410029928 (0.0100163605)  0.091     
  Culmen (mean (SD))                0.2045461757 (0.0285689132)  0.1972514276 (0.0350094972)  0.541     
  Declive (mean (SD))               0.0983933628 (0.0195622332)  0.0911969862 (0.0134679064)  0.246     
  Folium (mean (SD))                0.0418471653 (0.0093337522)  0.0393542682 (0.0126343898)  0.549     
  Tuber (mean (SD))                 0.0353483403 (0.0083807937)  0.0356978664 (0.0088429783)  0.913     
  Pyramid (mean (SD))               0.0367053806 (0.0154573992)  0.0409813449 (0.0183657952)  0.499     
  Uvula (mean (SD))                 0.0754553918 (0.0127880168)  0.0693945969 (0.0113216555)  0.179     
  Nodule (mean (SD))                0.0207119244 (0.0037738611)  0.0186717962 (0.0034814791)  0.135     
  Ala.lobuli.centralis (mean (SD))  1.1123470528 (0.2881107524)  1.0967520380 (0.2699453959)  0.879     
  AQL (mean (SD))                   1.6692274310 (0.4046689735)  1.5805097476 (0.3646286798)  0.533     
  PQL (mean (SD))                   1.2462629933 (0.2677358247)  1.1638442147 (0.1642482748)  0.311     
  SSL (mean (SD))                   1.3962001064 (0.2267285630)  1.2240858809 (0.2051269225)  0.038     
  ISL.gracile (mean (SD))           3.2680364313 (0.5872772943)  3.1790318236 (0.6761064068)  0.705     
  Biventer (mean (SD))              1.0339684324 (0.3635994447)  1.1709921559 (0.2530448256)  0.236     
  Tonsilla (mean (SD))              0.5592644032 (0.1111017647)  0.5349005213 (0.1236204433)  0.577     
  Flocculus (mean (SD))             0.0814959323 (0.0161449011)  0.0762441146 (0.0150096453)  0.364       
 
 
 
 
 
 
  
Table.Brainstem.Cerebellum.relative.RSD <- as.data.frame(Table.Brainstem.Cerebellum.relative)
Table.Brainstem.Cerebellum.relative.RSD <- data.frame(do.call('rbind', 
                                                              strsplit(as.character(Table.Brainstem.Cerebellum.relative.RSD[-1,]),
                                                                       ' (',fixed=TRUE)))
Table.Brainstem.Cerebellum.relative.RSD <- data.frame(cbind(str_replace_all(Table.Brainstem.Cerebellum.relative.RSD$X1, "[ ]", ""),
                                                            str_replace_all(Table.Brainstem.Cerebellum.relative.RSD$X2, "[)]", "")))
Table.Brainstem.Cerebellum.relative.RSD$X1 <- as.character(Table.Brainstem.Cerebellum.relative.RSD$X1)
Table.Brainstem.Cerebellum.relative.RSD$X2 <- as.character(Table.Brainstem.Cerebellum.relative.RSD$X2)
Table.Brainstem.Cerebellum.relative.RSD <- as.data.frame(sapply(Table.Brainstem.Cerebellum.relative.RSD, as.numeric))
Table.Brainstem.Cerebellum.relative.RSD <- as.data.frame(Table.Brainstem.Cerebellum.relative.RSD$X2/Table.Brainstem.Cerebellum.relative.RSD$X1)
Table.Brainstem.Cerebellum.relative.RSD <- round(Table.Brainstem.Cerebellum.relative.RSD * 100, 1)


Table.Brainstem.Cerebellum.relative.stratified.gender.RSD <- as.data.frame(Table.Brainstem.Cerebellum.relative.stratified.gender)
Table.Brainstem.Cerebellum.relative.stratified.gender.RSD <- select(Table.Brainstem.Cerebellum.relative.stratified.gender.RSD, - c(p, test))

Table.Brainstem.Cerebellum.relative.stratified.gender.RSD.female <- data.frame(do.call('rbind', 
                                                              strsplit(as.character(Table.Brainstem.Cerebellum.relative.stratified.gender.RSD[-1, "f"]),
                                                                       ' (',fixed=TRUE)))
Table.Brainstem.Cerebellum.relative.stratified.gender.RSD.female <- data.frame(cbind(str_replace_all(Table.Brainstem.Cerebellum.relative.stratified.gender.RSD.female$X1, "[ ]", ""),
                                                            str_replace_all(Table.Brainstem.Cerebellum.relative.stratified.gender.RSD.female$X2, "[)]", "")))
Table.Brainstem.Cerebellum.relative.stratified.gender.RSD.female$X1 <- as.character(Table.Brainstem.Cerebellum.relative.stratified.gender.RSD.female$X1)
Table.Brainstem.Cerebellum.relative.stratified.gender.RSD.female$X2 <- as.character(Table.Brainstem.Cerebellum.relative.stratified.gender.RSD.female$X2)
Table.Brainstem.Cerebellum.relative.stratified.gender.RSD.female <- as.data.frame(sapply(Table.Brainstem.Cerebellum.relative.stratified.gender.RSD.female, as.numeric))
Table.Brainstem.Cerebellum.relative.stratified.gender.RSD.female <- as.data.frame(Table.Brainstem.Cerebellum.relative.stratified.gender.RSD.female$X2/Table.Brainstem.Cerebellum.relative.stratified.gender.RSD.female$X1)
Table.Brainstem.Cerebellum.relative.stratified.gender.RSD.female <- round(Table.Brainstem.Cerebellum.relative.stratified.gender.RSD.female * 100, 1)

Table.Brainstem.Cerebellum.relative.stratified.gender.RSD.male <- data.frame(do.call('rbind', 
                                                              strsplit(as.character(Table.Brainstem.Cerebellum.relative.stratified.gender.RSD[-1, "m"]),
                                                                       ' (',fixed=TRUE)))
Table.Brainstem.Cerebellum.relative.stratified.gender.RSD.male <- data.frame(cbind(str_replace_all(Table.Brainstem.Cerebellum.relative.stratified.gender.RSD.male$X1, "[ ]", ""),
                                                            str_replace_all(Table.Brainstem.Cerebellum.relative.stratified.gender.RSD.male$X2, "[)]", "")))
Table.Brainstem.Cerebellum.relative.stratified.gender.RSD.male$X1 <- as.character(Table.Brainstem.Cerebellum.relative.stratified.gender.RSD.male$X1)
Table.Brainstem.Cerebellum.relative.stratified.gender.RSD.male$X2 <- as.character(Table.Brainstem.Cerebellum.relative.stratified.gender.RSD.male$X2)
Table.Brainstem.Cerebellum.relative.stratified.gender.RSD.male <- as.data.frame(sapply(Table.Brainstem.Cerebellum.relative.stratified.gender.RSD.male, as.numeric))
Table.Brainstem.Cerebellum.relative.stratified.gender.RSD.male <- as.data.frame(Table.Brainstem.Cerebellum.relative.stratified.gender.RSD.male$X2/Table.Brainstem.Cerebellum.relative.stratified.gender.RSD.male$X1)
Table.Brainstem.Cerebellum.relative.stratified.gender.RSD.male <- round(Table.Brainstem.Cerebellum.relative.stratified.gender.RSD.male * 100, 1)
  
 
 
 
 
 
 
  
kable(Table.Brainstem.Cerebellum.relative)  
 
 
 

 
 
 
  
 Overall 
 
 
 
 
 n 
 30 
 
 
 Mesencephalon (mean (SD)) 
 0.9207623291 (0.1105191701) 
 
 
 Pons (mean (SD)) 
 1.4005625067 (0.1727801503) 
 
 
 Medulla.oblongata (mean (SD)) 
 0.2524061308 (0.0357809075) 
 
 
 Cerebellar.peduncles (mean (SD)) 
 0.6213805087 (0.0717960379) 
 
 
 Vermis (mean (SD)) 
 0.5465728045 (0.0706767901) 
 
 
 Hemisphere (mean (SD)) 
 10.1852538324 (1.1214009177) 
 
 
 Anterior.lobe (mean (SD)) 
 2.9710945895 (0.4958752121) 
 
 
 Medial.lobe (mean (SD)) 
 2.6417850797 (0.3687226983) 
 
 
 Posterior.lobe (mean (SD)) 
 5.0206290086 (0.6951742366) 
 
 
 Flocculonodular.lobe (mean (SD)) 
 0.0983188189 (0.0174019012) 
 
 
 Central (mean (SD)) 
 0.0444979014 (0.0120965572) 
 
 
 Culmen (mean (SD)) 
 0.2006556434 (0.0318361517) 
 
 
 Declive (mean (SD)) 
 0.0945552953 (0.0166943131) 
 
 
 Folium (mean (SD)) 
 0.0405176202 (0.0111004272) 
 
 
 Tuber (mean (SD)) 
 0.0355347542 (0.0084832011) 
 
 
 Pyramid (mean (SD)) 
 0.0389858949 (0.0169198532) 
 
 
 Uvula (mean (SD)) 
 0.0722229678 (0.0122092488) 
 
 
 Nodule (mean (SD)) 
 0.0196238560 (0.0037047686) 
 
 
 Ala.lobuli.centralis (mean (SD)) 
 1.1040297116 (0.2737967502) 
 
 
 AQL (mean (SD)) 
 1.6219113332 (0.3797422298) 
 
 
 PQL (mean (SD)) 
 1.2023063114 (0.2187152577) 
 
 
 SSL (mean (SD)) 
 1.3044058528 (0.2289872084) 
 
 
 ISL.gracile (mean (SD)) 
 3.2205673072 (0.6269680120) 
 
 
 Biventer (mean (SD)) 
 1.1070477516 (0.3117981715) 
 
 
 Tonsilla (mean (SD)) 
 0.5462703329 (0.1165790011) 
 
 
 Flocculus (mean (SD)) 
 0.0786949629 (0.0155073243) 
 
 
 


 
   
  kable(Table.Brainstem.Cerebellum.relative.RSD)  
 
 
 

 
 
 
 
 
 
 Table.Brainstem.Cerebellum.relative.RSD  X 2/ T  a  b  l  e . B  r  a  i  n  s  t  e  m . C  e  r  e  b  e  l  l  u  m . r  e  l  a  t  i  v  e . R  S  D  X1 
 
 
 
 
 12.0 
 
 
 12.3 
 
 
 14.2 
 
 
 11.6 
 
 
 12.9 
 
 
 11.0 
 
 
 16.7 
 
 
 14.0 
 
 
 13.8 
 
 
 17.7 
 
 
 27.2 
 
 
 15.9 
 
 
 17.7 
 
 
 27.4 
 
 
 23.9 
 
 
 43.4 
 
 
 16.9 
 
 
 18.9 
 
 
 24.8 
 
 
 23.4 
 
 
 18.2 
 
 
 17.6 
 
 
 19.5 
 
 
 28.2 
 
 
 21.3 
 
 
 19.7 
 
 
 


 
   
  kable(Table.Brainstem.Cerebellum.relative.stratified.gender)  
 
 
 

 
 
 
 
 
 
 
 
 
 
  
 f 
 m 
 p 
 test 
 
 
 
 
 n 
 14 
 16 
  
  
 
 
 Mesencephalon (mean (SD)) 
 0.9547649875 (0.1034767729) 
 0.8910100029 (0.1109575365) 
 0.116 
  
 
 
 Pons (mean (SD)) 
 1.4035711394 (0.1967402245) 
 1.3979299532 (0.1554158961) 
 0.931 
  
 
 
 Medulla.oblongata (mean (SD)) 
 0.2607569676 (0.0330361387) 
 0.2450991485 (0.0375138378) 
 0.238 
  
 
 
 Cerebellar.peduncles (mean (SD)) 
 0.6310216888 (0.0701223453) 
 0.6129444762 (0.0744412960) 
 0.501 
  
 
 
 Vermis (mean (SD)) 
 0.5614348351 (0.0700918934) 
 0.5335685277 (0.0708028232) 
 0.289 
  
 
 
 Hemisphere (mean (SD)) 
 10.3668103244 (1.1765578929) 
 10.0263919019 (1.0834401354) 
 0.416 
  
 
 
 Anterior.lobe (mean (SD)) 
 3.0346127423 (0.5156137908) 
 2.9155162059 (0.4877717301) 
 0.521 
  
 
 
 Medial.lobe (mean (SD)) 
 2.7827036278 (0.3948805255) 
 2.5184813501 (0.3048895712) 
 0.048 
  
 
 
 Posterior.lobe (mean (SD)) 
 5.0087783797 (0.7749481080) 
 5.0309983090 (0.6431165505) 
 0.932 
  
 
 
 Flocculonodular.lobe (mean (SD)) 
 0.1022078567 (0.0181582471) 
 0.0949159108 (0.0165298769) 
 0.259 
  
 
 
 Central (mean (SD)) 
 0.0484920828 (0.0133583251) 
 0.0410029928 (0.0100163605) 
 0.091 
  
 
 
 Culmen (mean (SD)) 
 0.2045461757 (0.0285689132) 
 0.1972514276 (0.0350094972) 
 0.541 
  
 
 
 Declive (mean (SD)) 
 0.0983933628 (0.0195622332) 
 0.0911969862 (0.0134679064) 
 0.246 
  
 
 
 Folium (mean (SD)) 
 0.0418471653 (0.0093337522) 
 0.0393542682 (0.0126343898) 
 0.549 
  
 
 
 Tuber (mean (SD)) 
 0.0353483403 (0.0083807937) 
 0.0356978664 (0.0088429783) 
 0.913 
  
 
 
 Pyramid (mean (SD)) 
 0.0367053806 (0.0154573992) 
 0.0409813449 (0.0183657952) 
 0.499 
  
 
 
 Uvula (mean (SD)) 
 0.0754553918 (0.0127880168) 
 0.0693945969 (0.0113216555) 
 0.179 
  
 
 
 Nodule (mean (SD)) 
 0.0207119244 (0.0037738611) 
 0.0186717962 (0.0034814791) 
 0.135 
  
 
 
 Ala.lobuli.centralis (mean (SD)) 
 1.1123470528 (0.2881107524) 
 1.0967520380 (0.2699453959) 
 0.879 
  
 
 
 AQL (mean (SD)) 
 1.6692274310 (0.4046689735) 
 1.5805097476 (0.3646286798) 
 0.533 
  
 
 
 PQL (mean (SD)) 
 1.2462629933 (0.2677358247) 
 1.1638442147 (0.1642482748) 
 0.311 
  
 
 
 SSL (mean (SD)) 
 1.3962001064 (0.2267285630) 
 1.2240858809 (0.2051269225) 
 0.038 
  
 
 
 ISL.gracile (mean (SD)) 
 3.2680364313 (0.5872772943) 
 3.1790318236 (0.6761064068) 
 0.705 
  
 
 
 Biventer (mean (SD)) 
 1.0339684324 (0.3635994447) 
 1.1709921559 (0.2530448256) 
 0.236 
  
 
 
 Tonsilla (mean (SD)) 
 0.5592644032 (0.1111017647) 
 0.5349005213 (0.1236204433) 
 0.577 
  
 
 
 Flocculus (mean (SD)) 
 0.0814959323 (0.0161449011) 
 0.0762441146 (0.0150096453) 
 0.364 
  
 
 
 


 
   
  kable(Table.Brainstem.Cerebellum.relative.stratified.gender.RSD.female)  
 
 
 

 
 
 
 
 
 
 Table.Brainstem.Cerebellum.relative.stratified.gender.RSD.female  X 2/ T  a  b  l  e . B  r  a  i  n  s  t  e  m . C  e  r  e  b  e  l  l  u  m . r  e  l  a  t  i  v  e . s  t  r  a  t  i  f  i  e  d . g  e  n  d  e  r . R  S  D . f  e  m  a  l  e  X1 
 
 
 
 
 10.8 
 
 
 14.0 
 
 
 12.7 
 
 
 11.1 
 
 
 12.5 
 
 
 11.3 
 
 
 17.0 
 
 
 14.2 
 
 
 15.5 
 
 
 17.8 
 
 
 27.5 
 
 
 14.0 
 
 
 19.9 
 
 
 22.3 
 
 
 23.7 
 
 
 42.1 
 
 
 16.9 
 
 
 18.2 
 
 
 25.9 
 
 
 24.2 
 
 
 21.5 
 
 
 16.2 
 
 
 18.0 
 
 
 35.2 
 
 
 19.9 
 
 
 19.8 
 
 
 


 
   
  kable(Table.Brainstem.Cerebellum.relative.stratified.gender.RSD.male)  
 
 
 

 
 
 
 
 
 
 Table.Brainstem.Cerebellum.relative.stratified.gender.RSD.male  X 2/ T  a  b  l  e . B  r  a  i  n  s  t  e  m . C  e  r  e  b  e  l  l  u  m . r  e  l  a  t  i  v  e . s  t  r  a  t  i  f  i  e  d . g  e  n  d  e  r . R  S  D . m  a  l  e  X1 
 
 
 
 
 12.5 
 
 
 11.1 
 
 
 15.3 
 
 
 12.1 
 
 
 13.3 
 
 
 10.8 
 
 
 16.7 
 
 
 12.1 
 
 
 12.8 
 
 
 17.4 
 
 
 24.4 
 
 
 17.7 
 
 
 14.8 
 
 
 32.1 
 
 
 24.8 
 
 
 44.8 
 
 
 16.3 
 
 
 18.6 
 
 
 24.6 
 
 
 23.1 
 
 
 14.1 
 
 
 16.8 
 
 
 21.3 
 
 
 21.6 
 
 
 23.1 
 
 
 19.7 
 
 
 


 
   
  NA  
 
 
 
 
 
 
  
names.anatomical.structures.temporary <- c("Mesencephalon",
"Pons",
"Medulla.oblongata", 
"Cerebellar.peduncles", 
"Vermis", 
"Hemisphere", 
"Anterior.lobe", 
"Medial.lobe", 
"Posterior.lobe", 
"Flocculonodular.lobe")

names.anatomical.structures.definitive <- c("Mesencephalon",
"Pons",
"Medulla oblongata", 
"Cerebellar peduncles", 
"Vermis", 
"Hemisphere", 
"Anterior lobe", 
"Medial lobe", 
"Posterior lobe", 
"Flocculonodular lobe")

Brainstem.Cerebellum.relative.plotdata1 <- gather(Brainstem.Cerebellum.relative1, "anatomical.structure", "relative.volume")
Brainstem.Cerebellum.relative.plotdata1$Gender <- All.Volumes$Gender
Brainstem.Cerebellum.relative.plotdata1$Age <- All.Volumes$`Age (years)`

Brainstem.Cerebellum.relative.plotdata1$Gender <- factor(Brainstem.Cerebellum.relative.plotdata1$Gender, levels = c("f", "m"), c("f", "m"))
Brainstem.Cerebellum.relative.plotdata1$anatomical.structure <- factor(Brainstem.Cerebellum.relative.plotdata1$anatomical.structure, 
                                                                levels = rev(c(names.anatomical.structures.temporary)), rev(c(names.anatomical.structures.definitive)))

Brainstem.Cerebellum.relative.plot1 <-  ggplot(Brainstem.Cerebellum.relative.plotdata1, aes(x=anatomical.structure, y = relative.volume))  +
  stat_summary(alpha = 0.3, fun = mean, geom = "bar", width = 0.3, fill = "gray50") + 
  geom_boxplot(aes(fill = Gender), alpha = 0.5, width = 0.4, size = 0.2, position = position_dodge(width = 0.6), 
               outlier.shape = NA, color = "gray30") +
  scale_fill_manual(values = c("chartreuse4", "orangered2")) +
  geom_quasirandom(aes(color = Age), size = 0.7, alpha = 0.8, shape = 16, position = "dodge") +
  scale_color_continuous(low = "steelblue1", high = "red4") +
  xlab("") + ylab("Relative volume (in %)") +
  theme_minimal() +
  coord_flip() +
  ggtitle("BRAINSTEM & CEREBELLUM") +
  theme(plot.title = element_text(hjust = 0.5))

Brainstem.Cerebellum.relative.plot1
ggsave("Brainstem.Cerebellum.relative.plot1.pdf", plot = Brainstem.Cerebellum.relative.plot1, width = 12, height = 6, units = "in", dpi = 600)
  
 
 
   
 
 
 
 
 
 
  
names.anatomical.structures.temporary <- c(
"Central", 
"Culmen", 
"Declive", 
"Folium", 
"Tuber",
"Pyramid", 
"Uvula", 
"Nodule",
"Ala.lobuli.centralis",
"AQL",
"PQL",
"SSL",
"ISL.gracile", 
"Biventer",
"Tonsilla", 
"Flocculus")

names.anatomical.structures.definitive <- c(
"Central", 
"Culmen", 
"Declive", 
"Folium", 
"Tuber",
"Pyramid", 
"Uvula", 
"Nodule",
"Ala lobuli centralis",
"AQL",
"PQL",
"SSL",
"ISL/gracile", 
"Biventer",
"Tonsilla", 
"Flocculus")

Brainstem.Cerebellum.relative.plotdata2 <- gather(Brainstem.Cerebellum.relative2, "anatomical.structure", "relative.volume")
Brainstem.Cerebellum.relative.plotdata2$Gender <- All.Volumes$Gender
Brainstem.Cerebellum.relative.plotdata2$Age <- All.Volumes$`Age (years)`

Brainstem.Cerebellum.relative.plotdata2$Gender <- factor(Brainstem.Cerebellum.relative.plotdata2$Gender, levels = c("f", "m"), c("f", "m"))
Brainstem.Cerebellum.relative.plotdata2$anatomical.structure <- factor(Brainstem.Cerebellum.relative.plotdata2$anatomical.structure, 
                                                                levels = rev(c(names.anatomical.structures.temporary)), rev(c(names.anatomical.structures.definitive)))

Brainstem.Cerebellum.relative.plot2 <-  ggplot(Brainstem.Cerebellum.relative.plotdata2, aes(x=anatomical.structure, y = relative.volume))  +
  stat_summary(alpha = 0.3, fun = mean, geom = "bar", width = 0.3, fill = "gray50") + 
  geom_boxplot(aes(fill = Gender), alpha = 0.5, width = 0.4, size = 0.2, position = position_dodge(width = 0.6), 
               outlier.shape = NA, color = "gray30") +
  scale_fill_manual(values = c("chartreuse4", "orangered2")) +
  geom_quasirandom(aes(color = Age), size = 0.7, alpha = 0.8, shape = 16, position = "dodge") +
  scale_color_continuous(low = "steelblue1", high = "red4") +
  xlab("") + ylab("Relative volume (in %)") +
  theme_minimal() +
  coord_flip() +
  ggtitle("CEREBELLAR LOBES & LOBULES") +
  theme(plot.title = element_text(hjust = 0.5))

Brainstem.Cerebellum.relative.plot2
ggsave("Brainstem.Cerebellum.relative.plot2.pdf", plot = Brainstem.Cerebellum.relative.plot2, width = 12, height = 6, units = "in", dpi = 600)
  
 
 
   
 
 
 
 
 
 
  
#Brainstem.Cerebellum.relative$Gender <- All.Volumes$Gender
Brainstem.Cerebellum.relative$Age <- All.Volumes$`Age (years)`

Relative.Mesencephalon.Age.plot <-  ggplot(Brainstem.Cerebellum.relative, aes(y=Mesencephalon, x = Age))  +
  geom_point(aes(color = Gender), size = 1.5, alpha = 1, shape = 16) +
  scale_color_manual(values = c("chartreuse4", "orangered2")) +
  geom_smooth(method='lm', alpha = 0.2, colour = "dodgerblue4", size = 0.8, weight = 0.3) +
  geom_smooth(aes(color = Gender), method='lm', se = F, alpha = 0.2, linetype = "longdash", size = 0.3, weight = 0.3) +
  stat_cor(method = "pearson", label.y = 1.02, label.x = 70, color = "dodgerblue4") +
  ylab("Relative Volume (in %)") + xlab("Age (in years)") +
  theme_minimal() +
  ggtitle("RELATIVE VOLUME mesencephalon") +
  theme(plot.title = element_text(hjust = 0.5))
Relative.Mesencephalon.Age.plot
ggsave("Relative.Mesencephalon.Age.plot.pdf", plot = Relative.Mesencephalon.Age.plot, width = 8, height = 6, units = "in", dpi = 600)  
 
 
   
 
 
  
Relative.Pons.Age.plot <-  ggplot(Brainstem.Cerebellum.relative, aes(y=Pons, x = Age))  +
  geom_point(aes(color = Gender), size = 1.5, alpha = 1, shape = 16) +
  scale_color_manual(values = c("chartreuse4", "orangered2")) +
  geom_smooth(method='lm', alpha = 0.2, colour = "dodgerblue4", size = 0.8, weight = 0.3) +
  geom_smooth(aes(color = Gender), method='lm', se = F, alpha = 0.2, linetype = "longdash", size = 0.3, weight = 0.3) +
  stat_cor(method = "pearson", label.y = 1.93, label.x = 70, color = "dodgerblue4") +
  ylab("Relative Volume (in %)") + xlab("Age (in years)") +
  theme_minimal() +
  ggtitle("RELATIVE VOLUME pons") +
  theme(plot.title = element_text(hjust = 0.5))
Relative.Pons.Age.plot
ggsave("Relative.Pons.Age.plot.pdf", plot = Relative.Pons.Age.plot, width = 8, height = 6, units = "in", dpi = 600)  
 
 
   
 
 
  
Relative.Medulla.oblongata.Age.plot <-  ggplot(Brainstem.Cerebellum.relative, aes(y=Medulla.oblongata, x = Age))  +
  geom_point(aes(color = Gender), size = 1.5, alpha = 1, shape = 16) +
  scale_color_manual(values = c("chartreuse4", "orangered2")) +
  geom_smooth(method='lm', alpha = 0.2, colour = "dodgerblue4", size = 0.8, weight = 0.3) +
  geom_smooth(aes(color = Gender), method='lm', se = F, alpha = 0.2, linetype = "longdash", size = 0.3, weight = 0.3) +
  stat_cor(method = "pearson", label.y = 0.33, label.x = 70, color = "dodgerblue4") +
  ylab("Relative Volume (in %)") + xlab("Age (in years)") +
  theme_minimal() +
  ggtitle("RELATIVE VOLUME medulla oblongata") +
  theme(plot.title = element_text(hjust = 0.5))
Relative.Medulla.oblongata.Age.plot
ggsave("Relative.Medulla.oblongata.Age.plot.pdf", plot = Relative.Medulla.oblongata.Age.plot, width = 8, height = 6, units = "in", dpi = 600)  
 
 
   
 
 
  
Relative.Cerebellar.peduncles.Age.plot <-  ggplot(Brainstem.Cerebellum.relative, aes(y=Cerebellar.peduncles, x = Age))  +
  geom_point(aes(color = Gender), size = 1.5, alpha = 1, shape = 16) +
  scale_color_manual(values = c("chartreuse4", "orangered2")) +
  geom_smooth(method='lm', alpha = 0.2, colour = "dodgerblue4", size = 0.8, weight = 0.3) +
  geom_smooth(aes(color = Gender), method='lm', se = F, alpha = 0.2, linetype = "longdash", size = 0.3, weight = 0.3) +
  stat_cor(method = "pearson", label.y = 0.83, label.x = 70, color = "dodgerblue4") +
  ylab("Relative Volume (in %)") + xlab("Age (in years)") +
  theme_minimal() +
  ggtitle("RELATIVE VOLUME cerebellar peduncle") +
  theme(plot.title = element_text(hjust = 0.5))
Relative.Cerebellar.peduncles.Age.plot
ggsave("Relative.Cerebellar.peduncles.Age.plot.pdf", plot = Relative.Cerebellar.peduncles.Age.plot, width = 8, height = 6, units = "in", dpi = 600)  
 
 
   
 
 
  
Relative.Vermis.Age.plot <-  ggplot(Brainstem.Cerebellum.relative, aes(y=Vermis, x = Age))  +
  geom_point(aes(color = Gender), size = 1.5, alpha = 1, shape = 16) +
  scale_color_manual(values = c("chartreuse4", "orangered2")) +
  geom_smooth(method='lm', alpha = 0.2, colour = "dodgerblue4", size = 0.8, weight = 0.3) +
  geom_smooth(aes(color = Gender), method='lm', se = F, alpha = 0.2, linetype = "longdash", size = 0.3, weight = 0.3) +
  stat_cor(method = "pearson", label.y = 0.67, label.x = 70, color = "dodgerblue4") +
  ylab("Relative Volume (in %)") + xlab("Age (in years)") +
  theme_minimal() +
  ggtitle("RELATIVE VOLUME vermis") +
  theme(plot.title = element_text(hjust = 0.5))
Relative.Vermis.Age.plot
ggsave("Relative.Vermis.Age.plot.pdf", plot = Relative.Vermis.Age.plot, width = 8, height = 6, units = "in", dpi = 600)  
 
 
   
 
 
  
Relative.Hemisphere.Age.plot <-  ggplot(Brainstem.Cerebellum.relative, aes(y=Hemisphere, x = Age))  +
  geom_point(aes(color = Gender), size = 1.5, alpha = 1, shape = 16) +
  scale_color_manual(values = c("chartreuse4", "orangered2")) +
  geom_smooth(method='lm', alpha = 0.2, colour = "dodgerblue4", size = 0.8, weight = 0.3) +
  geom_smooth(aes(color = Gender), method='lm', se = F, alpha = 0.2, linetype = "longdash", size = 0.3, weight = 0.3) +
  stat_cor(method = "pearson", label.y = 11.7, label.x = 70, color = "dodgerblue4") +
  ylab("Relative Volume (in %)") + xlab("Age (in years)") +
  theme_minimal() +
  ggtitle("RELATIVE VOLUME hemisphere") +
  theme(plot.title = element_text(hjust = 0.5))
Relative.Hemisphere.Age.plot
ggsave("Relative.Hemisphere.Age.plot.pdf", plot = Relative.Hemisphere.Age.plot, width = 8, height = 6, units = "in", dpi = 600)  
 
 
   
 
 
  
Relative.Anterior.lobe.Age.plot <-  ggplot(Brainstem.Cerebellum.relative, aes(y=Anterior.lobe, x = Age))  +
  geom_point(aes(color = Gender), size = 1.5, alpha = 1, shape = 16) +
  scale_color_manual(values = c("chartreuse4", "orangered2")) +
  geom_smooth(method='lm', alpha = 0.2, colour = "dodgerblue4", size = 0.8, weight = 0.3) +
  geom_smooth(aes(color = Gender), method='lm', se = F, alpha = 0.2, linetype = "longdash", size = 0.3, weight = 0.3) +
  stat_cor(method = "pearson", label.y = 3.6, label.x = 70, color = "dodgerblue4") +
  ylab("Relative Volume (in %)") + xlab("Age (in years)") +
  theme_minimal() +
  ggtitle("RELATIVE VOLUME anterior lobe") +
  theme(plot.title = element_text(hjust = 0.5))
Relative.Anterior.lobe.Age.plot
ggsave("Relative.Anterior.lobe.Age.plot.pdf", plot = Relative.Anterior.lobe.Age.plot, width = 8, height = 6, units = "in", dpi = 600)  
 
 
   
 
 
  
Relative.Medial.lobe.Age.plot <-  ggplot(Brainstem.Cerebellum.relative, aes(y=Medial.lobe, x = Age))  +
  geom_point(aes(color = Gender), size = 1.5, alpha = 1, shape = 16) +
  scale_color_manual(values = c("chartreuse4", "orangered2")) +
  geom_smooth(method='lm', alpha = 0.2, colour = "dodgerblue4", size = 0.8, weight = 0.3) +
  geom_smooth(aes(color = Gender), method='lm', se = F, alpha = 0.2, linetype = "longdash", size = 0.3, weight = 0.3) +
  stat_cor(method = "pearson", label.y = 3.1, label.x = 70, color = "dodgerblue4") +
  ylab("Relative Volume (in %)") + xlab("Age (in years)") +
  theme_minimal() +
  ggtitle("RELATIVE VOLUME medial lobe") +
  theme(plot.title = element_text(hjust = 0.5))
Relative.Medial.lobe.Age.plot
ggsave("Relative.Medial.lobe.Age.plot.pdf", plot = Relative.Medial.lobe.Age.plot, width = 8, height = 6, units = "in", dpi = 600)  
 
 
   
 
 
  
Relative.Posterior.lobe.Age.plot <-  ggplot(Brainstem.Cerebellum.relative, aes(y=Posterior.lobe, x = Age))  +
  geom_point(aes(color = Gender), size = 1.5, alpha = 1, shape = 16) +
  scale_color_manual(values = c("chartreuse4", "orangered2")) +
  geom_smooth(method='lm', alpha = 0.2, colour = "dodgerblue4", size = 0.8, weight = 0.3) +
  geom_smooth(aes(color = Gender), method='lm', se = F, alpha = 0.2, linetype = "longdash", size = 0.3, weight = 0.3) +
  stat_cor(method = "pearson", label.y = 6.6, label.x = 70, color = "dodgerblue4") +
  ylab("Relative Volume (in %)") + xlab("Age (in years)") +
  theme_minimal() +
  ggtitle("RELATIVE VOLUME posterior lobe") +
  theme(plot.title = element_text(hjust = 0.5))
Relative.Posterior.lobe.Age.plot
ggsave("Relative.Posterior.lobe.Age.plot.pdf", plot = Relative.Posterior.lobe.Age.plot, width = 8, height = 6, units = "in", dpi = 600)  
 
 
   
 
 
  
Relative.Flocculonodular.lobe.Age.plot <-  ggplot(Brainstem.Cerebellum.relative, aes(y=Flocculonodular.lobe, x = Age))  +
  geom_point(aes(color = Gender), size = 1.5, alpha = 1, shape = 16) +
  scale_color_manual(values = c("chartreuse4", "orangered2")) +
  geom_smooth(method='lm', alpha = 0.2, colour = "dodgerblue4", size = 0.8, weight = 0.3) +
  geom_smooth(aes(color = Gender), method='lm', se = F, alpha = 0.2, linetype = "longdash", size = 0.3, weight = 0.3) +
  stat_cor(method = "pearson", label.y = 0.121, label.x = 70, color = "dodgerblue4") +
  ylab("Relative Volume (in %)") + xlab("Age (in years)") +
  theme_minimal() +
  ggtitle("RELATIVE VOLUME flocculonodular lobe") +
  theme(plot.title = element_text(hjust = 0.5))
Relative.Flocculonodular.lobe.Age.plot
ggsave("Relative.Flocculonodular.lobe.Age.plot.pdf", plot = Relative.Flocculonodular.lobe.Age.plot, width = 8, height = 6, units = "in", dpi = 600)  
 
 
   
 
 
  
Relative.Central.Age.plot <-  ggplot(Brainstem.Cerebellum.relative, aes(y=Central, x = Age))  +
  geom_point(aes(color = Gender), size = 1.5, alpha = 1, shape = 16) +
  scale_color_manual(values = c("chartreuse4", "orangered2")) +
  geom_smooth(method='lm', alpha = 0.2, colour = "dodgerblue4", linetype = "longdash", size = 0.5, weight = 0.3) +
  geom_smooth(aes(color = Gender), method='lm', se = F, alpha = 0.2, linetype = "longdash", size = 0.5, weight = 0.3) +
  stat_cor(method = "pearson", label.y = 0.072, label.x = 70, color = "dodgerblue4") +
  ylab("Relative Volume (in %)") + xlab("Age (in years)") +
  theme_minimal() +
  ggtitle("RELATIVE VOLUME CENTRAL") +
  theme(plot.title = element_text(hjust = 0.5))
Relative.Central.Age.plot
ggsave("Relative.Central.Age.plot.pdf", plot = Relative.Central.Age.plot, width = 8, height = 6, units = "in", dpi = 600)  
 
 
   
 
 
  
Relative.Culmen.Age.plot <-  ggplot(Brainstem.Cerebellum.relative, aes(y=Culmen, x = Age))  +
  geom_point(aes(color = Gender), size = 1.5, alpha = 1, shape = 16) +
  scale_color_manual(values = c("chartreuse4", "orangered2")) +
  geom_smooth(method='lm', alpha = 0.2, colour = "dodgerblue4", linetype = "longdash", size = 0.5, weight = 0.3) +
  geom_smooth(aes(color = Gender), method='lm', se = F, alpha = 0.2, linetype = "longdash", size = 0.5, weight = 0.3) +
  stat_cor(method = "pearson", label.y = 0.228, label.x = 70, color = "dodgerblue4") +
  ylab("Relative Volume (in %)") + xlab("Age (in years)") +
  theme_minimal() +
  ggtitle("RELATIVE VOLUME CULMEN") +
  theme(plot.title = element_text(hjust = 0.5))
Relative.Culmen.Age.plot
ggsave("Relative.Culmen.Age.plot.pdf", plot = Relative.Culmen.Age.plot, width = 8, height = 6, units = "in", dpi = 600)  
 
 
   
 
 
  
Relative.Declive.Age.plot <-  ggplot(Brainstem.Cerebellum.relative, aes(y=Declive, x = Age))  +
  geom_point(aes(color = Gender), size = 1.5, alpha = 1, shape = 16) +
  scale_color_manual(values = c("chartreuse4", "orangered2")) +
  geom_smooth(method='lm', alpha = 0.2, colour = "dodgerblue4", linetype = "longdash", size = 0.5, weight = 0.3) +
  geom_smooth(aes(color = Gender), method='lm', se = F, alpha = 0.2, linetype = "longdash", size = 0.5, weight = 0.3) +
  stat_cor(method = "pearson", label.y = 0.13, label.x = 70, color = "dodgerblue4") +
  ylab("Relative Volume (in %)") + xlab("Age (in years)") +
  theme_minimal() +
  ggtitle("RELATIVE VOLUME DECLIVE") +
  theme(plot.title = element_text(hjust = 0.5))
Relative.Declive.Age.plot
ggsave("Relative.Declive.Age.plot.pdf", plot = Relative.Declive.Age.plot, width = 8, height = 6, units = "in", dpi = 600)  
 
 
   
 
 
  
Relative.Folium.Age.plot <-  ggplot(Brainstem.Cerebellum.relative, aes(y=Folium, x = Age))  +
  geom_point(aes(color = Gender), size = 1.5, alpha = 1, shape = 16) +
  scale_color_manual(values = c("chartreuse4", "orangered2")) +
  geom_smooth(method='lm', alpha = 0.2, colour = "dodgerblue4", linetype = "longdash", size = 0.5, weight = 0.3) +
  geom_smooth(aes(color = Gender), method='lm', se = F, alpha = 0.2, linetype = "longdash", size = 0.5, weight = 0.3) +
  stat_cor(method = "pearson", label.y = 0.062, label.x = 70, color = "dodgerblue4") +
  ylab("Relative Volume (in %)") + xlab("Age (in years)") +
  theme_minimal() +
  ggtitle("RELATIVE VOLUME FOLIUM") +
  theme(plot.title = element_text(hjust = 0.5))
Relative.Folium.Age.plot
ggsave("Relative.Folium.Age.plot.pdf", plot = Relative.Folium.Age.plot, width = 8, height = 6, units = "in", dpi = 600)  
 
 
   
 
 
  
Relative.Tuber.Age.plot <-  ggplot(Brainstem.Cerebellum.relative, aes(y=Tuber, x = Age))  +
  geom_point(aes(color = Gender), size = 1.5, alpha = 1, shape = 16) +
  scale_color_manual(values = c("chartreuse4", "orangered2")) +
  geom_smooth(method='lm', alpha = 0.2, colour = "dodgerblue4", linetype = "longdash", size = 0.5, weight = 0.3) +
  geom_smooth(aes(color = Gender), method='lm', se = F, alpha = 0.2, linetype = "longdash", size = 0.5, weight = 0.3) +
  stat_cor(method = "pearson", label.y = 0.052, label.x = 70, color = "dodgerblue4") +
  ylab("Relative Volume (in %)") + xlab("Age (in years)") +
  theme_minimal() +
  ggtitle("RELATIVE VOLUME TUBER") +
  theme(plot.title = element_text(hjust = 0.5))
Relative.Tuber.Age.plot
ggsave("Relative.Tuber.Age.plot.pdf", plot = Relative.Tuber.Age.plot, width = 8, height = 6, units = "in", dpi = 600)  
 
 
   
 
 
  
Relative.Pyramid.Age.plot <-  ggplot(Brainstem.Cerebellum.relative, aes(y=Pyramid, x = Age))  +
  geom_point(aes(color = Gender), size = 1.5, alpha = 1, shape = 16) +
  scale_color_manual(values = c("chartreuse4", "orangered2")) +
  geom_smooth(method='lm', alpha = 0.2, colour = "dodgerblue4", linetype = "longdash", size = 0.5, weight = 0.3) +
  geom_smooth(aes(color = Gender), method='lm', se = F, alpha = 0.2, linetype = "longdash", size = 0.5, weight = 0.3) +
  stat_cor(method = "pearson", label.y = 0.062, label.x = 70, color = "dodgerblue4") +
  ylab("Relative Volume (in %)") + xlab("Age (in years)") +
  theme_minimal() +
  ggtitle("RELATIVE VOLUME PYRAMID") +
  theme(plot.title = element_text(hjust = 0.5))
Relative.Pyramid.Age.plot
ggsave("Relative.Pyramid.Age.plot.pdf", plot = Relative.Pyramid.Age.plot, width = 8, height = 6, units = "in", dpi = 600)  
 
 
   
 
 
  
Relative.Uvula.Age.plot <-  ggplot(Brainstem.Cerebellum.relative, aes(y=Uvula, x = Age))  +
  geom_point(aes(color = Gender), size = 1.5, alpha = 1, shape = 16) +
  scale_color_manual(values = c("chartreuse4", "orangered2")) +
  geom_smooth(method='lm', alpha = 0.2, colour = "dodgerblue4", linetype = "longdash", size = 0.5, weight = 0.3) +
  geom_smooth(aes(color = Gender), method='lm', se = F, alpha = 0.2, linetype = "longdash", size = 0.5, weight = 0.3) +
  stat_cor(method = "pearson", label.y = 0.097, label.x = 70, color = "dodgerblue4") +
  ylab("Relative Volume (in %)") + xlab("Age (in years)") +
  theme_minimal() +
  ggtitle("RELATIVE VOLUME UVULA") +
  theme(plot.title = element_text(hjust = 0.5))
Relative.Uvula.Age.plot
ggsave("Relative.Uvula.Age.plot.pdf", plot = Relative.Uvula.Age.plot, width = 8, height = 6, units = "in", dpi = 600)  
 
 
   
 
 
  
Relative.Nodule.Age.plot <-  ggplot(Brainstem.Cerebellum.relative, aes(y=Nodule, x = Age))  +
  geom_point(aes(color = Gender), size = 1.5, alpha = 1, shape = 16) +
  scale_color_manual(values = c("chartreuse4", "orangered2")) +
  geom_smooth(method='lm', alpha = 0.2, colour = "dodgerblue4", linetype = "longdash", size = 0.5, weight = 0.3) +
  geom_smooth(aes(color = Gender), method='lm', se = F, alpha = 0.2, linetype = "longdash", size = 0.5, weight = 0.3) +
  stat_cor(method = "pearson", label.y = 0.023, label.x = 70, color = "dodgerblue4") +
  ylab("Relative Volume (in %)") + xlab("Age (in years)") +
  theme_minimal() +
  ggtitle("RELATIVE VOLUME NODULE") +
  theme(plot.title = element_text(hjust = 0.5))
Relative.Nodule.Age.plot
ggsave("Relative.Nodule.Age.plot.pdf", plot = Relative.Nodule.Age.plot, width = 8, height = 6, units = "in", dpi = 600)  
 
 
   
 
 
  
Relative.Ala.lobuli.centralis.Age.plot <-  ggplot(Brainstem.Cerebellum.relative, aes(y=Ala.lobuli.centralis, x = Age))  +
  geom_point(aes(color = Gender), size = 1.5, alpha = 1, shape = 16) +
  scale_color_manual(values = c("chartreuse4", "orangered2")) +
  geom_smooth(method='lm', alpha = 0.2, colour = "dodgerblue4", linetype = "longdash", size = 0.5, weight = 0.3) +
  geom_smooth(aes(color = Gender), method='lm', se = F, alpha = 0.2, linetype = "longdash", size = 0.5, weight = 0.3) +
  stat_cor(method = "pearson", label.y = 1.45, label.x = 70, color = "dodgerblue4") +
  ylab("Relative Volume (in %)") + xlab("Age (in years)") +
  theme_minimal() +
  ggtitle("RELATIVE VOLUME ALA LOBULI CENTRALIS") +
  theme(plot.title = element_text(hjust = 0.5))
Relative.Ala.lobuli.centralis.Age.plot
ggsave("Relative.Ala.lobuli.centralis.Age.plot.pdf", plot = Relative.Ala.lobuli.centralis.Age.plot, width = 8, height = 6, units = "in", dpi = 600)  
 
 
   
 
 
  
Relative.AQL.Age.plot <-  ggplot(Brainstem.Cerebellum.relative, aes(y=AQL, x = Age))  +
  geom_point(aes(color = Gender), size = 1.5, alpha = 1, shape = 16) +
  scale_color_manual(values = c("chartreuse4", "orangered2")) +
  geom_smooth(method='lm', alpha = 0.2, colour = "dodgerblue4", size = 0.7, weight = 0.3) +
  geom_smooth(aes(color = Gender), method='lm', se = F, alpha = 0.2, linetype = "longdash", size = 0.5, weight = 0.3) +
  stat_cor(method = "pearson", label.y = 2.1, label.x = 70, color = "dodgerblue4") +
  ylab("Relative Volume (in %)") + xlab("Age (in years)") +
  theme_minimal() +
  ggtitle("RELATIVE VOLUME AQL") +
  theme(plot.title = element_text(hjust = 0.5))
Relative.AQL.Age.plot
ggsave("Relative.AQL.Age.plot.pdf", plot = Relative.AQL.Age.plot, width = 8, height = 6, units = "in", dpi = 600)  
 
 
   
 
 
  
Relative.PQL.Age.plot <-  ggplot(Brainstem.Cerebellum.relative, aes(y=PQL, x = Age))  +
  geom_point(aes(color = Gender), size = 1.5, alpha = 1, shape = 16) +
  scale_color_manual(values = c("chartreuse4", "orangered2")) +
  geom_smooth(method='lm', alpha = 0.2, colour = "dodgerblue4", linetype = "longdash", size = 0.5, weight = 0.3) +
  geom_smooth(aes(color = Gender), method='lm', se = F, alpha = 0.2, linetype = "longdash", size = 0.5, weight = 0.3) +
  stat_cor(method = "pearson", label.y = 1.55, label.x = 70, color = "dodgerblue4") +
  ylab("Relative Volume (in %)") + xlab("Age (in years)") +
  theme_minimal() +
  ggtitle("RELATIVE VOLUME PQL") +
  theme(plot.title = element_text(hjust = 0.5))
Relative.PQL.Age.plot
ggsave("Relative.PQL.Age.plot.pdf", plot = Relative.PQL.Age.plot, width = 8, height = 6, units = "in", dpi = 600)  
 
 
   
 
 
  
Relative.SSL.Age.plot <-  ggplot(Brainstem.Cerebellum.relative, aes(y=SSL, x = Age))  +
  geom_point(aes(color = Gender), size = 1.5, alpha = 1, shape = 16) +
  scale_color_manual(values = c("chartreuse4", "orangered2")) +
  geom_smooth(method='lm', alpha = 0.2, colour = "dodgerblue4", linetype = "longdash", size = 0.5, weight = 0.3) +
  geom_smooth(aes(color = Gender), method='lm', se = F, alpha = 0.2, linetype = "longdash", size = 0.5, weight = 0.3) +
  stat_cor(method = "pearson", label.y = 1.55, label.x = 70, color = "dodgerblue4") +
  ylab("Relative Volume (in %)") + xlab("Age (in years)") +
  theme_minimal() +
  ggtitle("RELATIVE VOLUME SSL") +
  theme(plot.title = element_text(hjust = 0.5))
Relative.SSL.Age.plot
ggsave("Relative.SSL.Age.plot.pdf", plot = Relative.SSL.Age.plot, width = 8, height = 6, units = "in", dpi = 600)  
 
 
   
 
 
  
Relative.ISL.gracile.Age.plot <-  ggplot(Brainstem.Cerebellum.relative, aes(y=ISL.gracile, x = Age))  +
  geom_point(aes(color = Gender), size = 1.5, alpha = 1, shape = 16) +
  scale_color_manual(values = c("chartreuse4", "orangered2")) +
  geom_smooth(method='lm', alpha = 0.2, colour = "dodgerblue4", linetype = "longdash", size = 0.5, weight = 0.3) +
  geom_smooth(aes(color = Gender), method='lm', se = F, alpha = 0.2, linetype = "longdash", size = 0.5, weight = 0.3) +
  stat_cor(method = "pearson", label.y = 4.2, label.x = 70, color = "dodgerblue4") +
  ylab("Relative Volume (in %)") + xlab("Age (in years)") +
  theme_minimal() +
  ggtitle("RELATIVE VOLUME ISL/GRACILE") +
  theme(plot.title = element_text(hjust = 0.5))
Relative.ISL.gracile.Age.plot
ggsave("Relative.ISL.gracile.Age.plot.pdf", plot = Relative.ISL.gracile.Age.plot, width = 8, height = 6, units = "in", dpi = 600)  
 
 
   
 
 
  
Relative.Biventer.Age.plot <-  ggplot(Brainstem.Cerebellum.relative, aes(y=Biventer, x = Age))  +
  geom_point(aes(color = Gender), size = 1.5, alpha = 1, shape = 16) +
  scale_color_manual(values = c("chartreuse4", "orangered2")) +
  geom_smooth(method='lm', alpha = 0.2, colour = "dodgerblue4", linetype = "longdash", size = 0.5, weight = 0.3) +
  geom_smooth(aes(color = Gender), method='lm', se = F, alpha = 0.2, linetype = "longdash", size = 0.5, weight = 0.3) +
  stat_cor(method = "pearson", label.y = 1.7, label.x = 70, color = "dodgerblue4") +
  ylab("Relative Volume (in %)") + xlab("Age (in years)") +
  theme_minimal() +
  ggtitle("RELATIVE VOLUME BIVENTER") +
  theme(plot.title = element_text(hjust = 0.5))
Relative.Biventer.Age.plot
ggsave("Relative.Biventer.Age.plot.pdf", plot = Relative.Biventer.Age.plot, width = 8, height = 6, units = "in", dpi = 600)  
 
 
   
 
 
  
Relative.Tonsilla.Age.plot <-  ggplot(Brainstem.Cerebellum.relative, aes(y=Tonsilla, x = Age))  +
  geom_point(aes(color = Gender), size = 1.5, alpha = 1, shape = 16) +
  scale_color_manual(values = c("chartreuse4", "orangered2")) +
  geom_smooth(method='lm', alpha = 0.2, colour = "dodgerblue4", linetype = "longdash", size = 0.5, weight = 0.3) +
  geom_smooth(aes(color = Gender), method='lm', se = F, alpha = 0.2, linetype = "longdash", size = 0.5, weight = 0.3) +
  stat_cor(method = "pearson", label.y = 0.72, label.x = 70, color = "dodgerblue4") +
  ylab("Relative Volume (in %)") + xlab("Age (in years)") +
  theme_minimal() +
  ggtitle("RELATIVE VOLUME TONSILLA") +
  theme(plot.title = element_text(hjust = 0.5))
Relative.Tonsilla.Age.plot
ggsave("Relative.Tonsilla.Age.plot.pdf", plot = Relative.Tonsilla.Age.plot, width = 8, height = 6, units = "in", dpi = 600)  
 
 
   
 
 
  
Relative.Flocculus.Age.plot <-  ggplot(Brainstem.Cerebellum.relative, aes(y=Flocculus, x = Age))  +
  geom_point(aes(color = Gender), size = 1.5, alpha = 1, shape = 16) +
  scale_color_manual(values = c("chartreuse4", "orangered2")) +
  geom_smooth(method='lm', alpha = 0.2, colour = "dodgerblue4", linetype = "longdash", size = 0.5, weight = 0.3) +
  geom_smooth(aes(color = Gender), method='lm', se = F, alpha = 0.2, linetype = "longdash", size = 0.5, weight = 0.3) +
  stat_cor(method = "pearson", label.y = 0.105, label.x = 70, color = "dodgerblue4") +
  ylab("Relative Volume (in %)") + xlab("Age (in years)") +
  theme_minimal() +
  ggtitle("RELATIVE VOLUME FLOCCULUS") +
  theme(plot.title = element_text(hjust = 0.5))
Relative.Flocculus.Age.plot
ggsave("Relative.Flocculus.Age.plot.pdf", plot = Relative.Flocculus.Age.plot, width = 8, height = 6, units = "in", dpi = 600)
  
 
 
   
 
 
 
 
 
 
 The Ventricular System 
 
 Absolute Volumes 
 
 
 
  
Total.volume.ventricles <- All.Volumes$`Total volume ventricles`
LV.total <-  All.Volumes$`Total volume lateral ventricles`
LV.frontal.horn <- All.Volumes$`Total volume frontal horn`
LV.body <- All.Volumes$`Total volume body of LV`
LV.atrium <- All.Volumes$`Total volume atrium`
LV.occipital.horn <- All.Volumes$`Total volume occipital horn`
LV.temporal.horn <- All.Volumes$`Total volume temporal horn`
Third.ventricle <- All.Volumes$`3rd ventricle`
Fourth.ventricle.total <- All.Volumes$`4th ventricle`
Apex <- All.Volumes$`Apex of 4th`
Lateral.recess <- All.Volumes$`Total volume lateral recess`
Obex <- All.Volumes$`Obex of 4th`
Fastigium <- All.Volumes$Fastigium

Ventricles.absolute <- as.data.frame(cbind(
Total.volume.ventricles,
LV.total,
LV.frontal.horn,
LV.body, 
LV.atrium, 
LV.occipital.horn, 
LV.temporal.horn, 
Third.ventricle, 
Fourth.ventricle.total,
Apex,
Lateral.recess,
Obex,
Fastigium
))

Ventricles.absolute$Gender <- All.Volumes$Gender

Table.Ventricles.absolute <- CreateTableOne(
  vars = c("Total.volume.ventricles",
"LV.total",
"LV.frontal.horn",
"LV.body", 
"LV.atrium", 
"LV.occipital.horn", 
"LV.temporal.horn", 
"Third.ventricle", 
"Fourth.ventricle.total",
"Apex",
"Lateral.recess",
"Obex",
"Fastigium"),
  data = Ventricles.absolute)

Table.Ventricles.absolute.stratified.gender <- CreateTableOne(
  vars = c("Total.volume.ventricles",
"LV.total",
"LV.frontal.horn",
"LV.body", 
"LV.atrium", 
"LV.occipital.horn", 
"LV.temporal.horn", 
"Third.ventricle", 
"Fourth.ventricle.total",
"Apex",
"Lateral.recess",
"Obex",
"Fastigium"),
strata = c("Gender"),
  data = Ventricles.absolute)

Table.Ventricles.absolute <- print(Table.Ventricles.absolute, contDigits = 10)  
 
 
                                       
                                      Overall                            
  n                                                 30                   
  Total.volume.ventricles (mean (SD)) 21184.5666666667 (16714.0388331514)
  LV.total (mean (SD))                18429.8333333333 (15998.6774174991)
  LV.frontal.horn (mean (SD))          6124.0666666667 (5138.9726528205) 
  LV.body (mean (SD))                  4852.4333333333 (4797.8072123759) 
  LV.atrium (mean (SD))                5499.3666666667 (5464.3878193472) 
  LV.occipital.horn (mean (SD))        1093.7000000000 (1013.8288691205) 
  LV.temporal.horn (mean (SD))          860.3333333333 (619.2339724560)  
  Third.ventricle (mean (SD))          1134.4000000000 (807.5322391344)  
  Fourth.ventricle.total (mean (SD))   1620.4000000000 (345.0322923418)  
  Apex (mean (SD))                      162.8333333333 (45.5911994936)   
  Lateral.recess (mean (SD))            198.6666666667 (46.2708169989)   
  Obex (mean (SD))                      174.4000000000 (109.4032717156)  
  Fastigium (mean (SD))                 199.3333333333 (53.8640263379)     
 
 
  Table.Ventricles.absolute.stratified.gender <- print(Table.Ventricles.absolute.stratified.gender, contDigits = 10)  
 
 
                                       Stratified by Gender
                                      f                                   m                                   p      test
  n                                                 14                                  16                               
  Total.volume.ventricles (mean (SD)) 19612.2857142857 (16045.3969552189) 22560.3125000000 (17681.6478029952)  0.638     
  LV.total (mean (SD))                17214.2857142857 (15961.2766090000) 19493.4375000000 (16476.3998614938)  0.704     
  LV.frontal.horn (mean (SD))          6001.6428571429 (5667.2323133437)   6231.1875000000 (4816.2291365583)   0.905     
  LV.body (mean (SD))                  4804.7857142857 (5434.6698035508)   4894.1250000000 (4347.6185492750)   0.960     
  LV.atrium (mean (SD))                4547.0714285714 (3755.7582347087)   6332.6250000000 (6626.9495031022)   0.381     
  LV.occipital.horn (mean (SD))        1175.2857142857 (1256.6319104025)   1022.3125000000 (779.0714745773)    0.688     
  LV.temporal.horn (mean (SD))          685.4285714286 (400.5451504917)    1013.3750000000 (740.7818279809)    0.151     
  Third.ventricle (mean (SD))           847.3571428571 (371.9043729742)    1385.5625000000 (998.3400201501)    0.068     
  Fourth.ventricle.total (mean (SD))   1550.7142857143 (294.6558432037)    1681.3750000000 (382.6409761992)    0.309     
  Apex (mean (SD))                      153.0000000000 (44.1047703682)      171.4375000000 (46.5130358072)     0.277     
  Lateral.recess (mean (SD))            195.2857142857 (45.7391072723)      201.6250000000 (48.0220435495)     0.715     
  Obex (mean (SD))                      179.5714285714 (109.0622364917)     169.8750000000 (113.0698161904)    0.813     
  Fastigium (mean (SD))                 194.2142857143 (54.4880771312)      203.8125000000 (54.6835974798)     0.635       
 
 
  write.csv(Table.Ventricles.absolute, "Table.Ventricles.absolute.csv")
write.csv(Table.Ventricles.absolute.stratified.gender, "Table.Ventricles.absolute.stratified.gender.csv")
  
 
 
 
 
 
 
  
Table.Ventricles.absolute.RSD <- as.data.frame(Table.Ventricles.absolute)
Table.Ventricles.absolute.RSD <- data.frame(do.call('rbind', 
                                                              strsplit(as.character(Table.Ventricles.absolute.RSD[-1,]),' (',fixed=TRUE)))
Table.Ventricles.absolute.RSD <- data.frame(cbind(str_replace_all(Table.Ventricles.absolute.RSD$X1, "[ ]", ""),
                                                            str_replace_all(Table.Ventricles.absolute.RSD$X2, "[)]", "")))
Table.Ventricles.absolute.RSD$X1 <- as.character(Table.Ventricles.absolute.RSD$X1)
Table.Ventricles.absolute.RSD$X2 <- as.character(Table.Ventricles.absolute.RSD$X2)
Table.Ventricles.absolute.RSD <- as.data.frame(sapply(Table.Ventricles.absolute.RSD, as.numeric))
Table.Ventricles.absolute.RSD <- as.data.frame(Table.Ventricles.absolute.RSD$X2/Table.Ventricles.absolute.RSD$X1)
Table.Ventricles.absolute.RSD <- round(Table.Ventricles.absolute.RSD * 100, 1)


Table.Ventricles.absolute.stratified.gender.RSD <- as.data.frame(Table.Ventricles.absolute.stratified.gender)
Table.Ventricles.absolute.stratified.gender.RSD <- select(Table.Ventricles.absolute.stratified.gender.RSD, - c(p, test))

Table.Ventricles.absolute.stratified.gender.RSD.female <- data.frame(do.call('rbind', 
                                                              strsplit(as.character(Table.Ventricles.absolute.stratified.gender.RSD[-1, "f"]),
                                                                       ' (',fixed=TRUE)))
Table.Ventricles.absolute.stratified.gender.RSD.female <- data.frame(cbind(str_replace_all(Table.Ventricles.absolute.stratified.gender.RSD.female$X1, "[ ]", ""),
                                                            str_replace_all(Table.Ventricles.absolute.stratified.gender.RSD.female$X2, "[)]", "")))
Table.Ventricles.absolute.stratified.gender.RSD.female$X1 <- as.character(Table.Ventricles.absolute.stratified.gender.RSD.female$X1)
Table.Ventricles.absolute.stratified.gender.RSD.female$X2 <- as.character(Table.Ventricles.absolute.stratified.gender.RSD.female$X2)
Table.Ventricles.absolute.stratified.gender.RSD.female <- as.data.frame(sapply(Table.Ventricles.absolute.stratified.gender.RSD.female, as.numeric))
Table.Ventricles.absolute.stratified.gender.RSD.female <- as.data.frame(Table.Ventricles.absolute.stratified.gender.RSD.female$X2/Table.Ventricles.absolute.stratified.gender.RSD.female$X1)
Table.Ventricles.absolute.stratified.gender.RSD.female <- round(Table.Ventricles.absolute.stratified.gender.RSD.female * 100, 1)

Table.Ventricles.absolute.stratified.gender.RSD.male <- data.frame(do.call('rbind', 
                                                              strsplit(as.character(Table.Ventricles.absolute.stratified.gender.RSD[-1, "m"]),
                                                                       ' (',fixed=TRUE)))
Table.Ventricles.absolute.stratified.gender.RSD.male <- data.frame(cbind(str_replace_all(Table.Ventricles.absolute.stratified.gender.RSD.male$X1, "[ ]", ""),
                                                            str_replace_all(Table.Ventricles.absolute.stratified.gender.RSD.male$X2, "[)]", "")))
Table.Ventricles.absolute.stratified.gender.RSD.male$X1 <- as.character(Table.Ventricles.absolute.stratified.gender.RSD.male$X1)
Table.Ventricles.absolute.stratified.gender.RSD.male$X2 <- as.character(Table.Ventricles.absolute.stratified.gender.RSD.male$X2)
Table.Ventricles.absolute.stratified.gender.RSD.male <- as.data.frame(sapply(Table.Ventricles.absolute.stratified.gender.RSD.male, as.numeric))
Table.Ventricles.absolute.stratified.gender.RSD.male <- as.data.frame(Table.Ventricles.absolute.stratified.gender.RSD.male$X2/Table.Ventricles.absolute.stratified.gender.RSD.male$X1)
Table.Ventricles.absolute.stratified.gender.RSD.male <- round(Table.Ventricles.absolute.stratified.gender.RSD.male * 100, 1)
  
 
 
 
 
 
 
  
kable(Table.Ventricles.absolute)  
 
 
 

 
 
 
  
 Overall 
 
 
 
 
 n 
 30 
 
 
 Total.volume.ventricles (mean (SD)) 
 21184.5666666667 (16714.0388331514) 
 
 
 LV.total (mean (SD)) 
 18429.8333333333 (15998.6774174991) 
 
 
 LV.frontal.horn (mean (SD)) 
 6124.0666666667 (5138.9726528205) 
 
 
 LV.body (mean (SD)) 
 4852.4333333333 (4797.8072123759) 
 
 
 LV.atrium (mean (SD)) 
 5499.3666666667 (5464.3878193472) 
 
 
 LV.occipital.horn (mean (SD)) 
 1093.7000000000 (1013.8288691205) 
 
 
 LV.temporal.horn (mean (SD)) 
 860.3333333333 (619.2339724560) 
 
 
 Third.ventricle (mean (SD)) 
 1134.4000000000 (807.5322391344) 
 
 
 Fourth.ventricle.total (mean (SD)) 
 1620.4000000000 (345.0322923418) 
 
 
 Apex (mean (SD)) 
 162.8333333333 (45.5911994936) 
 
 
 Lateral.recess (mean (SD)) 
 198.6666666667 (46.2708169989) 
 
 
 Obex (mean (SD)) 
 174.4000000000 (109.4032717156) 
 
 
 Fastigium (mean (SD)) 
 199.3333333333 (53.8640263379) 
 
 
 


 
   
  kable(Table.Ventricles.absolute.RSD)  
 
 
 

 
 
 
 Table.Ventricles.absolute.RSD  X 2/ T  a  b  l  e . V  e  n  t  r  i  c  l  e  s . a  b  s  o  l  u  t  e . R  S  D  X1 
 
 
 
 
 78.9 
 
 
 86.8 
 
 
 83.9 
 
 
 98.9 
 
 
 99.4 
 
 
 92.7 
 
 
 72.0 
 
 
 71.2 
 
 
 21.3 
 
 
 28.0 
 
 
 23.3 
 
 
 62.7 
 
 
 27.0 
 
 
 


 
   
  kable(Table.Ventricles.absolute.stratified.gender)  
 
 
 

 
 
 
 
 
 
 
 
 
 
  
 f 
 m 
 p 
 test 
 
 
 
 
 n 
 14 
 16 
  
  
 
 
 Total.volume.ventricles (mean (SD)) 
 19612.2857142857 (16045.3969552189) 
 22560.3125000000 (17681.6478029952) 
 0.638 
  
 
 
 LV.total (mean (SD)) 
 17214.2857142857 (15961.2766090000) 
 19493.4375000000 (16476.3998614938) 
 0.704 
  
 
 
 LV.frontal.horn (mean (SD)) 
 6001.6428571429 (5667.2323133437) 
 6231.1875000000 (4816.2291365583) 
 0.905 
  
 
 
 LV.body (mean (SD)) 
 4804.7857142857 (5434.6698035508) 
 4894.1250000000 (4347.6185492750) 
 0.960 
  
 
 
 LV.atrium (mean (SD)) 
 4547.0714285714 (3755.7582347087) 
 6332.6250000000 (6626.9495031022) 
 0.381 
  
 
 
 LV.occipital.horn (mean (SD)) 
 1175.2857142857 (1256.6319104025) 
 1022.3125000000 (779.0714745773) 
 0.688 
  
 
 
 LV.temporal.horn (mean (SD)) 
 685.4285714286 (400.5451504917) 
 1013.3750000000 (740.7818279809) 
 0.151 
  
 
 
 Third.ventricle (mean (SD)) 
 847.3571428571 (371.9043729742) 
 1385.5625000000 (998.3400201501) 
 0.068 
  
 
 
 Fourth.ventricle.total (mean (SD)) 
 1550.7142857143 (294.6558432037) 
 1681.3750000000 (382.6409761992) 
 0.309 
  
 
 
 Apex (mean (SD)) 
 153.0000000000 (44.1047703682) 
 171.4375000000 (46.5130358072) 
 0.277 
  
 
 
 Lateral.recess (mean (SD)) 
 195.2857142857 (45.7391072723) 
 201.6250000000 (48.0220435495) 
 0.715 
  
 
 
 Obex (mean (SD)) 
 179.5714285714 (109.0622364917) 
 169.8750000000 (113.0698161904) 
 0.813 
  
 
 
 Fastigium (mean (SD)) 
 194.2142857143 (54.4880771312) 
 203.8125000000 (54.6835974798) 
 0.635 
  
 
 
 


 
   
  kable(Table.Ventricles.absolute.stratified.gender.RSD.female)  
 
 
 

 
 
 
 
 
 
 Table.Ventricles.absolute.stratified.gender.RSD.female  X 2/ T  a  b  l  e . V  e  n  t  r  i  c  l  e  s . a  b  s  o  l  u  t  e . s  t  r  a  t  i  f  i  e  d . g  e  n  d  e  r . R  S  D . f  e  m  a  l  e  X1 
 
 
 
 
 81.8 
 
 
 92.7 
 
 
 94.4 
 
 
 113.1 
 
 
 82.6 
 
 
 106.9 
 
 
 58.4 
 
 
 43.9 
 
 
 19.0 
 
 
 28.8 
 
 
 23.4 
 
 
 60.7 
 
 
 28.1 
 
 
 


 
   
  kable(Table.Ventricles.absolute.stratified.gender.RSD.male)  
 
 
 

 
 
 
 
 
 
 Table.Ventricles.absolute.stratified.gender.RSD.male  X 2/ T  a  b  l  e . V  e  n  t  r  i  c  l  e  s . a  b  s  o  l  u  t  e . s  t  r  a  t  i  f  i  e  d . g  e  n  d  e  r . R  S  D . m  a  l  e  X1 
 
 
 
 
 78.4 
 
 
 84.5 
 
 
 77.3 
 
 
 88.8 
 
 
 104.6 
 
 
 76.2 
 
 
 73.1 
 
 
 72.1 
 
 
 22.8 
 
 
 27.1 
 
 
 23.8 
 
 
 66.6 
 
 
 26.8 
 
 
 


 
   
  NA  
 
 
 
 
 
 
  
Ventricles.absolute <- select(Ventricles.absolute, - c(Gender))
Ventricles.absolute.red <- select(Ventricles.absolute, - c(Total.volume.ventricles))

names.anatomical.structures.temporary <- c("LV.total",
"LV.frontal.horn",
"LV.body", 
"LV.atrium", 
"LV.occipital.horn", 
"LV.temporal.horn", 
"Third.ventricle", 
"Fourth.ventricle.total",
"Apex",
"Lateral.recess",
"Obex",
"Fastigium")

names.anatomical.structures.definitive <- c("LV- Total",
"LV - Frontal horn",
"LV - Body", 
"LV - Atrium", 
"LV - Occipital horn", 
"LV - Temporal horn", 
"Third ventricle", 
"Fourth ventricle - Total",
"Apex",
"Lateral recess",
"Obex",
"Fastigium")

Ventricles.absolute.plotdata <- gather(Ventricles.absolute.red, "anatomical.structure", "relative.volume")
Ventricles.absolute.plotdata$Gender <- All.Volumes$Gender
Ventricles.absolute.plotdata$Age <- All.Volumes$`Age (years)`

Ventricles.absolute.plotdata$Gender <- factor(Ventricles.absolute.plotdata$Gender, levels = c("f", "m"), c("f", "m"))
Ventricles.absolute.plotdata$anatomical.structure <- factor(Ventricles.absolute.plotdata$anatomical.structure, 
                                                                levels = rev(c(names.anatomical.structures.temporary)), rev(c(names.anatomical.structures.definitive)))

Ventricles.absolute.plot <-  ggplot(Ventricles.absolute.plotdata, aes(x=anatomical.structure, y = relative.volume))  +
  stat_summary(alpha = 0.3, fun = mean, geom = "bar", width = 0.3, fill = "gray50") + 
  geom_boxplot(aes(fill = Gender), alpha = 0.5, width = 0.4, size = 0.2, position = position_dodge(width = 0.6), 
               outlier.shape = NA, color = "gray30") +
  scale_fill_manual(values = c("chartreuse4", "orangered2")) +
  geom_quasirandom(aes(color = Age), size = 0.7, alpha = 0.8, shape = 16, position = "dodge") +
  scale_color_continuous(low = "steelblue1", high = "red4") +
  xlab("") + ylab("Absolute volume (in mm3)") +
  theme_minimal() +
  coord_flip() +
  ggtitle("VENTRICULAR SYSTEM") +
  theme(plot.title = element_text(hjust = 0.5))

Ventricles.absolute.plot
ggsave("Ventricles.absolute.plot.pdf", plot = Ventricles.absolute.plot, width = 12, height = 5, units = "in", dpi = 600)
  
 
 
   
 
 
 
 
 
 
  
Total.ventricles.Age.plot <-  ggplot(All.Volumes, aes(y=`Total volume ventricles`, x = `Age (years)`))  +
  geom_point(aes(color = Gender), size = 1.5, alpha = 1, shape = 16) +
  scale_color_manual(values = c("chartreuse4", "orangered2")) +
  geom_smooth(method='lm', alpha = 0.2, colour = "dodgerblue4", size = 0.8, weight = 0.3) +
  geom_smooth(aes(color = Gender), method='lm', se = F, alpha = 0.2, linetype = "longdash", size = 0.3, weight = 0.3) +
  stat_cor(method = "pearson", label.y = 82000, label.x = 70, color = "dodgerblue4") +
  ylab("Volume in mm3") + xlab("Age (in years)") +
  theme_minimal() +
  ggtitle("ABSOLUTE VOLUME total ventricular system") +
  theme(plot.title = element_text(hjust = 0.5))
Total.ventricles.Age.plot
ggsave("Total.Total.ventricles.Age.plot.pdf", plot = Total.ventricles.Age.plot, width = 8, height = 6, units = "in", dpi = 600)  
 
 
   
 
 
  
Lateral.ventricle.Age.plot <- ggplot(All.Volumes, aes(y=`Total volume lateral ventricles`, x = `Age (years)`))  +
  geom_point(aes(color = Gender), size = 1.5, alpha = 1, shape = 16) +
  scale_color_manual(values = c("chartreuse4", "orangered2")) +
  geom_smooth(method='lm', alpha = 0.2, colour = "dodgerblue4", size = 0.8, weight = 0.3) +
  geom_smooth(aes(color = Gender), method='lm', se = F, alpha = 0.2, linetype = "longdash", size = 0.3, weight = 0.3) +
  stat_cor(method = "pearson", label.y = 72000, label.x = 65, color = "dodgerblue4") +
  ylab("Volume in mm3") + xlab("Age (in years)") +
  theme_minimal() +
  ggtitle("ABSOLUTE VOLUME lateral ventricles") +
  theme(plot.title = element_text(hjust = 0.5))
Lateral.ventricle.Age.plot
ggsave("Total.Lateral.ventricle.Age.plot.pdf", plot = Lateral.ventricle.Age.plot, width = 8, height = 6, units = "in", dpi = 600)  
 
 
   
 
 
  
Lateral.ventricle.frontal.horn.Age.plot <- ggplot(All.Volumes, aes(y=`Total volume frontal horn`, x = `Age (years)`))  +
  geom_point(aes(color = Gender), size = 1.5, alpha = 1, shape = 16) +
  scale_color_manual(values = c("chartreuse4", "orangered2")) +
  geom_smooth(method='lm', alpha = 0.2, colour = "dodgerblue4", size = 0.8, weight = 0.3) +
  geom_smooth(aes(color = Gender), method='lm', se = F, alpha = 0.2, linetype = "longdash", size = 0.3, weight = 0.3) +
  stat_cor(method = "pearson", label.y = 21000, label.x = 65, color = "dodgerblue4") +
  ylab("Volume in mm3") + xlab("Age (in years)") +
  theme_minimal() +
  ggtitle("ABSOLUTE VOLUME frontal horn") +
  theme(plot.title = element_text(hjust = 0.5))
Lateral.ventricle.frontal.horn.Age.plot
ggsave("Total.Lateral.ventricle.frontal.horn.Age.plot.pdf", plot = Lateral.ventricle.frontal.horn.Age.plot, width = 8, height = 6, units = "in", dpi = 600)  
 
 
   
 
 
  
Lateral.ventricle.body.Age.plot <- ggplot(All.Volumes, aes(y=`Total volume body of LV`, x = `Age (years)`))  +
  geom_point(aes(color = Gender), size = 1.5, alpha = 1, shape = 16) +
  scale_color_manual(values = c("chartreuse4", "orangered2")) +
  geom_smooth(method='lm', alpha = 0.2, colour = "dodgerblue4", size = 0.8, weight = 0.3) +
  geom_smooth(aes(color = Gender), method='lm', se = F, alpha = 0.2, linetype = "longdash", size = 0.3, weight = 0.3) +
  stat_cor(method = "pearson", label.y = 19000, label.x = 65, color = "dodgerblue4") +
  ylab("Volume in mm3") + xlab("Age (in years)") +
  theme_minimal() +
  ggtitle("ABSOLUTE VOLUME body") +
  theme(plot.title = element_text(hjust = 0.5))
Lateral.ventricle.body.Age.plot
ggsave("Total.Lateral.ventricle.body.Age.plot.pdf", plot = Lateral.ventricle.body.Age.plot, width = 8, height = 6, units = "in", dpi = 600)  
 
 
   
 
 
  
Lateral.ventricle.atrium.Age.plot <- ggplot(All.Volumes, aes(y=`Total volume atrium`, x = `Age (years)`))  +
  geom_point(aes(color = Gender), size = 1.5, alpha = 1, shape = 16) +
  scale_color_manual(values = c("chartreuse4", "orangered2")) +
  geom_smooth(method='lm', alpha = 0.2, colour = "dodgerblue4", size = 0.8, weight = 0.3) +
  geom_smooth(aes(color = Gender), method='lm', se = F, alpha = 0.2, linetype = "longdash", size = 0.3, weight = 0.3) +
  stat_cor(method = "pearson", label.y = 24000, label.x = 65, color = "dodgerblue4") +
  ylab("Volume in mm3") + xlab("Age (in years)") +
  theme_minimal() +
  ggtitle("ABSOLUTE VOLUME atrium") +
  theme(plot.title = element_text(hjust = 0.5))
Lateral.ventricle.atrium.Age.plot
ggsave("Total.Lateral.ventricle.atrium.Age.plot.pdf", plot = Lateral.ventricle.atrium.Age.plot, width = 8, height = 6, units = "in", dpi = 600)  
 
 
   
 
 
  
Lateral.ventricle.occipital.horn.Age.plot <- ggplot(All.Volumes, aes(y=`Total volume occipital horn`, x = `Age (years)`))  +
  geom_point(aes(color = Gender), size = 1.5, alpha = 1, shape = 16) +
  scale_color_manual(values = c("chartreuse4", "orangered2")) +
  geom_smooth(method='lm', alpha = 0.2, colour = "dodgerblue4", size = 0.8, weight = 0.3) +
  geom_smooth(aes(color = Gender), method='lm', se = F, alpha = 0.2, linetype = "longdash", size = 0.3, weight = 0.3) +
  stat_cor(method = "pearson", label.y = 4100, label.x = 65, color = "dodgerblue4") +
  ylab("Volume in mm3") + xlab("Age (in years)") +
  theme_minimal() +
  ggtitle("ABSOLUTE VOLUME occipital horn") +
  theme(plot.title = element_text(hjust = 0.5))
Lateral.ventricle.occipital.horn.Age.plot
ggsave("Total.Lateral.ventricle.occipital.horn.Age.plot.pdf", plot = Lateral.ventricle.occipital.horn.Age.plot, width = 8, height = 6, units = "in", dpi = 600)  
 
 
   
 
 
  
Lateral.ventricle.temporal.horn.Age.plot <- ggplot(All.Volumes, aes(y=`Total volume temporal horn`, x = `Age (years)`))  +
  geom_point(aes(color = Gender), size = 1.5, alpha = 1, shape = 16) +
  scale_color_manual(values = c("chartreuse4", "orangered2")) +
  geom_smooth(method='lm', alpha = 0.2, colour = "dodgerblue4", size = 0.8, weight = 0.3) +
  geom_smooth(aes(color = Gender), method='lm', se = F, alpha = 0.2, linetype = "longdash", size = 0.3, weight = 0.3) +
  stat_cor(method = "pearson", label.y = 3100, label.x = 65, color = "dodgerblue4") +
  ylab("Volume in mm3") + xlab("Age (in years)") +
  theme_minimal() +
  ggtitle("ABSOLUTE VOLUME temporal horn") +
  theme(plot.title = element_text(hjust = 0.5))
Lateral.ventricle.temporal.horn.Age.plot
ggsave("Total.Lateral.ventricle.temporal.horn.Age.plot.pdf", plot = Lateral.ventricle.temporal.horn.Age.plot, width = 8, height = 6, units = "in", dpi = 600)  
 
 
   
 
 
  
Third.ventricle.Age.plot <- ggplot(All.Volumes, aes(y =`3rd ventricle`, x = `Age (years)`))  +
  geom_point(aes(color = Gender), size = 1.5, alpha = 1, shape = 16) +
  scale_color_manual(values = c("chartreuse4", "orangered2")) +
  geom_smooth(method='lm', alpha = 0.2, colour = "dodgerblue4", size = 0.8, weight = 0.3) +
  geom_smooth(aes(color = Gender), method='lm', se = F, alpha = 0.2, linetype = "longdash", size = 0.3, weight = 0.3) +
  stat_cor(method = "pearson", label.y = 4250, label.x = 70, color = "dodgerblue4") +
  ylab("Volume in mm3") + xlab("Age (in years)") +
  theme_minimal() +
  ggtitle("ABSOLUTE VOLUME third ventricle") +
  theme(plot.title = element_text(hjust = 0.5))
Third.ventricle.Age.plot
ggsave("Total.Third.ventricle.Age.plot.pdf", plot = Third.ventricle.Age.plot, width = 8, height = 6, units = "in", dpi = 600)  
 
 
   
 
 
  
Fourth.ventricle.Age.plot <- ggplot(All.Volumes, aes(y =`4th ventricle`, x = `Age (years)`))  +
  geom_point(aes(color = Gender), size = 1.5, alpha = 1, shape = 16) +
  scale_color_manual(values = c("chartreuse4", "orangered2")) +
  geom_smooth(method='lm', alpha = 0.2, colour = "dodgerblue4", size = 0.8, weight = 0.3) +
  geom_smooth(aes(color = Gender), method='lm', se = F, alpha = 0.2, linetype = "longdash", size = 0.3, weight = 0.3) +
  stat_cor(method = "pearson", label.y = 2700, label.x = 70, color = "dodgerblue4") +
  ylab("Volume in mm3") + xlab("Age (in years)") +
  theme_minimal() +
  ggtitle("ABSOLUTE VOLUME fourth ventricle") +
  theme(plot.title = element_text(hjust = 0.5))
Fourth.ventricle.Age.plot
ggsave("Total.Fourth.ventricle.Age.plot.pdf", plot = Fourth.ventricle.Age.plot, width = 8, height = 6, units = "in", dpi = 600)
  
 
 
   
 
 
 
 
 
 Relative Volumes 1: Normalized to Total Encephalic Volume 
 
 
 
  
#Ventricles.absolute <- select(Ventricles.absolute, - c(Gender))
Total.ventricular.volume.relative <- (100 * (Ventricles.absolute$Total.volume.ventricles/All.Volumes$`Total encephalic volume (without ventricles)`))

Ventricles.relative <- (100 * (Ventricles.absolute[, -1]/All.Volumes$`Total encephalic volume (without ventricles)`))

Table.Ventricles.relative <- cbind(Total.ventricular.volume.relative, Ventricles.relative)

Table.Ventricles.relative$Gender <- All.Volumes$Gender

Table.Ventricles.relative1 <- CreateTableOne(
  vars = c("Total.ventricular.volume.relative",
"LV.total",
"LV.frontal.horn",
"LV.body", 
"LV.atrium", 
"LV.occipital.horn", 
"LV.temporal.horn", 
"Third.ventricle", 
"Fourth.ventricle.total",
"Apex",
"Lateral.recess",
"Obex",
"Fastigium"),
  data = Table.Ventricles.relative)

Table.Ventricles.relative.stratified.gender <- CreateTableOne(
  vars = c("Total.ventricular.volume.relative",
"LV.total",
"LV.frontal.horn",
"LV.body", 
"LV.atrium", 
"LV.occipital.horn", 
"LV.temporal.horn", 
"Third.ventricle", 
"Fourth.ventricle.total",
"Apex",
"Lateral.recess",
"Obex",
"Fastigium"),
strata = c("Gender"),
  data = Table.Ventricles.relative)

Table.Ventricles.relative1 <- print(Table.Ventricles.relative1, contDigits = 10)  
 
 
                                                 
                                                Overall                    
  n                                                       30               
  Total.ventricular.volume.relative (mean (SD)) 1.9273127232 (1.5160752528)
  LV.total (mean (SD))                          1.6743917636 (1.4458630666)
  LV.frontal.horn (mean (SD))                   0.5544339978 (0.4457583254)
  LV.body (mean (SD))                           0.4411089345 (0.4277559363)
  LV.atrium (mean (SD))                         0.4999720439 (0.5129573777)
  LV.occipital.horn (mean (SD))                 0.0992362568 (0.0919251551)
  LV.temporal.horn (mean (SD))                  0.0796461516 (0.0609277346)
  Third.ventricle (mean (SD))                   0.1036226244 (0.0756082790)
  Fourth.ventricle.total (mean (SD))            0.1493055163 (0.0331934574)
  Apex (mean (SD))                              0.0149830331 (0.0042490310)
  Lateral.recess (mean (SD))                    0.0182578178 (0.0041320330)
  Obex (mean (SD))                              0.0159650951 (0.0097282418)
  Fastigium (mean (SD))                         0.0183133305 (0.0048392615)  
 
 
  Table.Ventricles.relative.stratified.gender <- print(Table.Ventricles.relative.stratified.gender, contDigits = 10)  
 
 
                                                 Stratified by Gender
                                                f                           m                           p      test
  n                                                       14                          16                           
  Total.ventricular.volume.relative (mean (SD)) 1.8554635462 (1.3268579426) 1.9901807530 (1.7055469213)  0.813     
  LV.total (mean (SD))                          1.6208567323 (1.3239504826) 1.7212349160 (1.5866708182)  0.853     
  LV.frontal.horn (mean (SD))                   0.5647158943 (0.4689867048) 0.5454373384 (0.4397126405)  0.908     
  LV.body (mean (SD))                           0.4491770751 (0.4459306767) 0.4340493115 (0.4257907279)  0.925     
  LV.atrium (mean (SD))                         0.4293273395 (0.3142686102) 0.5617861603 (0.6437228077)  0.490     
  LV.occipital.horn (mean (SD))                 0.1112826435 (0.1145831457) 0.0886956685 (0.0685889424)  0.512     
  LV.temporal.horn (mean (SD))                  0.0663469710 (0.0375726047) 0.0912829346 (0.0751259289)  0.271     
  Third.ventricle (mean (SD))                   0.0826068089 (0.0357482716) 0.1220114630 (0.0957687648)  0.158     
  Fourth.ventricle.total (mean (SD))            0.1520088916 (0.0289166876) 0.1469400629 (0.0373186717)  0.684     
  Apex (mean (SD))                              0.0150095263 (0.0043100506) 0.0149598516 (0.0043363600)  0.975     
  Lateral.recess (mean (SD))                    0.0190608542 (0.0040699140) 0.0175551609 (0.0041862858)  0.328     
  Obex (mean (SD))                              0.0174969331 (0.0102282698) 0.0146247369 (0.0093911238)  0.429     
  Fastigium (mean (SD))                         0.0189991246 (0.0050327628) 0.0177132608 (0.0047435307)  0.477       
 
 
 
 
 
 
  
Table.Ventricles.relative1.RSD <- as.data.frame(Table.Ventricles.relative1)
Table.Ventricles.relative1.RSD <- data.frame(do.call('rbind', 
                                                              strsplit(as.character(Table.Ventricles.relative1.RSD[-1,]),
                                                                       ' (',fixed=TRUE)))
Table.Ventricles.relative1.RSD <- data.frame(cbind(str_replace_all(Table.Ventricles.relative1.RSD$X1, "[ ]", ""),
                                                            str_replace_all(Table.Ventricles.relative1.RSD$X2, "[)]", "")))
Table.Ventricles.relative1.RSD$X1 <- as.character(Table.Ventricles.relative1.RSD$X1)
Table.Ventricles.relative1.RSD$X2 <- as.character(Table.Ventricles.relative1.RSD$X2)
Table.Ventricles.relative1.RSD <- as.data.frame(sapply(Table.Ventricles.relative1.RSD, as.numeric))
Table.Ventricles.relative1.RSD <- as.data.frame(Table.Ventricles.relative1.RSD$X2/Table.Ventricles.relative1.RSD$X1)
Table.Ventricles.relative1.RSD <- round(Table.Ventricles.relative1.RSD * 100, 1)


Table.Ventricles.relative.stratified.gender.RSD <- as.data.frame(Table.Ventricles.relative.stratified.gender)
Table.Ventricles.relative.stratified.gender.RSD <- select(Table.Ventricles.relative.stratified.gender.RSD, - c(p, test))

Table.Ventricles.relative.stratified.gender.RSD.female <- data.frame(do.call('rbind', 
                                                              strsplit(as.character(Table.Ventricles.relative.stratified.gender.RSD[-1, "f"]),
                                                                       ' (',fixed=TRUE)))
Table.Ventricles.relative.stratified.gender.RSD.female <- data.frame(cbind(str_replace_all(Table.Ventricles.relative.stratified.gender.RSD.female$X1, "[ ]", ""),
                                                            str_replace_all(Table.Ventricles.relative.stratified.gender.RSD.female$X2, "[)]", "")))
Table.Ventricles.relative.stratified.gender.RSD.female$X1 <- as.character(Table.Ventricles.relative.stratified.gender.RSD.female$X1)
Table.Ventricles.relative.stratified.gender.RSD.female$X2 <- as.character(Table.Ventricles.relative.stratified.gender.RSD.female$X2)
Table.Ventricles.relative.stratified.gender.RSD.female <- as.data.frame(sapply(Table.Ventricles.relative.stratified.gender.RSD.female, as.numeric))
Table.Ventricles.relative.stratified.gender.RSD.female <- as.data.frame(Table.Ventricles.relative.stratified.gender.RSD.female$X2/Table.Ventricles.relative.stratified.gender.RSD.female$X1)
Table.Ventricles.relative.stratified.gender.RSD.female <- round(Table.Ventricles.relative.stratified.gender.RSD.female * 100, 1)

Table.Ventricles.relative.stratified.gender.RSD.male <- data.frame(do.call('rbind', 
                                                              strsplit(as.character(Table.Ventricles.relative.stratified.gender.RSD[-1, "m"]),
                                                                       ' (',fixed=TRUE)))
Table.Ventricles.relative.stratified.gender.RSD.male <- data.frame(cbind(str_replace_all(Table.Ventricles.relative.stratified.gender.RSD.male$X1, "[ ]", ""),
                                                            str_replace_all(Table.Ventricles.relative.stratified.gender.RSD.male$X2, "[)]", "")))
Table.Ventricles.relative.stratified.gender.RSD.male$X1 <- as.character(Table.Ventricles.relative.stratified.gender.RSD.male$X1)
Table.Ventricles.relative.stratified.gender.RSD.male$X2 <- as.character(Table.Ventricles.relative.stratified.gender.RSD.male$X2)
Table.Ventricles.relative.stratified.gender.RSD.male <- as.data.frame(sapply(Table.Ventricles.relative.stratified.gender.RSD.male, as.numeric))
Table.Ventricles.relative.stratified.gender.RSD.male <- as.data.frame(Table.Ventricles.relative.stratified.gender.RSD.male$X2/Table.Ventricles.relative.stratified.gender.RSD.male$X1)
Table.Ventricles.relative.stratified.gender.RSD.male <- round(Table.Ventricles.relative.stratified.gender.RSD.male * 100, 1)
  
 
 
 
 
 
 
  
kable(Table.Ventricles.relative1)  
 
 
 

 
 
 
  
 Overall 
 
 
 
 
 n 
 30 
 
 
 Total.ventricular.volume.relative (mean (SD)) 
 1.9273127232 (1.5160752528) 
 
 
 LV.total (mean (SD)) 
 1.6743917636 (1.4458630666) 
 
 
 LV.frontal.horn (mean (SD)) 
 0.5544339978 (0.4457583254) 
 
 
 LV.body (mean (SD)) 
 0.4411089345 (0.4277559363) 
 
 
 LV.atrium (mean (SD)) 
 0.4999720439 (0.5129573777) 
 
 
 LV.occipital.horn (mean (SD)) 
 0.0992362568 (0.0919251551) 
 
 
 LV.temporal.horn (mean (SD)) 
 0.0796461516 (0.0609277346) 
 
 
 Third.ventricle (mean (SD)) 
 0.1036226244 (0.0756082790) 
 
 
 Fourth.ventricle.total (mean (SD)) 
 0.1493055163 (0.0331934574) 
 
 
 Apex (mean (SD)) 
 0.0149830331 (0.0042490310) 
 
 
 Lateral.recess (mean (SD)) 
 0.0182578178 (0.0041320330) 
 
 
 Obex (mean (SD)) 
 0.0159650951 (0.0097282418) 
 
 
 Fastigium (mean (SD)) 
 0.0183133305 (0.0048392615) 
 
 
 


 
   
  kable(Table.Ventricles.relative1.RSD)  
 
 
 

 
 
 
 Table.Ventricles.relative1.RSD  X 2/ T  a  b  l  e . V  e  n  t  r  i  c  l  e  s . r  e  l  a  t  i  v  e 1. R  S  D  X1 
 
 
 
 
 78.7 
 
 
 86.4 
 
 
 80.4 
 
 
 97.0 
 
 
 102.6 
 
 
 92.6 
 
 
 76.5 
 
 
 73.0 
 
 
 22.2 
 
 
 28.4 
 
 
 22.6 
 
 
 60.9 
 
 
 26.4 
 
 
 


 
   
  kable(Table.Ventricles.relative.stratified.gender)  
 
 
 

 
 
 
 
 
 
 
 
 
 
  
 f 
 m 
 p 
 test 
 
 
 
 
 n 
 14 
 16 
  
  
 
 
 Total.ventricular.volume.relative (mean (SD)) 
 1.8554635462 (1.3268579426) 
 1.9901807530 (1.7055469213) 
 0.813 
  
 
 
 LV.total (mean (SD)) 
 1.6208567323 (1.3239504826) 
 1.7212349160 (1.5866708182) 
 0.853 
  
 
 
 LV.frontal.horn (mean (SD)) 
 0.5647158943 (0.4689867048) 
 0.5454373384 (0.4397126405) 
 0.908 
  
 
 
 LV.body (mean (SD)) 
 0.4491770751 (0.4459306767) 
 0.4340493115 (0.4257907279) 
 0.925 
  
 
 
 LV.atrium (mean (SD)) 
 0.4293273395 (0.3142686102) 
 0.5617861603 (0.6437228077) 
 0.490 
  
 
 
 LV.occipital.horn (mean (SD)) 
 0.1112826435 (0.1145831457) 
 0.0886956685 (0.0685889424) 
 0.512 
  
 
 
 LV.temporal.horn (mean (SD)) 
 0.0663469710 (0.0375726047) 
 0.0912829346 (0.0751259289) 
 0.271 
  
 
 
 Third.ventricle (mean (SD)) 
 0.0826068089 (0.0357482716) 
 0.1220114630 (0.0957687648) 
 0.158 
  
 
 
 Fourth.ventricle.total (mean (SD)) 
 0.1520088916 (0.0289166876) 
 0.1469400629 (0.0373186717) 
 0.684 
  
 
 
 Apex (mean (SD)) 
 0.0150095263 (0.0043100506) 
 0.0149598516 (0.0043363600) 
 0.975 
  
 
 
 Lateral.recess (mean (SD)) 
 0.0190608542 (0.0040699140) 
 0.0175551609 (0.0041862858) 
 0.328 
  
 
 
 Obex (mean (SD)) 
 0.0174969331 (0.0102282698) 
 0.0146247369 (0.0093911238) 
 0.429 
  
 
 
 Fastigium (mean (SD)) 
 0.0189991246 (0.0050327628) 
 0.0177132608 (0.0047435307) 
 0.477 
  
 
 
 


 
   
  kable(Table.Ventricles.relative.stratified.gender.RSD.female)  
 
 
 

 
 
 
 
 
 
 Table.Ventricles.relative.stratified.gender.RSD.female  X 2/ T  a  b  l  e . V  e  n  t  r  i  c  l  e  s . r  e  l  a  t  i  v  e . s  t  r  a  t  i  f  i  e  d . g  e  n  d  e  r . R  S  D . f  e  m  a  l  e  X1 
 
 
 
 
 71.5 
 
 
 81.7 
 
 
 83.0 
 
 
 99.3 
 
 
 73.2 
 
 
 103.0 
 
 
 56.6 
 
 
 43.3 
 
 
 19.0 
 
 
 28.7 
 
 
 21.4 
 
 
 58.5 
 
 
 26.5 
 
 
 


 
   
  kable(Table.Ventricles.relative.stratified.gender.RSD.male)  
 
 
 

 
 
 
 
 
 
 Table.Ventricles.relative.stratified.gender.RSD.male  X 2/ T  a  b  l  e . V  e  n  t  r  i  c  l  e  s . r  e  l  a  t  i  v  e . s  t  r  a  t  i  f  i  e  d . g  e  n  d  e  r . R  S  D . m  a  l  e  X1 
 
 
 
 
 85.7 
 
 
 92.2 
 
 
 80.6 
 
 
 98.1 
 
 
 114.6 
 
 
 77.3 
 
 
 82.3 
 
 
 78.5 
 
 
 25.4 
 
 
 29.0 
 
 
 23.8 
 
 
 64.2 
 
 
 26.8 
 
 
 


 
   
  NA  
 
 
 
 
 
 
  
names.anatomical.structures.temporary <- c("LV.total",
"LV.frontal.horn",
"LV.body", 
"LV.atrium", 
"LV.occipital.horn", 
"LV.temporal.horn", 
"Third.ventricle", 
"Fourth.ventricle.total",
"Apex",
"Lateral.recess",
"Obex",
"Fastigium")

names.anatomical.structures.definitive <- c("LV- Total",
"LV - Frontal horn",
"LV - Body", 
"LV - Atrium", 
"LV - Occipital horn", 
"LV - Temporal horn", 
"Third ventricle", 
"Fourth ventricle - Total",
"Apex",
"Lateral recess",
"Obex",
"Fastigium")

Ventricles.relative.plotdata <- gather(Ventricles.relative, "anatomical.structure", "relative.volume")
Ventricles.relative.plotdata$Gender <- All.Volumes$Gender
Ventricles.relative.plotdata$Age <- All.Volumes$`Age (years)`

Ventricles.relative.plotdata$Gender <- factor(Ventricles.relative.plotdata$Gender, levels = c("f", "m"), c("f", "m"))
Ventricles.relative.plotdata$anatomical.structure <- factor(Ventricles.relative.plotdata$anatomical.structure, 
                                                                levels = rev(c(names.anatomical.structures.temporary)), rev(c(names.anatomical.structures.definitive)))

Ventricles.relative1.plot <-  ggplot(Ventricles.relative.plotdata, aes(x=anatomical.structure, y = relative.volume))  +
  stat_summary(alpha = 0.3, fun = mean, geom = "bar", width = 0.3, fill = "gray50") + 
  geom_boxplot(aes(fill = Gender), alpha = 0.5, width = 0.4, size = 0.2, position = position_dodge(width = 0.6), 
               outlier.shape = NA, color = "gray30") +
  scale_fill_manual(values = c("chartreuse4", "orangered2")) +
  geom_quasirandom(aes(color = Age), size = 0.7, alpha = 0.8, shape = 16, position = "dodge") +
  scale_color_continuous(low = "steelblue1", high = "red4") +
  xlab("") + ylab("Relative volume (in %)") +
  theme_minimal() +
  coord_flip() +
  ggtitle("VENTRICULAR SYSTEM") +
  theme(plot.title = element_text(hjust = 0.5))

Ventricles.relative1.plot
ggsave("Ventricles.relative1.plot.pdf", plot = Ventricles.relative1.plot, width = 12, height = 5, units = "in", dpi = 600)
  
 
 
   
 
 
 
 
 
 
  
Ventricles.relative$Gender <- All.Volumes$Gender
Ventricles.relative$Age <- All.Volumes$`Age (years)`
Ventricles.relative$Total.ventricular <- Total.ventricular.volume.relative

Relative.Total.ventricular.Age.plot <-  ggplot(Ventricles.relative, aes(y=Total.ventricular, x = Age))  +
  geom_point(aes(color = Gender), size = 1.5, alpha = 1, shape = 16) +
  scale_color_manual(values = c("chartreuse4", "orangered2")) +
  geom_smooth(method='lm', alpha = 0.2, colour = "dodgerblue4", size = 0.8, weight = 0.3) +
  geom_smooth(aes(color = Gender), method='lm', se = F, alpha = 0.2, linetype = "longdash", size = 0.3, weight = 0.3) +
  stat_cor(method = "pearson", label.y = 8.2, label.x = 70, color = "dodgerblue4") +
  ylab("Relative Volume (in %)") + xlab("Age (in years)") +
  theme_minimal() +
  ggtitle("RELATIVE VOLUME total ventricular system (E)") +
  theme(plot.title = element_text(hjust = 0.5))
Relative.Total.ventricular.Age.plot
ggsave("Relative.Total.ventricular.Age.plot.pdf", plot = Relative.Total.ventricular.Age.plot, width = 8, height = 6, units = "in", dpi = 600)  
 
 
   
 
 
  
Relative.LV.total.Age.plot <-  ggplot(Ventricles.relative, aes(y=LV.total, x = Age))  +
  geom_point(aes(color = Gender), size = 1.5, alpha = 1, shape = 16) +
  scale_color_manual(values = c("chartreuse4", "orangered2")) +
  geom_smooth(method='lm', alpha = 0.2, colour = "dodgerblue4", size = 0.8, weight = 0.3) +
  geom_smooth(aes(color = Gender), method='lm', se = F, alpha = 0.2, linetype = "longdash", size = 0.3, weight = 0.3) +
  stat_cor(method = "pearson", label.y = 7.2, label.x = 70, color = "dodgerblue4") +
  ylab("Relative Volume (in %)") + xlab("Age (in years)") +
  theme_minimal() +
  ggtitle("RELATIVE VOLUME lateral ventricles (E)") +
  theme(plot.title = element_text(hjust = 0.5))
Relative.LV.total.Age.plot
ggsave("Relative.LV.total.Age.plot.pdf", plot = Relative.LV.total.Age.plot, width = 8, height = 6, units = "in", dpi = 600)  
 
 
   
 
 
  
Relative.LV.frontal.horn.Age.plot <-  ggplot(Ventricles.relative, aes(y=LV.frontal.horn, x = Age))  +
  geom_point(aes(color = Gender), size = 1.5, alpha = 1, shape = 16) +
  scale_color_manual(values = c("chartreuse4", "orangered2")) +
  geom_smooth(method='lm', alpha = 0.2, colour = "dodgerblue4", size = 0.8, weight = 0.3) +
  geom_smooth(aes(color = Gender), method='lm', se = F, alpha = 0.2, linetype = "longdash", size = 0.3, weight = 0.3) +
  stat_cor(method = "pearson", label.y = 1.9, label.x = 70, color = "dodgerblue4") +
  ylab("Relative Volume (in %)") + xlab("Age (in years)") +
  theme_minimal() +
  ggtitle("RELATIVE VOLUME frontal horn (E)") +
  theme(plot.title = element_text(hjust = 0.5))
Relative.LV.frontal.horn.Age.plot
ggsave("Relative.LV.frontal.horn.Age.plot.pdf", plot = Relative.LV.frontal.horn.Age.plot, width = 8, height = 6, units = "in", dpi = 600)  
 
 
   
 
 
  
Relative.LV.body.Age.plot <-  ggplot(Ventricles.relative, aes(y=LV.body, x = Age))  +
  geom_point(aes(color = Gender), size = 1.5, alpha = 1, shape = 16) +
  scale_color_manual(values = c("chartreuse4", "orangered2")) +
  geom_smooth(method='lm', alpha = 0.2, colour = "dodgerblue4", size = 0.8, weight = 0.3) +
  geom_smooth(aes(color = Gender), method='lm', se = F, alpha = 0.2, linetype = "longdash", size = 0.3, weight = 0.3) +
  stat_cor(method = "pearson", label.y = 1.9, label.x = 70, color = "dodgerblue4") +
  ylab("Relative Volume (in %)") + xlab("Age (in years)") +
  theme_minimal() +
  ggtitle("RELATIVE VOLUME body (E)") +
  theme(plot.title = element_text(hjust = 0.5))
Relative.LV.body.Age.plot
ggsave("Relative.LV.body.Age.plot.pdf", plot = Relative.LV.body.Age.plot, width = 8, height = 6, units = "in", dpi = 600)  
 
 
   
 
 
  
Relative.LV.atrium.Age.plot <-  ggplot(Ventricles.relative, aes(y=LV.atrium, x = Age))  +
  geom_point(aes(color = Gender), size = 1.5, alpha = 1, shape = 16) +
  scale_color_manual(values = c("chartreuse4", "orangered2")) +
  geom_smooth(method='lm', alpha = 0.2, colour = "dodgerblue4", size = 0.8, weight = 0.3) +
  geom_smooth(aes(color = Gender), method='lm', se = F, alpha = 0.2, linetype = "longdash", size = 0.3, weight = 0.3) +
  stat_cor(method = "pearson", label.y = 2.4, label.x = 70, color = "dodgerblue4") +
  ylab("Relative Volume (in %)") + xlab("Age (in years)") +
  theme_minimal() +
  ggtitle("RELATIVE VOLUME atrium (E)") +
  theme(plot.title = element_text(hjust = 0.5))
Relative.LV.atrium.Age.plot
ggsave("Relative.LV.atrium.Age.plot.pdf", plot = Relative.LV.atrium.Age.plot, width = 8, height = 6, units = "in", dpi = 600)  
 
 
   
 
 
  
Relative.LV.occipital.horn.Age.plot <-  ggplot(Ventricles.relative, aes(y=LV.occipital.horn, x = Age))  +
  geom_point(aes(color = Gender), size = 1.5, alpha = 1, shape = 16) +
  scale_color_manual(values = c("chartreuse4", "orangered2")) +
  geom_smooth(method='lm', alpha = 0.2, colour = "dodgerblue4", size = 0.8, weight = 0.3) +
  geom_smooth(aes(color = Gender), method='lm', se = F, alpha = 0.2, linetype = "longdash", size = 0.3, weight = 0.3) +
  stat_cor(method = "pearson", label.y = 0.37, label.x = 70, color = "dodgerblue4") +
  ylab("Relative Volume (in %)") + xlab("Age (in years)") +
  theme_minimal() +
  ggtitle("RELATIVE VOLUME occipital horn (E)") +
  theme(plot.title = element_text(hjust = 0.5))
Relative.LV.occipital.horn.Age.plot
ggsave("Relative.LV.occipital.horn.Age.plot.pdf", plot = Relative.LV.occipital.horn.Age.plot, width = 8, height = 6, units = "in", dpi = 600)  
 
 
   
 
 
  
Relative.LV.temporal.horn.Age.plot <-  ggplot(Ventricles.relative, aes(y=LV.temporal.horn, x = Age))  +
  geom_point(aes(color = Gender), size = 1.5, alpha = 1, shape = 16) +
  scale_color_manual(values = c("chartreuse4", "orangered2")) +
  geom_smooth(method='lm', alpha = 0.2, colour = "dodgerblue4", size = 0.8, weight = 0.3) +
  geom_smooth(aes(color = Gender), method='lm', se = F, alpha = 0.2, linetype = "longdash", size = 0.3, weight = 0.3) +
  stat_cor(method = "pearson", label.y = 0.28, label.x = 70, color = "dodgerblue4") +
  ylab("Relative Volume (in %)") + xlab("Age (in years)") +
  theme_minimal() +
  ggtitle("RELATIVE VOLUME temporal horn (E)") +
  theme(plot.title = element_text(hjust = 0.5))
Relative.LV.temporal.horn.Age.plot
ggsave("Relative.LV.temporal.horn.Age.plot.pdf", plot = Relative.LV.temporal.horn.Age.plot, width = 8, height = 6, units = "in", dpi = 600)  
 
 
   
 
 
  
Relative.Third.ventricle.Age.plot <-  ggplot(Ventricles.relative, aes(y=Third.ventricle, x = Age))  +
  geom_point(aes(color = Gender), size = 1.5, alpha = 1, shape = 16) +
  scale_color_manual(values = c("chartreuse4", "orangered2")) +
  geom_smooth(method='lm', alpha = 0.2, colour = "dodgerblue4", size = 0.8, weight = 0.3) +
  geom_smooth(aes(color = Gender), method='lm', se = F, alpha = 0.2, linetype = "longdash", size = 0.3, weight = 0.3) +
  stat_cor(method = "pearson", label.y = 0.47, label.x = 70, color = "dodgerblue4") +
  ylab("Relative Volume (in %)") + xlab("Age (in years)") +
  theme_minimal() +
  ggtitle("RELATIVE VOLUME third ventricle (E)") +
  theme(plot.title = element_text(hjust = 0.5))
Relative.Third.ventricle.Age.plot
ggsave("Relative.Third.ventricle.Age.plot.pdf", plot = Relative.Third.ventricle.Age.plot, width = 8, height = 6, units = "in", dpi = 600)  
 
 
   
 
 
  
Relative.Fourth.ventricle.total.Age.plot <-  ggplot(Ventricles.relative, aes(y=Fourth.ventricle.total, x = Age))  +
  geom_point(aes(color = Gender), size = 1.5, alpha = 1, shape = 16) +
  scale_color_manual(values = c("chartreuse4", "orangered2")) +
  geom_smooth(method='lm', alpha = 0.2, colour = "dodgerblue4", size = 0.8, weight = 0.3) +
  geom_smooth(aes(color = Gender), method='lm', se = F, alpha = 0.2, linetype = "longdash", size = 0.3, weight = 0.3) +
  stat_cor(method = "pearson", label.y = 0.26, label.x = 70, color = "dodgerblue4") +
  ylab("Relative Volume (in %)") + xlab("Age (in years)") +
  theme_minimal() +
  ggtitle("RELATIVE VOLUME fourth ventricle (E)") +
  theme(plot.title = element_text(hjust = 0.5))
Relative.Fourth.ventricle.total.Age.plot
ggsave("Relative.Fourth.ventricle.total.Age.plot.pdf", plot = Relative.Fourth.ventricle.total.Age.plot, width = 8, height = 6, units = "in", dpi = 600)
  
 
 
   
 
 
 
 
 
 Relative Volumes 2: Normalized to Total Ventricular Volume 
 
 
 
  
#Ventricles.absolute <- select(Ventricles.absolute, - c(Gender))
#Total.ventricular.volume.relative <- (100 * (Ventricles.absolute$Total.volume.ventricles/All.Volumes$`Total encephalic volume (without ventricles)`))

Ventricles.relative.V <- (100 * (Ventricles.absolute[, -1]/Total.volume.ventricles))

Table.Ventricles.relative.V <- Ventricles.relative.V

Table.Ventricles.relative.V$Gender <- All.Volumes$Gender

Table.Ventricles.relative.V.1 <- CreateTableOne(
  vars = c("LV.total",
"LV.frontal.horn",
"LV.body", 
"LV.atrium", 
"LV.occipital.horn", 
"LV.temporal.horn", 
"Third.ventricle", 
"Fourth.ventricle.total",
"Apex",
"Lateral.recess",
"Obex",
"Fastigium"),
  data = Table.Ventricles.relative.V)

Table.Ventricles.relative.V.stratified.gender <- CreateTableOne(
  vars = c("LV.total",
"LV.frontal.horn",
"LV.body", 
"LV.atrium", 
"LV.occipital.horn", 
"LV.temporal.horn", 
"Third.ventricle", 
"Fourth.ventricle.total",
"Apex",
"Lateral.recess",
"Obex",
"Fastigium"),
strata = c("Gender"),
  data = Table.Ventricles.relative.V)

Table.Ventricles.relative.V.1 <- print(Table.Ventricles.relative.V.1, contDigits = 10)  
 
 
                                      
                                     Overall                     
  n                                             30               
  LV.total (mean (SD))               83.2019241187 (6.8352468610)
  LV.frontal.horn (mean (SD))        28.1033071294 (4.6967677272)
  LV.body (mean (SD))                21.1173441521 (4.9285078989)
  LV.atrium (mean (SD))              24.1985317285 (5.9615108255)
  LV.occipital.horn (mean (SD))       4.9538082192 (2.6076054839)
  LV.temporal.horn (mean (SD))        4.8294159930 (2.7406173762)
  Third.ventricle (mean (SD))         6.0195881402 (1.7866435917)
  Fourth.ventricle.total (mean (SD)) 10.7788178347 (5.5922504933)
  Apex (mean (SD))                    1.1099936692 (0.6736993658)
  Lateral.recess (mean (SD))          1.3060807781 (0.6557212781)
  Obex (mean (SD))                    1.1389316903 (0.8450359678)
  Fastigium (mean (SD))               1.3124771989 (0.6617054188)  
 
 
  Table.Ventricles.relative.V.stratified.gender <- print(Table.Ventricles.relative.V.stratified.gender, contDigits = 10)  
 
 
                                      Stratified by Gender
                                     f                            m                            p      test
  n                                             14                           16                           
  LV.total (mean (SD))               82.9014835540 (8.2210783349) 83.4648096127 (5.6208437624)  0.826     
  LV.frontal.horn (mean (SD))        28.7486156433 (4.3258459503) 27.5386621798 (5.0697128006)  0.491     
  LV.body (mean (SD))                21.5355720813 (6.0195063390) 20.7513947142 (3.9053473560)  0.671     
  LV.atrium (mean (SD))              22.9934302143 (4.6642173365) 25.2529955534 (6.8785301050)  0.309     
  LV.occipital.horn (mean (SD))       5.3475006354 (2.7639521142)  4.6093273550 (2.5007651169)  0.449     
  LV.temporal.horn (mean (SD))        4.2757800011 (1.9524248970)  5.3138474860 (3.2682007737)  0.309     
  Third.ventricle (mean (SD))         5.4244625196 (1.9402082378)  6.5403230583 (1.5129745442)  0.088     
  Fourth.ventricle.total (mean (SD)) 11.6741098633 (6.5598775626)  9.9954373097 (4.6652485061)  0.422     
  Apex (mean (SD))                    1.1864994023 (0.7826748904)  1.0430511527 (0.5799465405)  0.570     
  Lateral.recess (mean (SD))          1.4379087032 (0.7630568986)  1.1907313436 (0.5442810149)  0.311     
  Obex (mean (SD))                    1.3338238640 (0.9841474404)  0.9684010383 (0.6889767649)  0.244     
  Fastigium (mean (SD))               1.4478393571 (0.7744449436)  1.1940353105 (0.5428226641)  0.303       
 
 
 
 
 
 
  
Table.Ventricles.relative.V.1.RSD <- as.data.frame(Table.Ventricles.relative.V.1)
Table.Ventricles.relative.V.1.RSD <- data.frame(do.call('rbind', 
                                                              strsplit(as.character(Table.Ventricles.relative.V.1.RSD[-1,]),
                                                                       ' (',fixed=TRUE)))
Table.Ventricles.relative.V.1.RSD <- data.frame(cbind(str_replace_all(Table.Ventricles.relative.V.1.RSD$X1, "[ ]", ""),
                                                            str_replace_all(Table.Ventricles.relative.V.1.RSD$X2, "[)]", "")))
Table.Ventricles.relative.V.1.RSD$X1 <- as.character(Table.Ventricles.relative.V.1.RSD$X1)
Table.Ventricles.relative.V.1.RSD$X2 <- as.character(Table.Ventricles.relative.V.1.RSD$X2)
Table.Ventricles.relative.V.1.RSD <- as.data.frame(sapply(Table.Ventricles.relative.V.1.RSD, as.numeric))
Table.Ventricles.relative.V.1.RSD <- as.data.frame(Table.Ventricles.relative.V.1.RSD$X2/Table.Ventricles.relative.V.1.RSD$X1)
Table.Ventricles.relative.V.1.RSD <- round(Table.Ventricles.relative.V.1.RSD * 100, 1)


Table.Ventricles.relative.V.stratified.gender.RSD <- as.data.frame(Table.Ventricles.relative.V.stratified.gender)
Table.Ventricles.relative.V.stratified.gender.RSD <- select(Table.Ventricles.relative.V.stratified.gender.RSD, - c(p, test))

Table.Ventricles.relative.V.stratified.gender.RSD.female <- data.frame(do.call('rbind', 
                                                              strsplit(as.character(Table.Ventricles.relative.V.stratified.gender.RSD[-1, "f"]),
                                                                       ' (',fixed=TRUE)))
Table.Ventricles.relative.V.stratified.gender.RSD.female <- data.frame(cbind(str_replace_all(Table.Ventricles.relative.V.stratified.gender.RSD.female$X1, "[ ]", ""),
                                                            str_replace_all(Table.Ventricles.relative.V.stratified.gender.RSD.female$X2, "[)]", "")))
Table.Ventricles.relative.V.stratified.gender.RSD.female$X1 <- as.character(Table.Ventricles.relative.V.stratified.gender.RSD.female$X1)
Table.Ventricles.relative.V.stratified.gender.RSD.female$X2 <- as.character(Table.Ventricles.relative.V.stratified.gender.RSD.female$X2)
Table.Ventricles.relative.V.stratified.gender.RSD.female <- as.data.frame(sapply(Table.Ventricles.relative.V.stratified.gender.RSD.female, as.numeric))
Table.Ventricles.relative.V.stratified.gender.RSD.female <- as.data.frame(Table.Ventricles.relative.V.stratified.gender.RSD.female$X2/Table.Ventricles.relative.V.stratified.gender.RSD.female$X1)
Table.Ventricles.relative.V.stratified.gender.RSD.female <- round(Table.Ventricles.relative.V.stratified.gender.RSD.female * 100, 1)

Table.Ventricles.relative.V.stratified.gender.RSD.male <- data.frame(do.call('rbind', 
                                                              strsplit(as.character(Table.Ventricles.relative.V.stratified.gender.RSD[-1, "m"]),
                                                                       ' (',fixed=TRUE)))
Table.Ventricles.relative.V.stratified.gender.RSD.male <- data.frame(cbind(str_replace_all(Table.Ventricles.relative.V.stratified.gender.RSD.male$X1, "[ ]", ""),
                                                            str_replace_all(Table.Ventricles.relative.V.stratified.gender.RSD.male$X2, "[)]", "")))
Table.Ventricles.relative.V.stratified.gender.RSD.male$X1 <- as.character(Table.Ventricles.relative.V.stratified.gender.RSD.male$X1)
Table.Ventricles.relative.V.stratified.gender.RSD.male$X2 <- as.character(Table.Ventricles.relative.V.stratified.gender.RSD.male$X2)
Table.Ventricles.relative.V.stratified.gender.RSD.male <- as.data.frame(sapply(Table.Ventricles.relative.V.stratified.gender.RSD.male, as.numeric))
Table.Ventricles.relative.V.stratified.gender.RSD.male <- as.data.frame(Table.Ventricles.relative.V.stratified.gender.RSD.male$X2/Table.Ventricles.relative.V.stratified.gender.RSD.male$X1)
Table.Ventricles.relative.V.stratified.gender.RSD.male <- round(Table.Ventricles.relative.V.stratified.gender.RSD.male * 100, 1)
  
 
 
 
 
 
 
  
kable(Table.Ventricles.relative.V.1)  
 
 
 

 
 
 
  
 Overall 
 
 
 
 
 n 
 30 
 
 
 LV.total (mean (SD)) 
 83.2019241187 (6.8352468610) 
 
 
 LV.frontal.horn (mean (SD)) 
 28.1033071294 (4.6967677272) 
 
 
 LV.body (mean (SD)) 
 21.1173441521 (4.9285078989) 
 
 
 LV.atrium (mean (SD)) 
 24.1985317285 (5.9615108255) 
 
 
 LV.occipital.horn (mean (SD)) 
 4.9538082192 (2.6076054839) 
 
 
 LV.temporal.horn (mean (SD)) 
 4.8294159930 (2.7406173762) 
 
 
 Third.ventricle (mean (SD)) 
 6.0195881402 (1.7866435917) 
 
 
 Fourth.ventricle.total (mean (SD)) 
 10.7788178347 (5.5922504933) 
 
 
 Apex (mean (SD)) 
 1.1099936692 (0.6736993658) 
 
 
 Lateral.recess (mean (SD)) 
 1.3060807781 (0.6557212781) 
 
 
 Obex (mean (SD)) 
 1.1389316903 (0.8450359678) 
 
 
 Fastigium (mean (SD)) 
 1.3124771989 (0.6617054188) 
 
 
 


 
   
  kable(Table.Ventricles.relative.V.1.RSD)  
 
 
 

 
 
 
 Table.Ventricles.relative.V.1.RSD  X 2/ T  a  b  l  e . V  e  n  t  r  i  c  l  e  s . r  e  l  a  t  i  v  e . V .1. R  S  D  X1 
 
 
 
 
 8.2 
 
 
 16.7 
 
 
 23.3 
 
 
 24.6 
 
 
 52.6 
 
 
 56.7 
 
 
 29.7 
 
 
 51.9 
 
 
 60.7 
 
 
 50.2 
 
 
 74.2 
 
 
 50.4 
 
 
 


 
   
  kable(Table.Ventricles.relative.V.stratified.gender)  
 
 
 

 
 
 
 
 
 
 
 
 
 
  
 f 
 m 
 p 
 test 
 
 
 
 
 n 
 14 
 16 
  
  
 
 
 LV.total (mean (SD)) 
 82.9014835540 (8.2210783349) 
 83.4648096127 (5.6208437624) 
 0.826 
  
 
 
 LV.frontal.horn (mean (SD)) 
 28.7486156433 (4.3258459503) 
 27.5386621798 (5.0697128006) 
 0.491 
  
 
 
 LV.body (mean (SD)) 
 21.5355720813 (6.0195063390) 
 20.7513947142 (3.9053473560) 
 0.671 
  
 
 
 LV.atrium (mean (SD)) 
 22.9934302143 (4.6642173365) 
 25.2529955534 (6.8785301050) 
 0.309 
  
 
 
 LV.occipital.horn (mean (SD)) 
 5.3475006354 (2.7639521142) 
 4.6093273550 (2.5007651169) 
 0.449 
  
 
 
 LV.temporal.horn (mean (SD)) 
 4.2757800011 (1.9524248970) 
 5.3138474860 (3.2682007737) 
 0.309 
  
 
 
 Third.ventricle (mean (SD)) 
 5.4244625196 (1.9402082378) 
 6.5403230583 (1.5129745442) 
 0.088 
  
 
 
 Fourth.ventricle.total (mean (SD)) 
 11.6741098633 (6.5598775626) 
 9.9954373097 (4.6652485061) 
 0.422 
  
 
 
 Apex (mean (SD)) 
 1.1864994023 (0.7826748904) 
 1.0430511527 (0.5799465405) 
 0.570 
  
 
 
 Lateral.recess (mean (SD)) 
 1.4379087032 (0.7630568986) 
 1.1907313436 (0.5442810149) 
 0.311 
  
 
 
 Obex (mean (SD)) 
 1.3338238640 (0.9841474404) 
 0.9684010383 (0.6889767649) 
 0.244 
  
 
 
 Fastigium (mean (SD)) 
 1.4478393571 (0.7744449436) 
 1.1940353105 (0.5428226641) 
 0.303 
  
 
 
 


 
   
  kable(Table.Ventricles.relative.V.stratified.gender.RSD.female)  
 
 
 

 
 
 
 
 
 
 Table.Ventricles.relative.V.stratified.gender.RSD.female  X 2/ T  a  b  l  e . V  e  n  t  r  i  c  l  e  s . r  e  l  a  t  i  v  e . V . s  t  r  a  t  i  f  i  e  d . g  e  n  d  e  r . R  S  D . f  e  m  a  l  e  X1 
 
 
 
 
 9.9 
 
 
 15.0 
 
 
 28.0 
 
 
 20.3 
 
 
 51.7 
 
 
 45.7 
 
 
 35.8 
 
 
 56.2 
 
 
 66.0 
 
 
 53.1 
 
 
 73.8 
 
 
 53.5 
 
 
 


 
   
  kable(Table.Ventricles.relative.V.stratified.gender.RSD.male)  
 
 
 

 
 
 
 
 
 
 Table.Ventricles.relative.V.stratified.gender.RSD.male  X 2/ T  a  b  l  e . V  e  n  t  r  i  c  l  e  s . r  e  l  a  t  i  v  e . V . s  t  r  a  t  i  f  i  e  d . g  e  n  d  e  r . R  S  D . m  a  l  e  X1 
 
 
 
 
 6.7 
 
 
 18.4 
 
 
 18.8 
 
 
 27.2 
 
 
 54.3 
 
 
 61.5 
 
 
 23.1 
 
 
 46.7 
 
 
 55.6 
 
 
 45.7 
 
 
 71.1 
 
 
 45.5 
 
 
 


 
   
  NA  
 
 
 
 
 
 
  
names.anatomical.structures.temporary <- c("LV.total",
"LV.frontal.horn",
"LV.body", 
"LV.atrium", 
"LV.occipital.horn", 
"LV.temporal.horn", 
"Third.ventricle", 
"Fourth.ventricle.total",
"Apex",
"Lateral.recess",
"Obex",
"Fastigium")

names.anatomical.structures.definitive <- c("LV- Total",
"LV - Frontal horn",
"LV - Body", 
"LV - Atrium", 
"LV - Occipital horn", 
"LV - Temporal horn", 
"Third ventricle", 
"Fourth ventricle - Total",
"Apex",
"Lateral recess",
"Obex",
"Fastigium")

Ventricles.relative.V.plotdata <- gather(Ventricles.relative.V, "anatomical.structure", "relative.volume")
Ventricles.relative.V.plotdata$Gender <- All.Volumes$Gender
Ventricles.relative.V.plotdata$Age <- All.Volumes$`Age (years)`

Ventricles.relative.V.plotdata$Gender <- factor(Ventricles.relative.V.plotdata$Gender, levels = c("f", "m"), c("f", "m"))
Ventricles.relative.V.plotdata$anatomical.structure <- factor(Ventricles.relative.V.plotdata$anatomical.structure, 
                                                                levels = rev(c(names.anatomical.structures.temporary)), rev(c(names.anatomical.structures.definitive)))

Ventricles.relative2.plot <-  ggplot(Ventricles.relative.V.plotdata, aes(x=anatomical.structure, y = relative.volume))  +
  stat_summary(alpha = 0.3, fun = mean, geom = "bar", width = 0.3, fill = "gray50") + 
  geom_boxplot(aes(fill = Gender), alpha = 0.5, width = 0.4, size = 0.2, position = position_dodge(width = 0.6), 
               outlier.shape = NA, color = "gray30") +
  scale_fill_manual(values = c("chartreuse4", "orangered2")) +
  geom_quasirandom(aes(color = Age), size = 0.7, alpha = 0.8, shape = 16, position = "dodge") +
  scale_color_continuous(low = "steelblue1", high = "red4") +
  xlab("") + ylab("Relative volume (in %)") +
  theme_minimal() +
  coord_flip() +
  ggtitle("VENTRICULAR SYSTEM") +
  theme(plot.title = element_text(hjust = 0.5))

Ventricles.relative2.plot
ggsave("Ventricles.relative2.plot.pdf", plot = Ventricles.relative2.plot, width = 12, height = 5, units = "in", dpi = 600)
  
 
 
   
 
 
 
 
 
 
  
Ventricles.relative.V$Gender <- All.Volumes$Gender
Ventricles.relative.V$Age <- All.Volumes$`Age (years)`

V.Relative.LV.total.Age.plot <-  ggplot(Ventricles.relative.V, aes(y=LV.total, x = Age))  +
  geom_point(aes(color = Gender), size = 1.5, alpha = 1, shape = 16) +
  scale_color_manual(values = c("chartreuse4", "orangered2")) +
  geom_smooth(method='lm', alpha = 0.2, colour = "dodgerblue4", size = 0.8, weight = 0.3) +
  geom_smooth(aes(color = Gender), method='lm', se = F, alpha = 0.2, linetype = "longdash", size = 0.3, weight = 0.3) +
  stat_cor(method = "pearson", label.y = 102, label.x = 70, color = "dodgerblue4") +
  ylab("Relative Volume (in %)") + xlab("Age (in years)") +
  theme_minimal() +
  ggtitle("RELATIVE VOLUME lateral ventricles (V)") +
  theme(plot.title = element_text(hjust = 0.5))
V.Relative.LV.total.Age.plot
ggsave("V.Relative.LV.total.Age.plot.pdf", plot = V.Relative.LV.total.Age.plot, width = 8, height = 6, units = "in", dpi = 600)  
 
 
   
 
 
  
V.Relative.LV.frontal.horn.Age.plot <-  ggplot(Ventricles.relative.V, aes(y=LV.frontal.horn, x = Age))  +
  geom_point(aes(color = Gender), size = 1.5, alpha = 1, shape = 16) +
  scale_color_manual(values = c("chartreuse4", "orangered2")) +
  geom_smooth(method='lm', alpha = 0.2, colour = "dodgerblue4", size = 0.8, weight = 0.3) +
  geom_smooth(aes(color = Gender), method='lm', se = F, alpha = 0.2, linetype = "longdash", size = 0.3, weight = 0.3) +
  stat_cor(method = "pearson", label.y = 39, label.x = 70, color = "dodgerblue4") +
  ylab("Relative Volume (in %)") + xlab("Age (in years)") +
  theme_minimal() +
  ggtitle("RELATIVE VOLUME frontal horn (V)") +
  theme(plot.title = element_text(hjust = 0.5))
V.Relative.LV.frontal.horn.Age.plot
ggsave("V.Relative.LV.frontal.horn.Age.plot.pdf", plot = V.Relative.LV.frontal.horn.Age.plot, width = 8, height = 6, units = "in", dpi = 600)  
 
 
   
 
 
  
V.Relative.LV.body.Age.plot <-  ggplot(Ventricles.relative.V, aes(y=LV.body, x = Age))  +
  geom_point(aes(color = Gender), size = 1.5, alpha = 1, shape = 16) +
  scale_color_manual(values = c("chartreuse4", "orangered2")) +
  geom_smooth(method='lm', alpha = 0.2, colour = "dodgerblue4", size = 0.8, weight = 0.3) +
  geom_smooth(aes(color = Gender), method='lm', se = F, alpha = 0.2, linetype = "longdash", size = 0.3, weight = 0.3) +
  stat_cor(method = "pearson", label.y = 33, label.x = 70, color = "dodgerblue4") +
  ylab("Relative Volume (in %)") + xlab("Age (in years)") +
  theme_minimal() +
  ggtitle("RELATIVE VOLUME body (V)") +
  theme(plot.title = element_text(hjust = 0.5))
V.Relative.LV.body.Age.plot
ggsave("V.Relative.LV.body.Age.plot.pdf", plot = V.Relative.LV.body.Age.plot, width = 8, height = 6, units = "in", dpi = 600)  
 
 
   
 
 
  
V.Relative.LV.atrium.Age.plot <-  ggplot(Ventricles.relative.V, aes(y=LV.atrium, x = Age))  +
  geom_point(aes(color = Gender), size = 1.5, alpha = 1, shape = 16) +
  scale_color_manual(values = c("chartreuse4", "orangered2")) +
  geom_smooth(method='lm', alpha = 0.2, colour = "dodgerblue4", size = 0.8, weight = 0.3) +
  geom_smooth(aes(color = Gender), method='lm', se = F, alpha = 0.2, linetype = "longdash", size = 0.3, weight = 0.3) +
[truncated: 372,302 more chars]
