# Supplementary figures and images for: Topographic volume-standardization atlas of the human brain
Source: Brain Struct Funct. 2021 May 7;226(6):1699–711. doi: 10.1007/s00429-021-02280-1 (PMC8203509; doi:10.1007/s00429-021-02280-1)

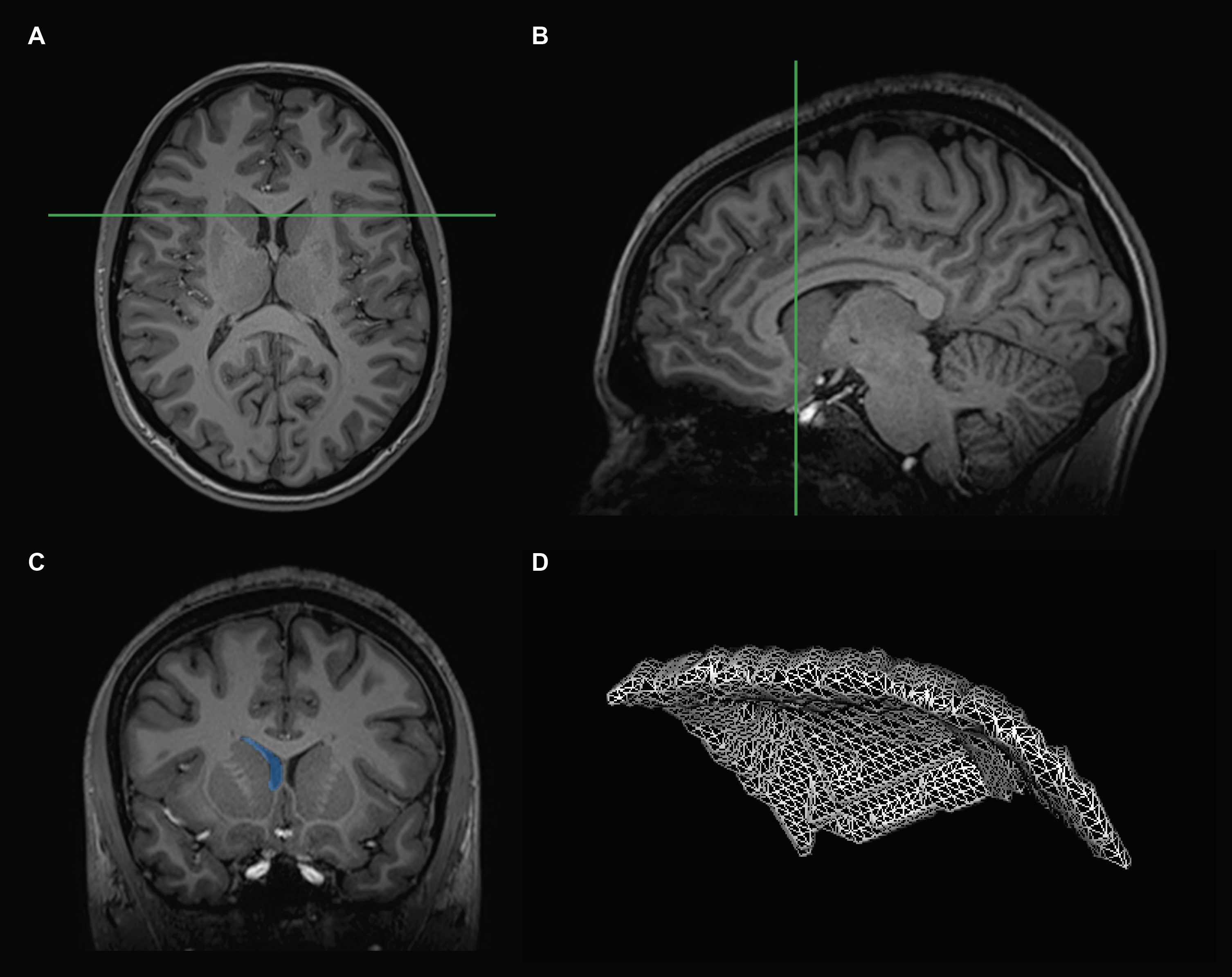

Supplement: Supplementary file 2 — Supplementary file2 (PNG 3814 KB) [file 429_2021_2280_MOESM2_ESM.png]

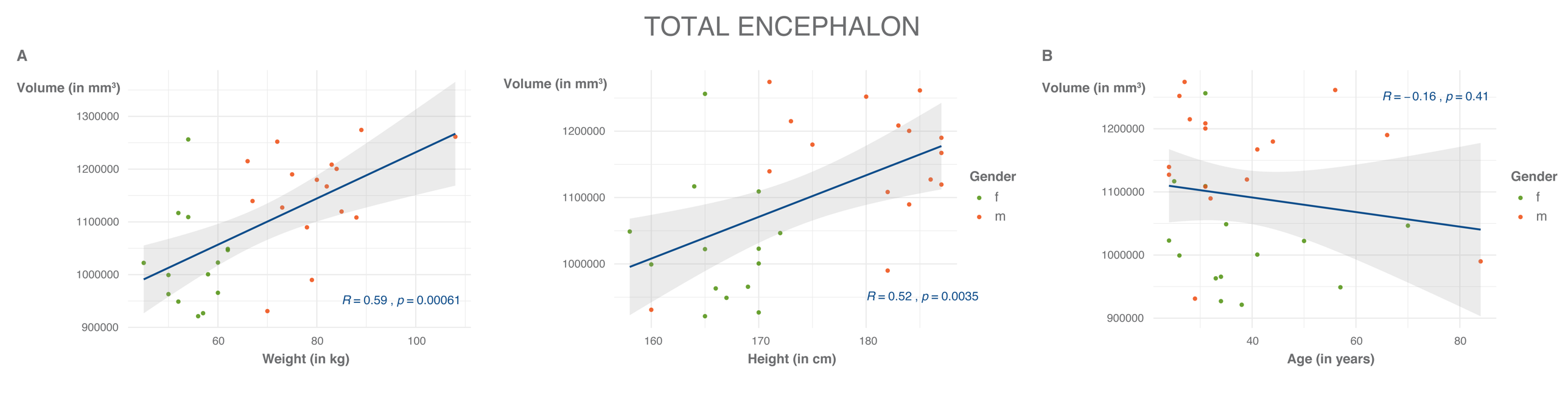

Supplement: Supplementary file 3 — Supplementary file3 (PNG 349 KB) [file 429_2021_2280_MOESM3_ESM.png]
